# Identification of π‑Stacking Motifs in Naphthalene Diimides via Solid-State NMR

**DOI:** 10.1021/jacs.5c06649

**Published:** 2025-11-04

**Authors:** Jennifer E. Mejia, Hannah E. Butler-Au, Nalaya E. Thompson, Karcher D. Goldman, Elizabeth R. Zengel, Robert D. Pike, Jingdong Mao, Craig A. Bayse

**Affiliations:** † Department of Chemistry and Biochemistry, 6042 Old Dominion University, Norfolk, Virginia 23518, United States; ‡ Department of Chemistry, 8604 College of William & Mary, Williamsburg, Virginia 23185, United States

## Abstract

Organic electronics, featuring π-conjugated small molecules and polymers, have gained significant attention for their potential in flexible, lightweight devices. However, characterization of the ordered, π-stacking domains within these materials using microscopy or X-ray diffraction (XRD) is challenging with complex systems or when crystallography is impractical. This study applied 1D ^13^C multiple cross-polarization magic angle spinning (multiCP/MAS) and 2D ^1^H–^13^C heteronuclear correlation (HetCor) solid-state nuclear magnetic resonance (ssNMR) to systematically characterize π-stacking motifs in a series of *N*,*N*′-dialkyl naphthalene diimides (NDIs). These techniques were shown to distinguish between the electronic environments attributed to different π-stacking motifs adopted in these NDIs, such that distinct packing types could be identified by their ssNMR “fingerprints” without requiring growth of XRD-quality crystals. Density functional theory (DFT) supported the experimental data by linking observed motifs with calculated chemical shifts and electronic effects due to stack-bonding interactions. These results lay the foundation for systematic ssNMR characterization of π-stacking domains in diverse organic materials, particularly in complex or blended systems where crystallography is challenging.

## Graphical abstract

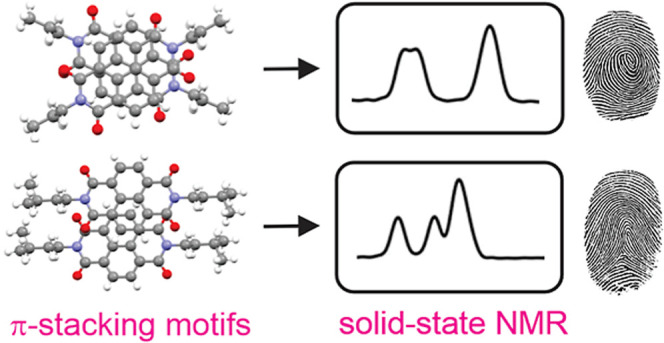

## Introduction

Organic electronic materials (OEMs) with their lighter weight and increased flexibility have the potential to overcome the physical limitations of traditional inorganic semiconductors.^1–3^ However, these materials, generally blends of π-conjugated semiconducting polymers and/or small molecules, often exhibit complex morphologies with a mix of ordered and disordered domains.^4,5^ Many interrelated factors influence morphology, including chemical composition, molecular architecture, deposition protocols, solvent selection, and thermal annealing.^6–12^ Semiconductor efficiency in these materials is closely related to the intermolecular interactions that govern the molecular order in the solid state.^13–17^ Specifically, π-stacking between adjacent molecules strengthens electronic coupling to increase efficiency.^18–23^ Conversely, disordered packing can reduce π-orbital overlap, weakening electronic coupling and diminishing performance.^24,25^ Exploring how these arrangements influence charge mobility could provide a framework for evaluating and improving morphology.^26^ However, characterizing these domains within OEMs remains challenging with conventional methods.

Techniques, such as atomic force microscopy (AFM), provide high-resolution surface images but are limited in their capacity to probe deeper layers.^27^ Powder X-ray diffraction (PXRD) is effective for analyzing crystalline domains, but amorphous regions often produce broad, poorly defined peaks that complicate data interpretation.^28^ Grazing incidence wide-angle X-ray scattering (GIWAXS) diffraction patterns contain information on crystalline domains and molecular orientation, but interpretation can rely on computational modeling.^29,30^ These limitations underscore the need for complementary, direct methods that offer insight into ordered packing arrangements within OEMs.^31,32^

Solid-state nuclear magnetic resonance (ssNMR) spectroscopy offers a powerful alternative for the characterization of complex materials.^33–35^ Unlike solution-based NMR, ssNMR directly probes the local chemical environment within the native, unaltered samples, enabling examination of a broader range of complex samples, such as blended OEMs.^34,36–38^ In the solid state, close packing restricts molecular motion such that neighboring molecules in the lattice can influence one another’s chemical environment.^39^ The sensitivity of ssNMR to the local environment has made it well-suited for examining π-stacking interactions within complex OEMs.^40–42^ In addition, prior studies on specific materials have identified slipped π-stacking motifs through ring current effects on aromatic protons in hexabenzocoronenes (HBC)^43–45^ and perylene diimides (PDIs).^46,47^

Application of ssNMR methods to broader classes of molecules could help identify trends that could be applied to electronic properties or device design considerations. This study takes advantage of the rapid measurement times of 1D ^13^C multiple cross-polarization magic angle spinning (multiCP/MAS)^48^ and 2D ^1^H–^13^C heteronuclear correlation (HetCor)^49,50^ spectroscopy to systematically examine a series of naphthalene diimides (NDIs, Scheme 1), a well-characterized class of rylene dyes with demonstrated application as n-type semiconductor materials in small-molecule and polymeric OEMs.^51–62^ *N*,*N*′-dialkyl NDIs were selected for this study because they are known to aggregate into ordered π-stacked layers, even when dropcast into thin films.^57^ The single fused-ring system and versatile packing behavior of NDIs make them ideal prototypes for systematic ssNMR studies that seek to identify ordered domains within OEMs. Our results show that, despite the common molecular structure, local electronic environments attributed to variations in the NDI π-stacking motif could be detected through “fingerprints” in the 1D and 2D ssNMR spectra. These features also enable the identification of π-stacking motifs in related newly synthesized NDIs, validated by single-crystal XRD. DFT calculations are included to assign peaks and interpret the observed π-stacking motifs in terms of orbital interactions. These results underscore ssNMR’s ability to detect variations in molecular packing and potentially provide a foundation for the systematic analysis of structure–property relationships in more complex systems.

**Scheme 1:**
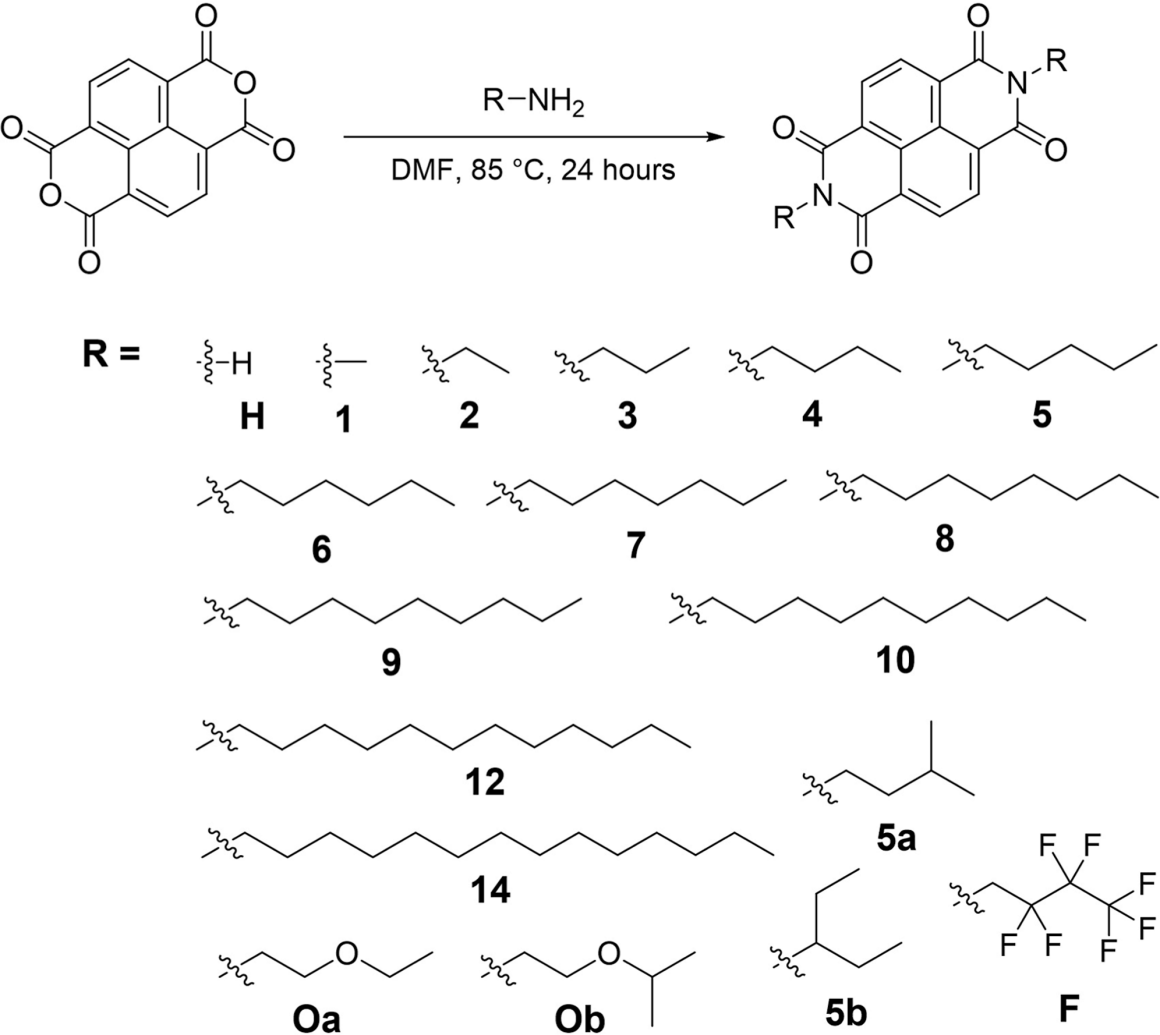
Synthesis of *N*,*N*′-dialkyl 1,4,5,8-Naphthalenediimides (NDIs) (See Supporting Information for Details)

## Results and Discussion

The series of NDIs in Scheme 1 were synthesized via imidization of naphthalene tetracarboxylic dianhydride in a pressure vessel using modified literature procedures (see Supporting Information).^63,64^ The ssNMR spectra of NDIs were collected using samples as isolated during the purification process. Single crystals were grown for XRD studies. Powder X-ray diffraction (PXRD) was performed on a representative subset of these materials. The experimental diffractograms of the bulk powders were in agreement with the patterns calculated from their corresponding single-crystal structures, confirming the representativeness of the single-crystal data. Furthermore, quantitative analysis of the PXRD data, including calculation of a crystallinity index (CI) and analysis of diffraction peak widths (fwhm at ∼15° 2θ), confirmed that the samples were predominately crystalline (83–97%) consistent with results obtained from ssNMR (see Supporting Information for full analysis).

### Comparative 1D NMR Analysis of NDIs: Insights from Solution and Solid-State Spectra

In solution, translational and rotational freedom minimizes orientation-dependent effects such as chemical shift anisotropy (CSA) such that equivalent nuclei, defined by the molecular symmetry, appear at the same chemical shift. For the series of *N*,*N*′-di-*n*-alkyl NDIs, spectra consisted of one aromatic and *n* alkyl resonances in ^1^H NMR, and three aromatic, one carbonyl, and *n* alkyl resonances in ^13^C NMR (see Supporting Information).^65^ For **NDI8** as a representative example , the four equivalent aromatic protons formed a singlet at 8.8 ppm. Three aromatic ^13^C resonances were assigned as nonprotonated carbons (npCs, 126.6 and 126.7 ppm, Figure 1 inset). The protonated carbon (pC) was found near 130.9 ppm with the carbonyl carbons at 162.8 ppm (See Supporting Information).

**Figure 1:**
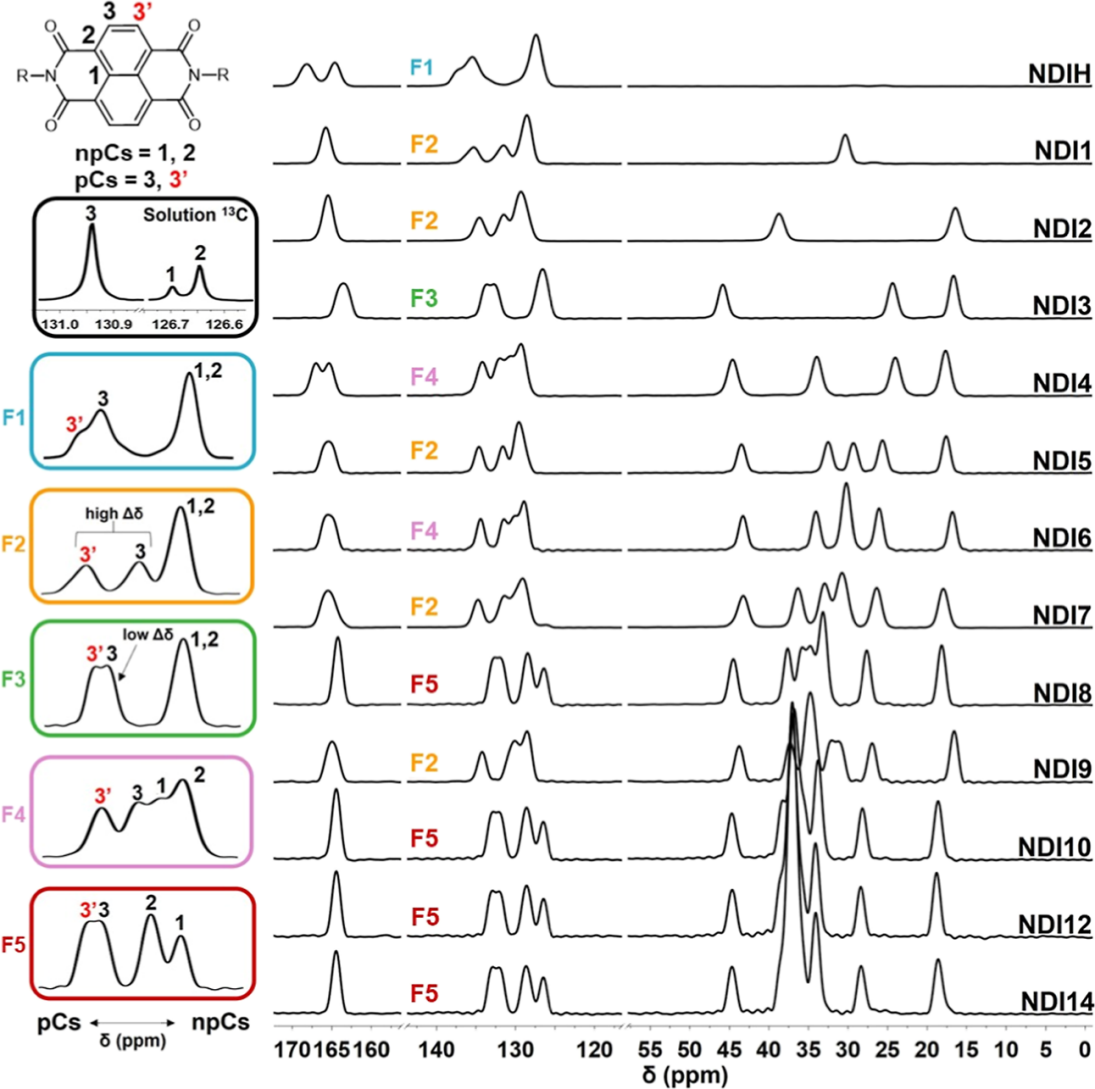
Comparison of solution phase ^13^C NMR (black inset) and ^13^C multiCP/MAS ssNMR for a series of *N*,*N*′-di-*n*-alkyl naphthalene diimides. **NDIH** and **NDIn** *n* = 1–14 (Scheme 1). The solution spectrum shows three aromatic carbons, labeled 1, 2, and 3. In ssNMR spectra, a fourth aromatic carbon (labeled 3′, red) emerges due to solid-state packing. Protonated carbons (pCs) are labeled as 3 (black) and 3′ (red), and nonprotonated carbons (npCs) as 1 and 2 (black). Protonated carbons were determined through dipolar dephasing applied to the ^13^C multiCP/MAS pulse sequence to produce spectra with suppressed pC signals (see Supporting Information). NDIs are grouped by spectral similarity into five “fingerprints”: F1 (teal), F2 (orange), F3 (green), F4 (pink), and F5 (red). These insets are aligned to facilitate pattern recognition, focusing on relative spectral features rather than absolute chemical shifts.

In the solid state, the space group symmetry of the crystal lattice defines the equivalency of the nuclei. Consequently, packing restricts molecular motion and introduces intermolecular interactions that can produce broadened or split resonances in solid-state NMR spectra. In aromatic systems such as NDIs, chemical shifts can be influenced by ring current effects^66^ within a π-stacked material. For example, splitting of aromatic resonances in hexabenzocoronene (HBC) derivatives was attributed to π-stacking and reduced symmetry in the solid state.^43–45^ For the series of *N*,*N*′-di-*n*-alkyl NDIs (Figure 1), the ^13^C multiCP/MAS ssNMR spectra had a comparable number of alkyl carbon peaks (∼12–55 ppm) to their solution spectra (e.g., four peaks for **NDI4** in both), except when resonances overlapped in long chain derivatives. Alkyl ^13^C chemical shifts in solution were upfield relative to ssNMR (Table 1, Supporting Information), due to the presence of gauche and trans conformers in the alkyl chains of free, solvated NDIs.^67^ However, the aromatic regions (∼120–145 ppm) varied in peak shape, chemical shift, and resolution. Notably, the signals for pCs, determined by dipolar dephasing (see Supporting Information), are split in some spectra, indicating changes to the local chemical environment for the packed molecules (Figure 1).

**Table 1:** Summary of 1D and 2D ssNMR and XRD Data for **NDIH** and **NDIn** (*n* = 1–10, 12, 14), Including the 1D Fingerprint Classification, 2D Pattern Observed, ^13^C Chemical Shifts and Δδ Values for the Non-Protonated (npC) and Protonated (pC) Aromatic Carbons, Chemical Shifts of Carbonyl Carbons (C=O), Single-Crystal Bulk Packing Mode, and Inter-Ring Stack and Slip Distances

	1D ^13^C multiCP MAS NMR						XRD^a^				2D NMR
NDI	1D fingerprint	δ npC,^b^ ppm	Δδ	δ pC,^c^ ppm	Δδ	δ C=O, ppm	symmetry	bulk packing	d_stack_	d_slip_	2D pattern
**H**	F1	127.36	0	135.42, 137.36	1.94	164.61, 168.15	*P*-1	unidirectional	3.34	3.00	Ia
**1**	F2	128.50	0	131.35, 135.22	3.87	165.73	*P*2_1_/*c*	bidirectional	3.34	3.20	IIa
**2**	F2	129.34	0	131.46, 134.51	3.05	165.45	*P*2_1_/*c*	bidirectional	3.26	3.61	IIa
**3**	F3	126.43	0	132.49, 133.48	0.99	163.27	*Pbca*	zigzag	3.35	0.97	Ib
**4**	F4	129.27, 130.55	1.28	131.93, 134.15	2.22	165.34, 166.98	*P*-1	unidirectional	3.30	4.05	IIa
**5**	F2	129.54	0	131.56, 134.57	3.01	165.30	*P*2_1_/*n*	bidirectional	3.25	3.83	IIa
**6**	F4	128.90, 130.12	1.22	131.45, 134.39	2.94	165.46	*P*-1	unidirectional	3.26	3.47	IIa
**7**	F2	129.08	0	131.36, 134.72	3.36	165.46	*P*2_1_/*n*	bidirectional	3.24	3.54	IIa
**8**	F5	126.52, 128.56	2.04	132.00, 132.75	0.75	165.43	*P*-1	unidirectional	3.31	3.19	IIb
**9**	F2	128.52	0	130.06, 134.20	4.14	164.95	*P*2_1_/*c*	bidirectional	3.29	3.57	IIa
**10**	F5	126.49, 128.59	2.10	132.09, 132.78	0.69	164.43	*P*-1	unidirectional	3.29	3.42	IIb
**12**	F5	126.46, 128.58	2.18	132.07, 132.89	0.82	164.32	*P*-1	unidirectional	3.30	3.22	IIb
**14**	F5	126.48, 128.64	2.16	132.06, 132.85	0.79	164.45	*P*-1	unidirectional	3.33	3.22	IIb

a Δδ_npC_ = δ_npC2_–δ_npC1_.

b ${\Delta \delta }_{pC}=\delta_{pC3^{\prime }}-\delta_{pC3}$

c See Supporting Information for references and details.

The NDIs were grouped by common patterns or “fingerprints” in the aromatic region (Table 1). **NDIH** (F1) was most similar to the solution spectrum with a single upfield npC peak (127.4 ppm) and two downfield pC resonances with a moderate Δδ of 2 ppm. **NDI1**, **NDI2**, and longer odd-chain NDIs (*n* = 5, 7, 9) (F2) have a single npC resonance with well-separated pC peaks (Δδ > 3 ppm). **NDI3** (F3) had an upfield npC signal and two pC resonances with modest resolution (Δδ < 1 ppm). **NDI4** and **NDI6** (F4) had four signals with two npC resonances (Δδ ≈1 ppm) and two more resolved downfield pC peaks (Δδ = 2–3 ppm). The npC resonances for long, even-chain NDIs (*n* = 8, 10, 12, 14) (F5) were more upfield (126–129 ppm) compared to other NDIs with a small Δδ (approximately 2 ppm) between pC resonances. The carbonyl carbon signals also varied with splitting found in **NDIH** and **NDI4** and sharper peaks in long, even-chain derivatives. Given the influence of molecular packing on ssNMR chemical shifts, these observed groups of patterns may identify NDIs with similar π-stacking motifs.

### Crystallographic Packing Motifs of NDIs

The review of X-ray data from the Cambridge Crystallographic Database or from freshly grown single crystals confirmed that NDIs adopted several common bulk packing patterns, predominantly forming either unidirectional (triclinic space group, *P*-1) or bidirectional (monoclinic, *P*2_1_/*c* or P2_1_/*n*) columns (Table 1 and Figure 2). Most NDIs form π-stacking interactions with their aromatic cores parallel displaced along the column axis, similar to the offset (Bernal-type) arrangement in graphite (Figure 3). The alkyl groups of **NDI4**, **NDI5**, and **NDI6** each pack with at least one C–C bond in a gauche conformation. All longer alkyl chains are fully extended with a slight twist in the C–C bond closest to the NDI of 157° to 172°. Comparison of the XRD data to the 1D ssNMR spectra demonstrated that NDIs with similar fingerprints also shared crystallographic features and packing arrangements.

**Figure 2:**
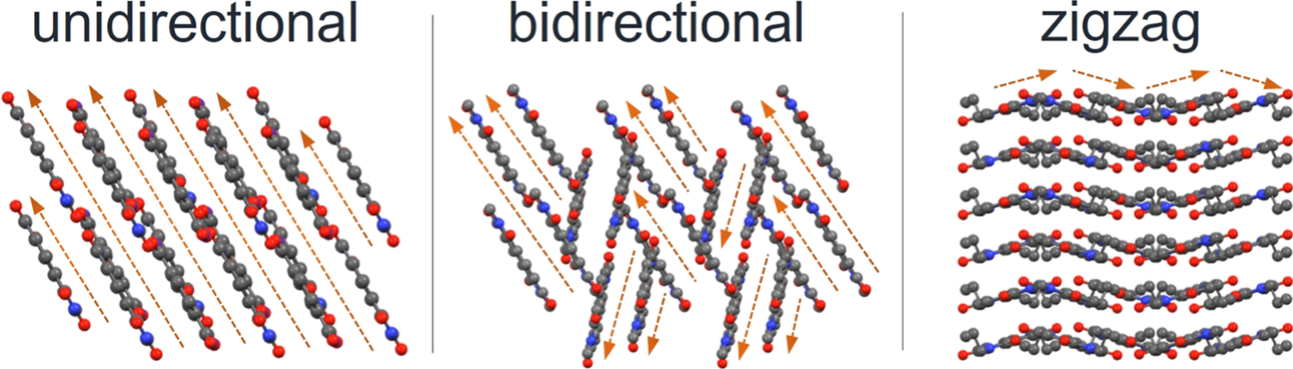
Bulk packing motifs for various NDIs (unidirectional packing, bidirectional packing with staggered alignment, and zigzag packing). Structures are shown with color-coded atoms (oxygen, red; nitrogen, blue; carbon, gray; hydrogen, white). Arrows indicate the stacking direction within each arrangement.

**Figure 3:**
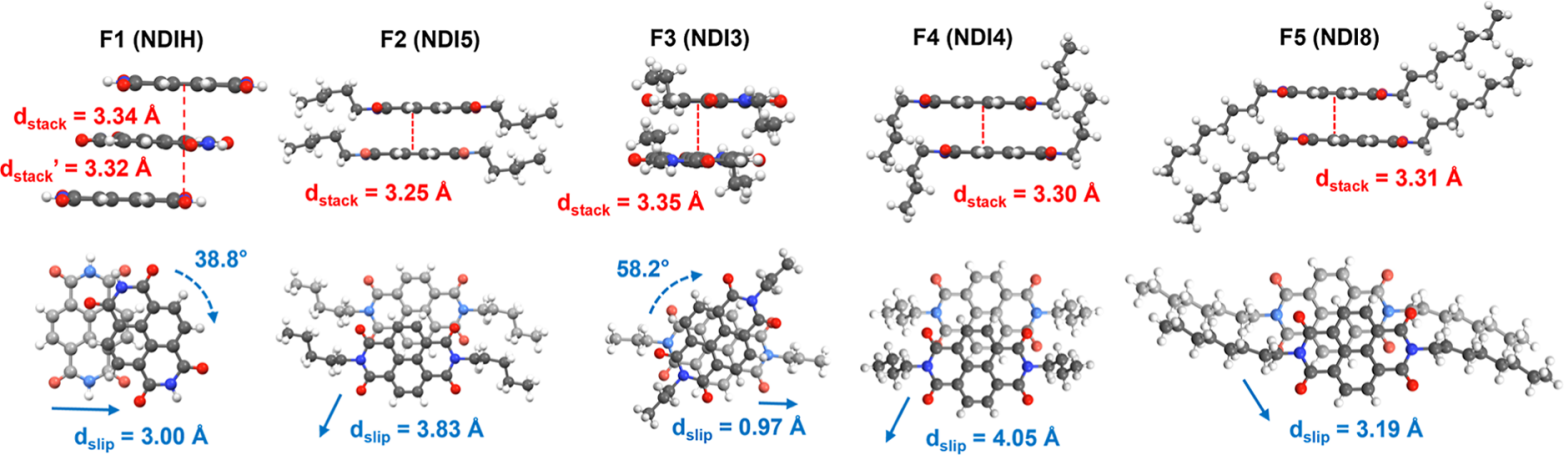
Representative structural and intermolecular interaction analysis of NDIs in various packing configurations corresponding to the five 1D ssNMR fingerprints: F1 (**NDIH**); **F2** (**NDIn** (*n* = 1, 2, 5, 7, and 9)); **F3** (**NDI3**); **F4** (**NDI4** and **NDI6**); **F5** (**NDIn** (*n* = 8, 10, 12, and 14)). Each fingerprint’s panel depicts the side profiles of stacking arrangements and interplanar separations (d_stack_) in the crystal structure and top-down views of aromatic molecular arrangements show the slip distance (d_slip_). Hashed and solid arrows indicate twist and slip directions, respectively.

The patterns F1 (**NDIH**) and F3 (**NDI3**) were both examples in which the single-crystal X-ray structures showed π-stacking interactions that were parallel-displaced and twisted with respect to the column axis. **NDIH** crystallized in the *P*-1 triclinic space group with unidirectional packing and minor variations in interplanar spacing (*d*_stack_ = 3.34 Å and *d*’_stack_ = 3.32 Å). Unlike the di-*n*-alkyl NDIs, its offset parallel-displaced and twisted π-stacking motif does not adopt graphite-like AB packing. **NDI3** crystallized in the *Pbca* orthorhombic space group with minimal parallel displacement (*d*_slip_ = 0.97 Å) of the π-stacked NDI cores which are twisted by ∼58° within the columns, bringing seven ring carbons into close contact. Unlike other NDIs, where R-groups aligned in a single direction, the propyl groups alternated orientations between adjacent molecules, which were packed into zigzag sheets (Figure 2). The Hirshfeld fingerprint plot had a greater population of C/C contacts, consistent with the larger π–π overlap of this motif, which likely contributed to the upfield ^13^C pC chemical shifts relative to other NDIs (See Supporting Information).

For NDIs with 1D ssNMR patterns F2 and F4, the aromatic cores overlap with close contacts between two outer edge ring carbons, stabilized by electrostatic interactions between opposing carbonyls. NDIs with pattern F2 (**NDI1**, **NDI2**, odd numbered alkyl chains ≥5) adopt a monoclinic (*P*2_1_/*c* or P2_1_/*n*) space group with bidirectional packing. In **NDI5**, the alkyl chains were twisted and curled where those of **NDI7** and **NDI9** are fully extended. **NDI11**, not included in this work, has similar packing to **NDI9** ^65^ and would be predicted to have an F2 fingerprint in its 1D ssNMR spectrum. In contrast, **NDI4** and **NDI6** with pattern F4 were found in the *P*-1 triclinic space group with unidirectional packing. **NDI6** has slight variations in cell dimensions and angles with comparable close contacts and interaction patterns to **NDI4** despite having twisted R-groups with significant terminal disorder. Although the π-stacking of the NDI cores is similar to NDIs with F2 fingerprints, the different orientation of the R-group and the unidirectional packing shifts npC_2_ slightly downfield relative to npC_1_.

NDIs with F5 fingerprints have greater core–core overlap with close contacts between four aromatic carbons. NDIs with long, even numbered alkyl chains (*n* ≥8) packed with fully extended R-groups in the *P*-1 triclinic space group with unidirectional packing and increased inter-ring overlap versus groups F2 and F4, consistent with the shielded electronic environments observed in the 1D ssNMR spectra.

Within these grouping of common 1D fingerprints, the π-stacking distances (*d*_stack_ = 3.24–3.35 Å (Table 1) are shorter than the sum of the van der Waals distances and contribute to the detectable local magnetic field differences observed in the aromatic chemical shift patterns in 1D ssNMR spectra. Hirshfeld surface analysis of the 3D distribution of intermolecular interactions for the NDI core also produced similar surfaces and fingerprint plots within the five groups of ^13^C multiCP/MAS patterns (see Supporting Information).^68–70^

### ^1^H–^13^C Heteronuclear Correlation (HetCor) 2D ssNMR

^1^H–^13^C 2D HetCor NMR investigates the correlations between proton- and carbon-13 nuclei, revealing how each is influenced by its local chemical environment. The spectra for the series of NDIs were grouped by the patterns in their aromatic proton environments designated I and II based upon the observed splitting of the ^1^H resonance. For 2D pattern I, a single ^1^H resonance is observed for both protons, and in pattern II, two proton environments emerge due to their proximity to electron-rich and electron-poor π-density regions of adjacent molecules. These patterns were further categorized based on variations in electronic shielding for the carbon environment (Figure 4). Peak shapes varied with crystallinity. NDIs with high crystallinity indices (**NDI3**, **NDI4**, **NDI8**) showed sharp, well-resolved aromatic and aliphatic peaks while precipitates with lower crystallinity indices (**NDIH**, **NDI1**, **NDI2**, **NDI5**, **NDI6**, **NDI7**, **NDI9**, **NDI10**, **NDI12**, and **NDI14**) exhibited broadening at the base of these peaks, consistent with disordered components and split alkyl signals that reflect local conformational variations (see Supporting Information).

**Figure 4:**
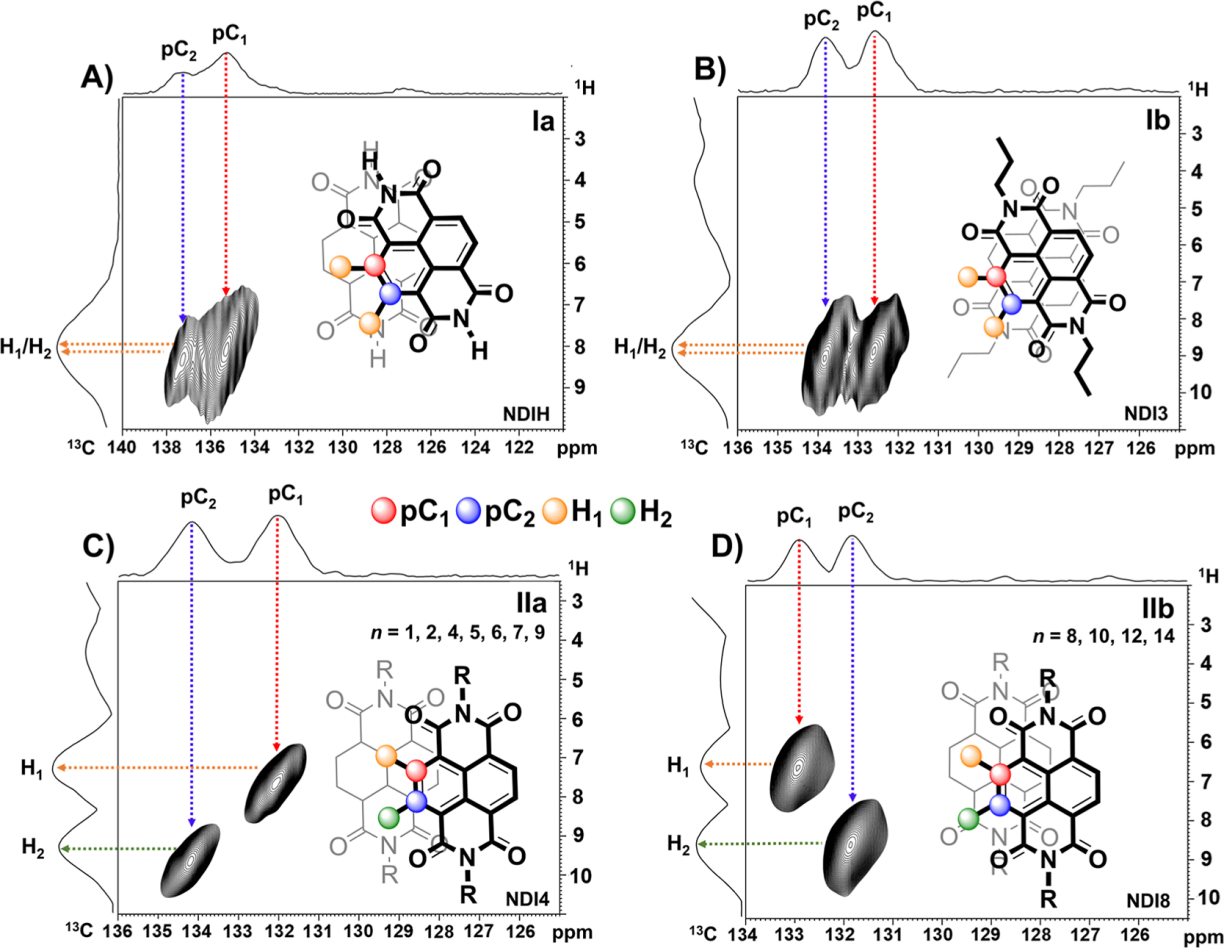
Representative 2D ^1^H–^13^C HetCor spectra of straight chain NDIs display complex correlation patterns due to shielding effects characteristic of their π-stacking arrangements. NDIs with an unresolved ^1^H peak are designated with I, those with split ^1^H resonances are II. These designations are further modified with a or b, based upon whether the ^13^C signal is split: (A) **NDIH** (Ia); (B) **NDI3** (Ib); (C) NDI withs F2 and F4 1D fingerprints (IIa, **NDI4** shown); and (D) NDIs with F5 1D fingerprints (IIb, **NDI8** shown).

A single ^1^H signal was observed for **NDIH** (Ia) and **NDI3** (Ib) due to similar local chemical environments for their aromatic protons despite the asymmetry of the twist along the packing axis. For **NDIH**, the pCs had distinct, broadened ^13^C resonances due to differential shielding by the naphthalene and diimide π-clouds of the adjacent molecule (Figure 4A) and increased disordered components due to its CI of 83%. **In NDI3**, nearly equivalent chemical environments were found for both protons, despite H_2_ being positioned closer to the imide nitrogen and H_1_ over the carbonyl. The splitting of the pC ^13^C resonances reflects the positioning of one pC within the ring current of an adjacent unit (Figure 4B).

For spectra in which the aromatic protons are resolved, the NDIs with 1D fingerprints F2 and F4 have their more shielded aromatic proton correlated with the more shielded pC (2D pattern IIa, Figure 4C). For example, in **NDI4** (Figure 4C), the ^13^C shifts at 132.1 ppm (pC_1_) and 134.2 ppm (pC_2_) correlate with ^1^H shifts at 7.9 ppm (H_1_) and 8.8 ppm (H_2_), respectively. Based on GIAO–DFT NMR calculations of the NDI dimer extracted from the X-ray structure, the H_1_ proton is shielded by stacking over the adjacent naphthyl ring, while H_2_ is centered over a less charge-dense imide ring. Likewise, the more shielded pC_1_ environment aligns with regions of greater π-electron density. Despite the differences in bulk packing between NDIs with 1D fingerprints F2 and F4, the 2D HetCor spectra show that the parallel-displaced π-stacking motif was similar between the groups. In addition, while the disorder of the **NDI6** alkyl groups observed in its X-ray structure contributed to splitting of the crosspeaks in 2D ssNMR, its overall IIa pattern was similar to other NDIs with F2 and F4 fingerprints (see Supporting Information). NDIs that pack in a similar fashion, such as alkylamine and cyclohexylamine derivatives, should also have IIa patterns in 2D HetCor ssNMR.^71,72^

In contrast, NDIs with 1D “fingerprint” F5 have their less shielded proton correlated with more shielded pC (2D pattern IIb, Figure 4D). For example, in **NDI8**, ^13^C shifts at 131.9 (pC_2_) and 132.9 ppm (pC_1_) correlated with proton shifts at 9.1 ppm (H_2_) and 7.8 ppm (H_1_), respectively. In GIAO–DFT NMR calculations of the dimer, the more shielded pC_2_ is positioned over the center of the imide ring, with its proton (H_2_) less shielded due to its proximity to the partially positive carbonyl carbon. Conversely, pC_1_ is deshielded by the interaction with the lower electron density at the partially positive central carbon of the adjacent NDI unit. Its proton (H_1_) is shielded by the ring current at the center of a naphthalene ring. The contrast between these correlation patterns in 2D HetCor spectra further demonstrates how molecular stacking arrangements lead to distinct chemical environments that can be detected through ssNMR.

### DFT Calculations

Parallel-displaced packing has been shown to enhance orbital overlap and strengthen electronic coupling.^23^ These π-stacking motifs result from the mixing of π-type monomer molecular orbitals (MOs) within the stack (Figure 5, see Supporting Information for computational details).^73–75^ While a fully eclipsed, or sandwich, conformation would be repulsive due to the two-orbital-four-electron (2o4e) interaction, the parallel-displaced and/or twisted conformations reduce these repulsions by distorting to a conformation where there is favorable overlap between the lobes of the π-type monomer MOs. The stack bonding model for π–π interactions is defined in analogy to MO theory for covalent bonds, using linear combinations of monomer MOs to construct the dimer MOs.^73–75^ Like the bond order of MO theory, the stack bond order (SBO) is the difference between the number of stack bonding (SB) and stack antibonding (SA) dimer MOs. π-Stacked dimers were extracted from the X-ray structures of Ib-, IIa-, and IIb-patterned NDIs, adjusted to a propyl group, and optimized at the DFT­(M06–2X-D3)/TZVP^76–78^ level to inspect the character of the dimer MOs. The frontier dialkyl NDI monomer MOs are analogous to the a_u_- and b_3u_-type MOs of **NDIH** and form additive (+) and subtractive (−) linear combinations with SA and SB character, respectively, at the S conformation. The equal number of SB and SA dimer MOs cancels for an SBO of zero, predicting no stacking in the fully eclipsed S conformation. Parallel displacement and/or twisting of the dimer, such as that observed in the NDIs, shifts the (+)-type dimer MOs from SA to SB while maintaining the SB character of the (−) dimer MOs for a nonzero SBO and a reduction in the 2o4e repulsions resulting in a stable π-stacking conformation. The increase in overlap of monomer MOs in the dimer is also consistent with the interpenetration of π-density that contributes to electronic mobility through OEMs. The splitting of the HOMO and LUMO pairs of the dimer can be used to estimate the hole and electron mobilities, respectively, for each of the π-stacking motifs.^79^ The twisted Ib motif had similar values for both HOMO and LUMO splittings (0.170 and 0.176 eV, respectively), suggesting comparable electron and hole mobilities. The parallel-displaced IIa motif had lower splitting values (0.077 and 0.067 eV, respectively), suggesting NDIs with this π-stacking would have lower hole and electron mobilities than Ib. The IIb motif is predicted to have a high electron mobility (0.219 eV), but a low hole mobility (0.023 eV). These wide ranges in predicted mobility suggest that materials favoring specific motifs would vary in performance.

**Figure 5:**
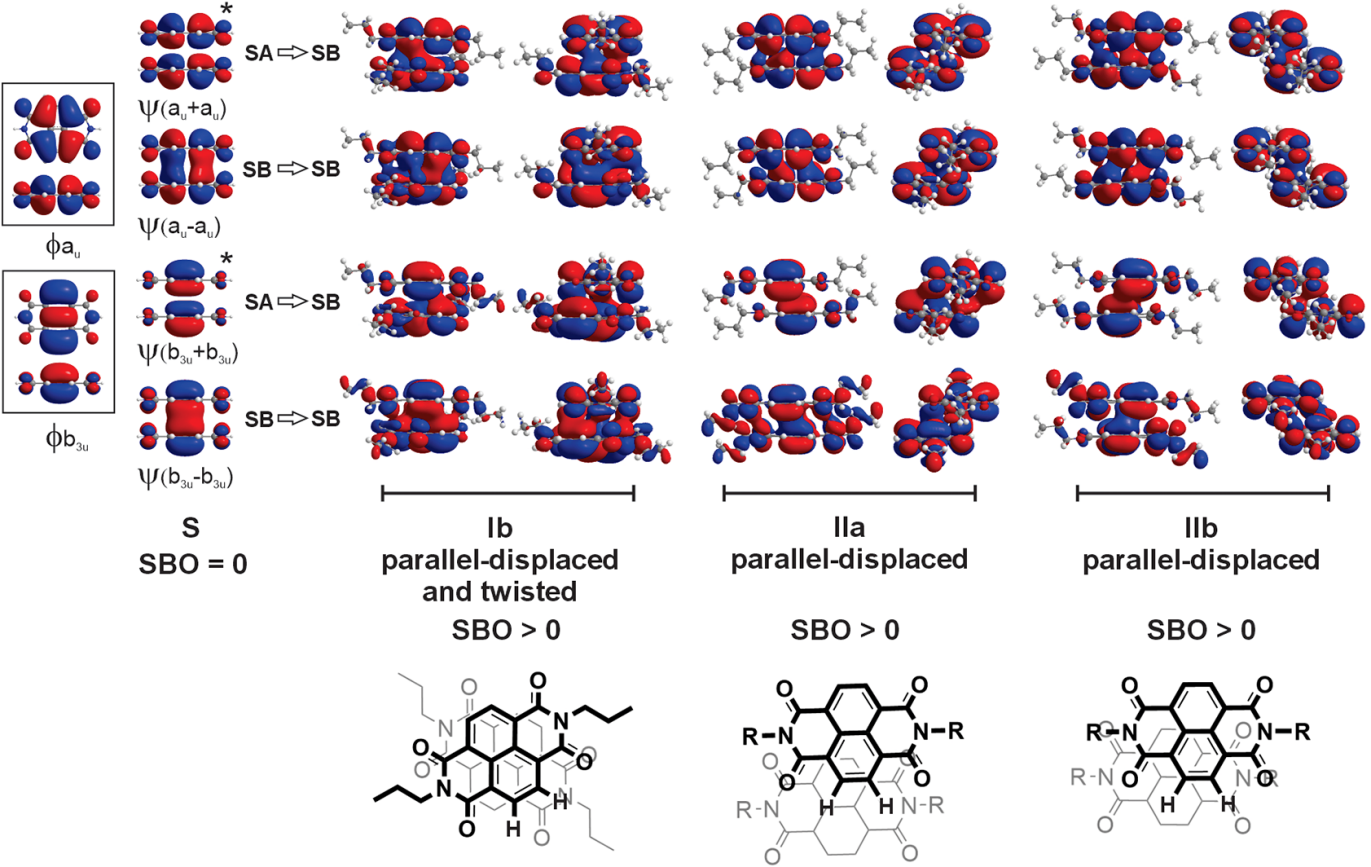
Stack bonding interactions in the Ib, IIa, and IIb conformations of **NDI3** viewed along the *x*- and *y*-axes. The equal numbers of stack bonding (SB) and stack antibonding (SA) linear combinations of the Frontier monomer MOs cancels in the fully eclipsed S conformation of **NDIH** (SBO = 0). In the parallel-displaced conformations, the MOs with SA character at S become SB for a favorable, nonzero SBO.

The conformation of the NDI within the lattice depends upon both the strength of the π-stacking interaction and the interactions between the imide R-groups. DFT­(M06–2X-D3)/TZVP relative energies of the Ib, IIa, and IIb conformations were calculated for the series **NDI2**–**NDI8** (Table 2) using dimers extracted from X-ray structures with alkyl groups modeled as straight chains. [Note: **NDI1** was omitted because it optimized only to the Ib and IIb conformations]. For **NDI3**, the twisted conformation of Ib, which has the most overlap of the monomer MO lobes, is the most stable conformation. The *E*_int_ of this conformation is constant with the length of the R-groups, which point in opposite directions in the motif. In contrast, the IIa and IIb conformations are stabilized as the dispersion interactions between the chains become favorable at longer chain lengths. Although the IIa dimer is more stable, crystal packing forces not accounted for in these gas-phase calculations will determine the observed bulk packing mode. In the lattice, steric interactions between short R-groups favor IIa where there is the least spatial overlap between the NDI cores. The dispersion interactions between the longer chains favor the more closely overlapped cores of the IIb.

**Table 2:** π-Stacking Interaction Energies *E*_int_ for NDIs at the Experimental Orientations Calculated Relative to Separated Monomers at the M06-2X (M06-2X-D3) Levels

	*E*_int_, M06–2X/TZVP (M06–2X-D3/TZVP)		
	type Ib	type IIa	type IIb
**NDI2**	–21.7 (−25.4)	–15.9 (−19.2)	–15.8 (−19.2)
**NDI3**	–21.6 (−25.6)	–16.7 (−20.6)	–16.5 (−20.4)
**NDI4**	–21.8 (−26.1)	–17.8 (−22.3)	–17.2 (−21.6)
**NDI5**	–21.7 (−26.2)	–19.3 (−24.2)	–18.2 (−23.0)
**NDI6**	–21.7 (−26.2)	–20.8 (−26.2)	–19.1 (−24.4)
**NDI7**	–21.7 (−26.2)	–22.4 (−28.2)	–20.2 (−25.9)
**NDI8**	–21.7 (−26.3)	–24.0 (−30.3)	–21.2 (−27.3)

### Branched and Substituted NDIs

A series of branched (**NDI5a**, **NDI5b**), ether-substituted (**NDIOa**, **NDIOb**), and fluorinated (**NDIF**) NDIs were examined with both 1D and 2D ssNMR techniques (Figures 6 and 7, Table 3). These NDIs provide a valuable contrast to the straight-chain variants, demonstrating how more complex substitutions influence both the solid-state organization and their corresponding NMR signatures. **NDI5a, NDIOa, and NDIOb** for which X-ray structures have not been previously reported (Table 3) were crystalline and had spectra similar to the short-chain NDIs of group F2 (1D ssNMR) and the type IIa (2D ssNMR) motifs (Figures 4 and 6A). However, chemical shift variations were observed, suggesting subtle differences in the local electronic environments. Specifically, both **NDIOa** and **NDIOb** showed increased separation between their npC and pC resonances. Additionally, the npC resonance of **NDI5a** was broader by approximately 1 ppm, potentially due to steric effects of its branched chain (Figure 6A). While **NDIOa** and **NDIOb** adopted monoclinic space group *P*2_1_/*n*, **NDI5a** crystallized in the triclinic *P*-1 space group with unidirectional bulk packing, a notable deviation from the characteristics of F2 NDIs. However, columnar packing of **NDI5a** was similar to F2 NDIs, resulting in the pattern IIa parallel-displaced π-stacking motif. Hirshfeld surface and fingerprint plots showed that close contacts (see Supporting Information), and intermolecular interactions were also similar to the F2 NDIs.

**Figure 6:**
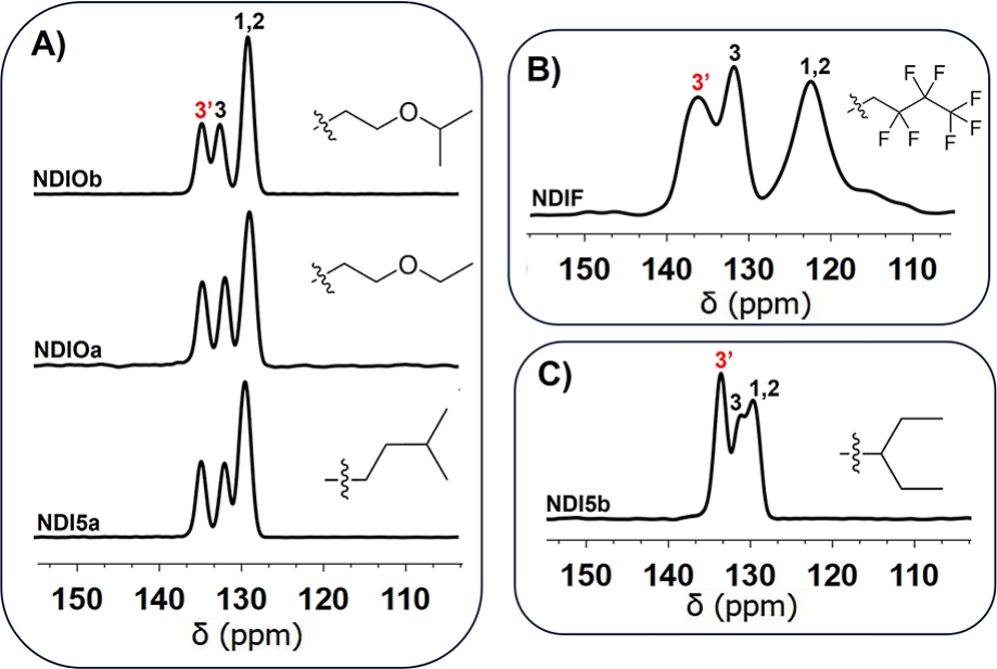
(A) 1D ^13^C multiCP/MAS spectra of the aromatic regions of the branched (**NDI5a**) and substituted (**NDIOa** and **NDIOb**) NDIs. (B) 1D ^13^C multiCP/MAS spectrum of **NDIF** showing broad pC and npC resonances attributed to deshielding from nearby fluorine atoms. (C) 1D ^13^C multiCP/MAS spectrum of **NDI5b**.

**Figure 7:**
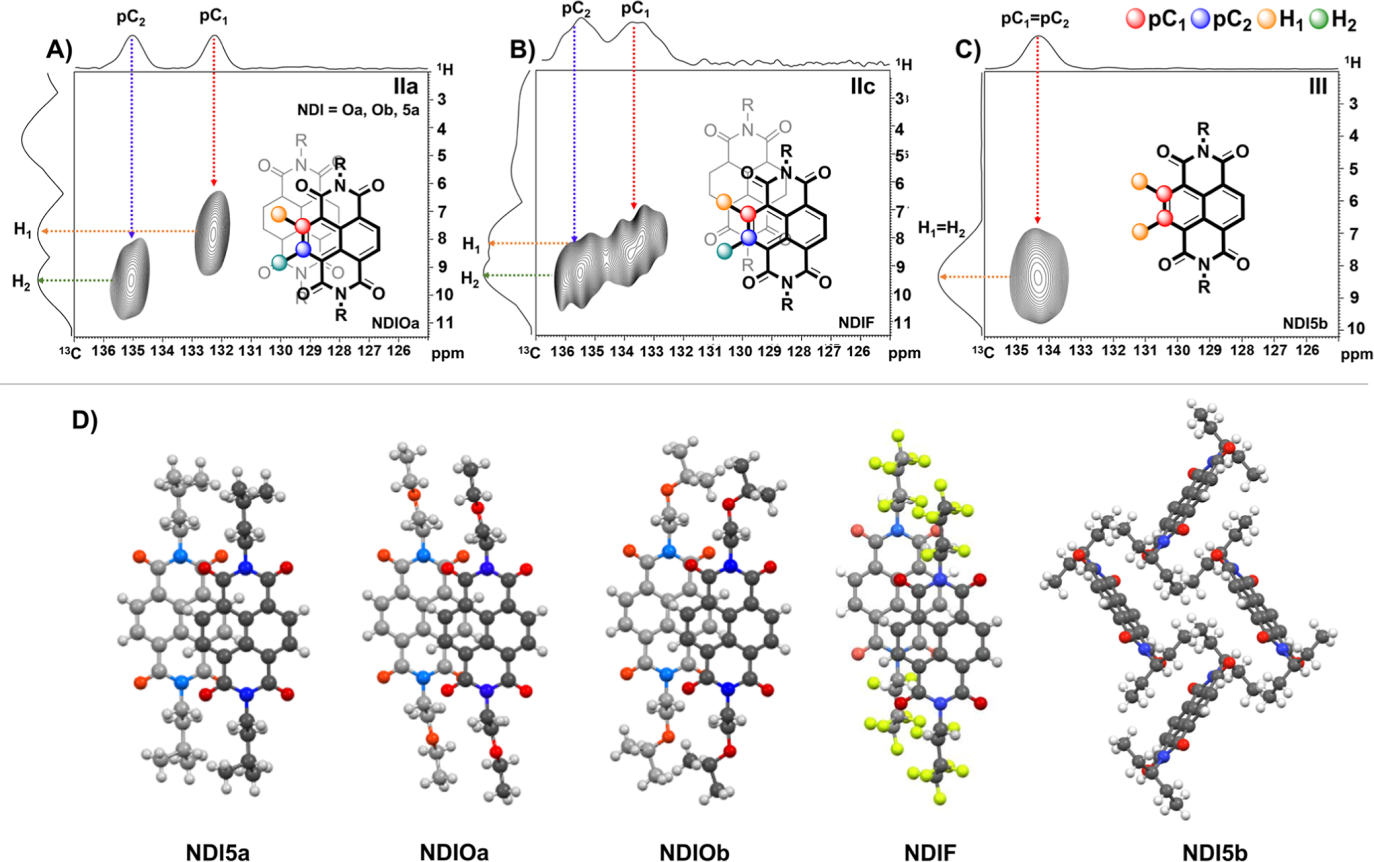
2D ^1^H–^13^C HetCor ssNMR spectra of branched and substituted NDIs. (A) **NDIOa**, **NDIOb**, and **NDI5a** (pattern IIa, parallel-displaced packing motifs) showing distinct correlation peaks for protonated carbons (pCs) and nonprotonated carbons (npCs), (B) **NDIF** (pattern IIc) displaying unique splitting due to fluorine substitution and short inter-ring close contacts within its Bernal-type parallel-displaced conformation, (C) **NDI5b** (pattern III), with a single correlation peak indicating that both protons have a similar local environment, (D) X-ray structures of **NDI5a**, **NDIOa**, **NDIOb**, **NDIF**, and **NDI5b**. Note that in **NDI5b**, the NDI units do not π-stack consistent with the single C–H correlation peak in (C).

**Table 3:** Summary of 1D and 2D ssNMR and XRD Data for **NDI5a**, **NDI5b**, **NDIOa**, and **NDIOb**, and **NDIF**, Including the 1D Fingerprint Classification, 2D Pattern Observed, ^13^C Chemical Shifts and Δδ Values for the Non-Protonated (npC) and Protonated (pC) Aromatic Carbons, Chemical Shifts of Carbonyl Carbons (C=O), Single-Crystal Bulk Packing Mode, and Inter-Ring Stack and Slip Distances

	1D ^13^C multiCP MAS NMR						XRD^a^				
NDI	1D fingerprint	δ npC,^b^ ppm	Δδ	δ pC,^c^ ppm	Δδ	δ C=O, ppm	symmetry	bulk packing	d_stack_	d_slip_	2D patterm
**5a**	**F2**	**129.09**	**0**	131.59, 134.44	**2.85**	**164.57, 165.80**	*P*-1	unidirectional	**3.25**	3.60	IIa
**5b**	**F6**	**129.65, 131.03**	**1.38**	133.63	**0**	**166.19, 167.26**	*P*2_1_/*c*	bidirectional	**3.23**	N/A	III
**Oa**	**F2**	**128.59**	**0**	131.52, 134.44	**2.92**	**165.38**	*P*2_1_/*n*	bidirectional	**3.23**	3.36	IIa
**Ob**	**F2**	**128.90**	**0**	132.23, 134.42	**2.19**	**164.75, 166.01**	*P*2_1_/*n*	bidirectional	**3.48**	3.43	IIa
**F**	**F7**	**119.84**	**0**	129.26, 133.50	**4.24**	**164.14**	*P*-1	unidirectional	**3.34**	3.85	IIc

a Δδ_npC_ = δ_npC2_–δ_npC1_.

b Δδ_pC_ = δ_pC3′_–δ_pC3_.

c See Supporting Information for references and details.

In contrast, **NDIF** and **NDI5b** did not fit into the patterns established for the straight-chain NDIs, indicating that these molecules adopt other packing motifs. **NDIF** had broader line widths for both pC and npC resonances due to deshielding by the electronegative fluorine atoms oriented close to the aromatic protons, resulting in a distinct ^13^C multiCP/MAS spectrum (Figure 6B). In the 2D HetCor, this resulted in additional upfield splitting of pC_1_ to two resonances at 133.4 and 133.8 ppm compared to the single resonance observed for pC_1_ in other type II NDIs (Figure 7B). In the X-ray structure, the cores are stacked in a parallel-displaced conformation with one carbonyl oxygen over the central naphthalene carbon, where aromatic Hs are either oriented over a bridgehead carbon or outside the aromatic ring system.^80^ The single ^1^H–^13^C correlation peak of **NDI5b** (Figure 7C) indicated a single chemical environment consistent with the absence of π-stacking interactions (Figure 7D). These results further demonstrate that ssNMR is highly sensitive to differences in molecular packing and underscores its potential as a primary tool for characterizing solid-state materials, even in cases where XRD is not feasible.

## Conclusions

This study demonstrates the ability of solid-state NMR (ssNMR) to distinguish between different π-stacking motifs in a series of di-*n*-alkyl *N*,*N*′-disubstituted naphthalene diimides (NDIs). Fingerprints in the aromatic region of their 1D multiCP/MAS ssNMR ^13^C spectra distinguished between variations in the bulk local environment due to the π-stacking motif adopted in solid-state molecular packing. The ^1^H–^13^C correlations in 2D HetCor ssNMR identified specific π-stacking motifs through the shielding of nuclei by the ring currents of nearby, closely packed molecules. These parallel displaced and/or twisted π-stacking motifs themselves are favored by orbital mixing between monomers, represented by a positive stack bond order, with variations in mobilities predicted for different motifs.

XRD analysis of NDI crystal structures confirmed that NDIs with matching ssNMR spectral patterns shared similar crystallographic features and had the same packing arrangements. Importantly, unlike single-crystal XRD, which requires a high-quality crystal and extensive analysis for each sample, ssNMR consistently enabled inference of packing arrangements across multiple samples, from spectral comparisons alone. Further, the patterns identified for the di-*n*-alkyl NDIs further allowed for packing motifs to be identified in newly synthesized NDIs, confirmed by single-crystal X-ray diffraction. These results underscore the value of accessible ssNMR methods for characterizing ordered π-stacked domains within OEMs where crystallography is not practical. Extension of this approach to map motif fingerprints of other OEMs offers a practical tool as straightforward as routine solution-phase NMR to probe packing domains or to guide the development of theoretical models of complex materials.

## Supplementary Material



## Notes

Experimental methods including details of NDI synthesis, solution and solid-state NMR spectra, determination of X-ray crystal structures, Hirshfeld surface analysis, DFT calculations including Cartesian coordinates of optimized structures (PDF)
