# Supplementary material for: Identification of π‑Stacking Motifs in Naphthalene Diimides via Solid-State NMR
Source: J Am Chem Soc. 2025 Nov 4;147(45):41261–71. doi: 10.1021/jacs.5c06649 (PMC12616687; doi:10.1021/jacs.5c06649)
Supplement: Supplementary file 1 [file ja5c06649_si_001.pdf]

Supporting Information for:

Identification of π-stacking Motifs in Naphthalene Diimides via Solid-state NMR

Jennifer E. Mejia[a], Hannah E. Butler-Au[a], Nalaya E. Thompson[a], Karcher D. Goldman[a], Elizabeth A. Zengel[a], Robert D. Pike[b], Jingdong Mao[a],*, Craig A. Bayse[a],*

a. Department of Chemistry and Biochemistry, Old Dominion University, Norfolk, Virginia 23518, USA

b. Department of Chemistry, College of William & Mary, Williamsburg, Virginia 23195, USA

* Address correspondence to jmao@odu.edu, cbayse@odu.edu

Experimental Methods

Synthesis of N,N'-di-*n*-alkyl naphthalene diimides (NDIs) compounds 3
General details for solution NMR, HRMS, FT-IR 3
Spectroscopic summary of NDIs 4
Solution 1D ^{1}H and ^{13}C NMR spectra 7
Solid-state NMR 25
Solid-state 1D ^{13}C multiCP/MAS and ^{13}C multiCP/MAS with dipolar dephasing spectra 25
Solid-state 2D ^{1}H -^{13}C HetCor spectra 36
Crystallography 45
Powder X-ray 47
Computational Methods 63
Hirshfeld surface analysis 63
DFT methods 68
Cartesian coordinates 68
References 120

Experimental Methods

Synthesis of N,N'-di-*n*-alkyl naphthalene diimides (NDIs) compounds

All reagents used were commercially available and purchased through Sigma Aldrich and Fisher Scientific. They were used as supplied without further purification.

Synthesis of N'N'-Diamide 1,4,5,8 naphthalenetetracarboxylic diimide (H): NDIH was prepared according to a previously published literature procedure.[1] 1,4,5,8-Naphthalenetetracarboxylic dianhydride (**NDA**) (0.51 g, 1.86 mmol) was dissolved and stirred in an ammonium hydroxide (50 mL, 28 %, w/w) aqueous solution. Then, the mixture was stirred for 6 h at room temperature and a pale-yellow product diimide precipitate was obtained after vacuum filtration.

Synthesis of N,N'-di-*n*-alkyl-naphthalene diimides: NDI1, NDI2, NDI3, NDI4, NDI5, NDI6, NDI7, NDI8, NDI9, NDI10, NDI12, NDI14, NDI5a, NDI5b, NDIOa, NDIOb, and NDIF, were prepared according to modified literature procedures.[2,3] A slurry of naphthalenetetracarboxylic dianhydride (0.5 g, 1.86 mmol) in 25 mL of N,N-dimethylformamide (DMF) was treated with 2 equivalents of primary amine. The homogenous mixture was sealed in a pressure vessel, heated at 85 °C overnight (~12 hours), and then cooled. The resulting slurry was diluted with several volumes of methanol. The precipitate was filtered, washed with cold methanol, and dried thoroughly.

General details for solution NMR, HRMS, FT-IR

HRMS (ESI) data were acquired on a Thermo Scientific Q Exactive Orbitrap mass spectrometer in positive-ion mode. Samples were prepared in methanol (1.5 mL) containing 0.1% (v/v) formic acid and directly infused at 5 µL/min with a spray voltage of 3.5 kV. The instrument resolution was 70,000 (at m/z 200), and the mass range was m/z 150–2000.

^{1}H and ^{13}C NMR spectra were acquired on a Bruker Avance III 400 spectrometer operating at a magnetic field strength of 400 MHz for ^{1}H and 100 MHz for ^{13}C. In solution NMR, samples were prepared in $CDCl_3$ and referenced to the residual solvent peak. ^{1}H NMR experiments were used to acquire the one-dimensional (1D) proton spectra using 1-second recycle delays. ^{13}C NMR experiments were used for the 1D ^{13}C spectra using 1.5 second recycle delays.

FTIR-ATR spectra were recorded on a Bruker ALPHA II FT-IR spectrometer equipped with a Platinum ATR module (ZnSe crystal). Samples were analyzed as solids and spectra were collected over a range of 4000–400 cm^{-1} with a resolution of 4 cm^{-1}. Background spectra were subtracted using OPUS software.

Spectroscopic summary of NDIs

N'N'-Diamide 1,4,5,8-naphthalenetetracarboxylic diimide (H): ^{1}H NMR (400 MHz, $CDCl_3$) δ [ppm]: 8.62 (s, 4H), 1.00 (s, 2H); ^{13}C NMR (100 MHz, DMSO-d_6) δ [ppm]: 163.82, 130.12, 128.20, 127.33. ATR-IR (cm^{-1}): 3054 ν(N-H), 1690 ν(C=O). HRMS-ESI: $C_{14}H_7N_2O_4$ $[M + H]^+$: 267.040; found: 267.040. The product obtained was a beige solid.

N,N'-Dimethyl-1,4,5,8-naphthalenetetracarboxylic diimide (1): ^{1}H NMR (400 MHz, $CDCl_3$) δ [ppm]: 8.80 (s, 4H), 3.63 (s, 6H); ^{13}C NMR (100 MHz, $CDCl_3$) δ [ppm]: 163.08, 130.97, 126.64, 126.62, 27.40. ATR-IR (cm^{-1}): 3080 ν(C-H), 1650 ν(C=O). HRMS-ESI: calcd for $C_{16}H_{11}N_2O_4$ $[M + H]^+$: 295.071; found: 295.071. The product obtained was a dark red solid.

N,N'-Diethyl-1,4,5,8-naphthalenetetracarboxylic diimide (2): ^{1}H NMR (400 MHz, $CDCl_3$) δ [ppm]: 8.79 (s, 4H), 4.31 (q, 4H), 1.39 (t, 6H); ^{13}C NMR (100 MHz, $CDCl_3$) δ [ppm]: 162.67, 130.89, 126.64, 36.06, 13.31. ATR-IR (cm^{-1}): 3090 ν(C-H), 1654 ν(C=O). HRMS-ESI: calcd for $C_{18}H_{15}N_2O_4$ $[M + H]^+$: 323.103; found: 323.104. The product obtained was a dark red solid.

N,N'-Dipropyl-1,4,5,8-naphthalenetetracarboxylic diimide (3): ^{1}H NMR (400 MHz, $CDCl_3$) δ [ppm]: 8.79 (s, 4H), 4.20 (t, 4H), 1.82 (p, 4H), 1.06 (t, 6H); ^{13}C NMR (100 MHz, $CDCl_3$) δ [ppm]: 162.88, 130.93, 126.74, 126.66, 42.42, 21.38, 11.45. ATR-IR (cm^{-1}): 3080 ν(C-H), 1670 ν(C=O). HRMS-ESI: calcd for $C_{20}H_{19}N_2O_4$ $[M + H]^+$: 351.134; found: 351.134. The product obtained was a dark red solid.

N,N'-Dibutyl-1,4,5,8-naphthalenetetracarboxylic diimide (4): ^{1}H NMR (400 MHz, $CDCl_3$) δ [ppm]: 8.78 (s, 4H), 4.25 (t, 4H), 1.76 (q, 4H, 1.50 (sext, 4H), 1.02 (t, 6H); ^{13}C NMR (100 MHz, $CDCl_3$) δ [ppm]: 162.85, 130.92, 126.72, 126.67, 40.75, 30.17, 20.33, 13.77. ATR-IR (cm^{-1}): 2940 ν(C-H), 1600 ν(C=O). HRMS-ESI: calcd for $C_{22}H_{23}N_2O_4$ $[M + H]^+$: 379.165; found: 379.166. The product obtained was a red solid.

N,N'-Dipentyl-1,4,5,8-naphthalenetetracarboxylic diimide (5): ^{1}H NMR (400 MHz, $CDCl_3$) δ [ppm]: 8.78 (s, 4H), 4.24 (t, 4H), 1.78 (q, 4H), 1.44 (m, 8H), 0.95 (t, 6H); ^{13}C NMR (100 MHz, $CDCl_3$) δ [ppm]: 162.85, 130.91, 126.72, 126.67, 40.96, 29.17, 27.75, 22.38, 13.94. ATR-IR (cm^{-1}): 2970 ν(C-H), 1620 ν(C=O). HRMS-ESI: calcd for $C_{24}H_{27}N_2O_4$ $[M + H]^+$: 407.196; found: 407.197. The product obtained was a dark pink solid.

N,N'-Dihexyl-1,4,5,8-naphthalenetetracarboxylic diimide (6): ^{1}H NMR (400 MHz, $CDCl_3$) δ [ppm]: 8.78 (s, 4H), 4.24 (t, 4H), 1.77 (q, 4H), 1.44 (m, 12H), 0.92 (t, 6H); ^{13}C NMR (100 MHz, $CDCl_3$) δ [ppm]: 162.85, 130.92, 126.73, 126.68, 40.99, 31.48, 28.03, 26.72, 22.52, 14.00. ATR-IR (cm^{-1}): 2910 ν(C-H), 1600 ν(C=O). HRMS-ESI: calcd for $C_{26}H_{31}N_2O_4$ $[M + H]^+$: 435.228; found: 435.228. The product obtained was a pink solid.

N,N'-Diheptyl-1,4,5,8-naphthalenetetracarboxylic diimide (7): ^{1}H NMR (400 MHz, $CDCl_3$) δ [ppm]: 8.78 (s, 4H), 4.22 (t, 4H), 1.77 (q, 4H), 1.45 (m, 16H), 0.91 (t, 6H); ^{13}C NMR (100 MHz, $CDCl_3$) δ [ppm]: 162.85, 130.92, 126.72, 126.67, 41.00, 31.72, 28.97, 28.09, 27.04, 22.57, 14.04. ATR-IR (cm^{-1}): 2900 ν(C-H), 1600 ν(C=O). HRMS-ESI: calcd for $C_{28}H_{35}N_2O_4$ $[M + H]^+$: 463.259; found: 463.260. The product obtained was a pastel pink solid.

N,N'-Dioctyl-1,4,5,8-naphthalenetetracarboxylic diimide (8): ^{1}H NMR (400 MHz, $CDCl_3$) δ [ppm]: 8.78 (s, 4H), 4.22 (t, 4H), 1.77 (q, 4H), 1.45 (m, 20H), 0.90 (t, 6H); ^{13}C NMR (100 MHz, $CDCl_3$) δ [ppm]: 162.84, 130.91, 126.72, 126.67, 41.00, 31.79, 29.27, 29.17, 28.09, 27.08, 22.61, 14.05. ATR-IR (cm^{-1}): 2970 ν(C-H), 1615 ν(C=O). HRMS-ESI: calcd for $C_{30}H_{39}N_2O_4$ $[M + H]^+$: 491.290; found: 491.292. The product obtained was a pastel pink solid.

N,N'-Dinonyl-1,4,5,8-naphthalenetetracarboxylic diimide (9): ^{1}H NMR (400 MHz, $CDCl_3$) δ [ppm]: 8.78 (s, 4H), 4.22 (t, 4H), 1.77 (q, 4H), 1.30 (m, 24H), 0.90 (t, 6H); ^{13}C NMR (100 MHz, $CDCl_3$) δ [ppm]: 162.85, 130.92, 126.72, 126.67, 41.00, 31.83, 29.47, 29.31, 29.23, 28.09, 27.08, 22.64, 14.07. ATR-IR (cm^{-1}): 2970 ν(C-H), 1615 ν(C=O). HRMS-ESI: calcd for $C_{32}H_{42}N_2O_4$ $[M + Na]^+$:541.303; found: 541.304. The product obtained was a pastel pink solid.

N,N'-Didecyl-1,4,5,8-naphthalenetetracarboxylic diimide (10): ^{1}H NMR (400 MHz, $CDCl_3$) δ [ppm]: 8.78 (s, 4H), 4.22 (t, 4H), 1.77 (q, 4H), 1.28 (m, 28H), 0.90 (t, 6H); ^{13}C NMR (100 MHz, $CDCl_3$) δ [ppm]: 162.84, 130.91, 126.72, 126.67, 41.00, 31.87, 29.53, 29.51, 29.31, 29.28, 28.09, 27.08, 22.66, 14.08. ATR-IR (cm^{-1}): 2920 ν(C-H), 1610 ν(C=O). HRMS-ESI: calcd for $C_{34}H_{47}N_2O_4$ $[M + H]^+$: 547.353; found: 547.354. The product obtained was a white solid.

N,N'-Didodecyl-1,4,5,8-naphthalenetetracarboxylic diimide (12): ^{1}H NMR (400 MHz, $CDCl_3$) δ [ppm]: 8.78 (s, 4H), 4.22 (t, 4H), 1.77 (q, 4H), 1.28 (m, 36H), 0.90 (t, 6H); ^{13}C NMR (100 MHz, $CDCl_3$) δ [ppm]: 162.84, 130.91, 126.72, 126.67, 41.00, 31.91, 29.62, 29.61, 29.57, 29.51, 29.32, 28.09, 27.08, 22.67, 14.09. ATR-IR (cm^{-1}): 2950 ν(C-H), 1690 ν(C=O). HRMS-ESI: calcd for $C_{38}H_{55}N_2O_4$ $[M + H]^+$: 603.415; found: 603.414. The product obtained was a white solid.

N,N'-Ditetradecyl-1,4,5,8-naphthalenetetracarboxylic diimide (14): ^{1}H NMR (400 MHz, $CDCl_3$) δ [ppm]: 8.78 (s, 4H), 4.22 (t, 4H), 1.77 (q, 4H), 1.55 (m, 44H), 0.90 (t, 6H); ^{13}C NMR (100 MHz, $CDCl_3$) δ [ppm]: 162.85, 130.91, 126.72, 126.67, 41.00, 31.91, 29.65, 29.58, 29.51, 29.31, 28.09, 27.08, 22.67, 14.09. ATR-IR (cm^{-1}): 2975 ν(C-H), 1680 ν(C=O). HRMS-ESI: calcd for $C_{42}H_{63}N_2O_4$ $[M + H]^+$: 659.478; found: 659.478. The product obtained was a white solid.

N,N'-Diisoamyl-1,4,5,8-naphthalenetetracarboxylic diimide (5A): ^{1}H NMR (400 MHz, $CDCl_3$) δ [ppm]: 8.78 (s, 4H), 4.22 (t, 4H), 1.77 (q, 4H), 1.55 (m, 44H), 0.90 (t, 6H); ^{13}C NMR (100 MHz, $CDCl_3$) δ [ppm]: 162.79, 130.89, 126.71, 126.68, 39.56, 36.85, 26.45, 22.48. ATR-IR (cm^{-1}): 2900 ν(C-H), 1600 ν(C=O). HRMS-ESI: calcd for $C_{24}H_{27}N_2O_4$ $[M + H]^+$: 407.196; found: 407.197. The product obtained was a white solid.

N,N'-Di(1-ethylpropyl)-1,4,5,8-naphthalenetetracarboxylic diimide (5B): ^{1}H NMR (400 MHz, $CDCl_3$) δ [ppm]: 8.75 (s, 4H), 5.06 (m, 2H), 2.26 (t, 4H), 1.95 (t, 4H) 0.93 (t, 12H) ; ^{13}C NMR (100 MHz, $CDCl_3$) δ [ppm]: 163.69, 130.96, 126.83, 126.65, 58.14, 24.96, 11.24. ATR-IR (cm^{-1}): 2910 ν(C-H), 1620 ν(C=O). HRMS-ESI: calcd for $C_{24}H_{27}N_2O_4$ $[M + H]^+$: 407.196; found: 407.196. The product obtained was an orange solid.

N,N'-Diethoxyethan-1,4,5,8-naphthalenetetracarboxylic diimide (OA): ^{1}H NMR (400 MHz, $CDCl_3$) δ [ppm]: 8.79 (s, 4H), 4.48 (t, 4H), 3.80 (t, 4H), 3.58 (t, 4H), 1.18 (t, 6H); ^{13}C NMR (100 MHz, $CDCl_3$) δ [ppm]: 162.90, 131.01, 126.81, 126.64, 67.02, 66.27, 39.82, 15.07. ATR-IR (cm^{-}

^{1}): 2990 ν(C-H), 1600 ν(C=O). HRMS-ESI: calcd for $C_{22}H_{23}N_2O_4$ $[M + H]^+$: 411.155; found: 411.155. The product obtained was a light blue solid.

N,N'-Diisopropoxyethan-1,4,5,8-naphthalenetetracarboxylic diimide (OB): ^{1}H NMR (400 MHz, $CDCl_3$) δ [ppm]: 8.79 (s, 4H), 4.45 (t, 4H), 3.78 (t, 4H), 3.69 (m, 2H), 1.15 (d, 12H); ^{13}C NMR (100 MHz, $CDCl_3$) δ [ppm]: 162.86, 130.99, 126.78, 126.63, 71.58, 64.23, 40.28, 21.98. ATR-IR (cm^{-1}): 2910 ν(C-H), 1610 ν(C=O). HRMS-ESI: calcd for $C_{39}H_{27}N_2O_4$ $[M + H]^+$: 439.186; found: 413.186. The product obtained was a light pink solid.

N,N'-Diheptafluorobutyl-1,4,5,8-naphthalenetetracarboxylic diimide (F): ^{1}H NMR (400 MHz, $CDCl_3$) δ [ppm]: 8.89 (s, 4H), 5.05 (t, 4H); ^{13}C NMR (100 MHz, $CDCl_3$) δ [ppm]: 162.22, 131.83, 127.01, 126.36, 38.64. ATR-IR (cm^{-1}): 2975 ν(C-H), 1600 ν(C=O). HRMS-ESI: calcd for $C_{22}H_9N_2O_4$ $[M + H]^+$: 631.033; found: 631.033. The product obtained was a pale blue solid.

Solution 1D ^{1}H and ^{13}C NMR spectra

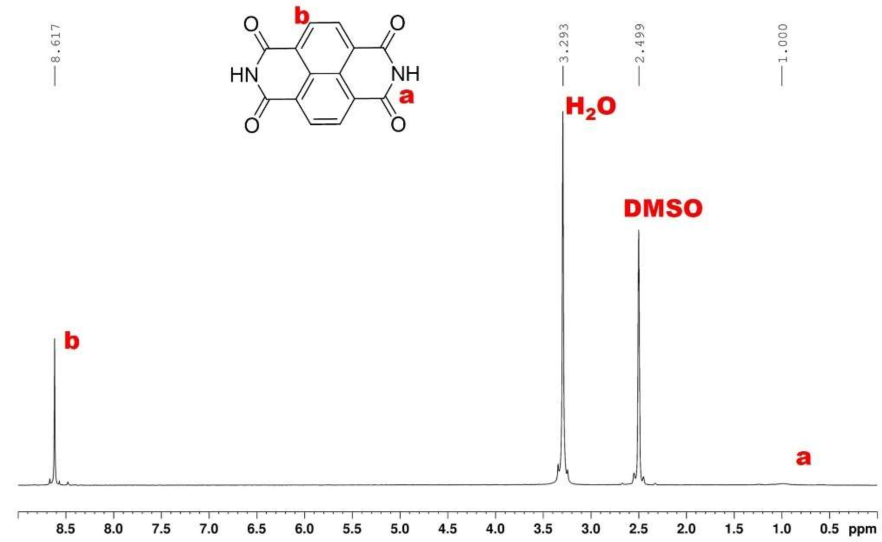

Figure 1. ^{1}H solution NMR spectrum of NDIH.

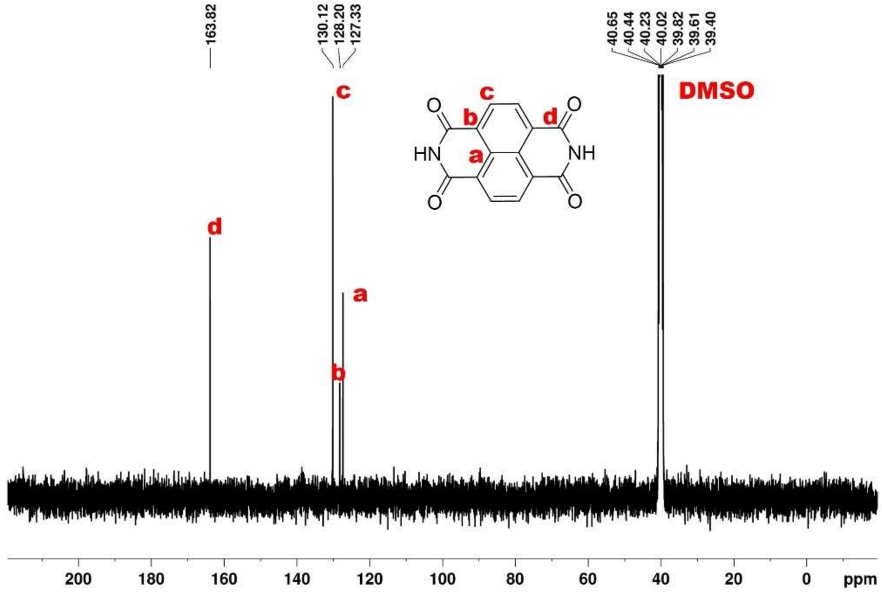

Figure 2. ^{13}C solution NMR spectrum of NDIH.

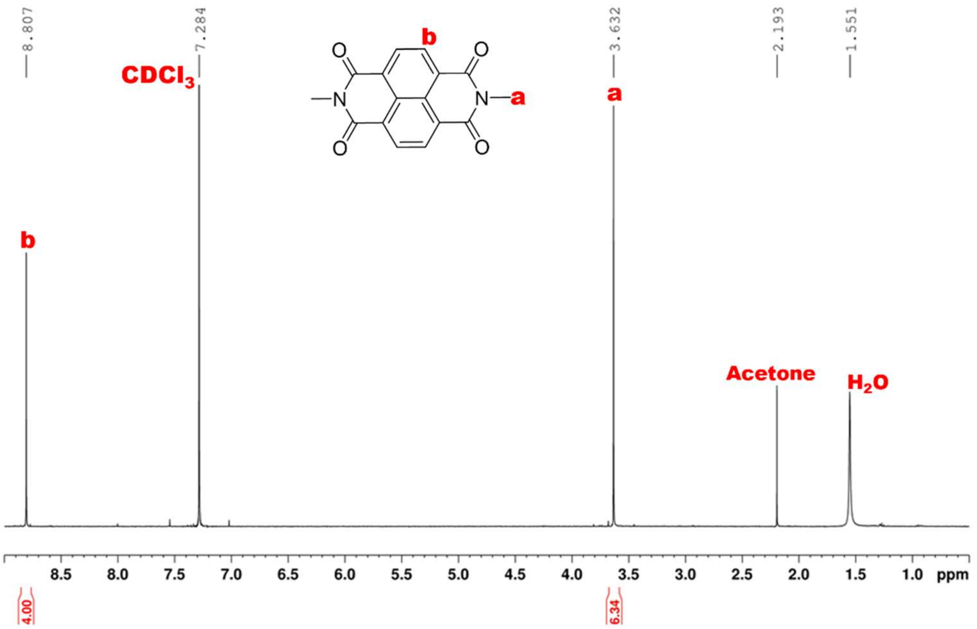

Figure 3. ^{1}H solution NMR spectrum of NDI1.

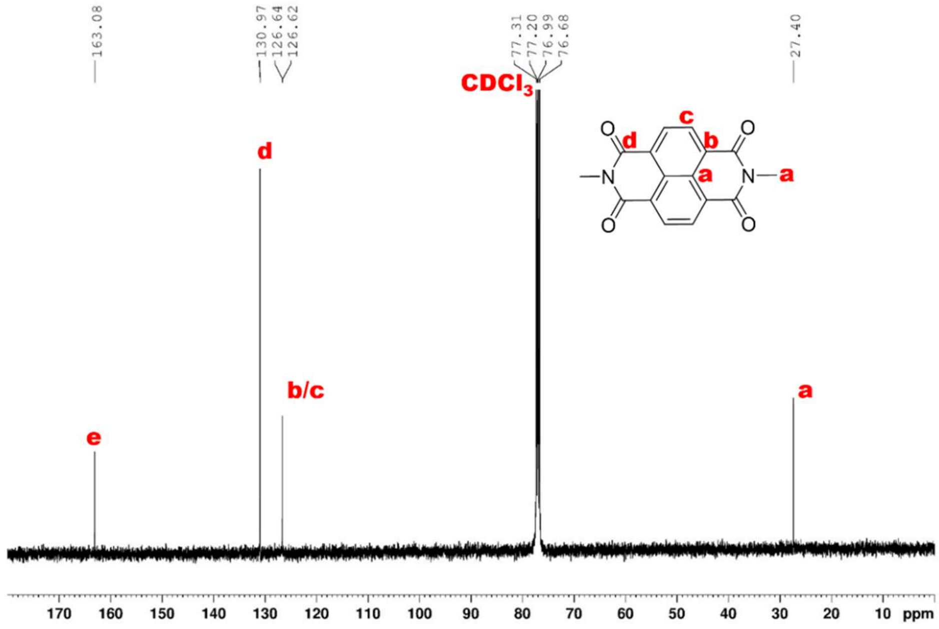

Figure 4. ^{13}C solution NMR spectrum of NDI1.

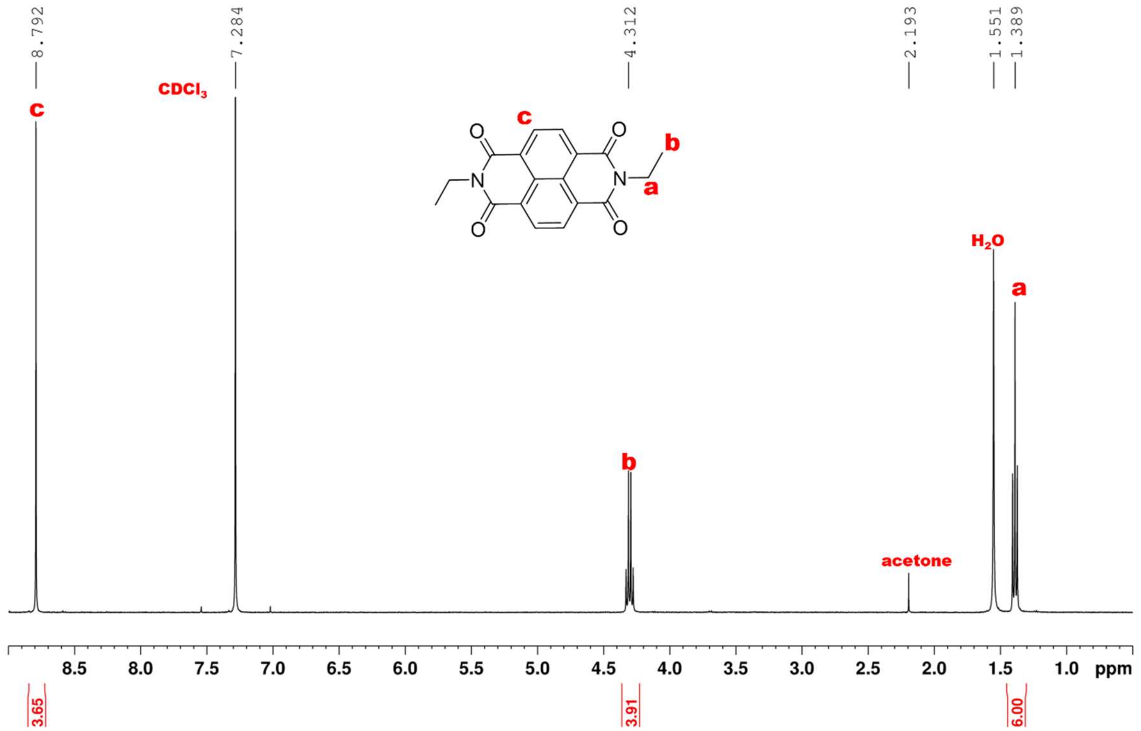

Figure 5. ^{1}H solution NMR spectrum of NDI2.

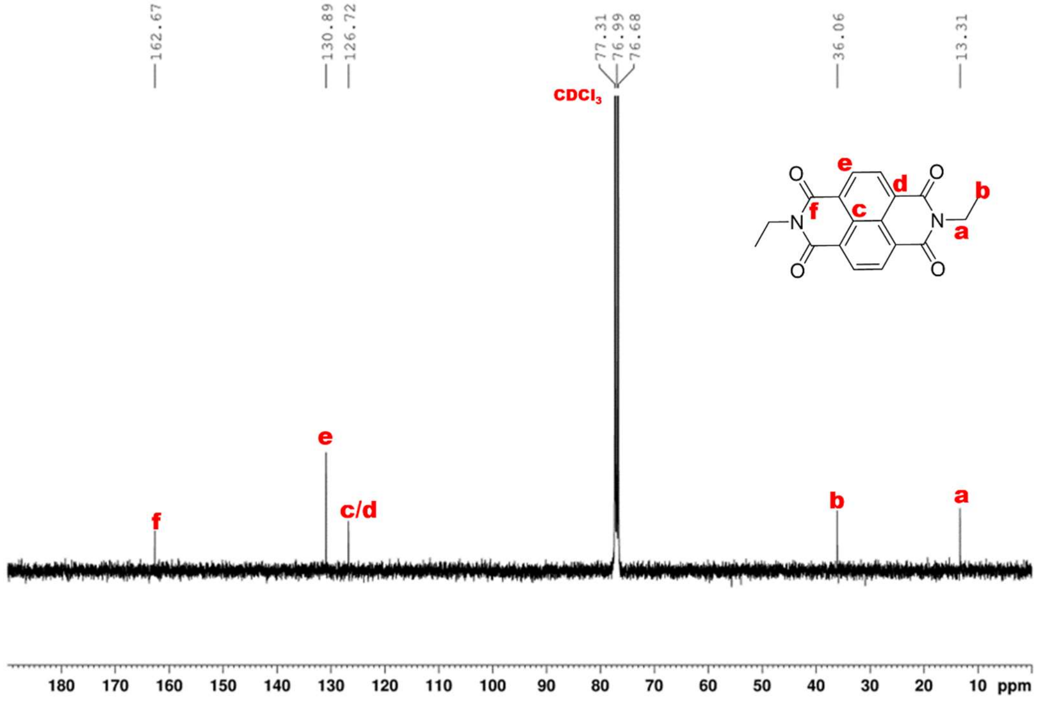

Figure 6. ^{13}C solution NMR spectrum of NDI2.

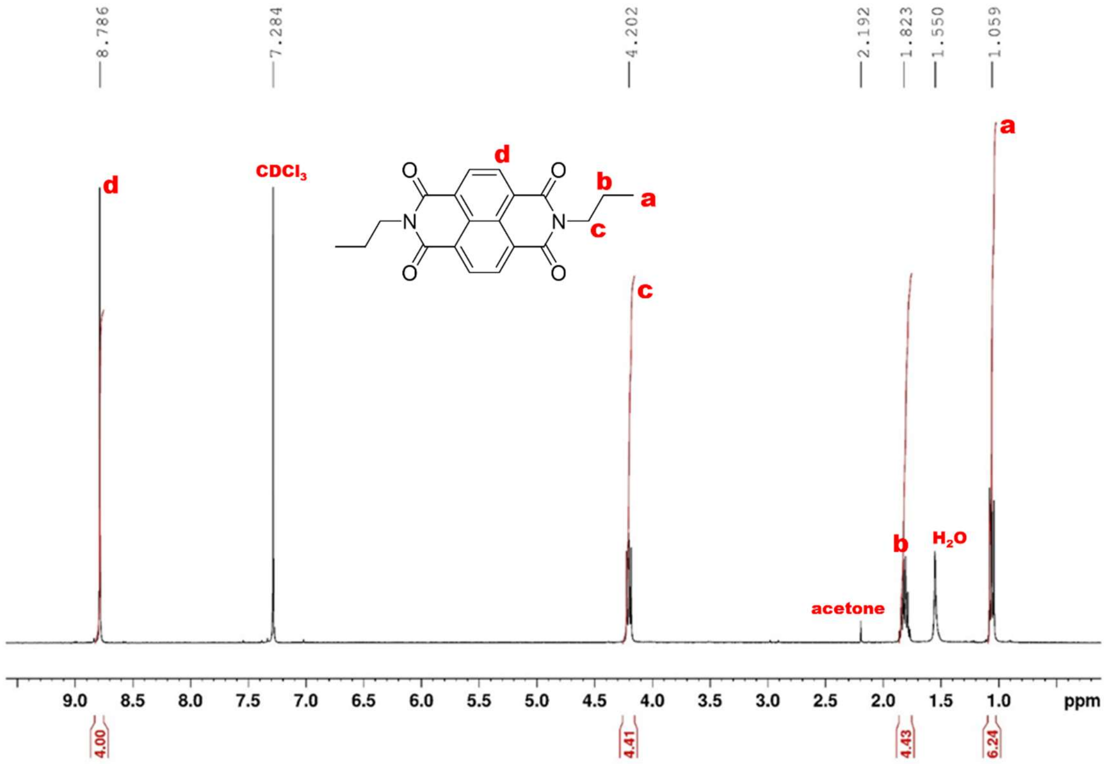

Figure 7. ^{1}H solution NMR spectrum of NDI3.

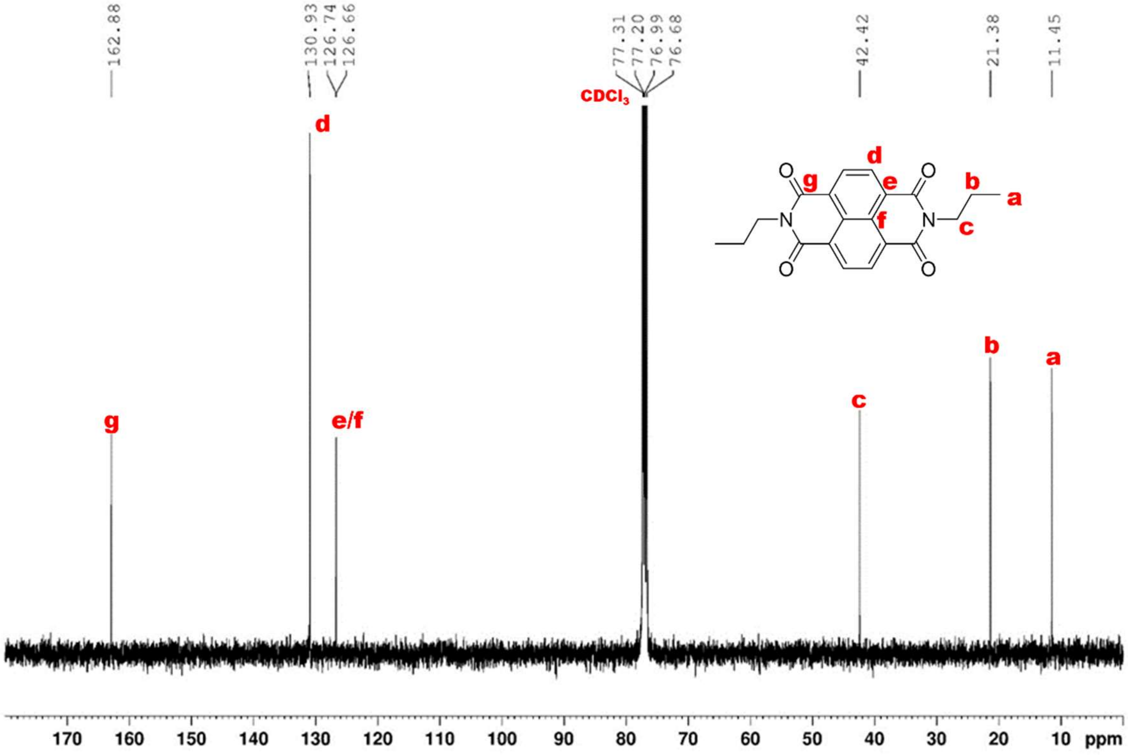

Figure 8. ^{13}C solution NMR spectrum of NDI3.

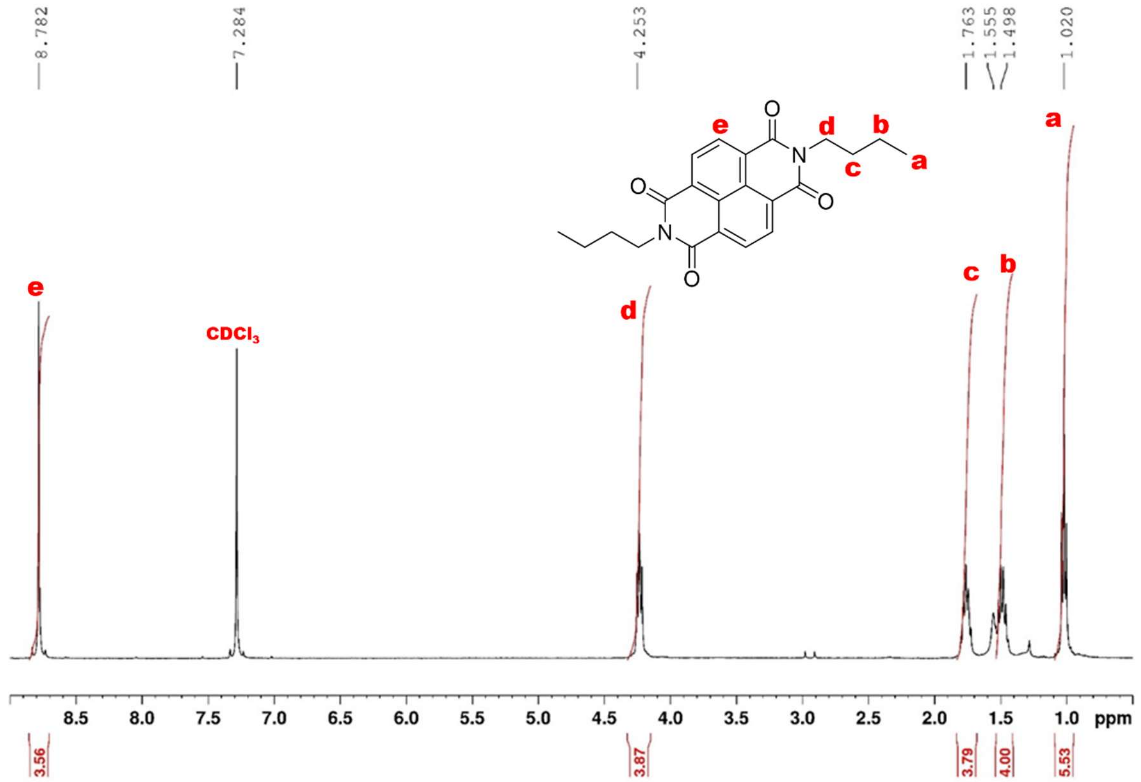

Figure 9. ^{1}H solution NMR spectrum of NDI4.

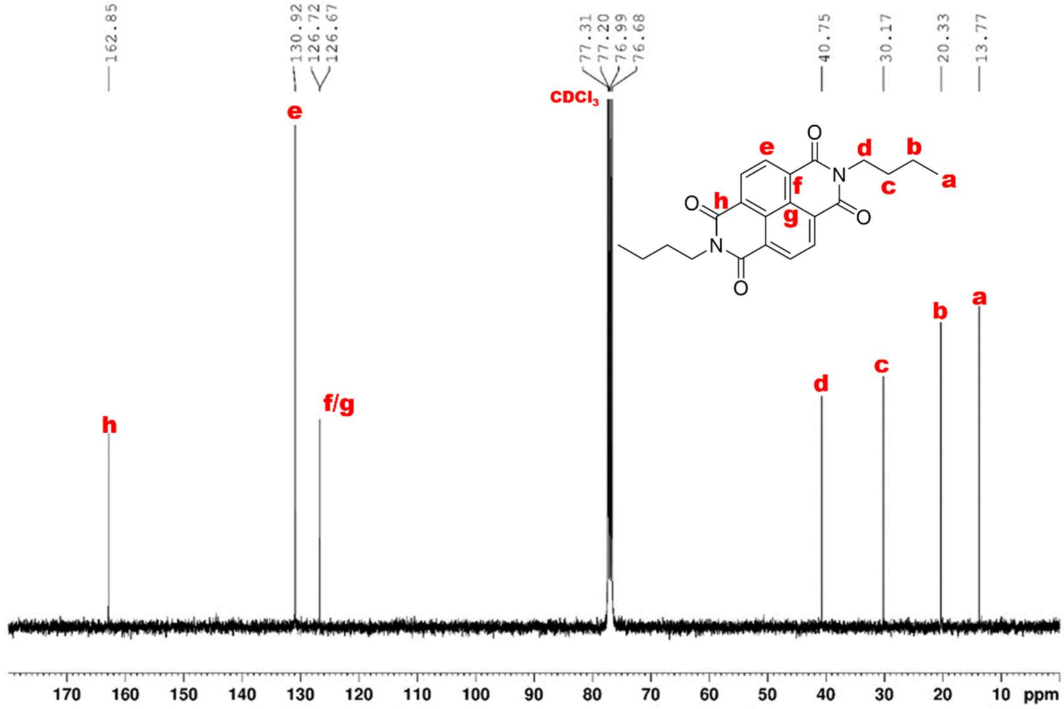

Figure 10. ^{13}C solution NMR spectrum of NDI4.

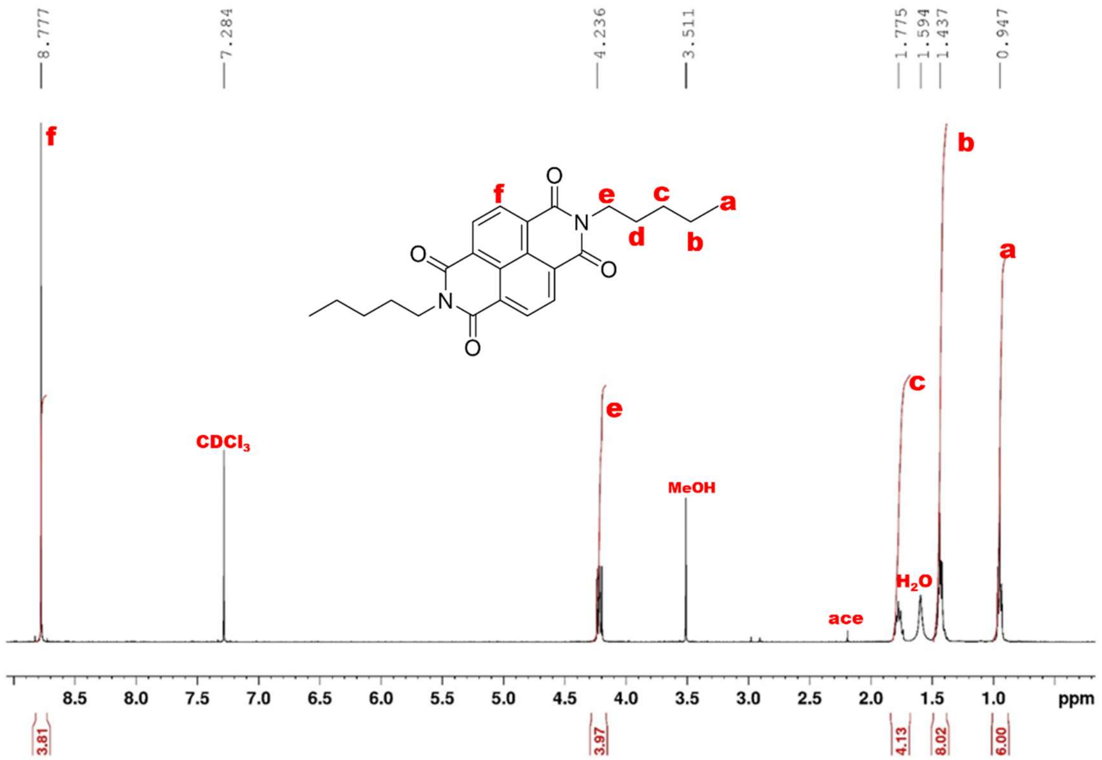

Figure 11. ^{1}H solution NMR spectrum of NDI5.

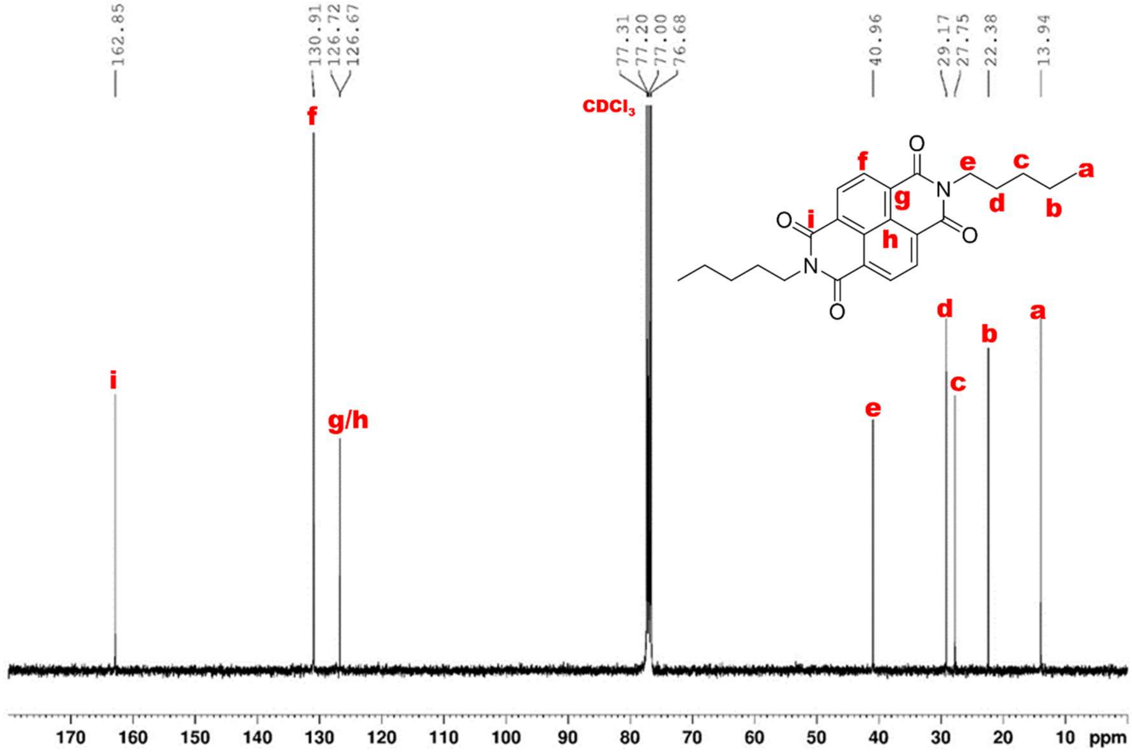

Figure 12. ^{13}C solution NMR spectrum of NDI5.

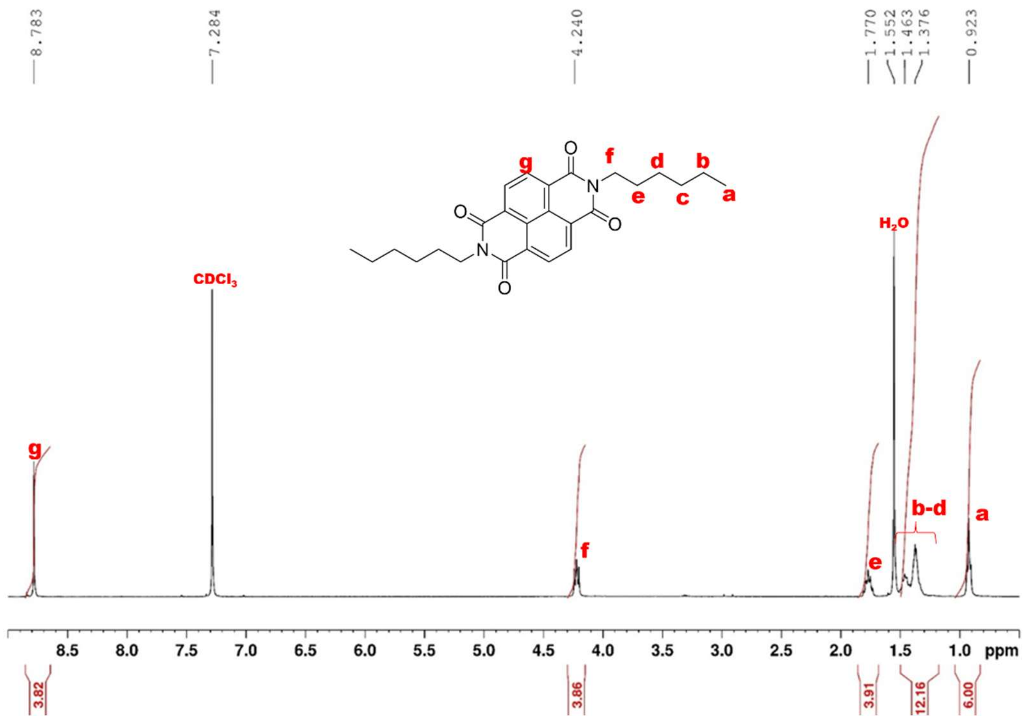

Figure 13. ^{1}H solution NMR spectrum of NDI6.

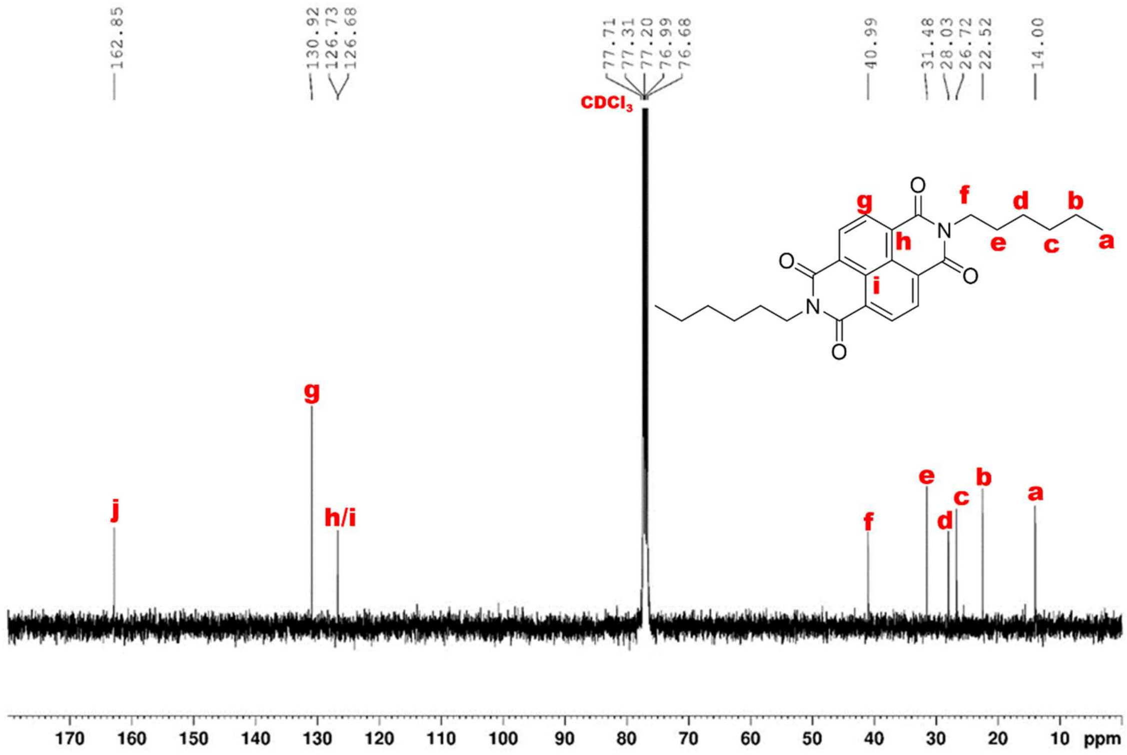

Figure 14. ^{13}C solution NMR spectrum of NDI6.

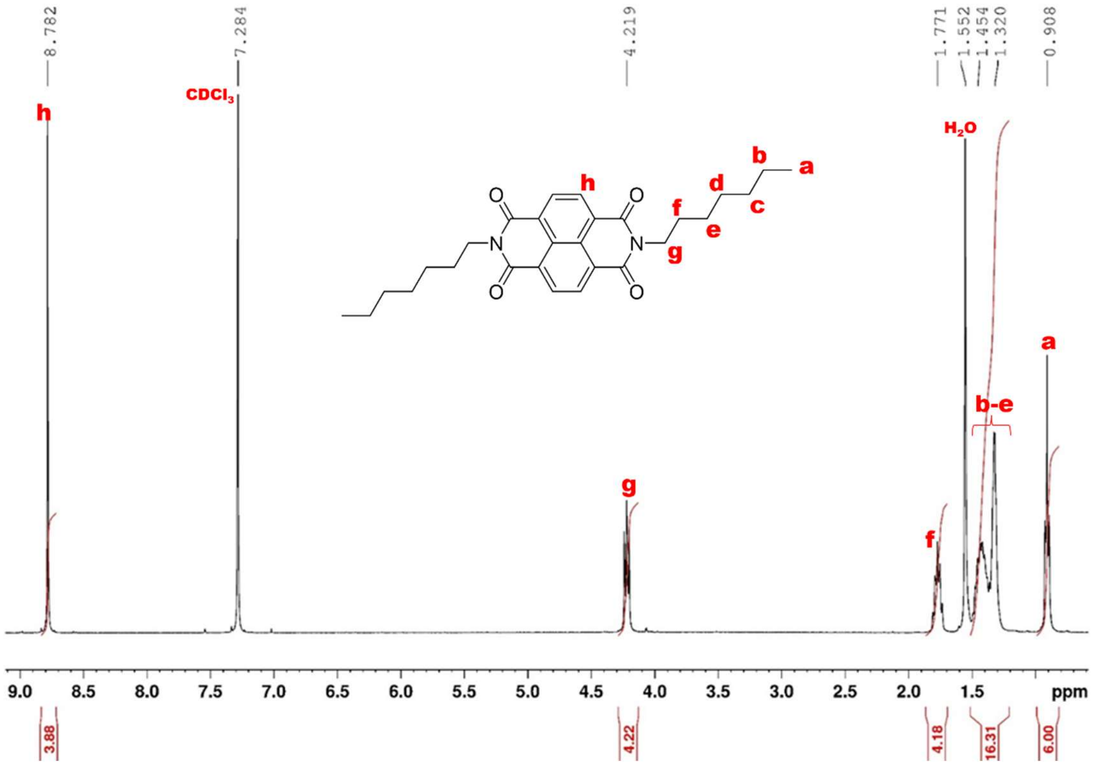

Figure 15. ^{1}H solution NMR spectrum of NDI7.

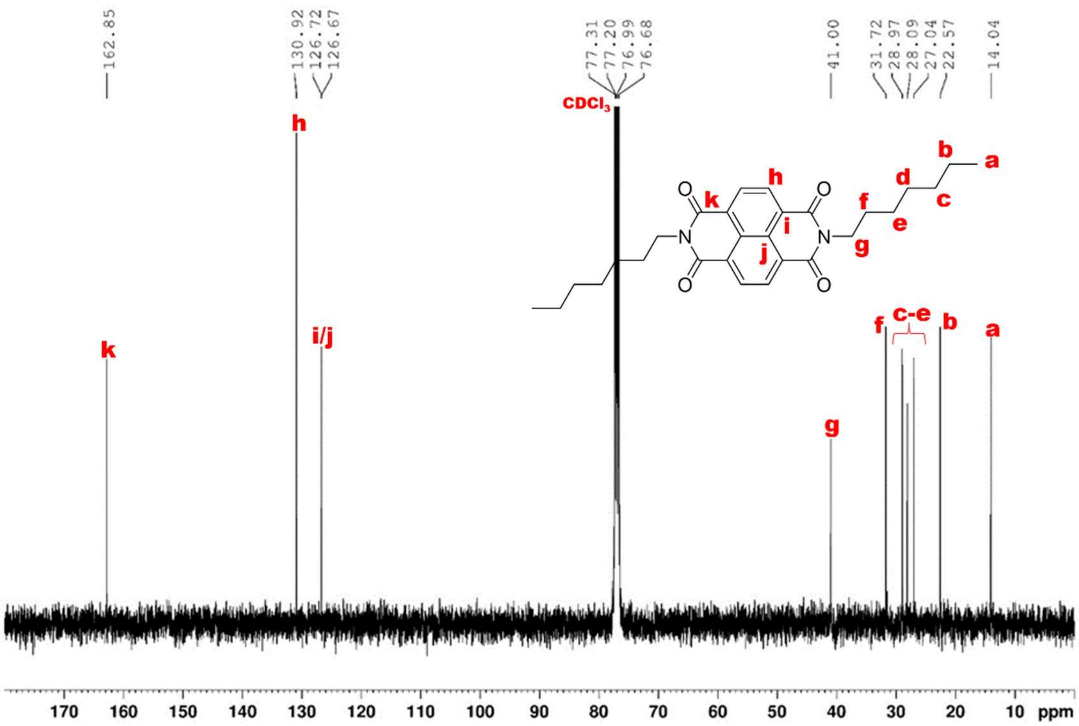

Figure 16. ^{13}C solution NMR spectrum of NDI7.

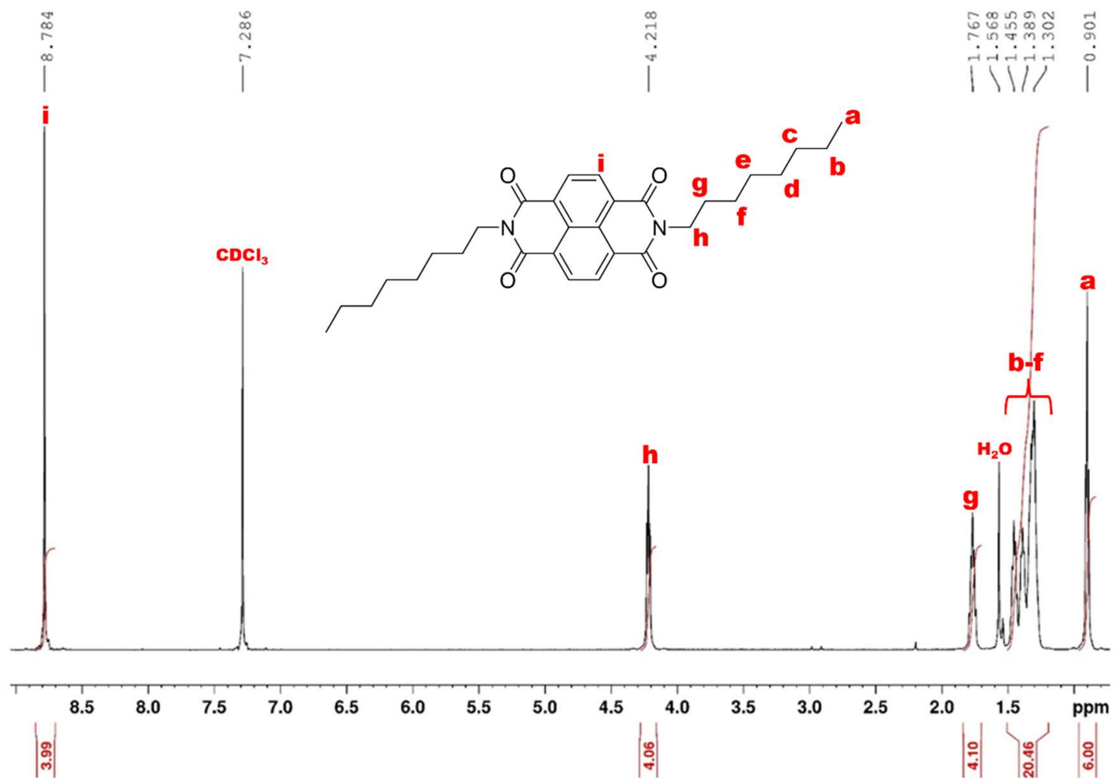

Figure 17. ^{1}H solution NMR spectrum of NDI8.

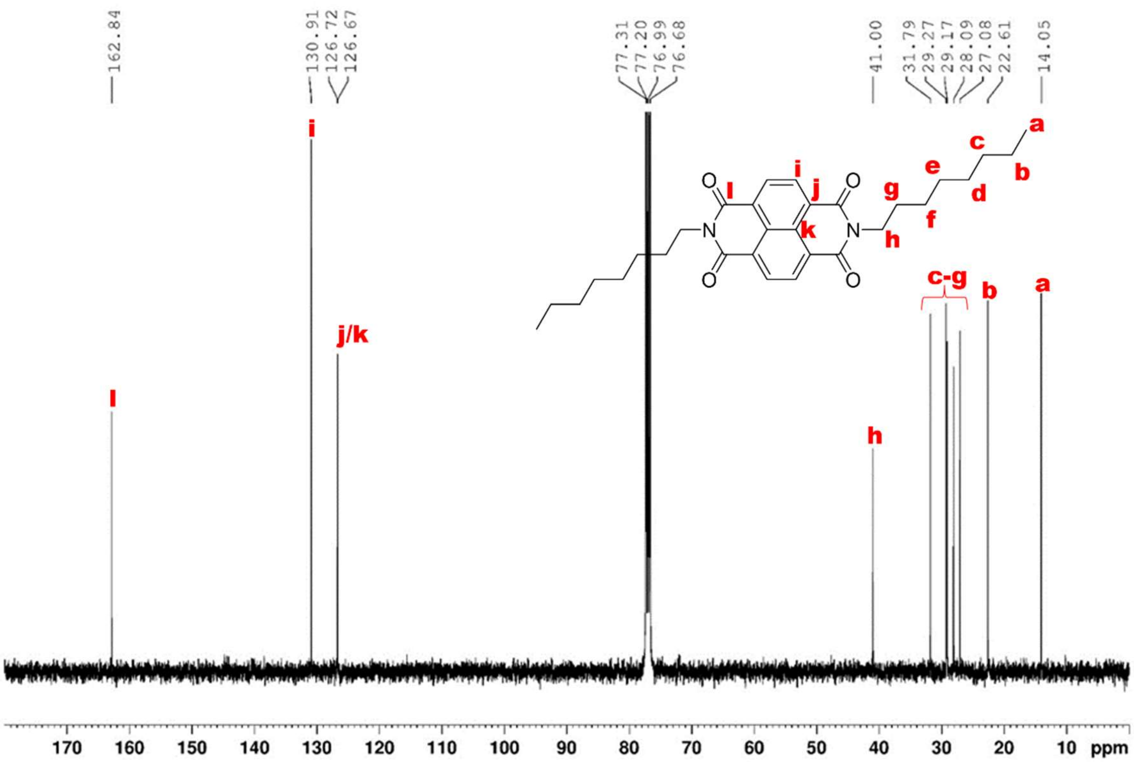

Figure 18. ^{13}C solution NMR spectrum of NDI8.

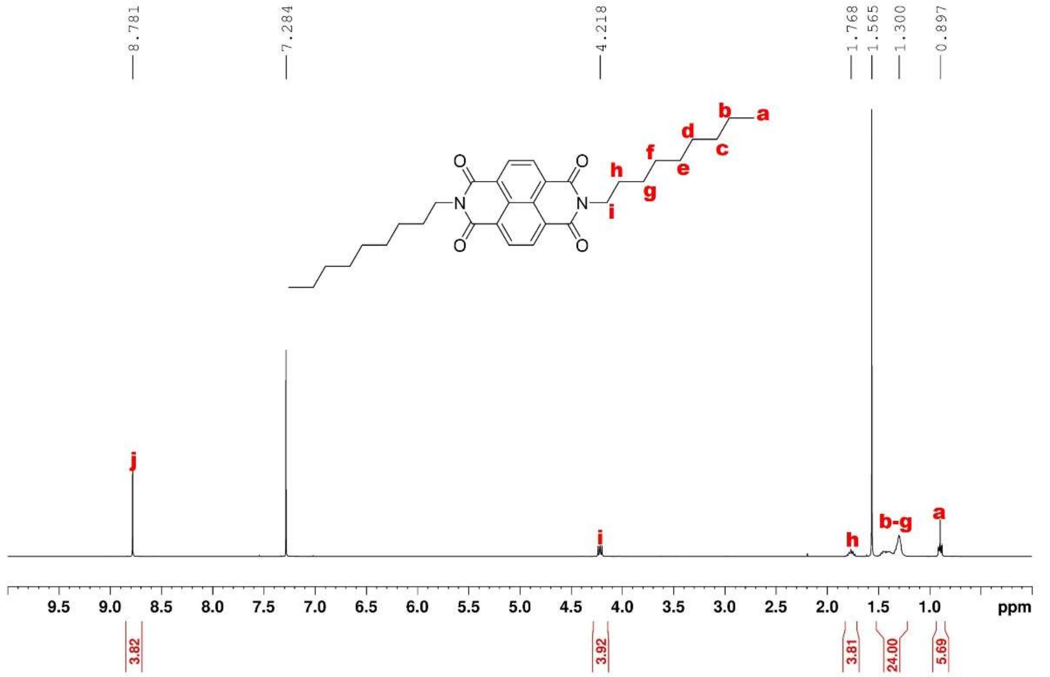

Figure 19. ^{1}H solution NMR spectrum of NDI9.

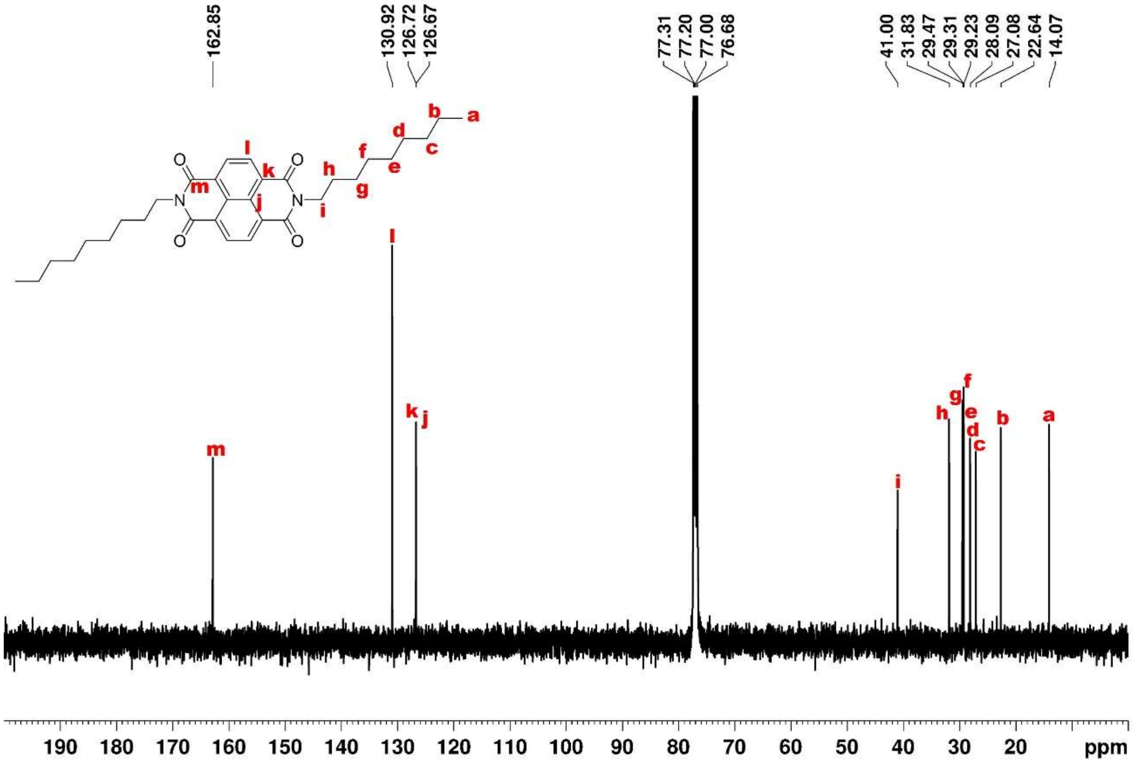

Figure 20. ^{1}H solution NMR spectrum of NDI9.

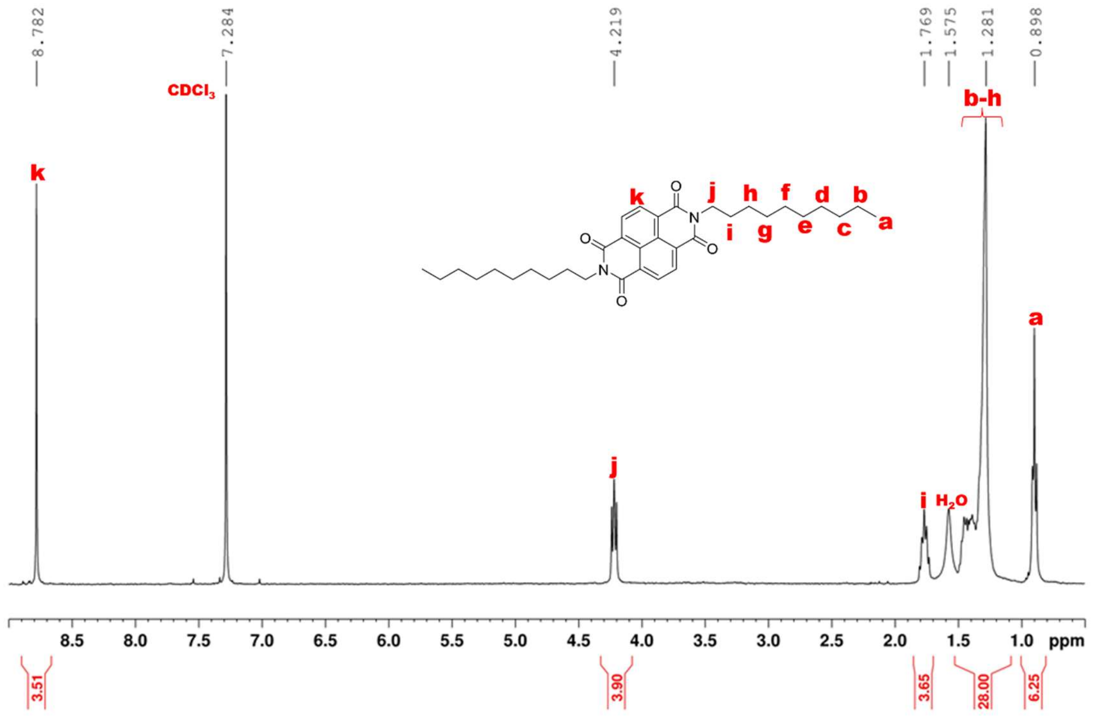

Figure 21. ^{1}H solution NMR spectrum of NDI10.

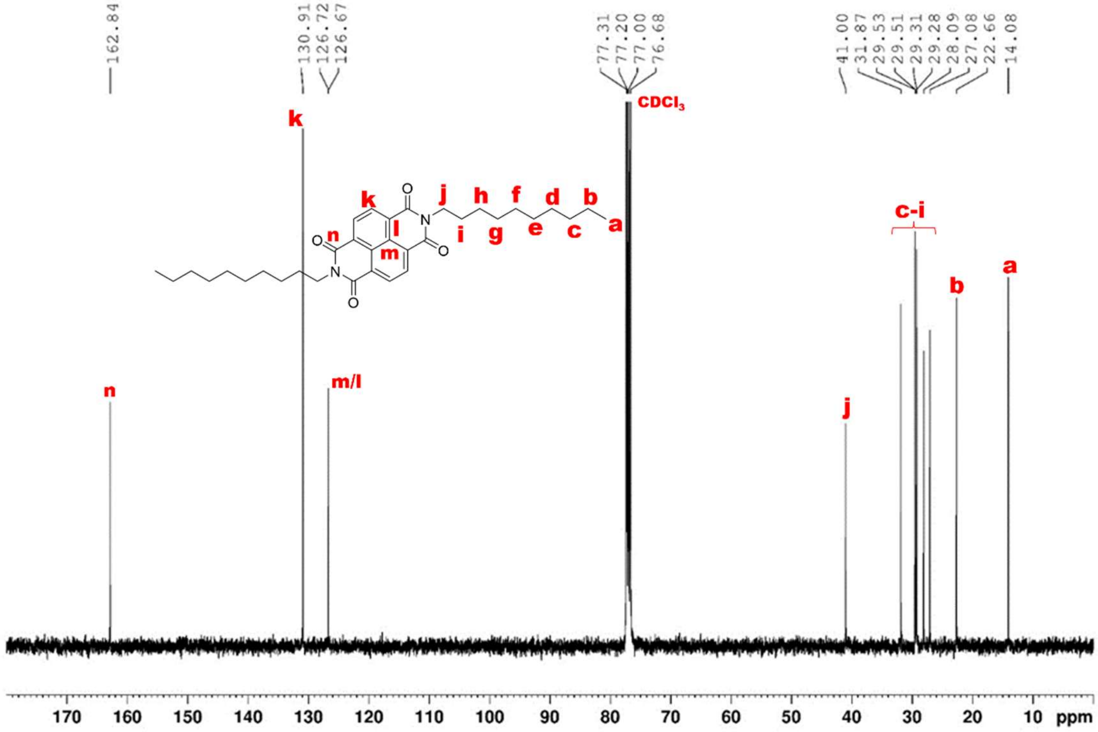

Figure 22. ^{13}C solution NMR spectrum of NDI10.

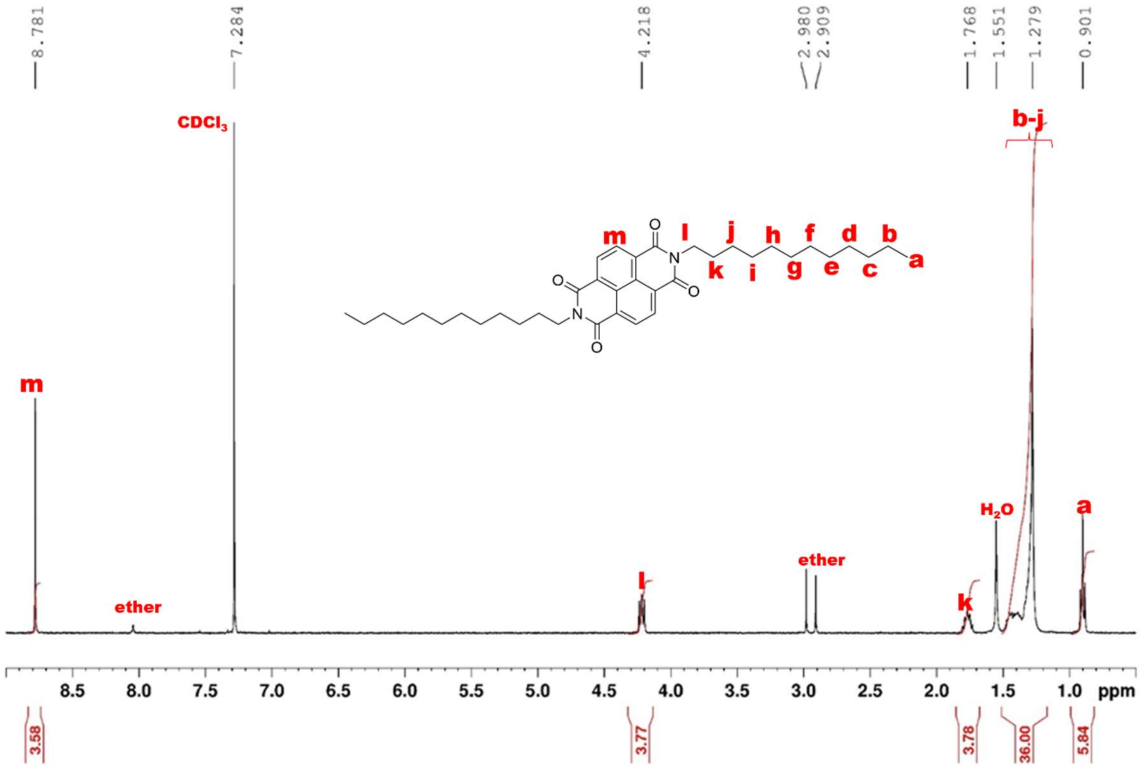

Figure 23. ^{1}H solution NMR spectrum of NDI12.

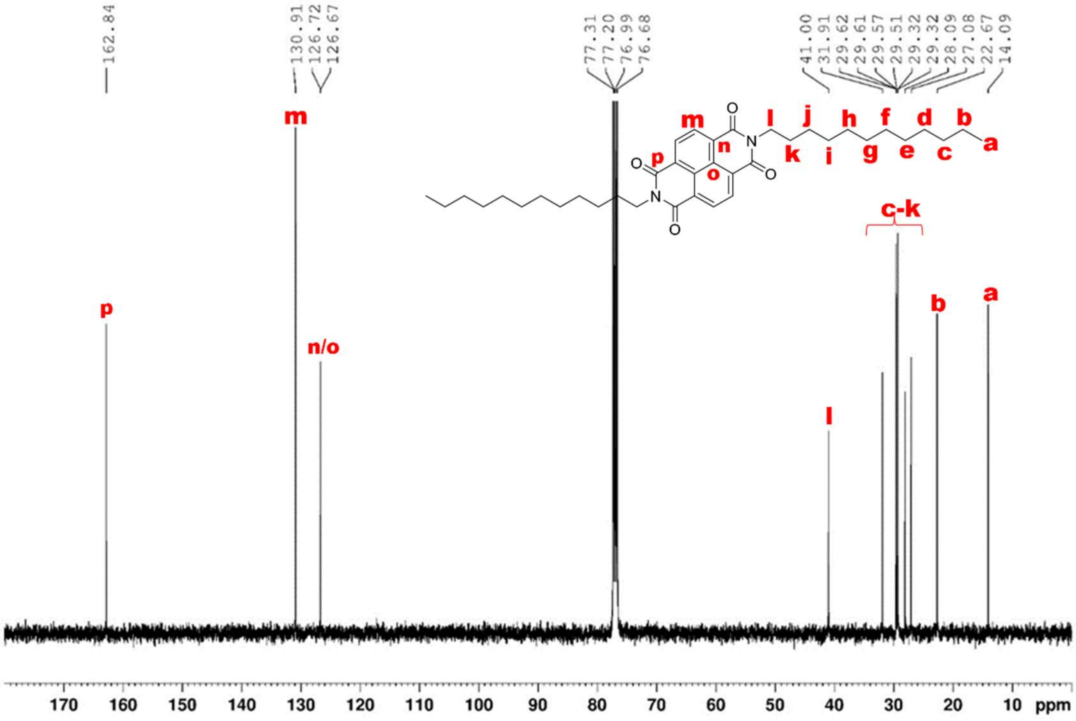

Figure 24. ^{13}C solution NMR spectrum of NDI12.

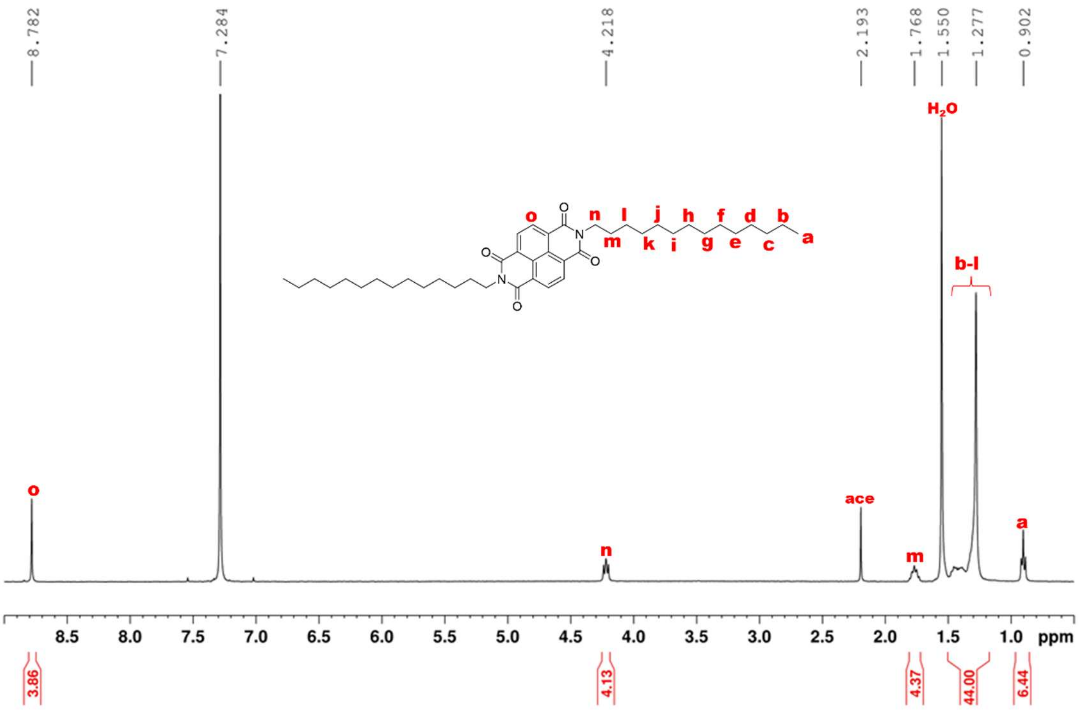

Figure 25. ^{1}H solution NMR spectrum of NDI14.

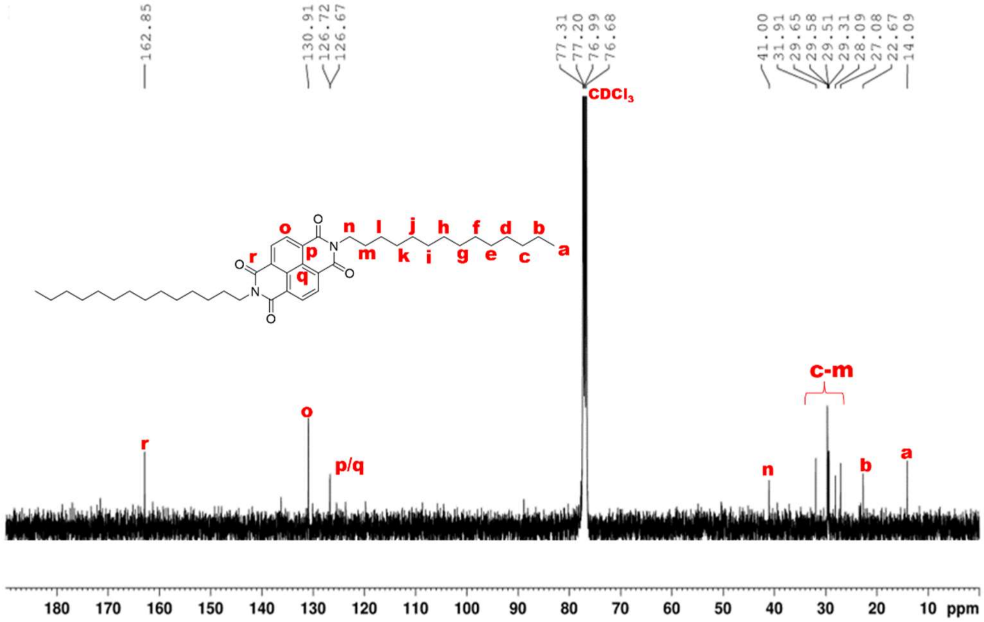

Figure 26. ^{13}C solution NMR spectrum of NDI14.

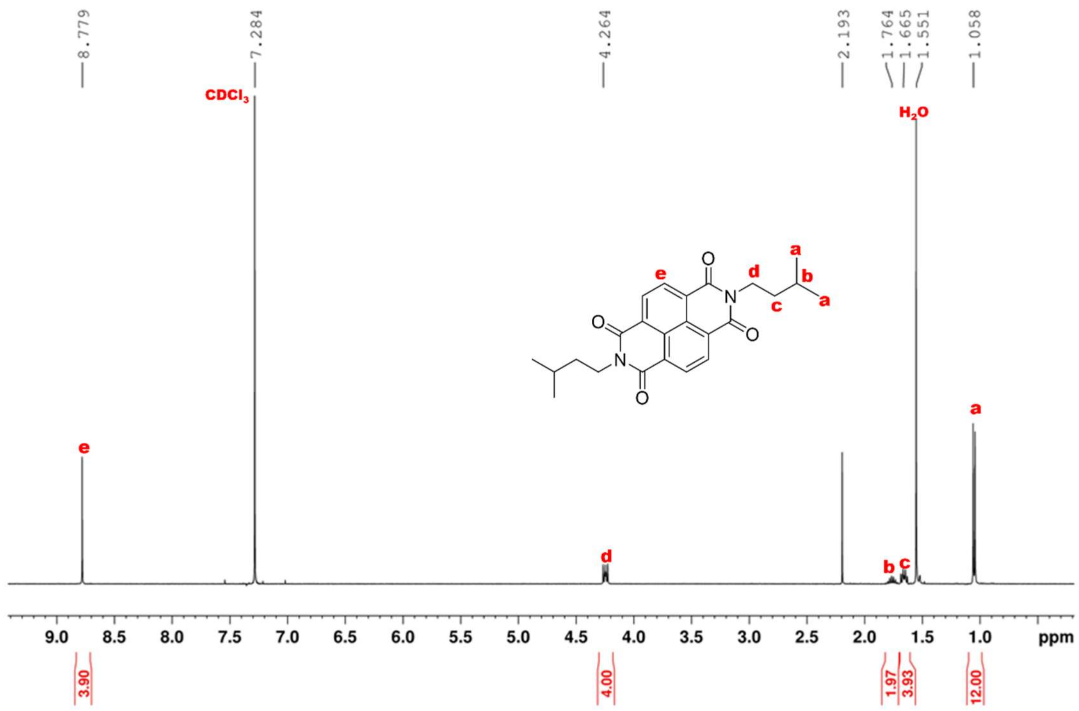

Figure 27. ^{1}H solution NMR spectrum of NDI5a.

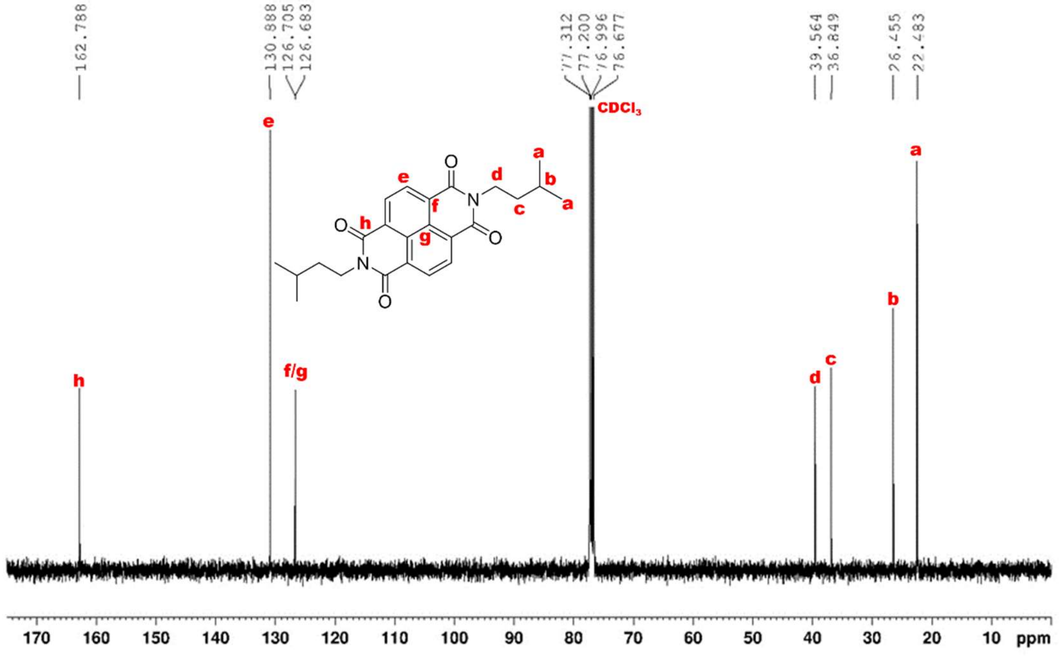

Figure 28. ^{13}C solution NMR spectrum of NDI5a.

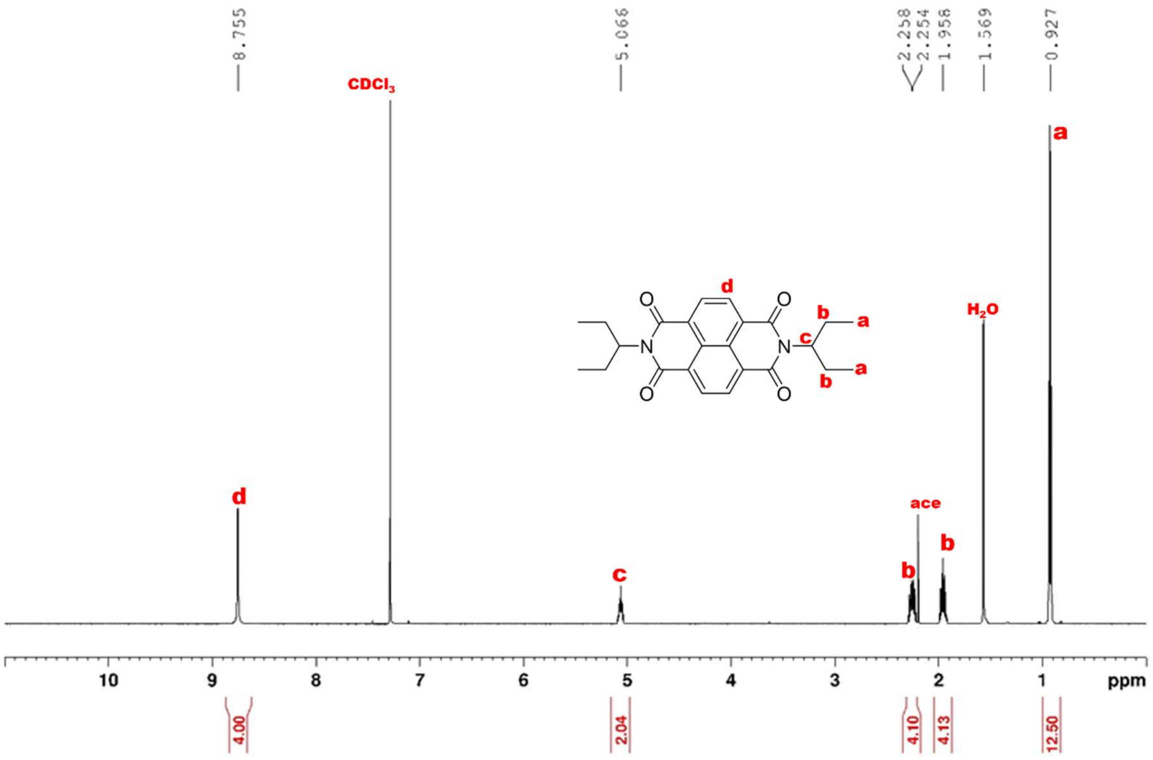

Figure 29. ^{1}H solution NMR spectrum of NDI5b.

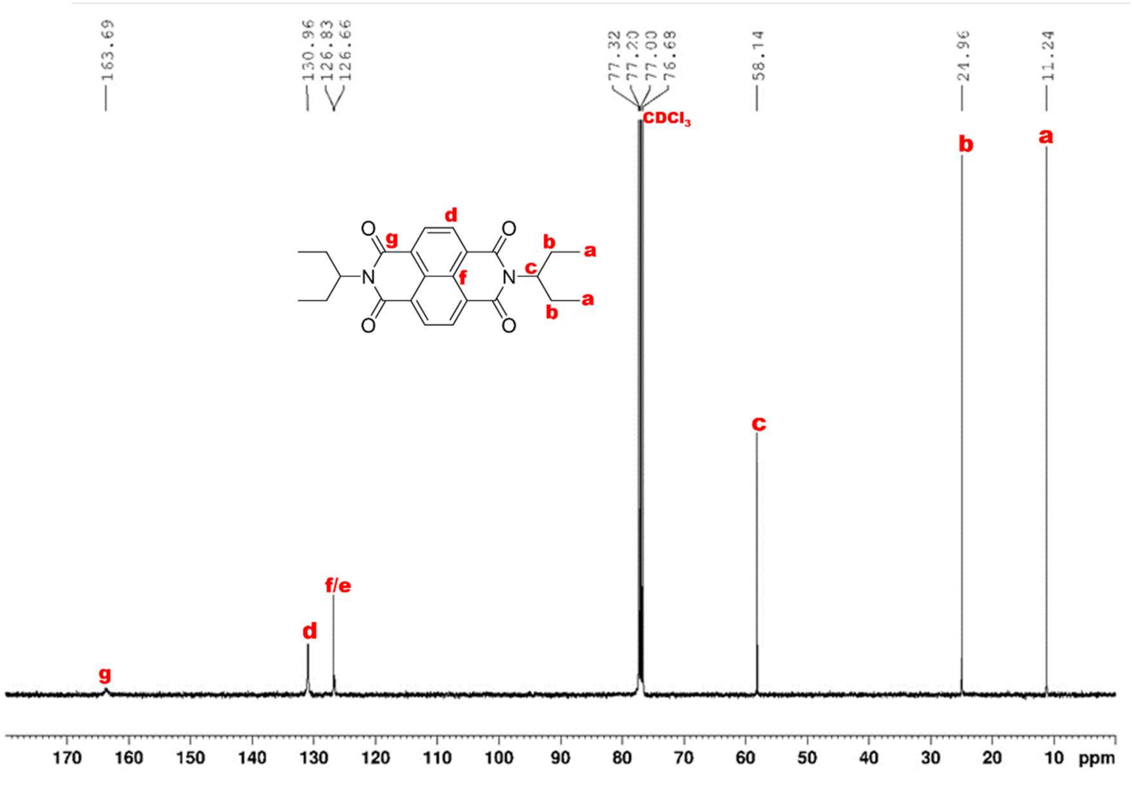

Figure 30. ^{13}C solution NMR spectrum of NDI5b.

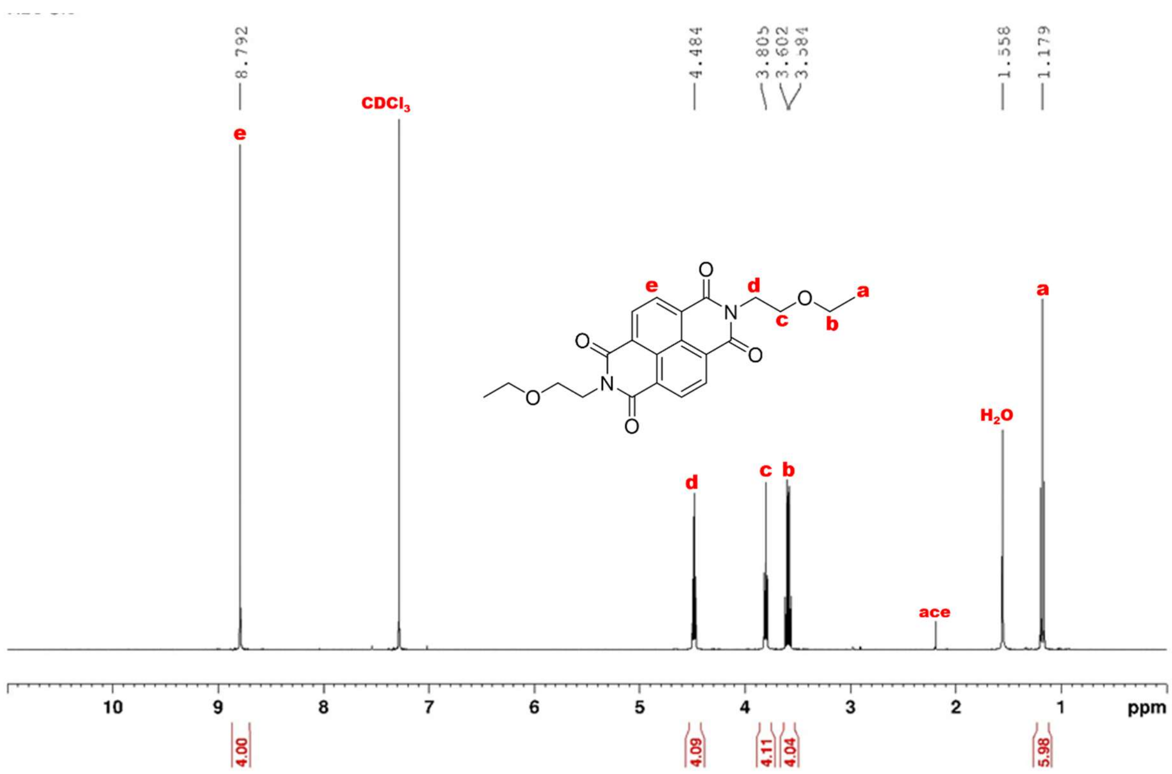

Figure 31. ^{1}H solution NMR spectrum of NDIOa.

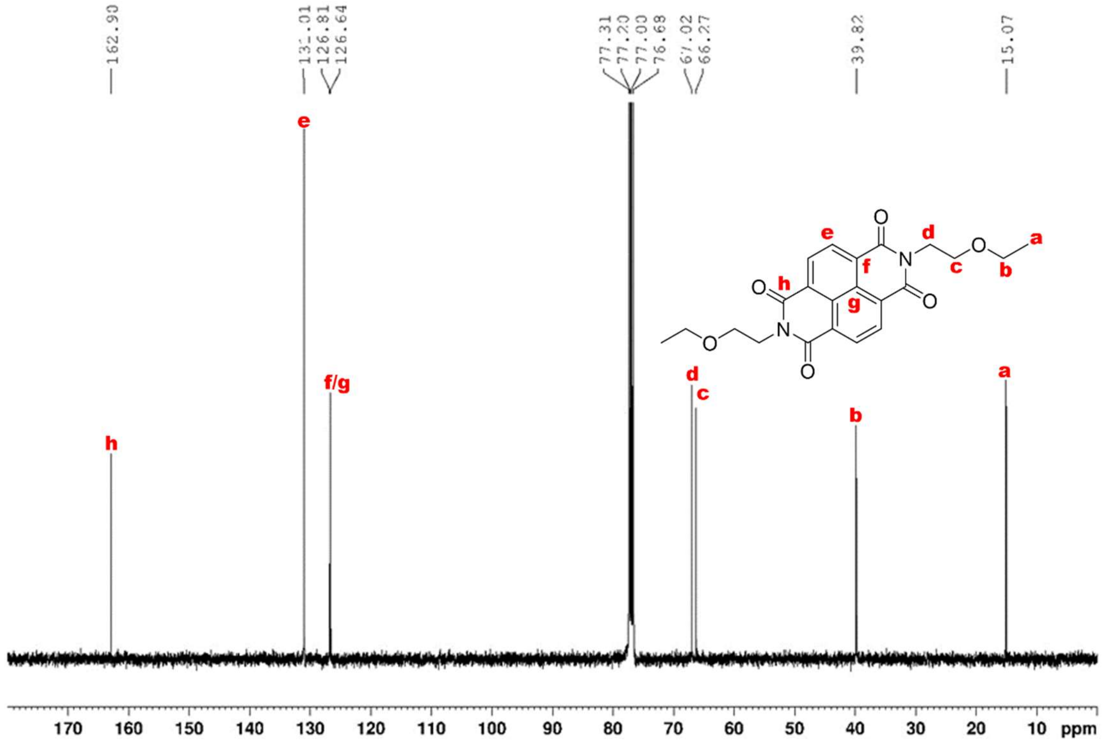

Figure 32. ^{13}C solution NMR spectrum of NDIOa.

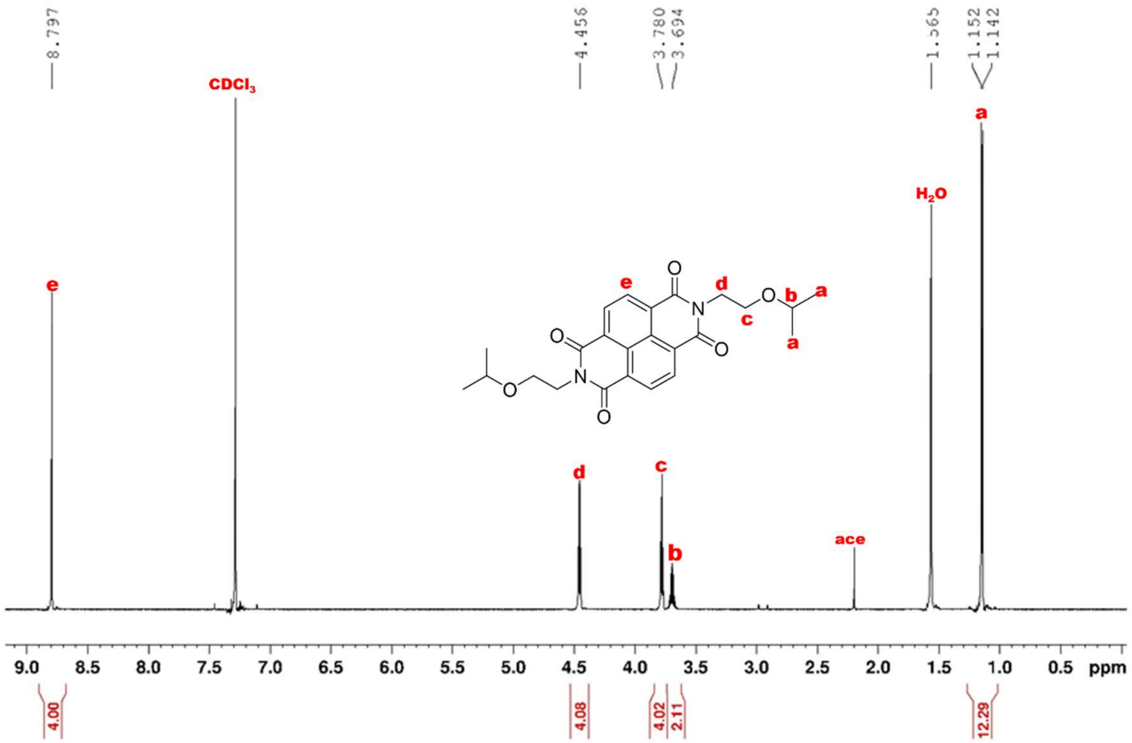

Figure 33. ^{1}H solution NMR spectrum of NDIOb.

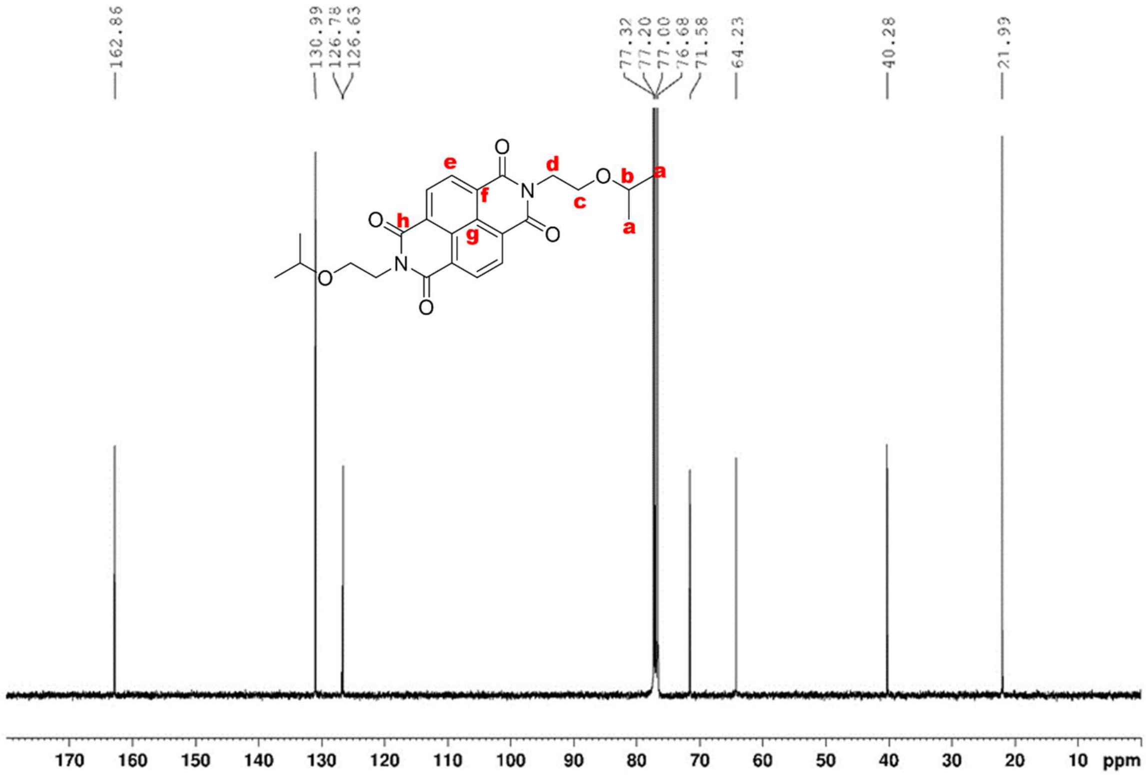

Figure 34. ^{13}C solution NMR spectrum of NDIOb.

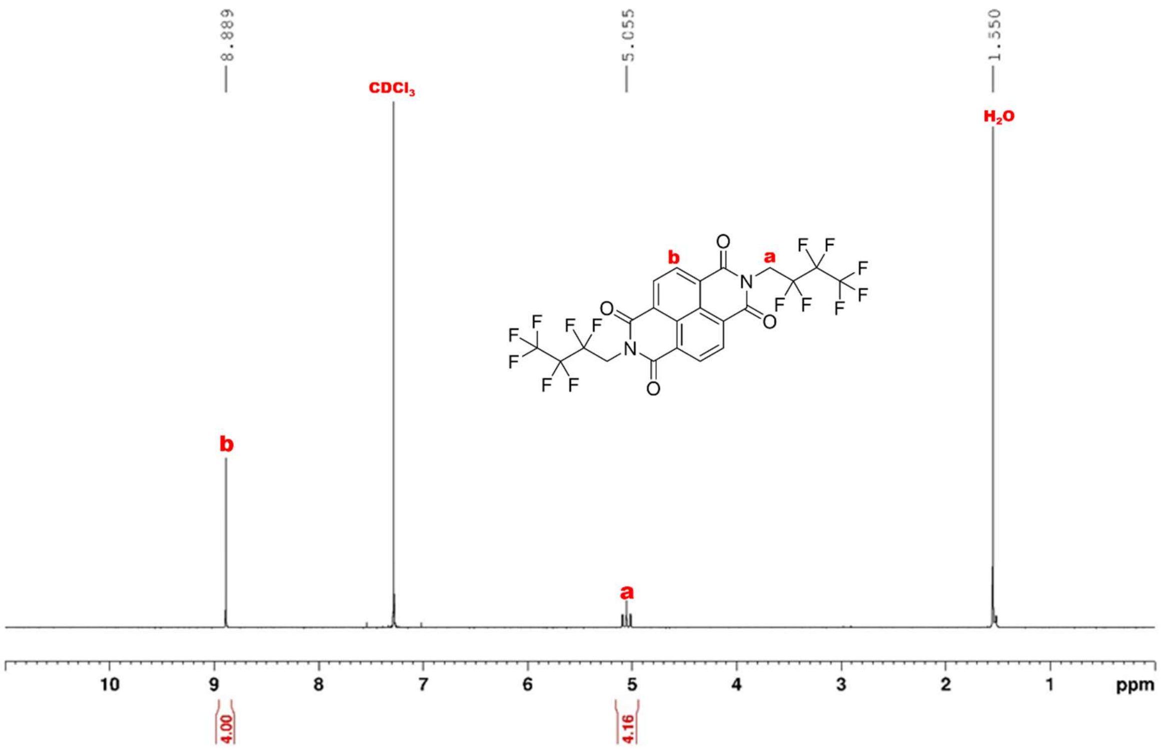

Figure 35. ^{1}H solution NMR spectrum of NDIF.

Solid-state NMR

All NMR experiments were performed on a Bruker Avance II 400 spectrometer at a ^{13}C frequency of 100 MHz with 4 mm zirconia rotors in a double-resonance probe head. The ^{13}C chemical shifts were referenced to tetramethylsilane, using the COO resonance of glycine in the α-modification at 176.46 ppm as a secondary reference. ^{1}H chemical shifts were referenced using adamantane at 1.85 ppm as a secondary reference. Full width half max (FWHM) values were determined by fitting spectra with a series of Gaussian/Lorentzian functions to allow for the deconvolution of overlapping resonances.

^{13}C multiple cross-polarization/magic angle spinning (multiCP/MAS) and ^{13}C multiCP/MAS with dipolar dephasing.

The 1D ^{13}C multiCP/MAS NMR excitation method described by Johnson and Schmidt-Rohr was used to obtain high-resolution solid-state ^{13}C NMR spectra of NDIs.[4] Spectra were recorded at a spinning frequency of 14 kHz, with a 90° ^{13}C pulse duration of 4.1 μs, 1 s delay, and 5 cycles. Corresponding multiCP spectra (red) of nonprotonated carbon and mobile groups were obtained after a dipolar dephasing (multiCP/DD) time of 68 μs. The multiCP/DD spectra were scaled down from the multiCP spectra for clarity but have the same intensity. The DD technique here was used to distinguish protonated carbons (pCs) from nonprotonated carbons (npCs).[5,6]

^{1}H-^{13}C Frequency Switched Lee Goldburg Heteronuclear Correlation (FSLG HetCor).

^{1}H-^{13}C Two-dimensional (2D) HetCor NMR experiments were used to provide ^{1}H-^{13}C proximities.[7] Experiments were performed at a spinning speed of 14 kHz. Lee-Goldberg cross polarization (LG-CP) with a contact time of 75 μs suppressed ^{1}H-^{1}H spin diffusion during polarization transfer and showed primarily one-bond ^{1}H-^{13}C connectivities.

Solid-state 1D ^{13}C multiCP/MAS and ^{13}C multiCP/MAS with dipolar dephasing spectra

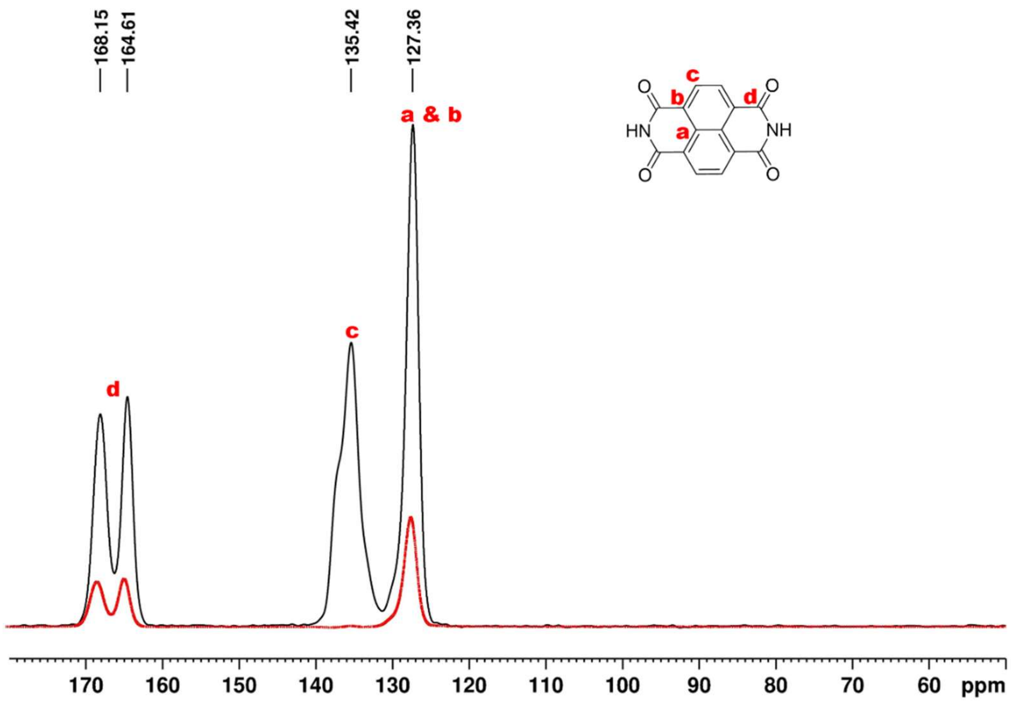

Figure 36. ^{13}C multiCP/MAS and ^{13}C multiCP/MAS with dipolar dephasing ssNMR spectra of NDIH.

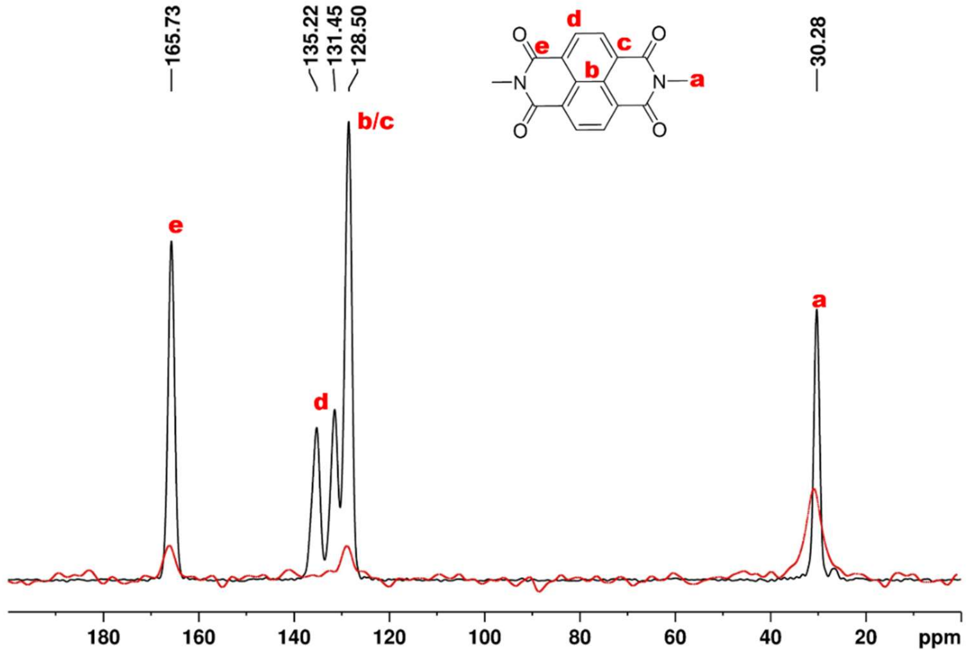

Figure 37. ^{13}C multiCP/MAS and ^{13}C multiCP/MAS with dipolar dephasing ssNMR spectra of NDI1.

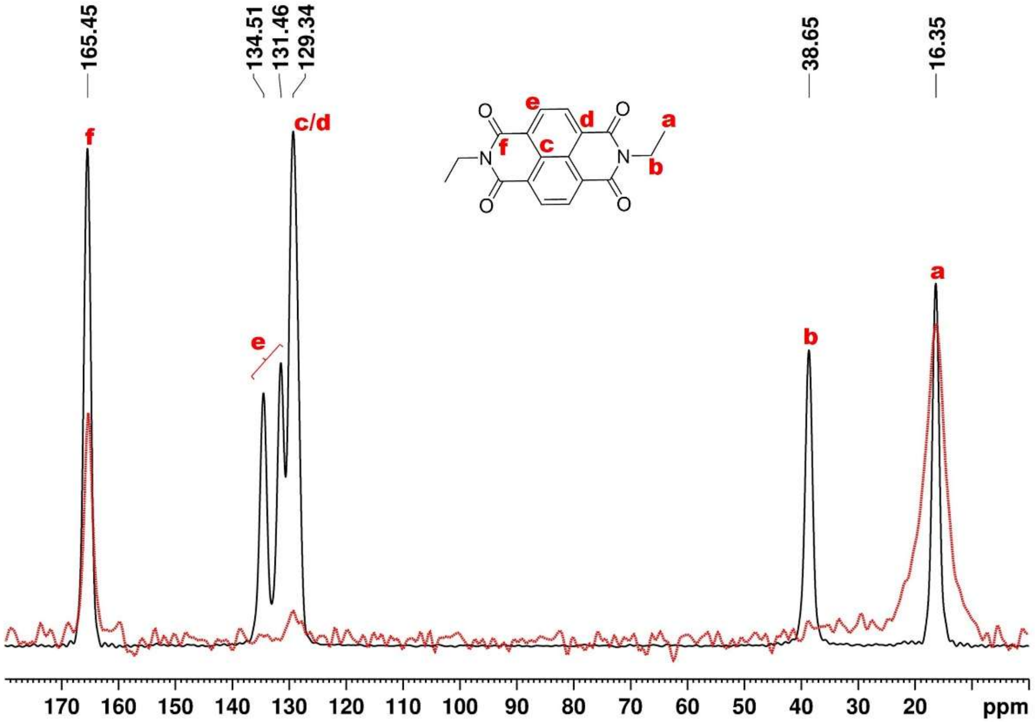

Figure 38. ^{13}C multiCP/MAS and ^{13}C multiCP/MAS with dipolar dephasing ssNMR spectra of NDI2.

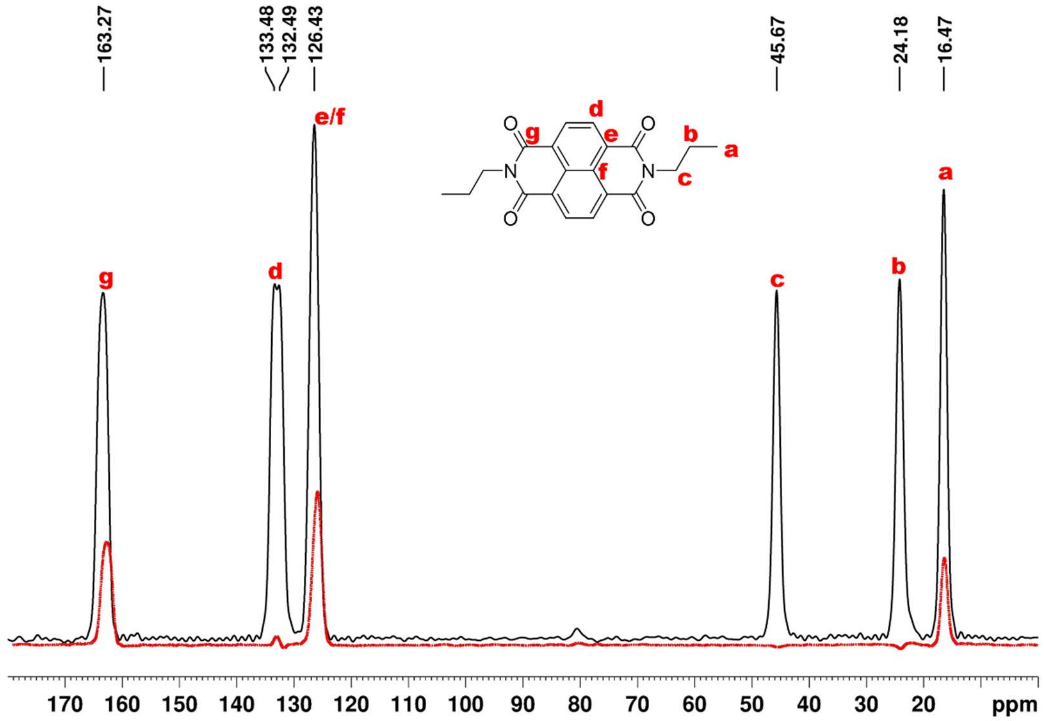

Figure 39. ^{13}C multiCP/MAS and ^{13}C multiCP/MAS with dipolar dephasing ssNMR spectra of NDI3.

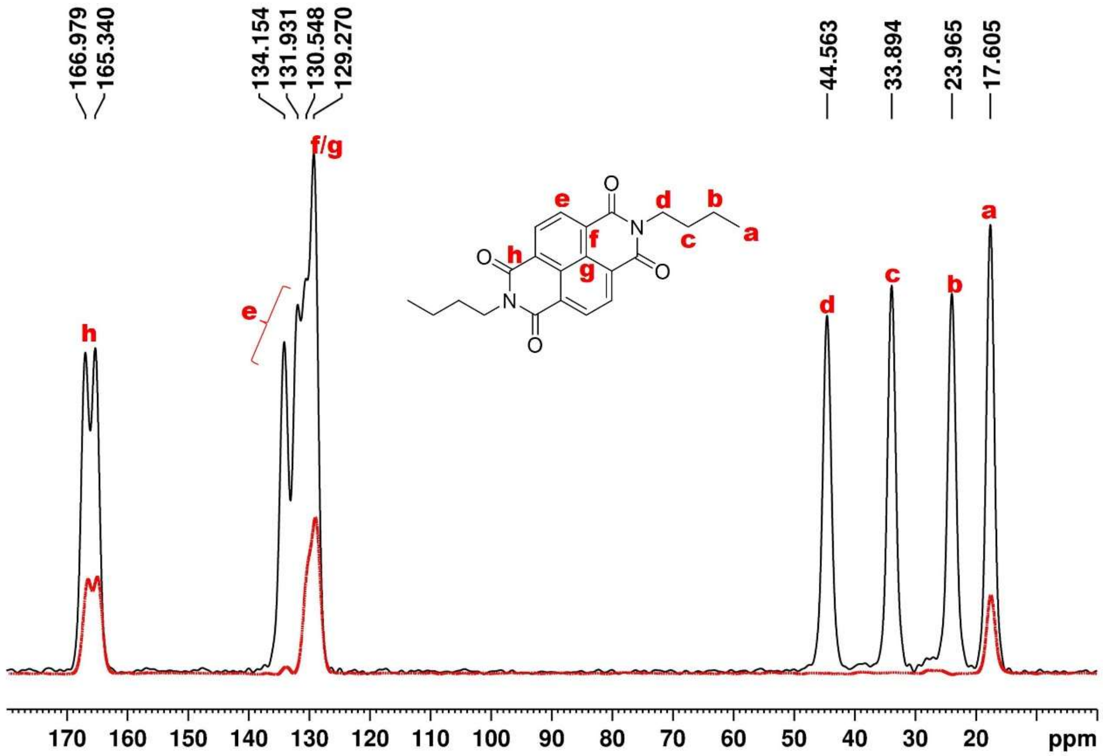

Figure 40. ^{13}C multiCP/MAS and ^{13}C multiCP/MAS with dipolar dephasing ssNMR spectra of NDI4.

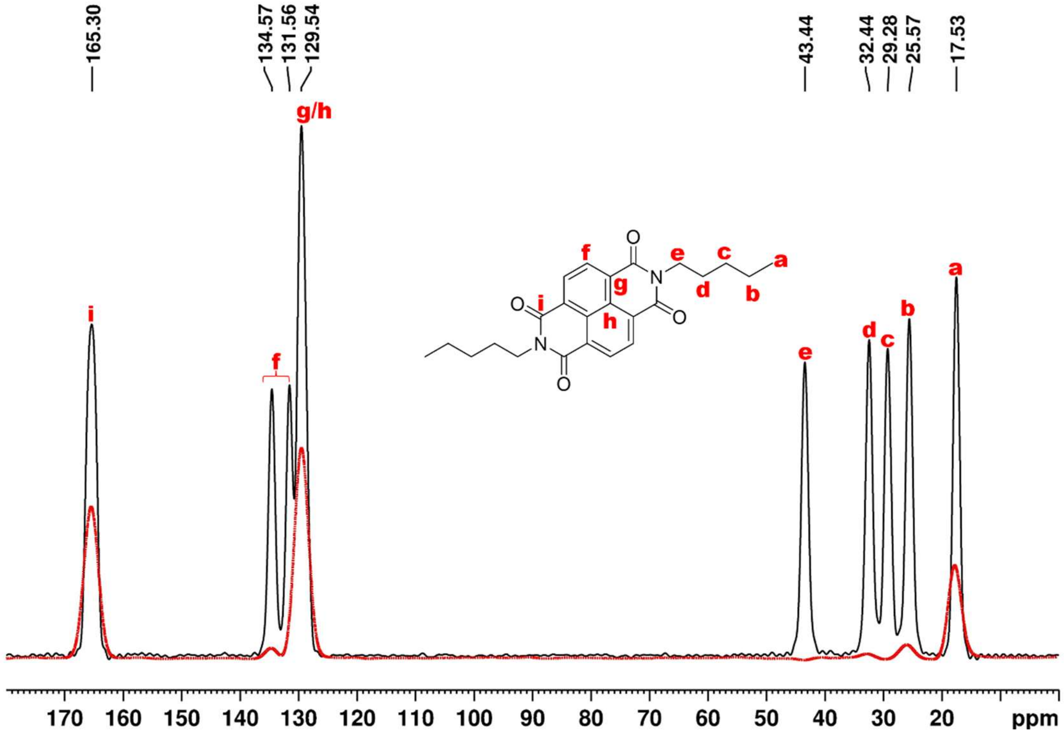

Figure 41. ^{13}C multiCP/MAS and ^{13}C multiCP/MAS with dipolar dephasing ssNMR spectra of NDI5.

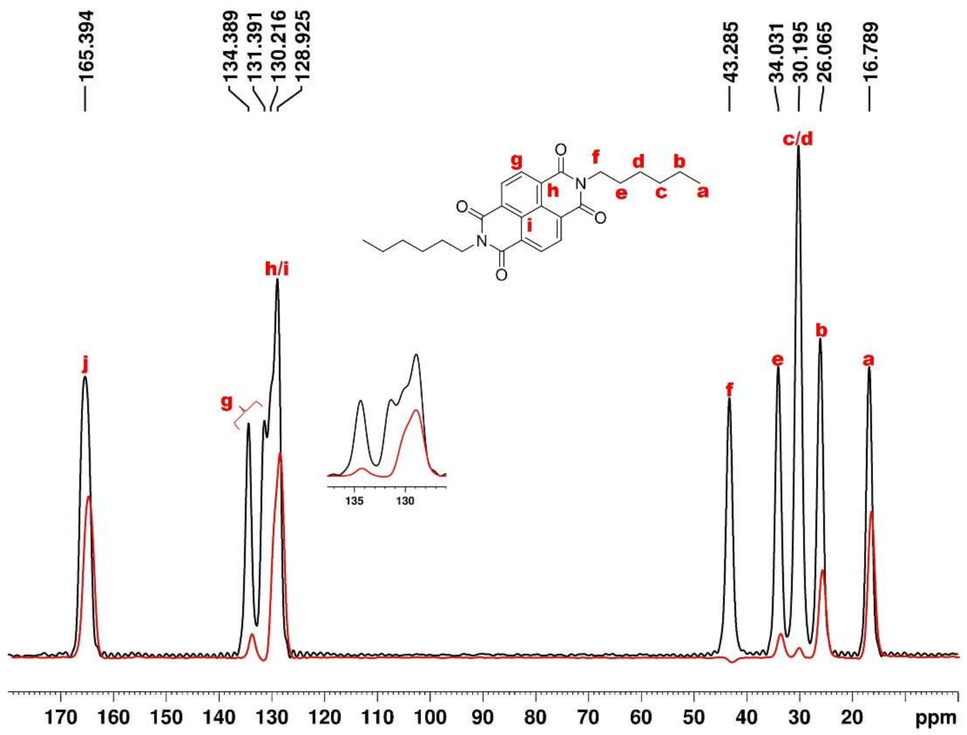

Figure 42. ^{13}C multiCP/MAS and ^{13}C multiCP/MAS with dipolar dephasing ssNMR spectra of NDI6.

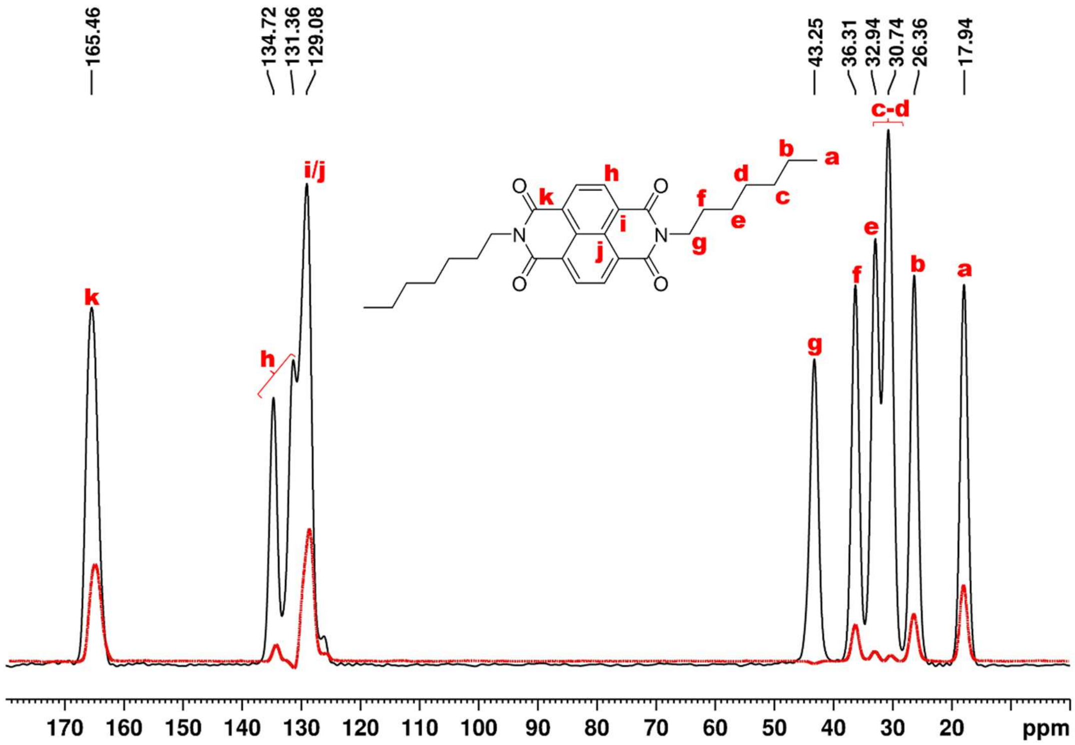

Figure 43. ^{13}C multiCP/MAS and ^{13}C multiCP/MAS with dipolar dephasing ssNMR spectra of NDI7.

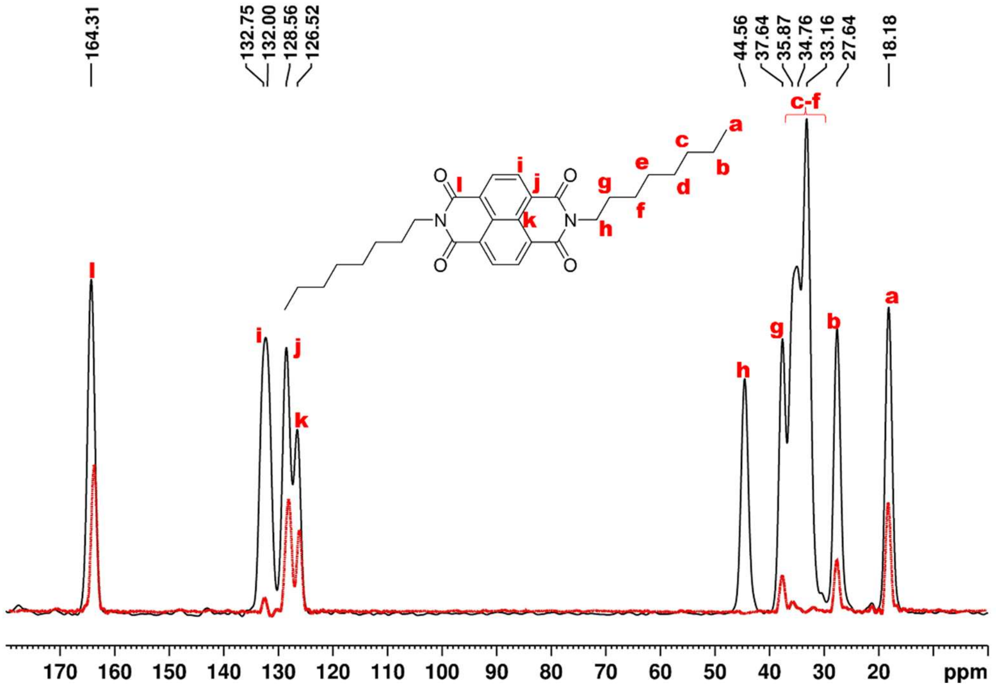

Figure 44. ^{13}C multiCP/MAS and ^{13}C multiCP/MAS with dipolar dephasing ssNMR spectra of NDI8.

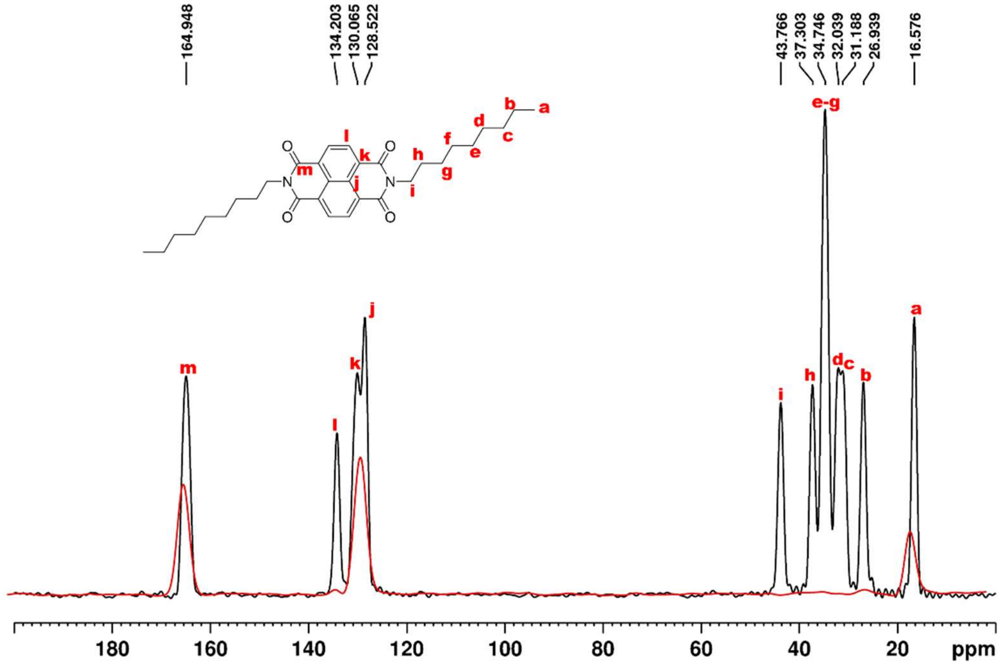

Figure 45. ^{13}C multiCP/MAS and ^{13}C multiCP/MAS with dipolar dephasing ssNMR spectra of NDI9.

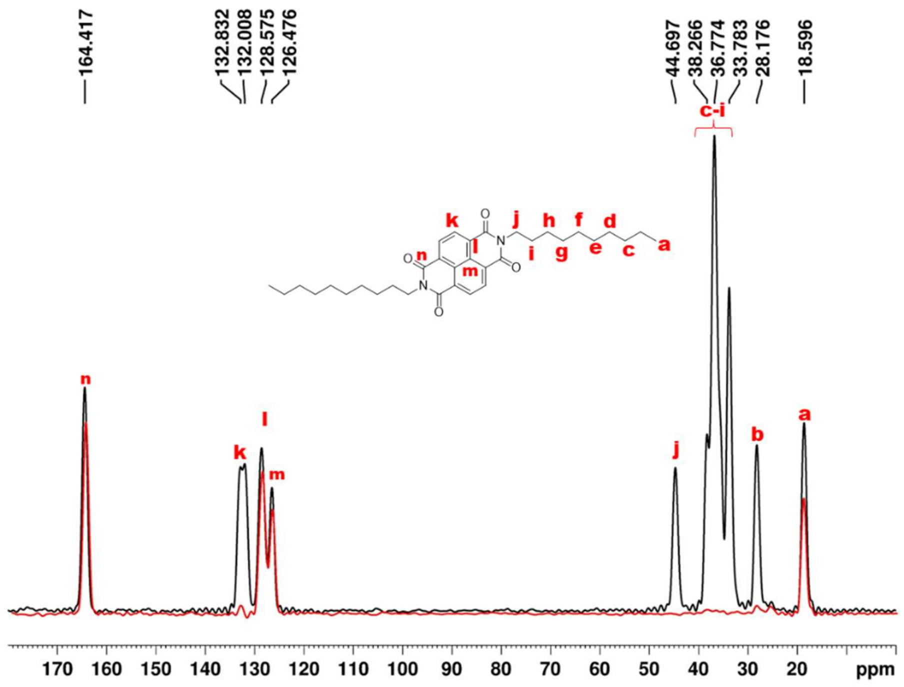

Figure 46. ^{13}C multiCP/MAS and ^{13}C multiCP/MAS with dipolar dephasing ssNMR spectra of NDI10.

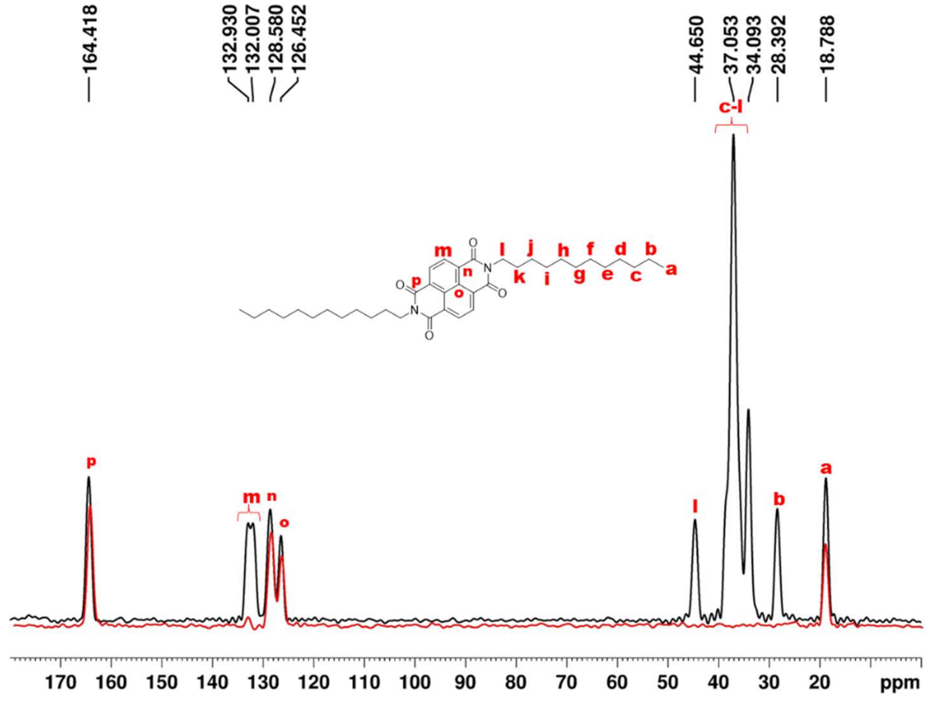

Figure 47. ^{13}C multiCP/MAS and ^{13}C multiCP/MAS with dipolar dephasing ssNMR spectra of NDI12.

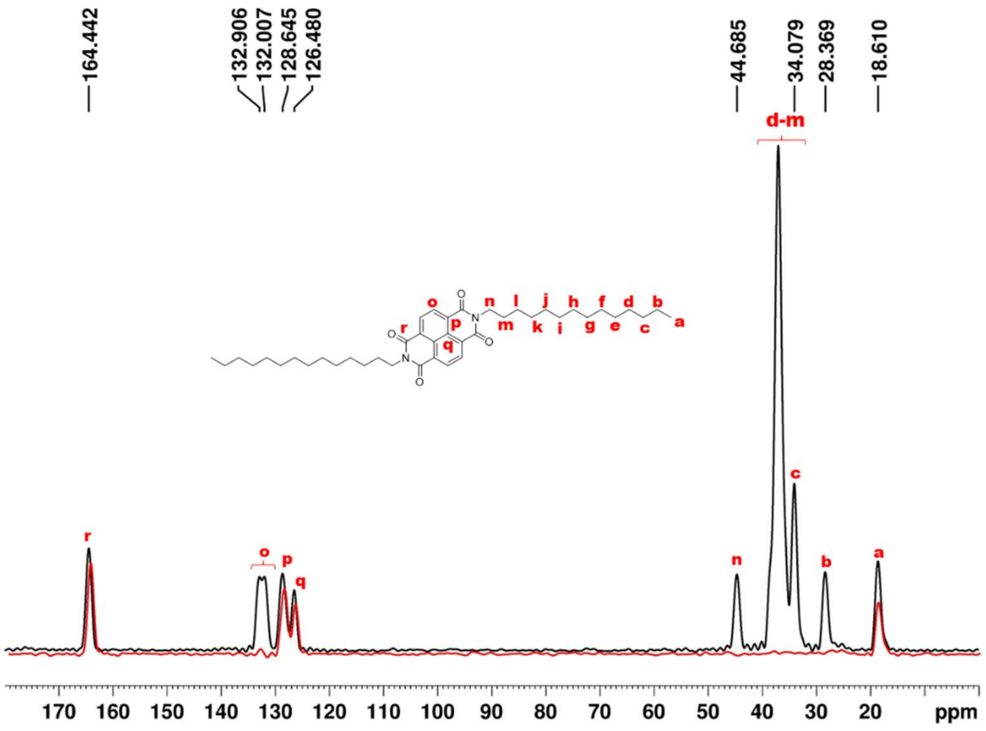

Figure 48. ^{13}C multiCP/MAS and ^{13}C multiCP/MAS with dipolar dephasing ssNMR spectra of NDI14.

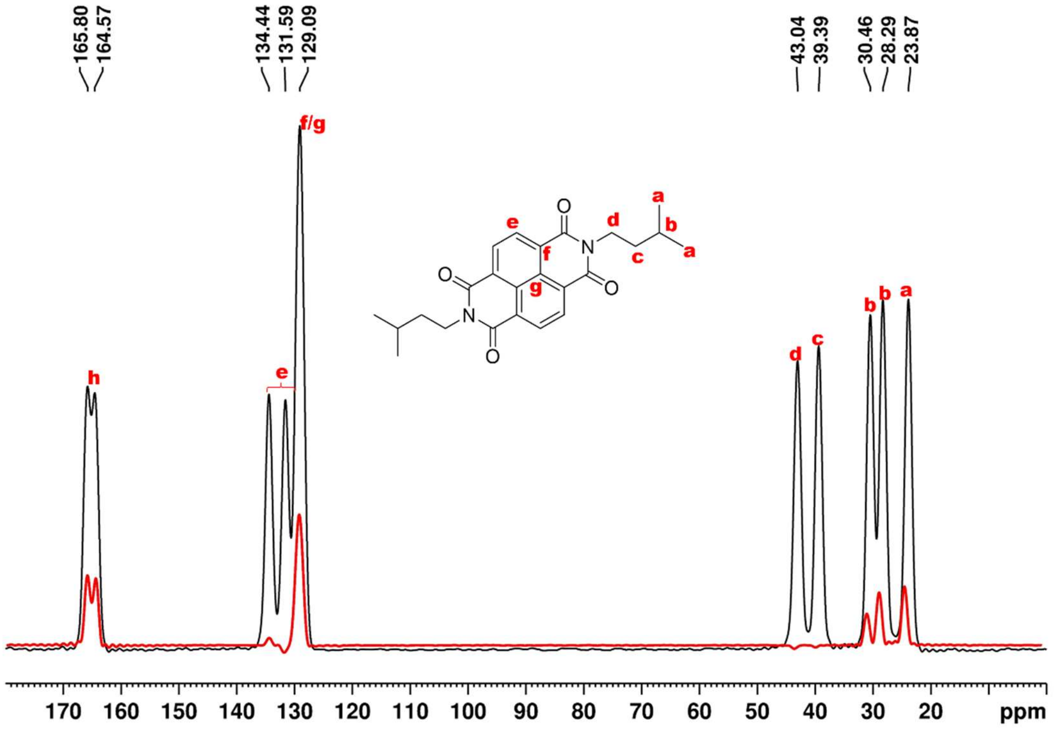

Figure 49. ^{13}C multiCP/MAS and ^{13}C multiCP/MAS with dipolar dephasing ssNMR spectra of NDI5a.

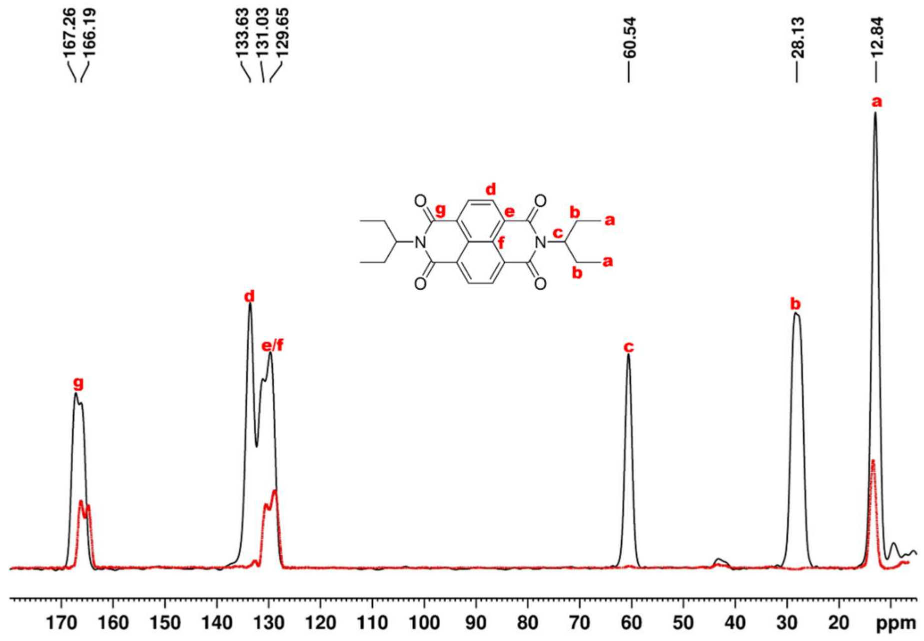

Figure 50. ^{13}C multiCP/MAS and ^{13}C multiCP/MAS with dipolar dephasing ssNMR spectra of NDI5b.

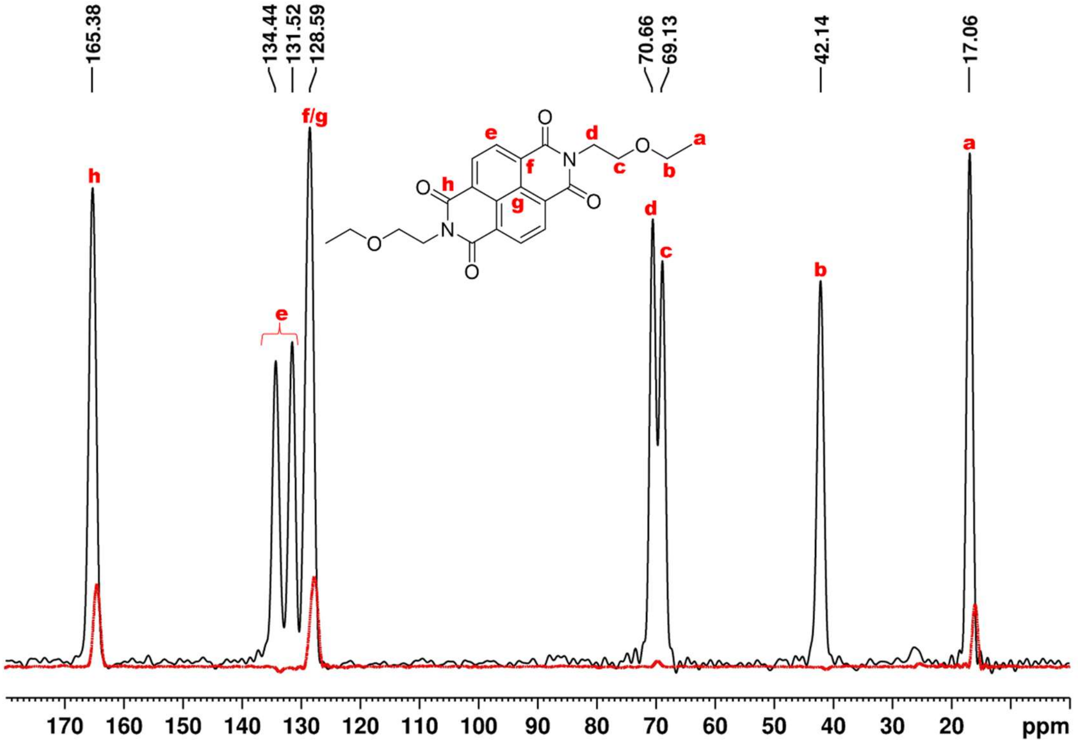

Figure 51. ^{13}C multiCP/MAS and ^{13}C multiCP/MAS with dipolar dephasing ssNMR spectra of NDIOa.

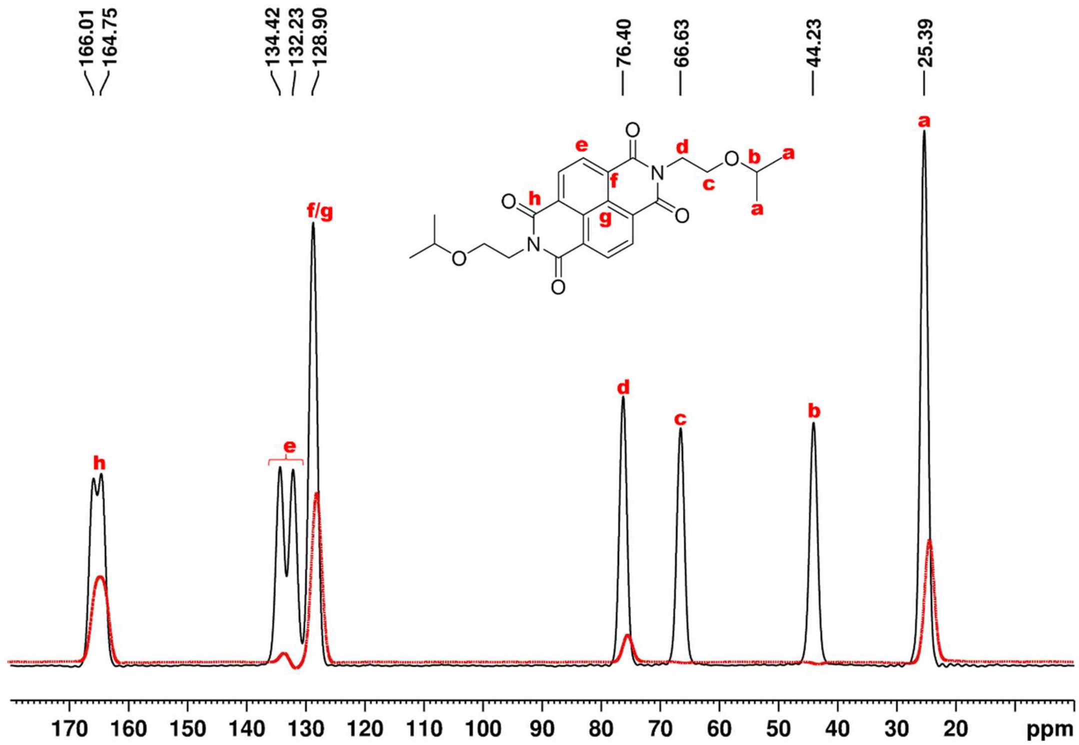

Figure 52. ^{13}C multiCP/MAS and ^{13}C multiCP/MAS with dipolar dephasing ssNMR spectra of NDIOb.

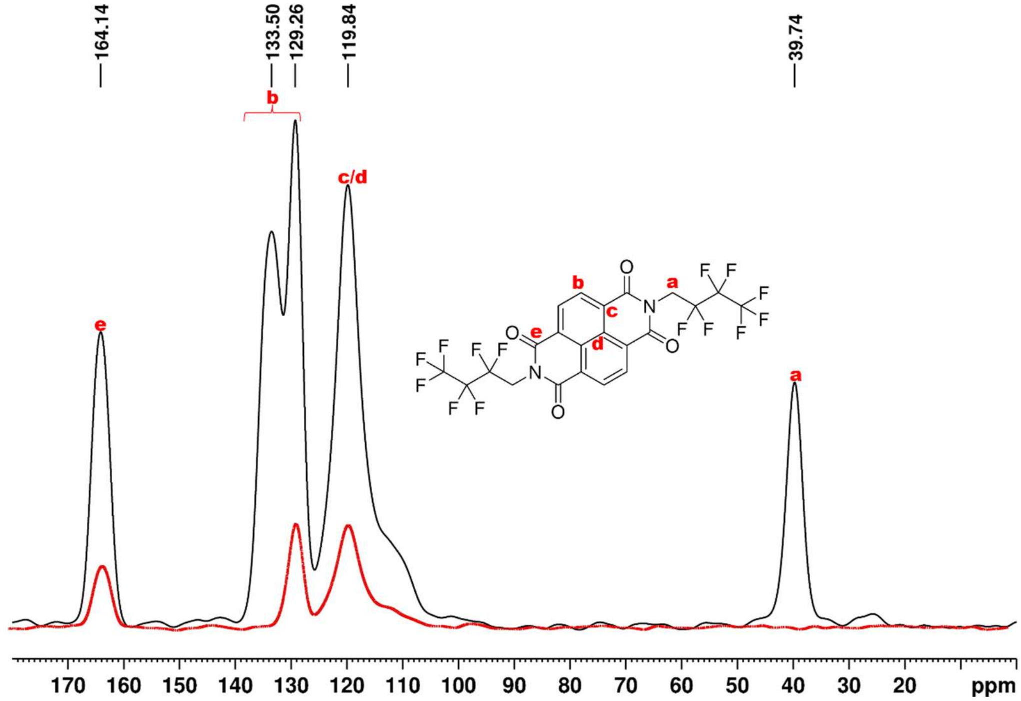

Figure 53. ^{13}C multiCP/MAS and ^{13}C multiCP/MAS with dipolar dephasing ssNMR spectra of NDIF.

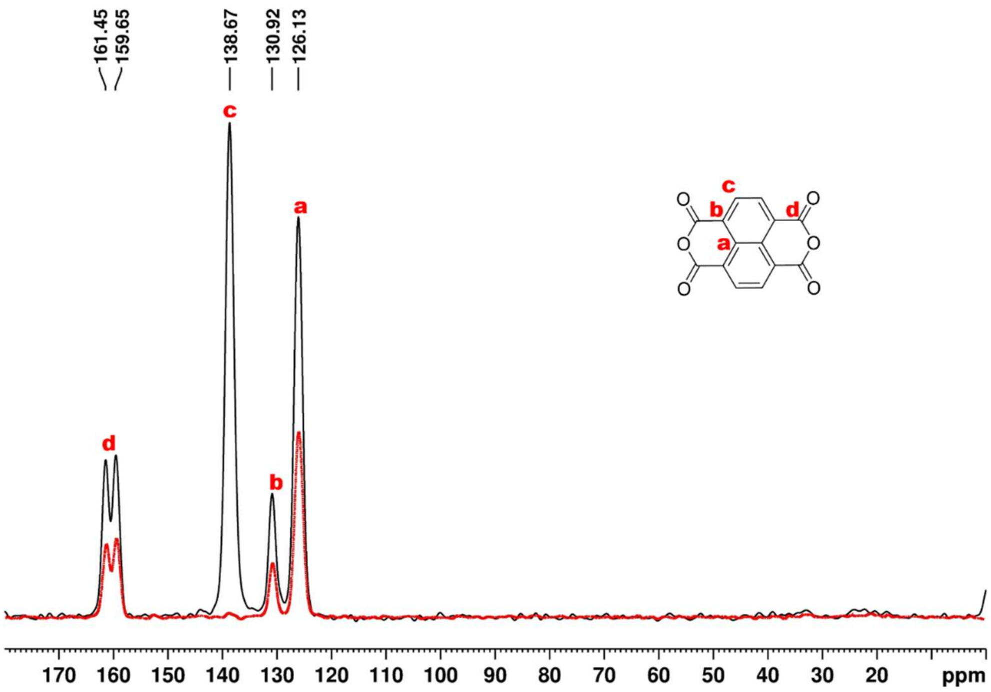

Figure 54. ^{13}C multiCP/MAS and ^{13}C multiCP/MAS with dipolar dephasing ssNMR spectra of NDA.

Table 1. NDI ^{13}C alkyl resonances for solid-state and solution NMR.

	**^{13}C alkyl resonances**	
**NDI**	Solution NMR	Solid-state NMR
**H**	N/A	N/A
**1**	27.40	30.28
**2**	36.06, 13.31	38.65, 16.35
**3**	42.42, 21.38, 11.45	45.67, 24.18, 16.47
**4**	40.75, 30.17, 20.33, 13.77	44.56, 33.89, 23.96, 17.60
**5**	40.96, 29.17, 27.75, 22.38, 13.94	43.44, 32.44, 29.28, 25.57, 17.53
**6**	40.99, 31.48, 28.03, 26.72, 22.52, 14.00.	43.29, 34.04, 30.20, 26.07, 16.79
**7**	41.00, 31.72, 28.97, 28.09, 27.04, 22.57, 14.04	43.25, 36.31, 32.94, 30.74, 26.36, 17.94
**8**	41.00, 31.79, 29.27, 29.17, 28.09, 27.08, 22.61, 14.05	44.56, 37.64, 35.87, 34,76, 33.16, 27.64, 18.18
**9**	41.00, 31.83, 29.47, 29.31, 29.23, 28.09, 27.08, 22.64, 14.07	43.77, 37.30, 34.75, 32.04, 31.19, 26.94, 16.68
**10**	41.00, 31.87, 29.53, 29.51, 29.31, 29.28, 28.09, 27.08, 22.66, 14.08	44.70, 38.23, 36.78, 33.79, 28.18, 18.60
**12**	41.00, 31.91, 29.62, 29.61, 29.57, 29.51, 29.32, 28.09, 27.08, 22.67, 14.09	44.65, 37.05, 34.09, 28.39, 18.79
**14**	41.00, 31.91, 29.65, 29.58, 29.51, 29.31, 28.09, 27.08, 22.67, 14.09	44.68, 37.03, 34.07, 28.37, 18.61
**5a**	39.56, 36.85, 26.45, 22.48	43.04, 39.39, 30.46, 28.29, 23.87
**5b**	58.14, 24.96, 11.24	60.54, 28.13, 12.85
**Oa**	67.02, 66.27, 39.82, 15.07	70.66, 69.13, 42.14, 17.06
**Ob**	71.58, 64.23, 40.28, 21.98	76.40, 66.63, 44.23, 25.39
**F**	38.64	39.74

Solid-state 2D ^{1}H -^{13}C HetCor spectra

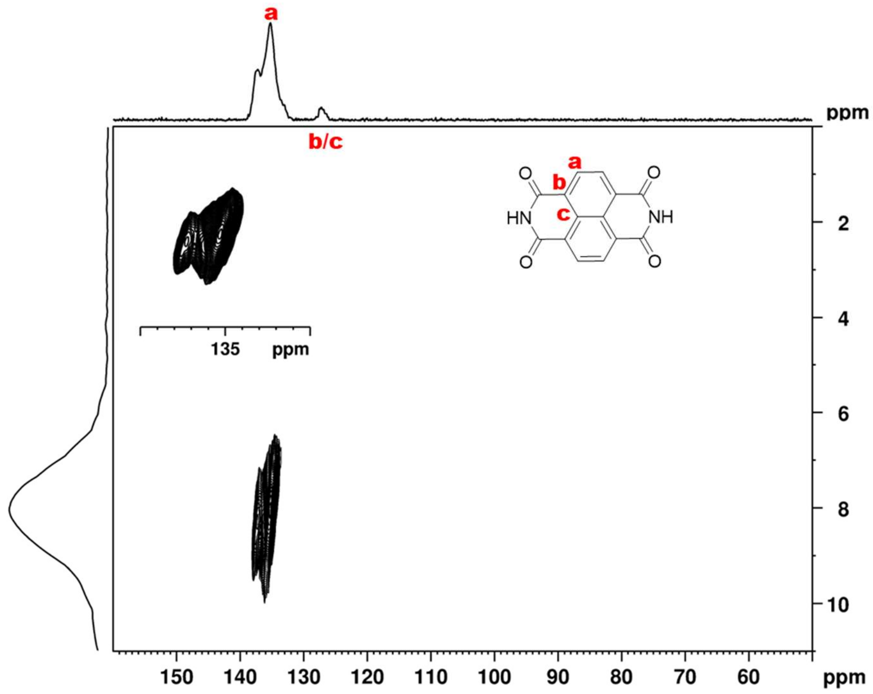

Figure 55. ^{1}H-^{13}C HetCor ssNMR spectrum of NDIH. Insets are zoomed in on aromatic regions of the molecule.

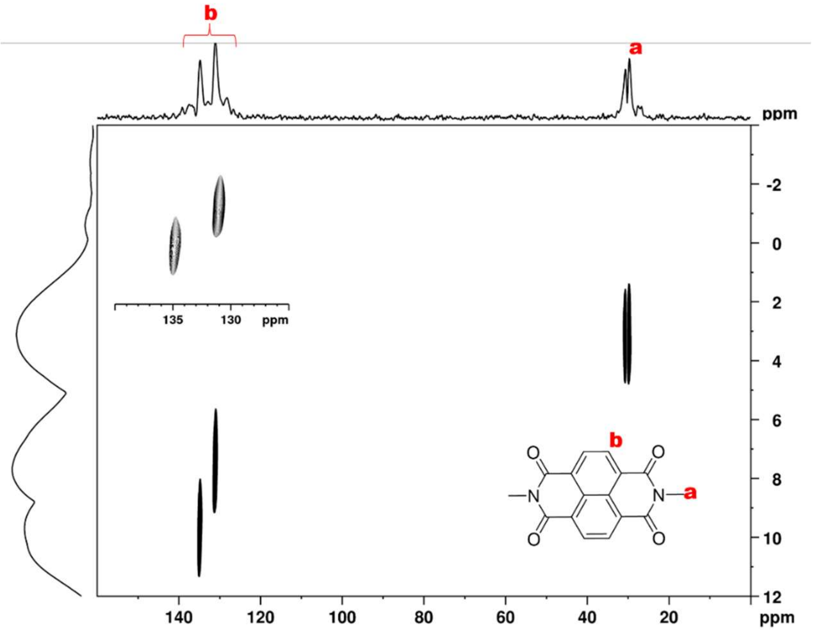

Figure 56. ^{1}H-^{13}C HetCor ssNMR spectrum of NDI1. Insets are zoomed in on aromatic regions of the molecule.

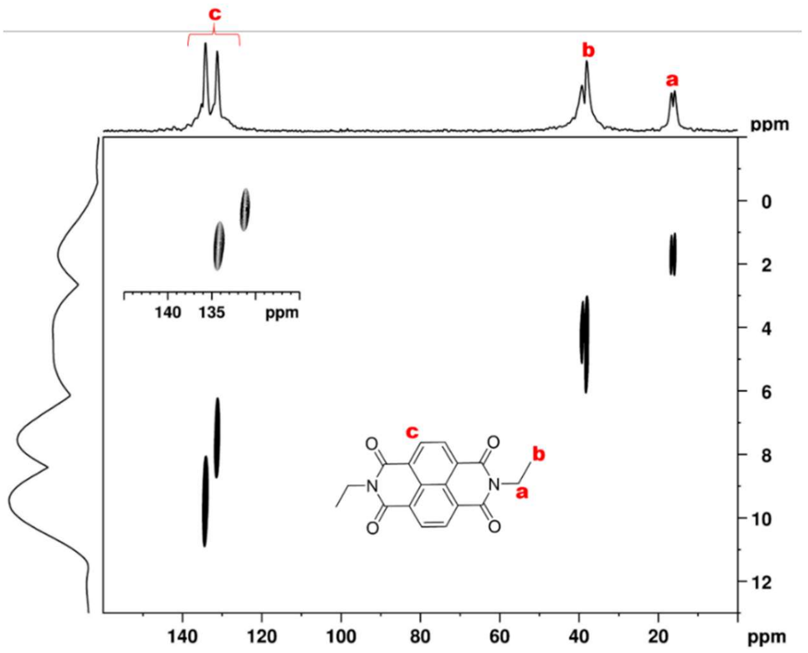

Figure 57. ^{1}H-^{13}C HetCor ssNMR spectrum of NDI2. Insets are zoomed in on aromatic regions of the molecule.

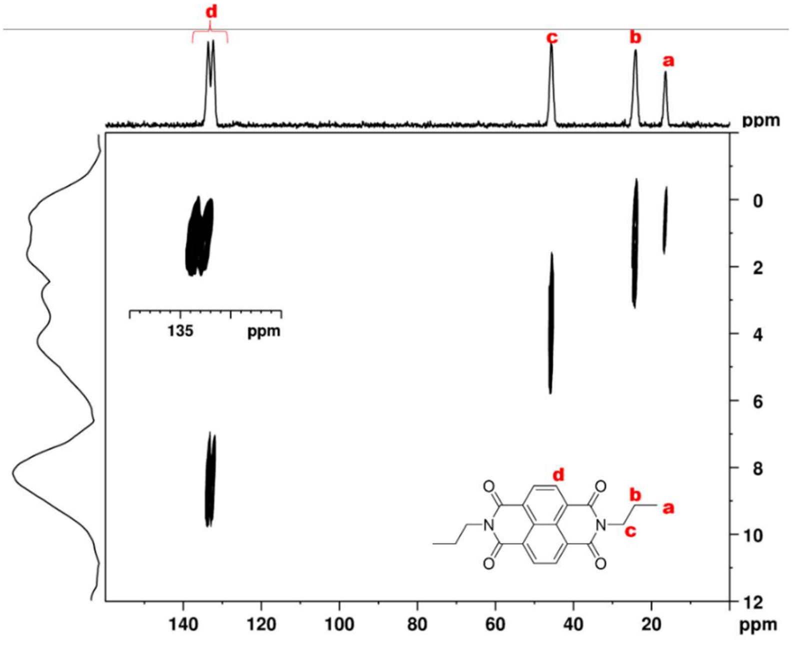

Figure 58. ^{1}H-^{13}C HetCor ssNMR spectrum of NDI3. Insets are zoomed in on aromatic regions of the molecule.

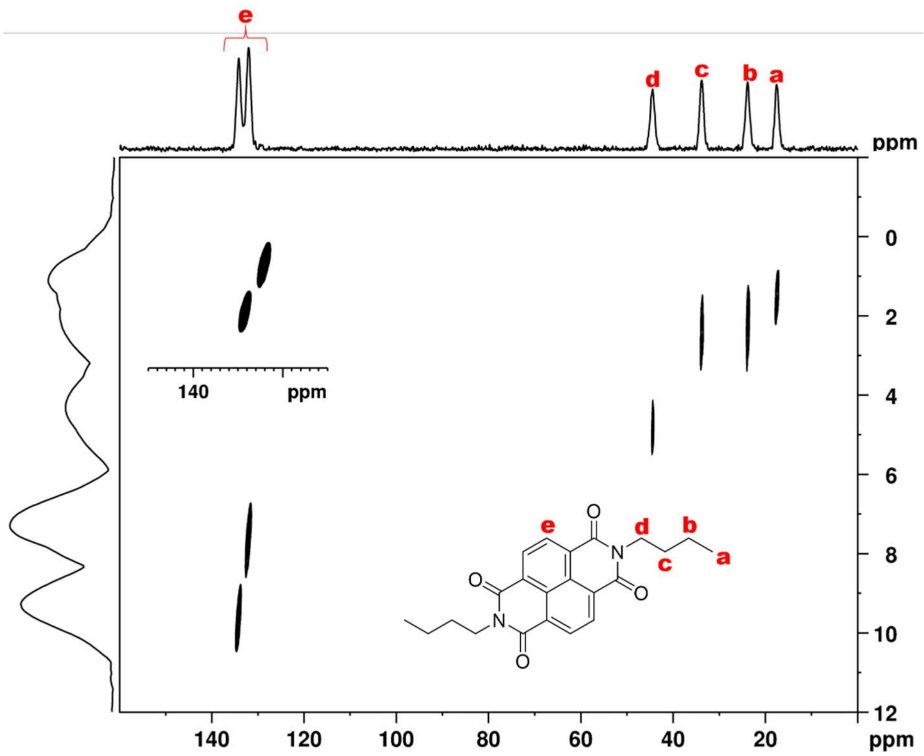

Figure 59. ^1H-^{13}C HetCor ssNMR spectrum of NDI4. Insets are zoomed in on aromatic regions of the molecule.

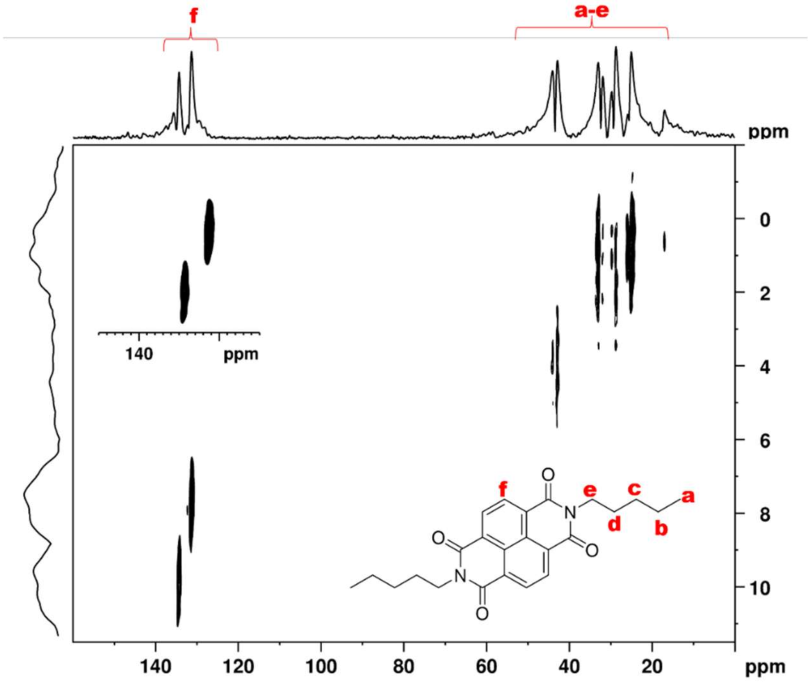

Figure 60. ^1H-^{13}C HetCor ssNMR spectrum of NDI5. Insets are zoomed in on aromatic regions of the molecule.

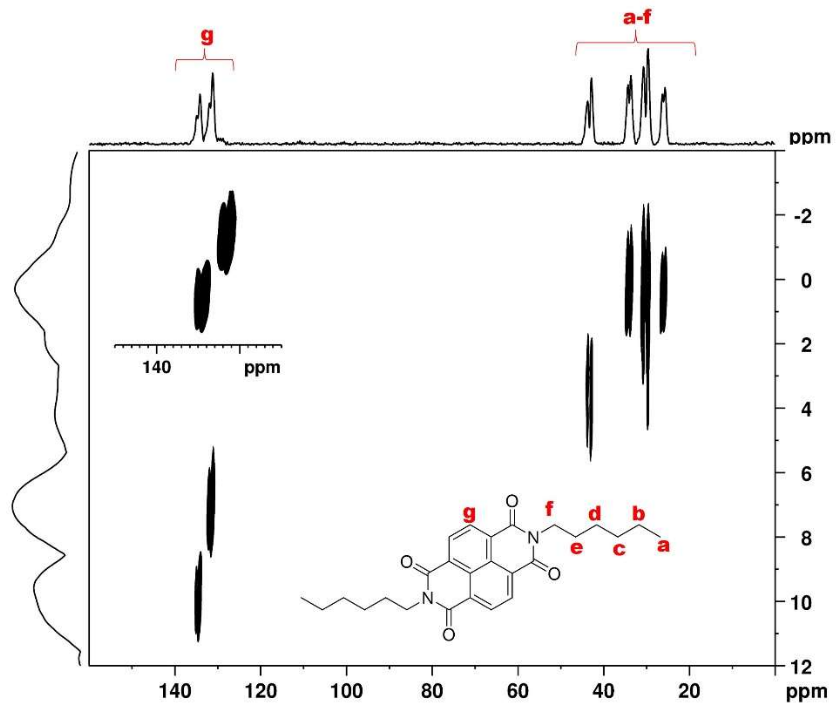

Figure 61. ^{1}H-^{13}C HetCor ssNMR spectrum of NDI6. Insets are zoomed in on aromatic regions of the molecule.

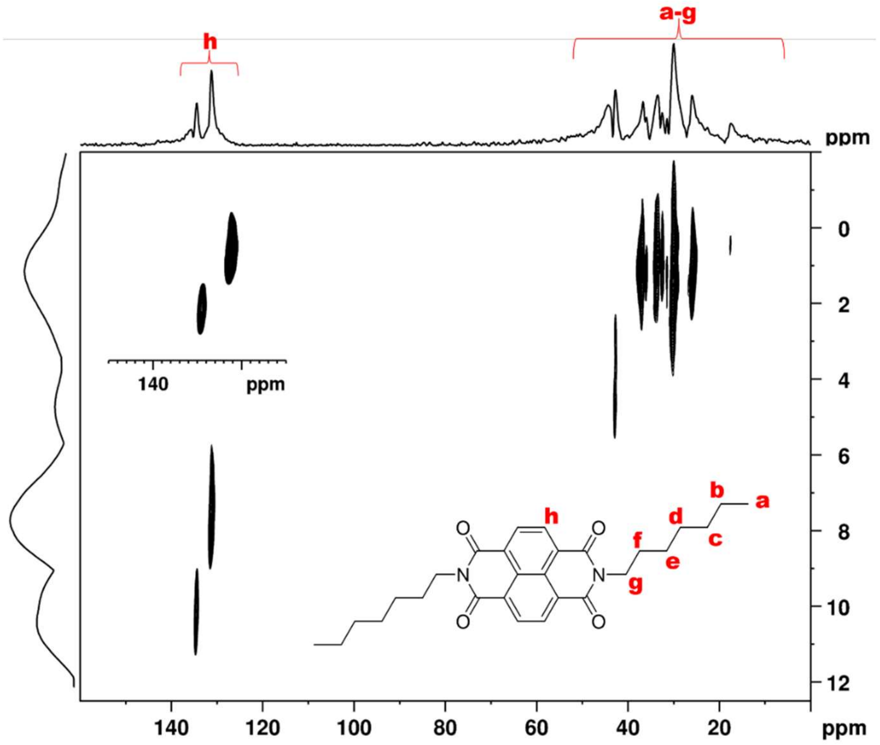

Figure 62. ^{1}H-^{13}C HetCor ssNMR spectrum of NDI7. Insets are zoomed in on aromatic regions of the molecule.

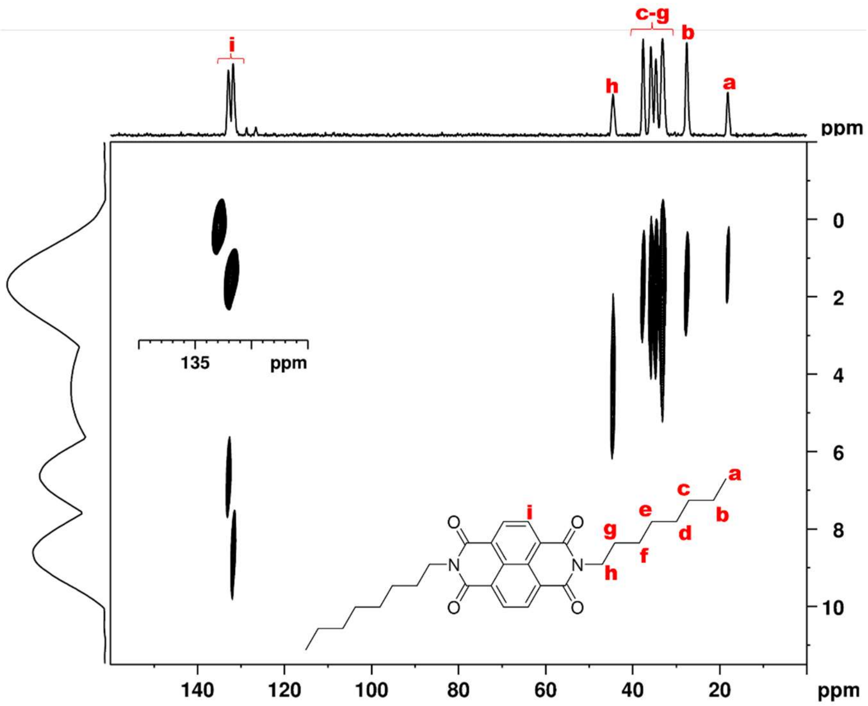

Figure 63. ^{1}H-^{13}C HetCor ssNMR spectrum of NDI8. Insets are zoomed in on aromatic regions of the molecule.

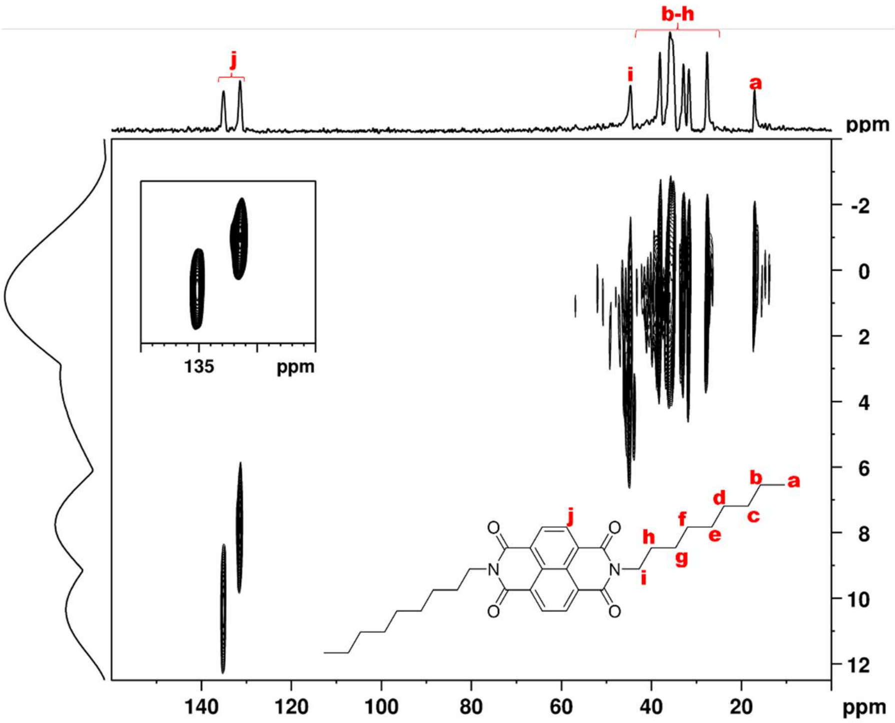

Figure 64. ^{1}H-^{13}C HetCor ssNMR spectrum of NDI9. Insets are zoomed in on aromatic regions of the molecule.

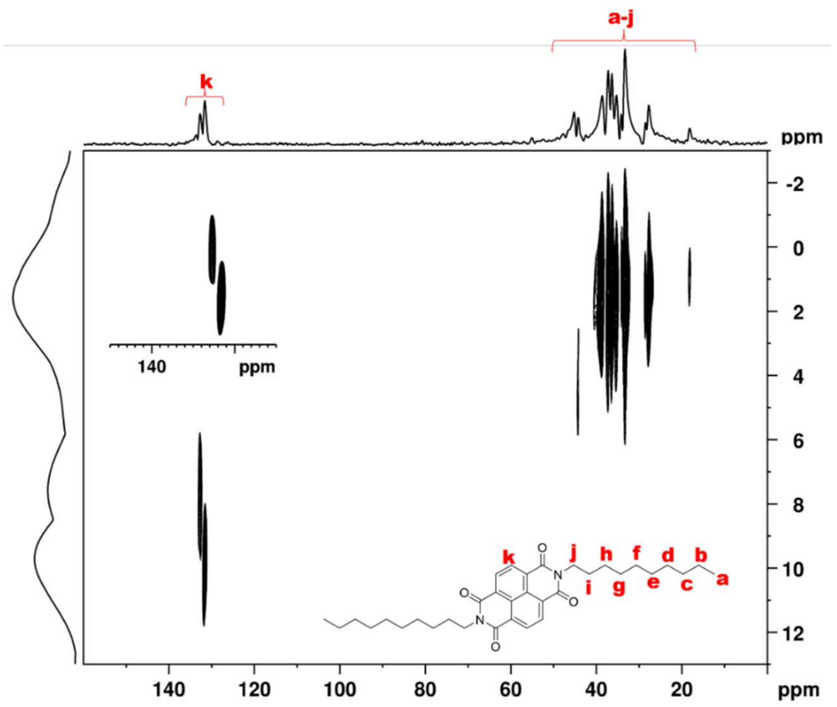

Figure 65. ^{1}H-^{13}C HetCor ssNMR spectrum of NDI10. Insets are zoomed in on aromatic regions of the molecule.

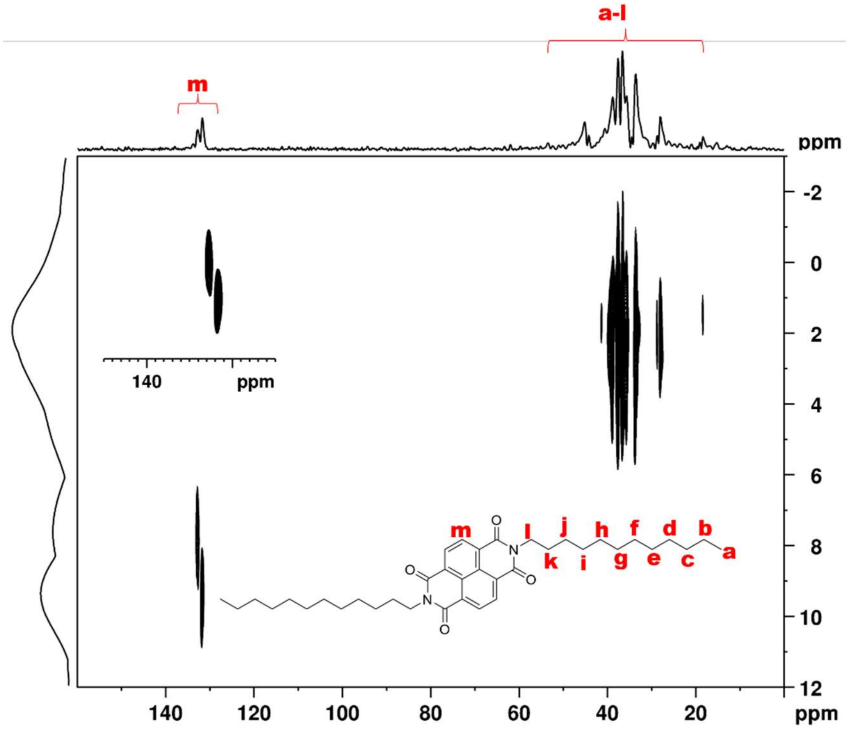

Figure 66. ^{1}H-^{13}C HetCor ssNMR spectrum of NDI12. Insets are zoomed in on aromatic regions of the molecule.

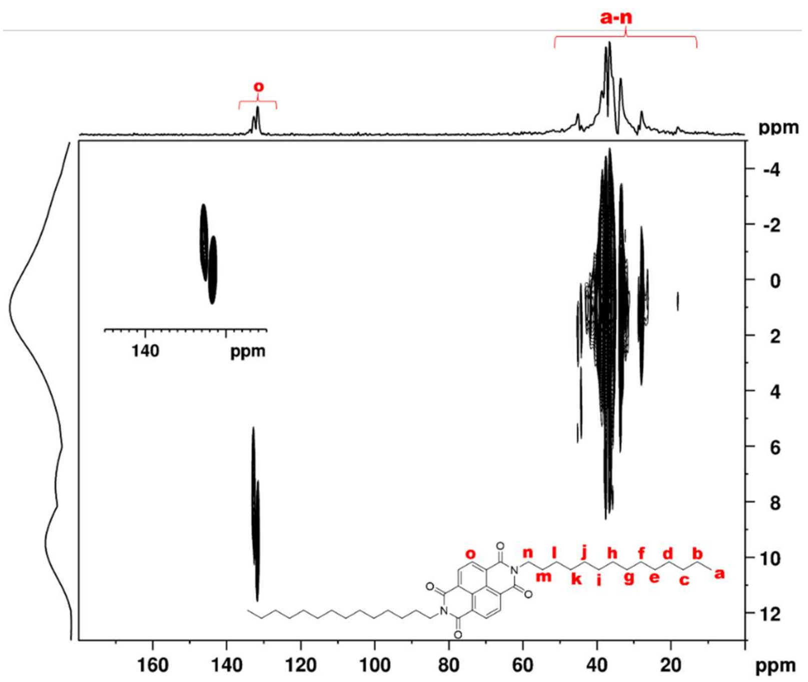

Figure 67. ^{1}H-^{13}C HetCor ssNMR spectrum of NDI14. Insets are zoomed in on aromatic regions of the molecule.

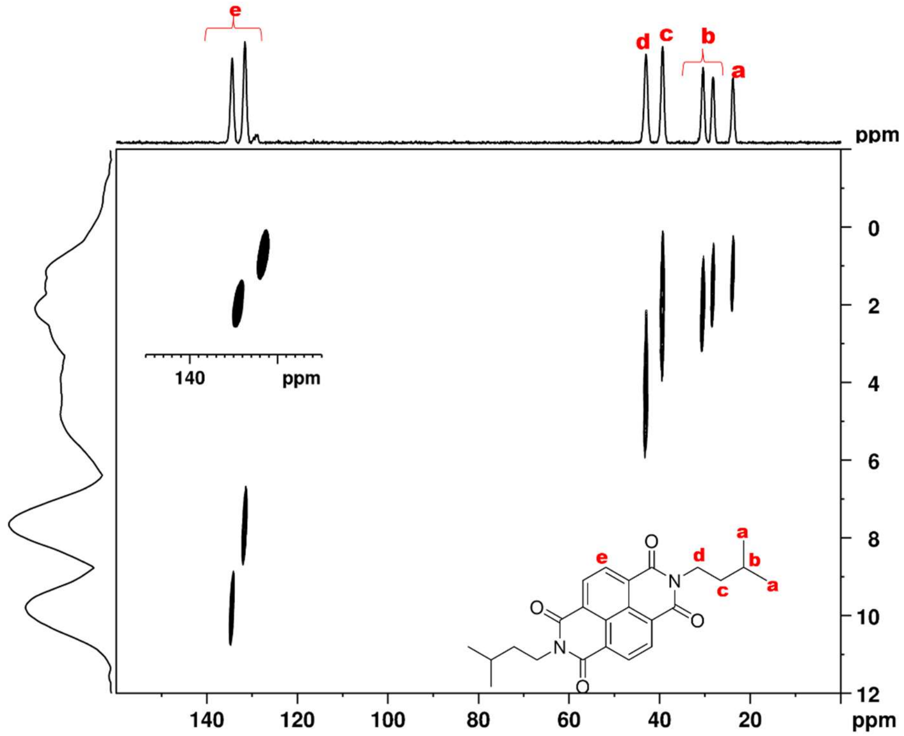

Figure 68. ^{1}H-^{13}C HetCor ssNMR spectrum of NDI5a. Insets are zoomed in on aromatic regions of the molecule.

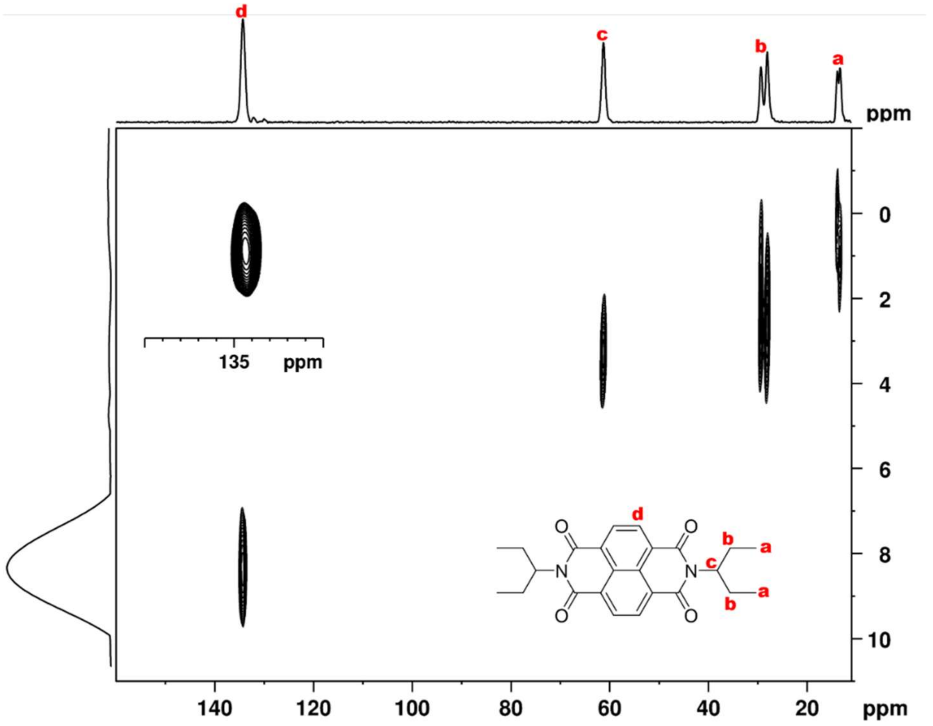

Figure 69. ^{1}H-^{13}C HetCor ssNMR spectrum of NDI5b. Insets are zoomed in on aromatic regions of the molecule.

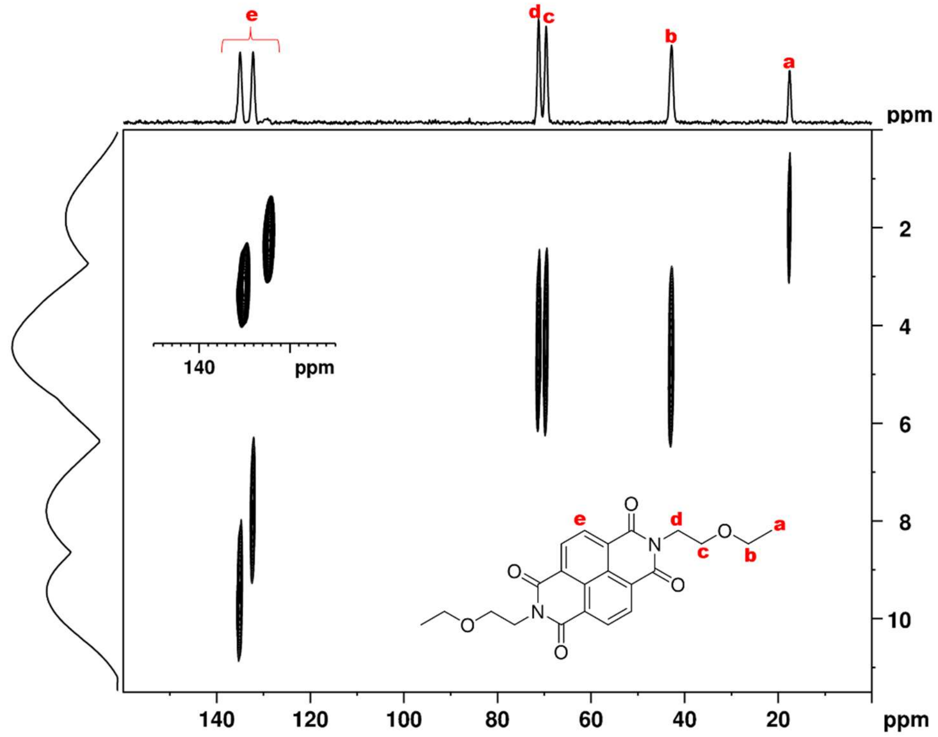

Figure 70. ^{1}H-^{13}C HetCor ssNMR spectrum of NDIOa. Insets are zoomed in on aromatic regions of the molecule.

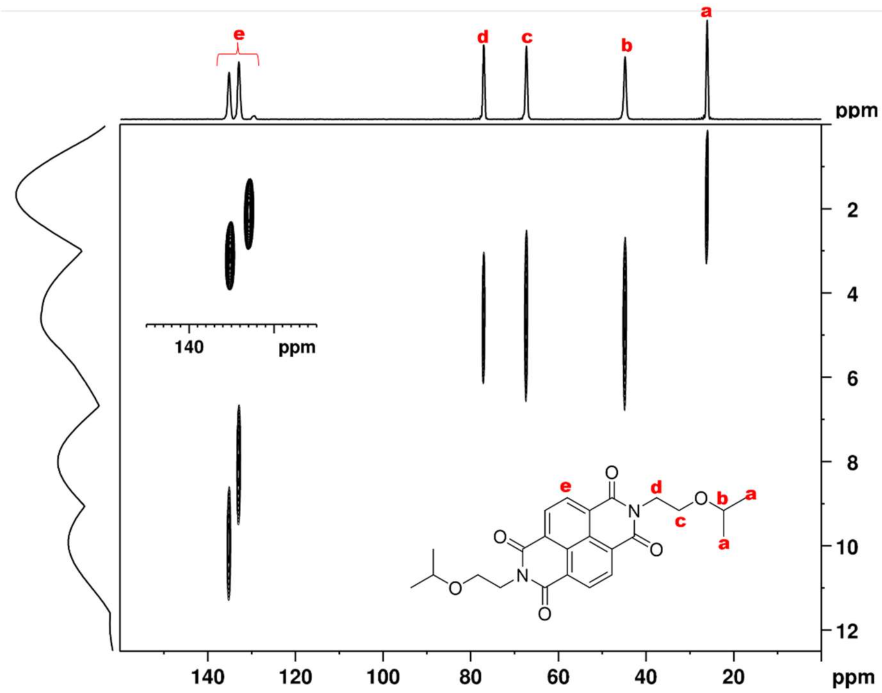

Figure 71. ^{1}H-^{13}C HetCor ssNMR spectrum of NDIOb. Insets are zoomed in on aromatic regions of the molecule.

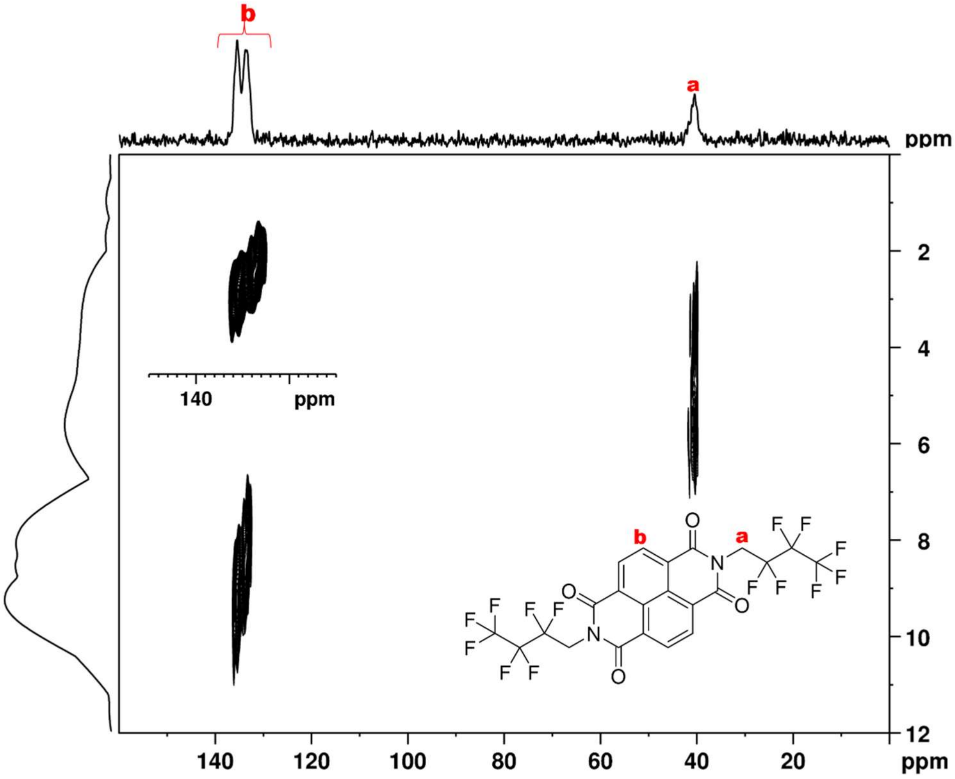

Figure 72. ^{1}H-^{13}C HetCor ssNMR spectrum of NDIF. Insets are zoomed in on aromatic regions of the molecule.

Crystallography

Crystals of **NDI1**, **NDI5a**, **NDIOa**, and **NDIOb** were grown by solvent diffusion from chloroform solution. X-ray diffraction data for **NDI1**, **NDI5a**, **NDIOa**, and **NDIOb** were collected using crystals that were mounted on glass fibers or on MiTeGen Micro Mounts. All measurements were made using either Cu Kα (**NDI1**) or Mo Kα (**NDI5a**) radiation on a Bruker-AXS Apex three-circle diffractometer, equipped with microfocus Cu and fine-focus Mo sealed tubes and a CCD detector, or using Mo Kα (**NDIOa**, **NDIOb**) radiation on a Bruker-AXS D8 Venture four-circle diffractometer, equipped with a microfocus tube and a Photon 3 CPAD detector. Initial space group determination was based on a matrix consisting of 36 or fast scan with 180 frames. Data sets were collected at 296 K. The data were reduced using SAINT+[8], and empirical absorption correction applied using SADABS.[9] Structures were solved using intrinsic phasing. Least-squares refinement for all structures was carried out on F^2. The non-hydrogen atoms were refined anisotropically. Hydrogen atoms were placed in riding positions and refined isotropically. Structure solution, refinement, and the calculation of derived results were performed using the SHELXTL package of computer programs[10] and ShelXle.[11] Further details regarding data collection and refinement parameters will be deposited with the Cambridge Crystallographic Data Centre (CCDC) [2444543-2444546]. Crystallographic data for the remaining NDIs [**NDIH**,155699[12]; **NDI2**, 937648[13]; **NDI3**, 1029340[14], **NDI4**, 819749[15]; **NDI5**, 238148[16]; **NDI6**, 804310[17], **NDI7**, 2242898[18], **NDI8**, 1972223[19]; **NDI9**, 2242899[20]; **NDI10**, 1972224[21]; **NDI12**, 819750[22]; **NDI14**, 230429[23]; **NDI5b**, 1551374[24]; **NDIF**, 712434[25]] were retrieved from the Cambridge Structural Database (CSD). Following synthesis, many NDIs had apparent amorphous precipitates. To verify that published crystal structure data mirrored our samples, a crystal structure of NDI1 was grown for comparison. The original deposit for NDI1[14] was similar to our solved structure [CCDC 2444543]. Detailed crystal and structure refinement data of NDIs solved and submitted in this publication are summarized in Table 2. Crystal structure summaries and calculations for all NDIs are in Tables 3 and 4.

Table 2. Crystal and Structure Refinement Data of NDIs.

	NDI1	NDI5a	NDIOa	NDIOb
CCDC deposit no.	2444543	2444546	2444544	2444545
colour and habit	orange needle	pink blade	colorless blade	colorless blade
size, mm	0.66 × 0.06 × 0.03	0.63 × 0.17 × 0.07	0.56 × 0.08 × 0.03	0.32 × 0.07 × 0.02
Formula	$C_{16}H_{10}N_2O_4$	$C_{24}H_{26}N_2O_4$	$C_{22}H_{22}N_2O_6$	$C_{24}H_{26}N_2O_6$
formula weight	294.26	406.47	410.41	438.47
space group	$P2_1/c$	*P-1*	$P2_{1/n}$	$P2_1/n$
a, Å	4.62210(10)	4.8470(12)	7.7579(3)	4.7132(4)
b, Å	8.0344(2)	7.784(2)	4.6611(2)	28.726(2)
c, Å	17.0421(5)	13.965(4)	26.2104(10)	7.7578(6)
α, deg	90	79.654(4)	90	90
β, deg	94.080(2)	82.107(4)	92.3110(10)	90.909(2)
γ, deg	90	89.651(5)	90	90
volume, Å³	631.27(3)	513.3(2)	947.01(7)	1050.21(15
Z	2	1	2	2
ρ_{calc}, g cm⁻³	1.548	1.315	1.439	1.387
F_{000}	304	216	432	464
μ(Mo Kα), mm⁻¹	0.100	0.090	0.106	0.100
Temp., K	296(2)	100(2)	100(2)	100(2)
residuals:[a] R; R_w	0.439, 0.1130	0.0451, 0.0990	0.0519, 0.1125	0.0737, 0.1849
goodness of fit	1.080	1.055	1.042	1.209
peak and hole, eÅ⁻³	0.195 and -0.174	0.275 and -0.200	0.258 and -0.259	0.313 and -0.322

Table 3. Crystallographic and ssNMR Data for NDI Compounds including space group, unit cell dimensions (a, b, c), angles (α, β, γ), cell type, packing arrangement, slip distance (dslip), stacking distance (dstack), and ssNMR spectral type are listed.

		Crystal Structure Summary										ssNMR
	CCDC #	Symmetry	Dimensions (Å)			Angles (°)			Bulk packing	d_{slip}	d_{stack}	1D /2Dtype
NDI			a	b	c	α	B	γ				
H	155699[a]	P-1	7.63	8.20	8.98	90.0	72.0	79.9	Unidirectional	3.00	3.34	F1/Ia
1	2444543[b]	$P2_1/c$	4.67	8.01	16.42	90.0	94.1	90.0	Bidirectional	3.20	3.34	F2/IIa
2	937648[a]	$P2_1/c$	4.87	7.76	18.38	90.0	90.2	90.0	Bidirectional	3.61	3.26	F2/IIa
3	1029340[a]	Pbca	6.96	17.24	27.58	90.0	90.0	90.0	Zigzag	0.97	3.35	F3/Ib
4	819749[a]	P-1	5.22	7.84	11.13	103.7	94.3	93.8	Unidirectional	4.05	3.30	F4/IIa
5	238148[a]	$P2_1/n$	5.03	8.11	24.21	90.0	90.8	90.0	Bidirectional	3.83	3.25	F2/IIa
6	804310[a]	P-1	4.82	8.30	14.26	96.6	97.5	93.0	Unidirectional	3.47	3.26	F4/IIa
7	2242898[a]	$P2_1/n$	7.87	4.84	33.02	90.0	95.0	90.0	Bidirectional	3.54	3.24	F2/IIa
8	1972223[a]	P-1	4.60	6.64	21.53	90.6	91.6	104.7	Unidirectional	3.19	3.31	F5/IIb
9	2242899[a]	$P2_1/c$	7.85	4.84	37.74	90.0	95.0	90.0	Bidirectional	3.57	3.29	F2/IIa
10	1972224[a]	P-1	4.75	6.55	25.79	94.4	95.2	104.4	Unidirectional	3.42	3.29	F5/IIb
12	819750[a]	P-1	4.61	6.59	27.73	93.3	94.5	104.6	Unidirectional	3.22	3.30	F5/IIb
14	230429[a]	P-1	4.64	6.64	30.90	89.8	88.4	75.6	Unidirectional	3.22	3.33	F5/IIb

Table 4. Comparison of crystallographic and ssNMR data for NDI5a, NDI5b, NDIF, NDIOa, and NDIOb.

		Crystal Structure Summary										ssNMR
	CCDC #	Symmetry	Dimensions (Å)			Angles (°)			Bulk packing	d_{slip}	d_{stack}	1D/2D type
NDI			a	b	c	A	B	γ				
**5a**	2444546[a]	P-1	4.85	7.78	13.96	79.6	82.1	89.6	Unidirectional	3.60	3.25	F2/IIa
**5b**	1551374[a]	$P2_1/c$	9.14	8.48	13.46	90.0	100.5	90.0	Bidirectional	N/A	3.23	F6/III
**Oa**	2444544[a]	$P2_1/n$	7.76	4.66	26.21	90.0	92.3	90.0	Bidirectional	3.36	3.23	F2/IIa
**Ob**	2444545[a]	$P2_1/n$	4.71	28.73	7.76	90.0	90.9	90.0	Bidirectional	3.43	3.48	F2/IIa
**F**	712434[b]	P-1	5.19	10.14	11.60	66.7	79.0	89.1	Unidirectional	3.85	3.34	F7/IIc

a. This work

b. See reference in Supporting Information

Powder X-ray

Powder X-ray diffraction (PXRD) traces were collected on a Thermo Scientific ARL Equinox 100 with Co radiation. Data were subsequently converted to Cu wavelength.

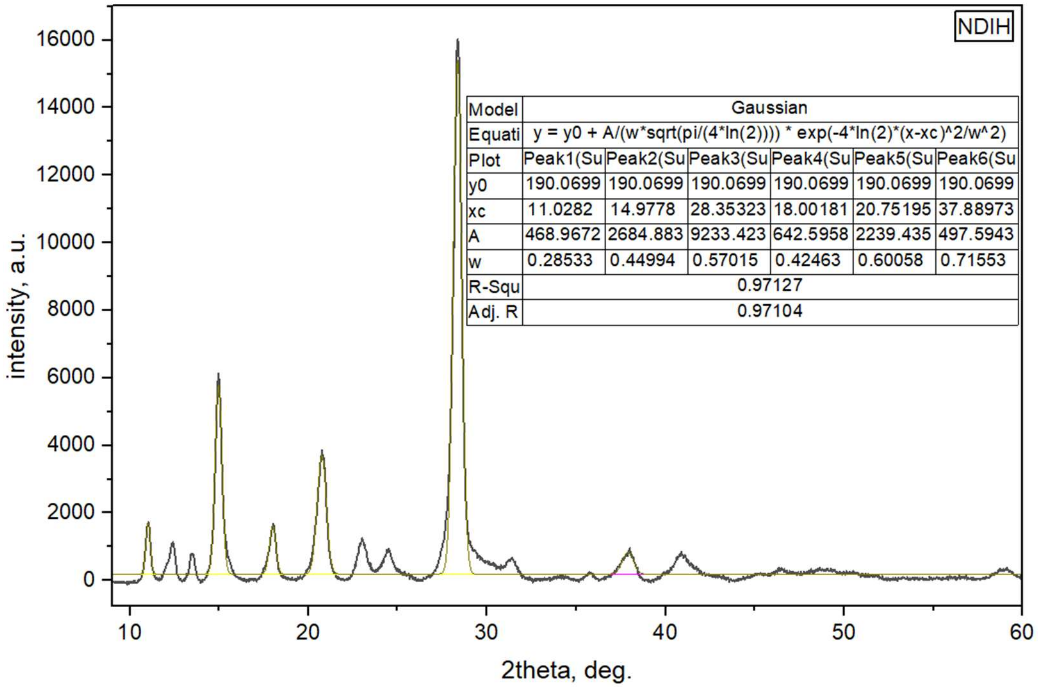

Figure 73. PXRD trace of NDIH and fwhm calculations.

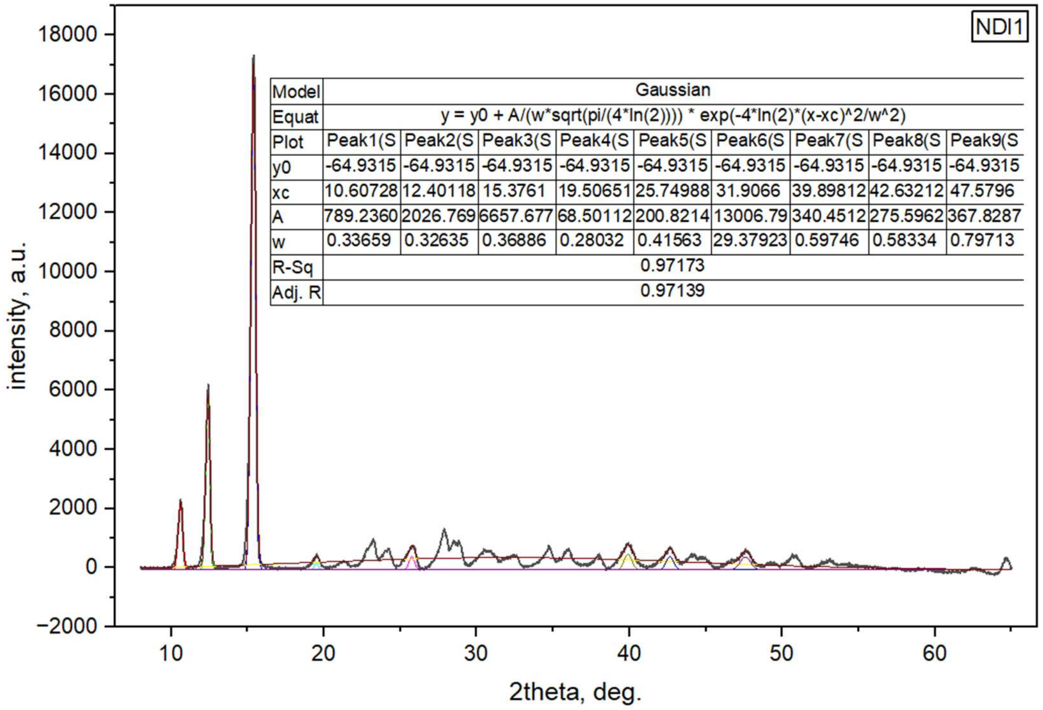

Figure 74. PXRD trace of NDI1 and fwhm calculations.

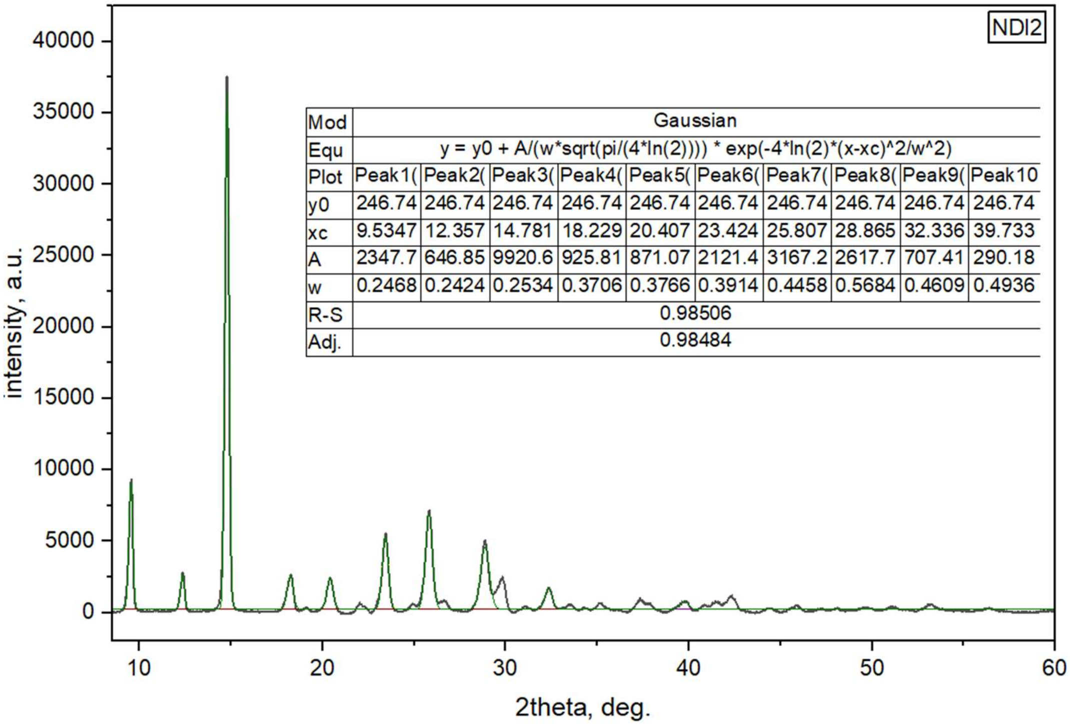

Figure 75. PXRD trace of NDI2 and fwhm calculations.

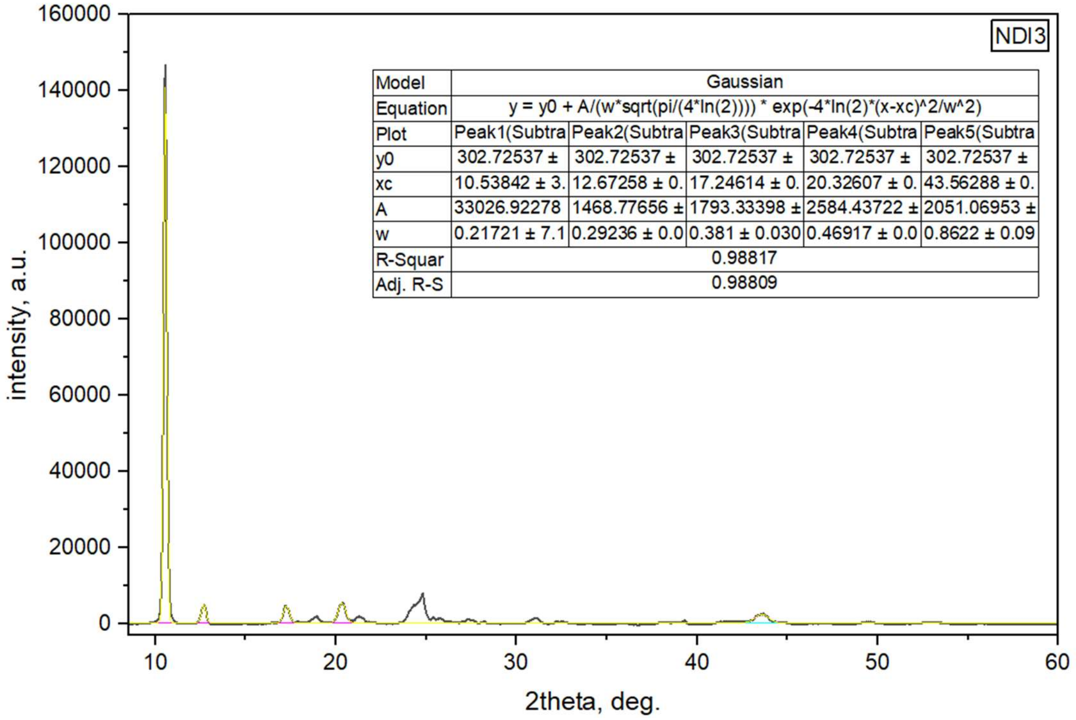

Figure 76. PXRD trace of NDI3 and fwhm calculations.

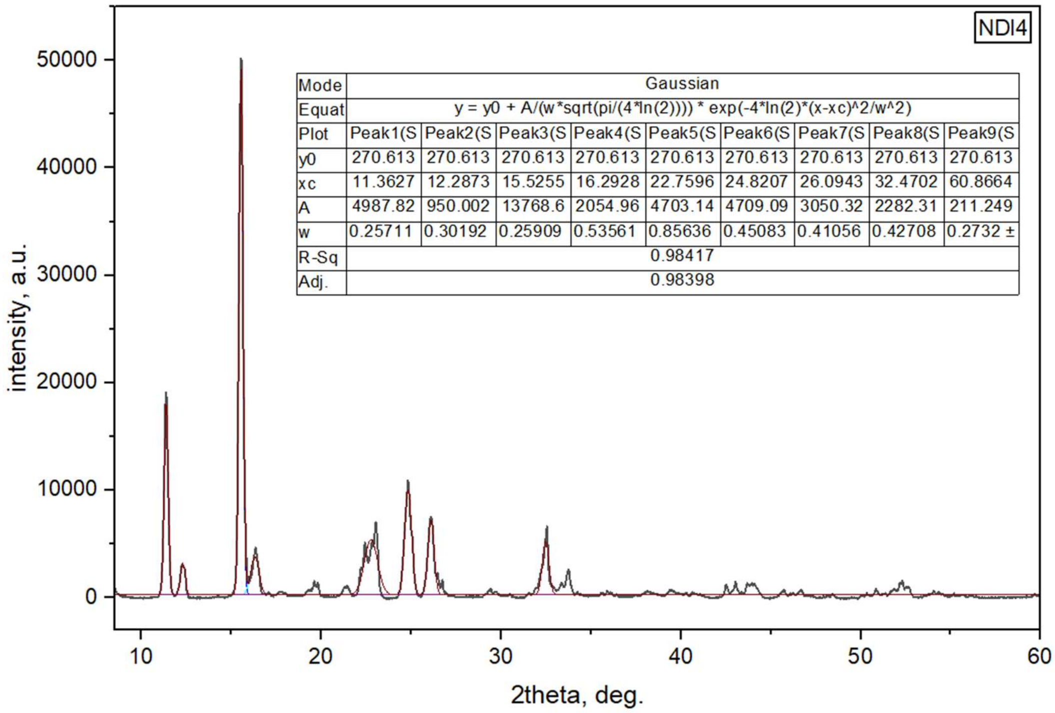

Figure 77. PXRD trace of NDI4 and fwhm calculations.

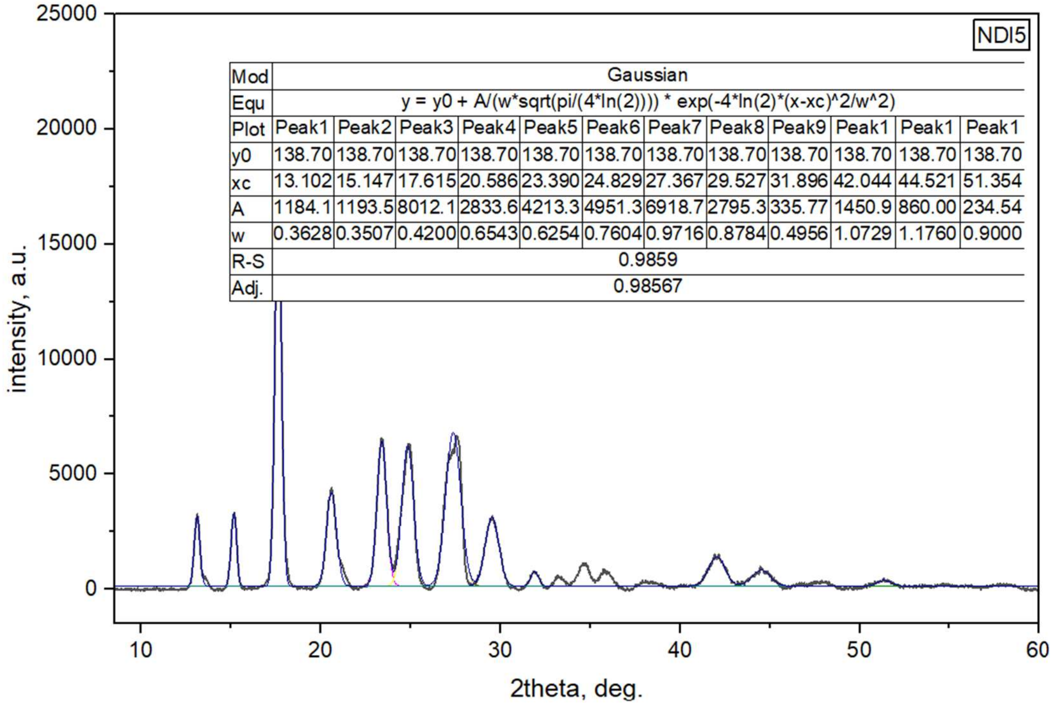

Figure 78. PXRD trace of NDI5 and fwhm calculations.

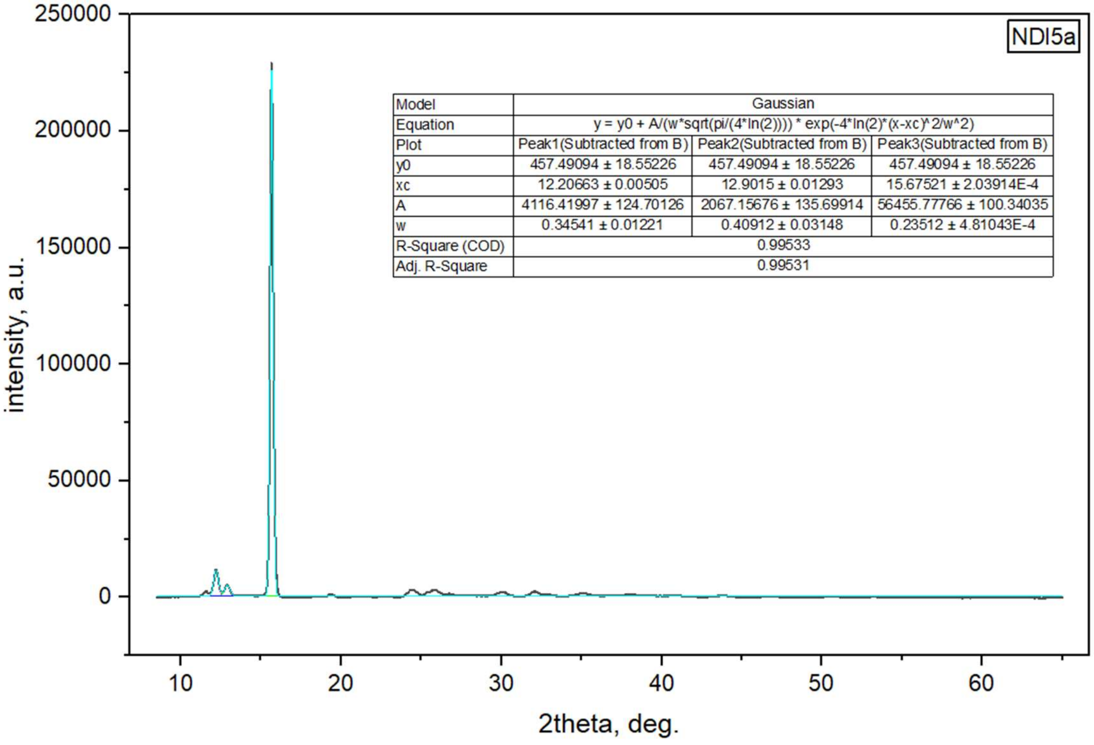

1

Figure 79. PXRD trace of NDI5a and fwhm calculations.

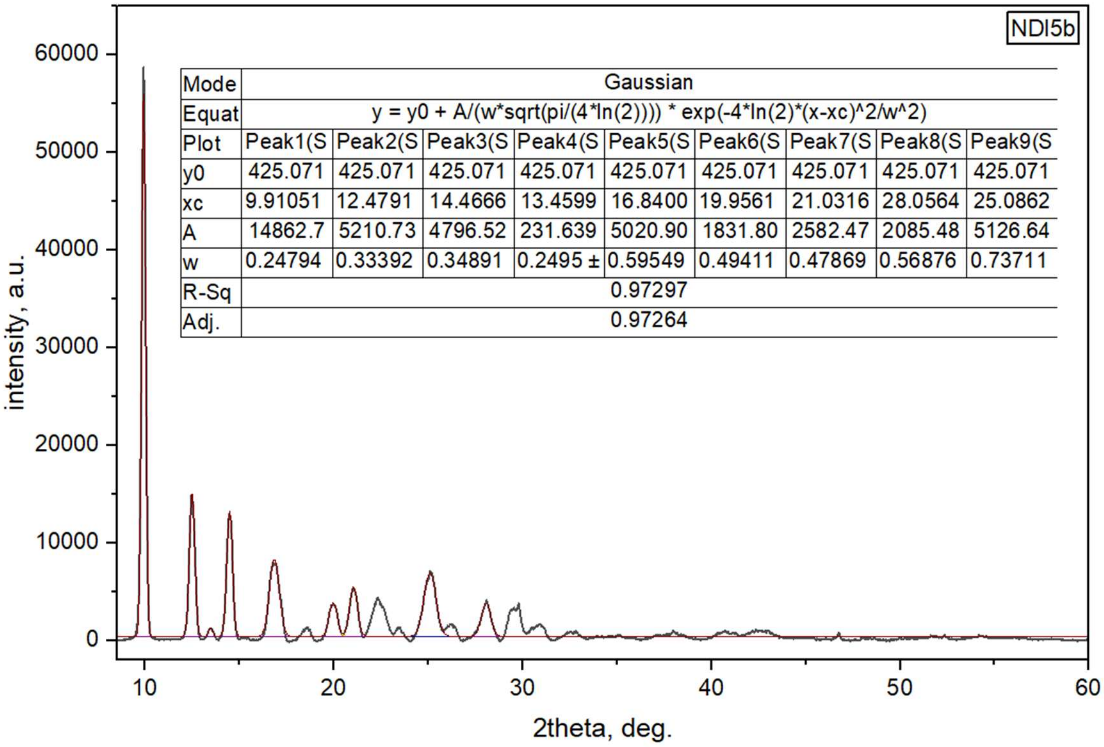

Figure 80. PXRD trace of NDI5b and fwhm calculations.

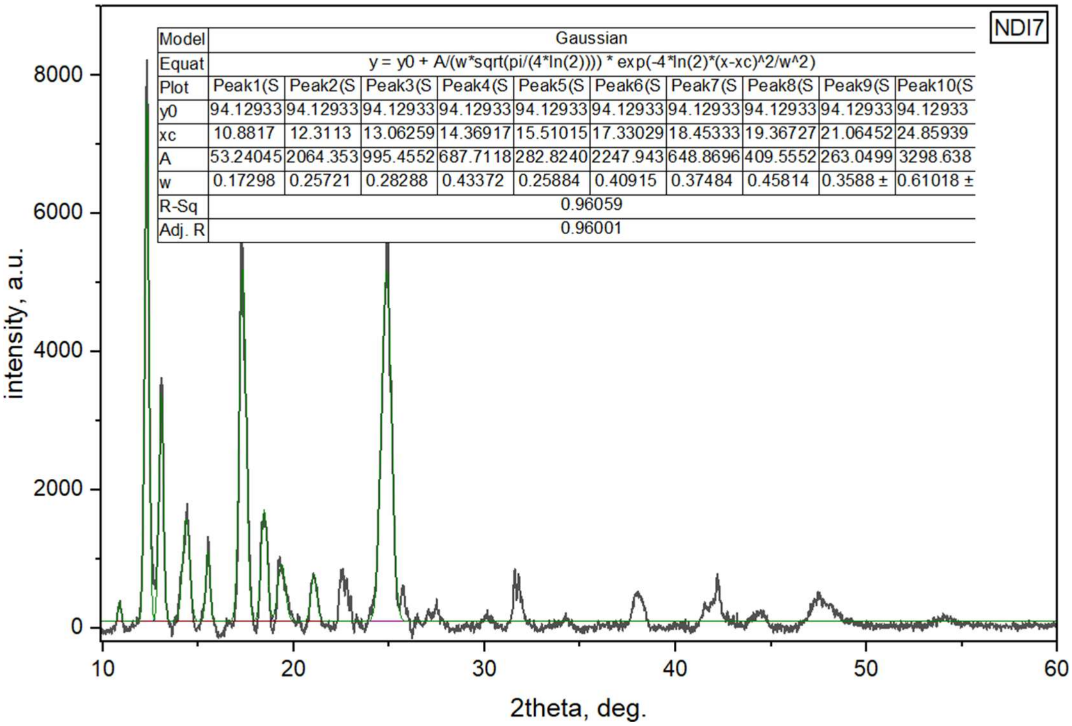

Figure 81. PXRD trace of NDI7 and fwhm calculations.

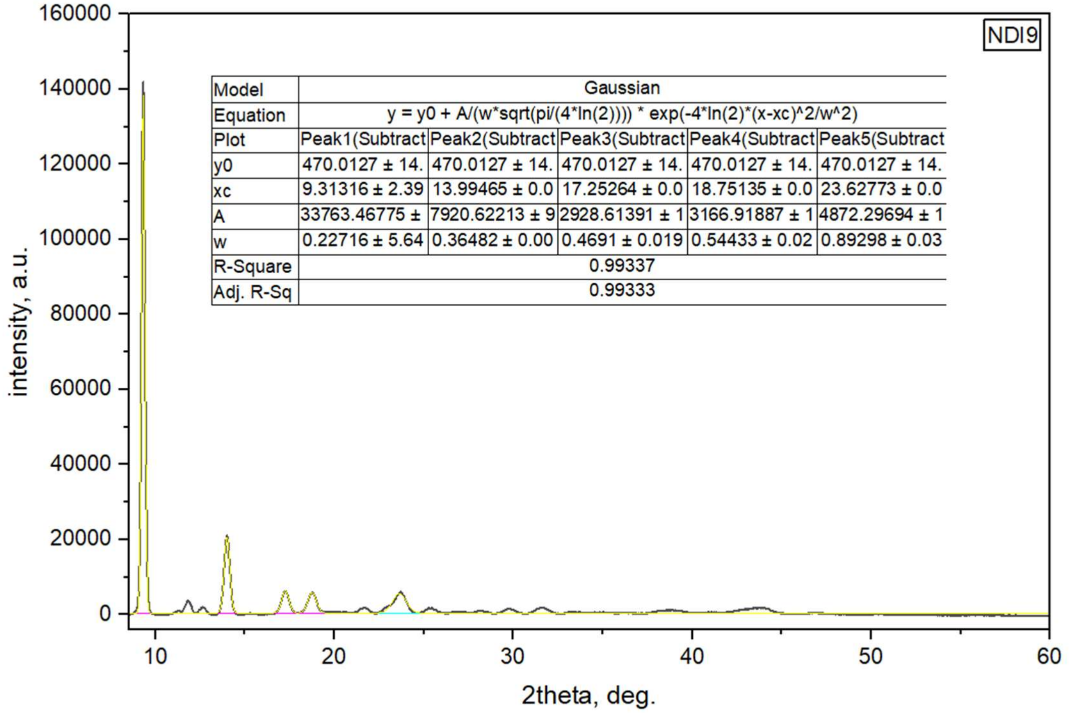

Figure 82. PXRD trace of NDI9 and fwhm calculations.

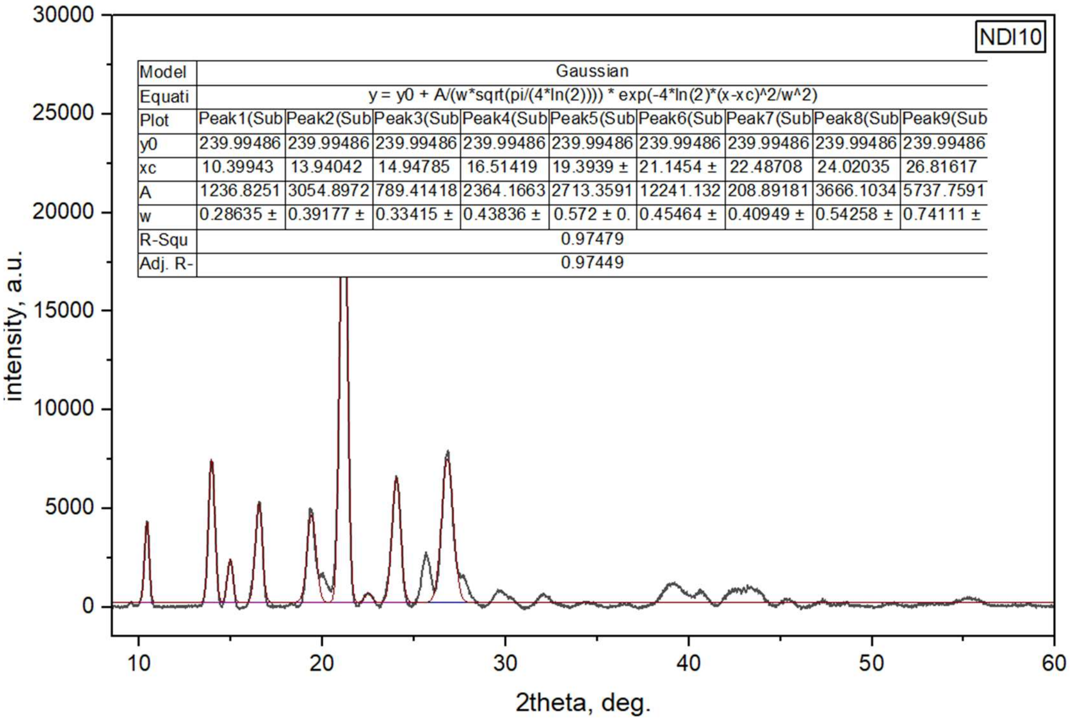

Figure 83. PXRD trace of NDI10 and fwhm calculations.

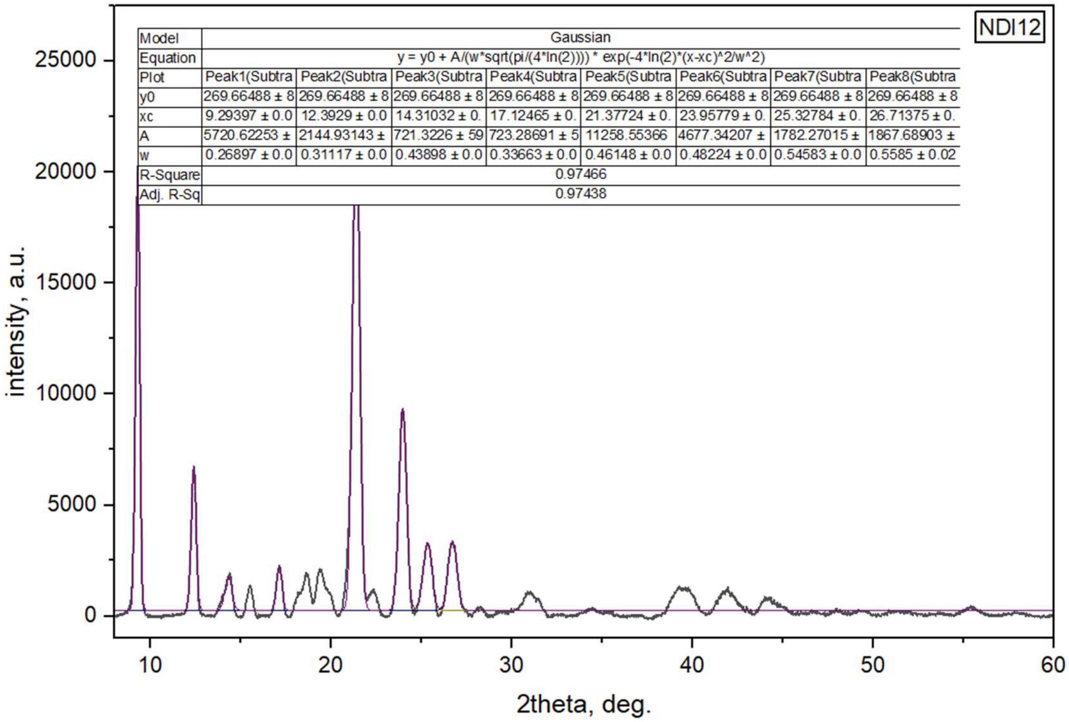

Figure 84. PXRD trace of NDI12 and fwhm calculations.

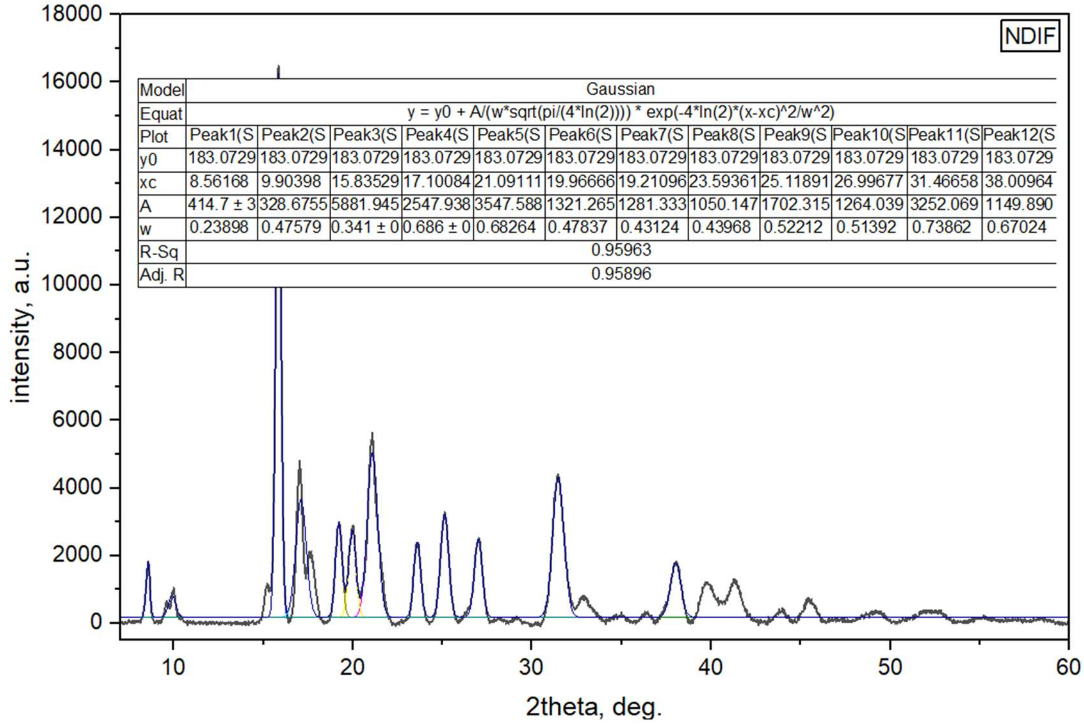

Model	Gaussian											
Equat	y = y0 + A/(w*sqrt(pi/(4*ln(2)))) * exp(-4*ln(2)*(x-xc)^2/w^2)											
Plot	Peak1(S	Peak2(S	Peak3(S	Peak4(S	Peak5(S	Peak6(S	Peak7(S	Peak8(S	Peak9(S	Peak10(S	Peak11(S	Peak12(S
y0	183.0729	183.0729	183.0729	183.0729	183.0729	183.0729	183.0729	183.0729	183.0729	183.0729	183.0729	183.0729
xc	8.56168	9.90398	15.83529	17.10084	21.09111	19.96666	19.21096	23.59361	25.11891	26.99677	31.46658	38.00964
A	414.7 ± 3	328.6755	5881.945	2547.938	3547.588	1321.265	1281.333	1050.147	1702.315	1264.039	3252.069	1149.890
w	0.23898	0.47579	0.341 ± 0	0.686 ± 0	0.68264	0.47837	0.43124	0.43968	0.52212	0.51392	0.73862	0.67024
R-Sq	0.95963											
Adj. R	0.95896											

Figure 85. PXRD trace of NDIF and fwhm calculations.

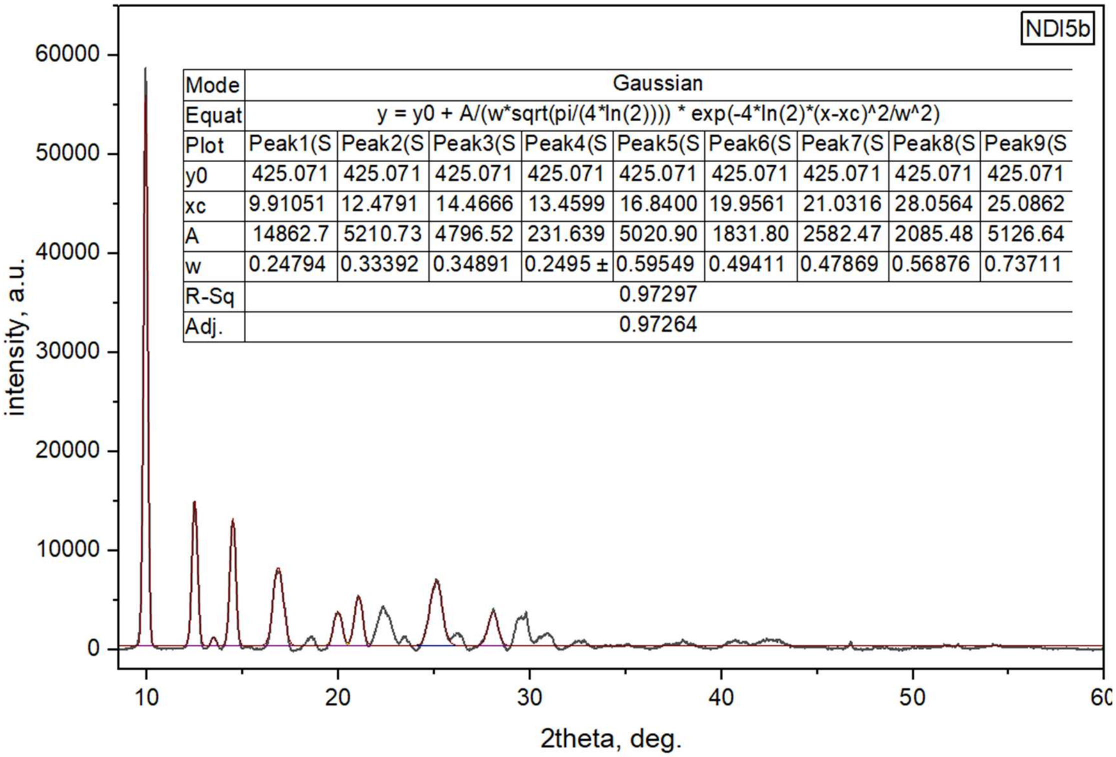

Mode	Gaussian								
Equat	y = y0 + A/(w*sqrt(pi/(4*ln(2)))) * exp(-4*ln(2)*(x-xc)^2/w^2)								
Plot	Peak1(S	Peak2(S	Peak3(S	Peak4(S	Peak5(S	Peak6(S	Peak7(S	Peak8(S	Peak9(S
y0	425.071	425.071	425.071	425.071	425.071	425.071	425.071	425.071	425.071
xc	9.91051	12.4791	14.4666	13.4599	16.8400	19.9561	21.0316	28.0564	25.0862
A	14862.7	5210.73	4796.52	231.639	5020.90	1831.80	2582.47	2085.48	5126.64
w	0.24794	0.33392	0.34891	0.2495 ±	0.59549	0.49411	0.47869	0.56876	0.73711
R-Sq	0.97297								
Adj.	0.97264								

Figure 86. PXRD trace of NDI5b and fwhm calculations.

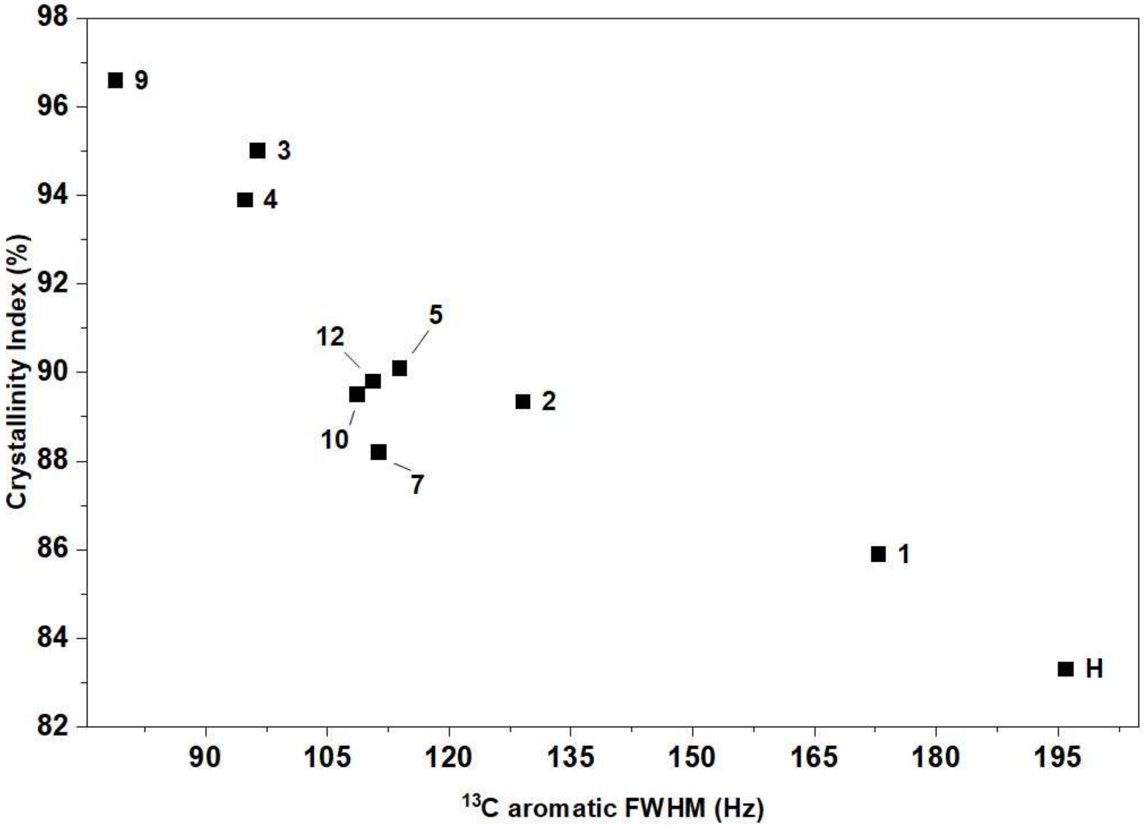

Figure 87. Comparison of ^{13}C ssNMR aromatic FWHM (Hz) vs Crystallinity Index (%).

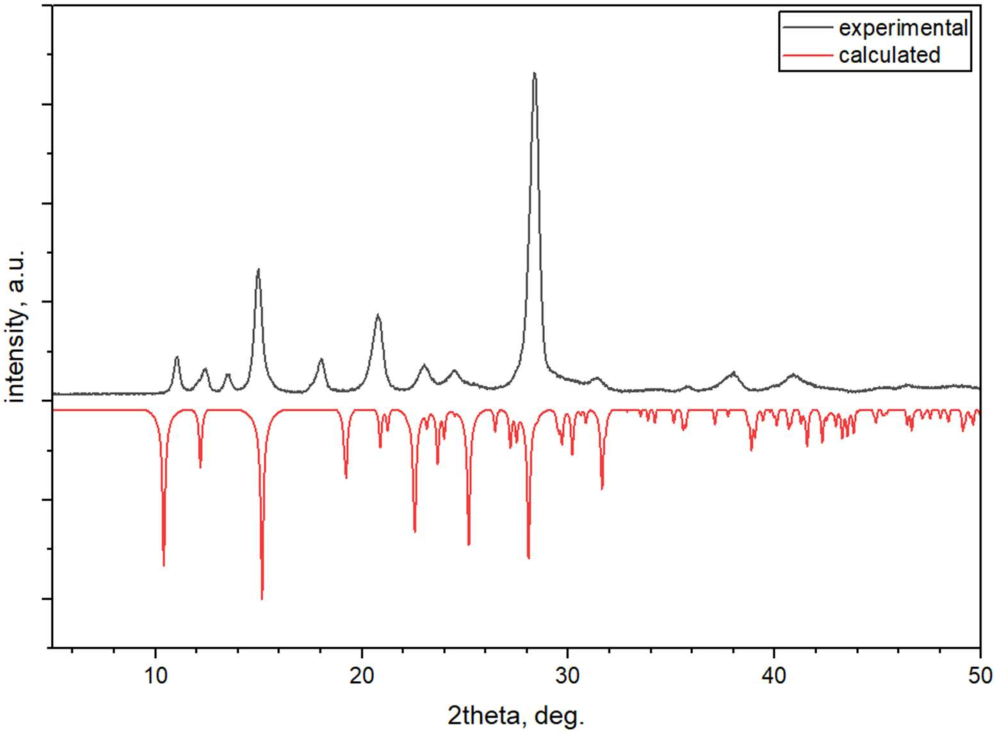

Figure 88. Experimental vs Calculated PXRD traces of NDIH.

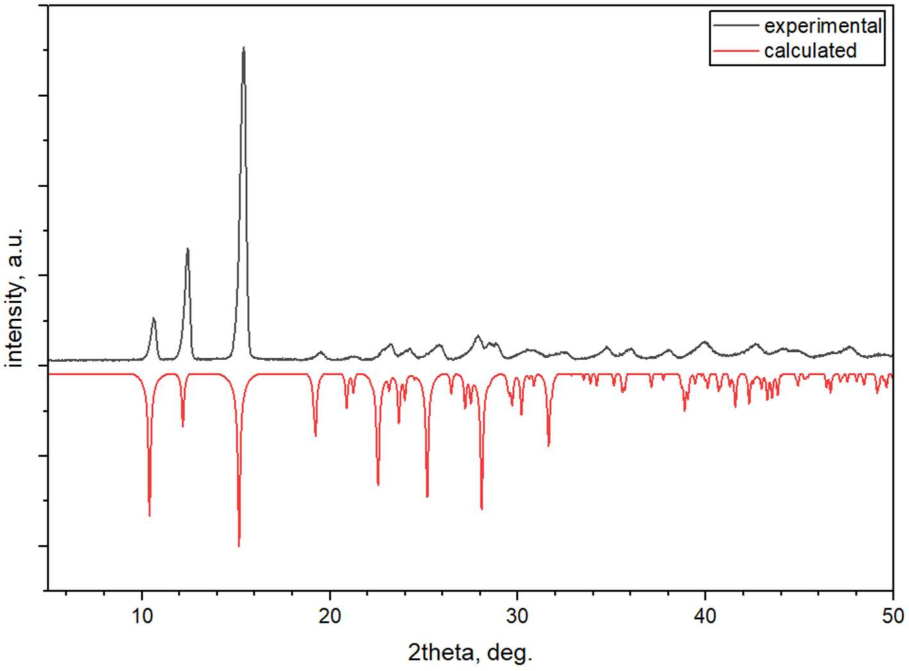

Figure 89. Experimental vs Calculated PXRD traces of NDI1.

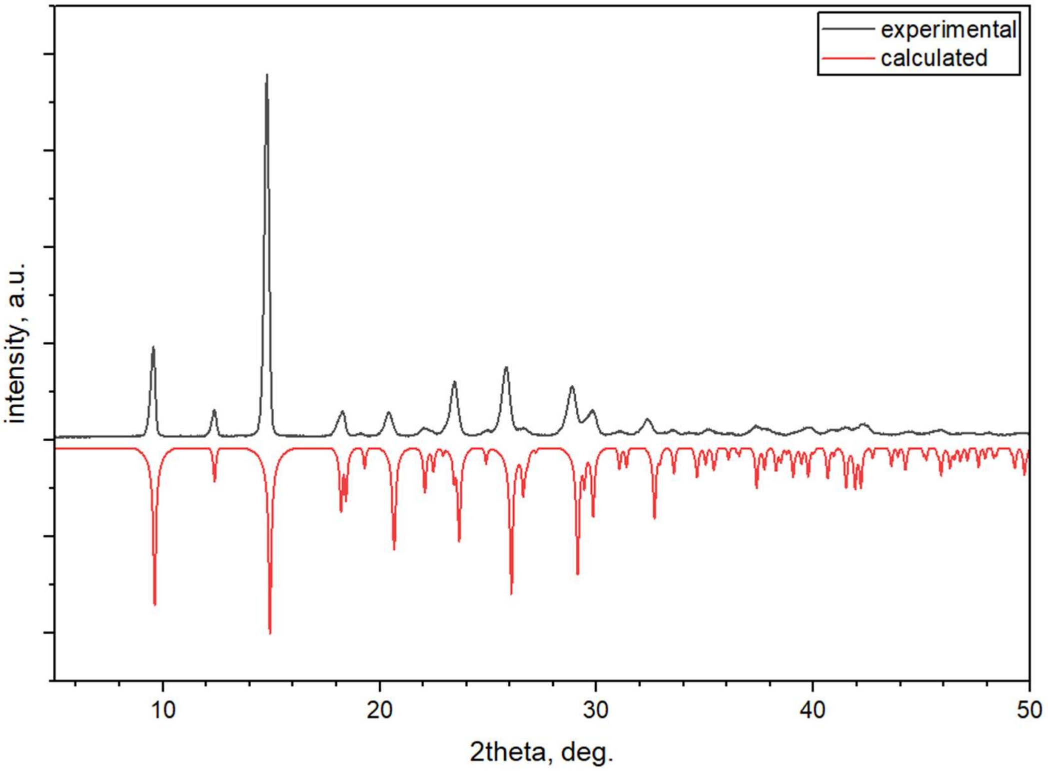

Figure 90. Experimental vs Calculated PXRD traces of NDI2.

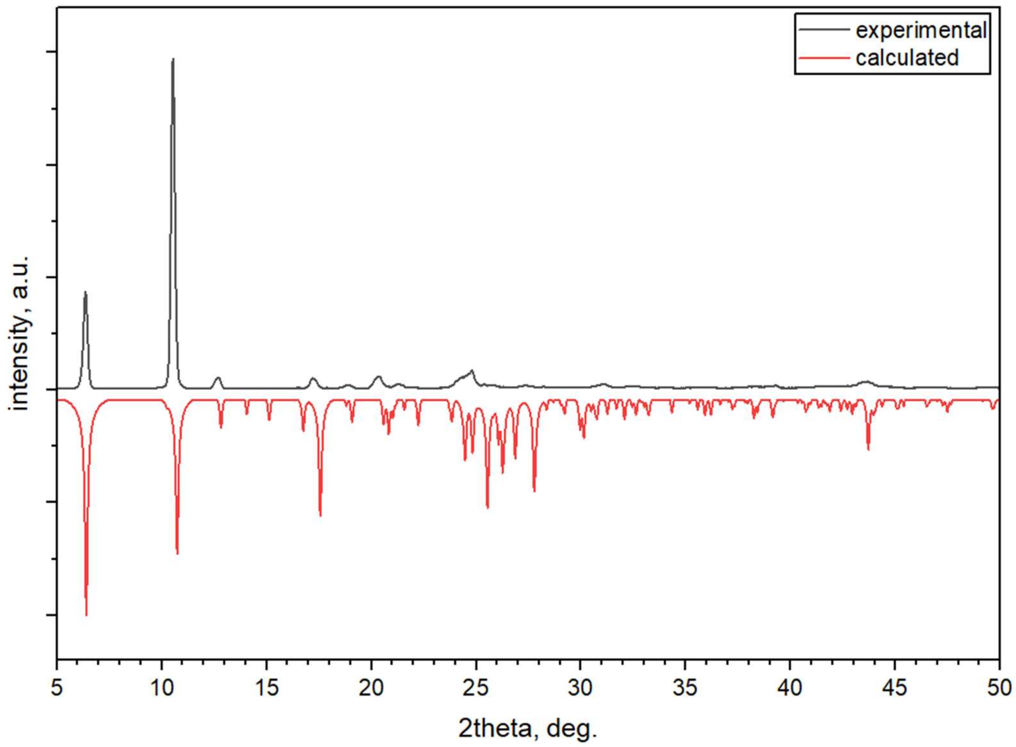

Figure 91. Experimental vs Calculated PXRD traces of NDI3.

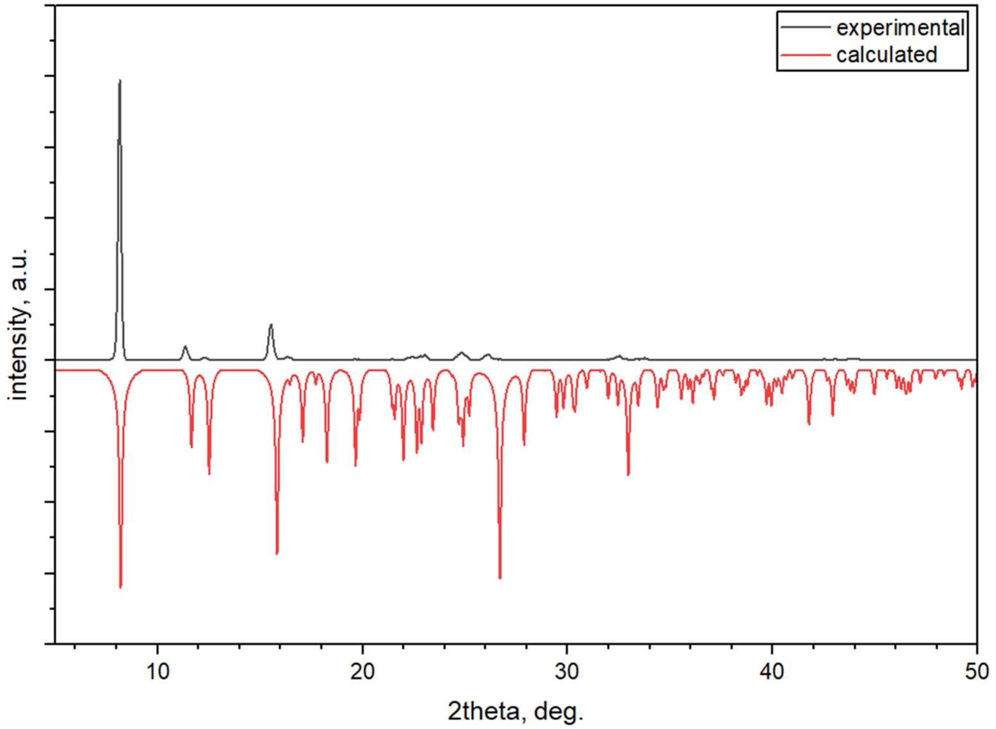

Figure 92. Experimental vs Calculated PXRD traces of NDI4.

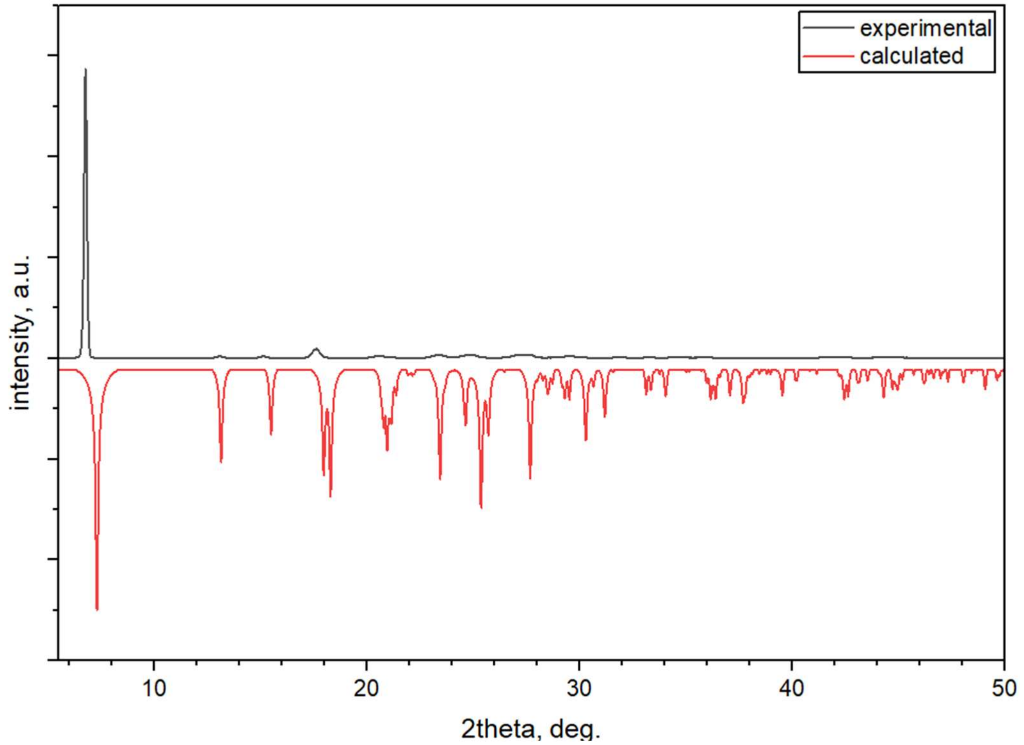

Figure 93. Experimental vs Calculated PXRD traces of NDI5.

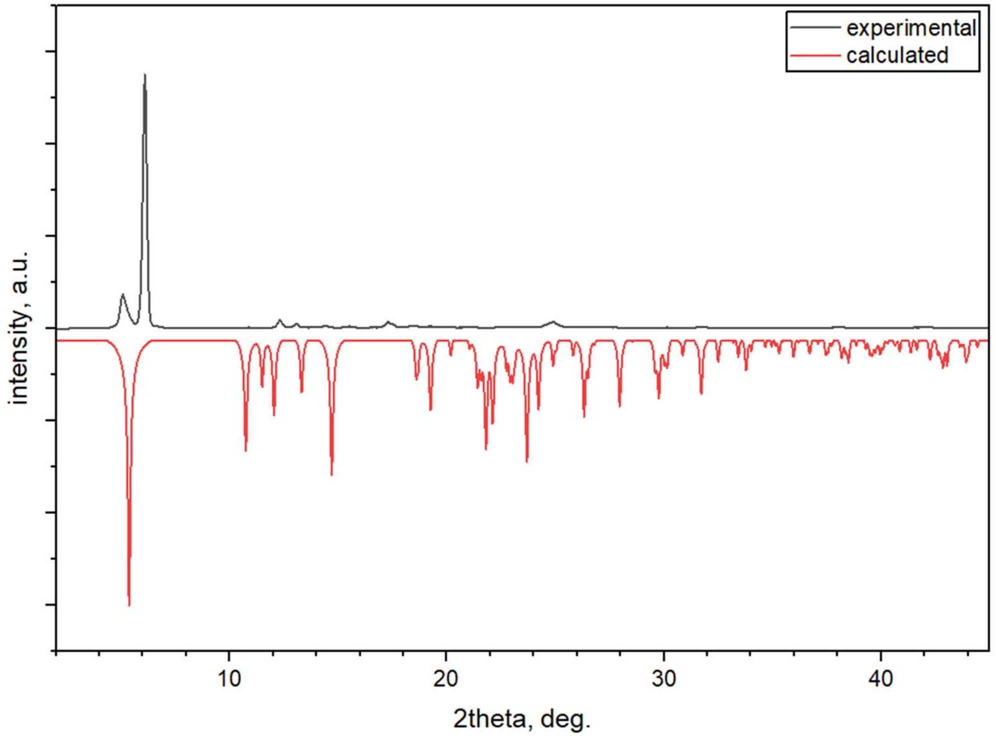

Figure 94. Experimental vs Calculated PXRD traces of NDI7.

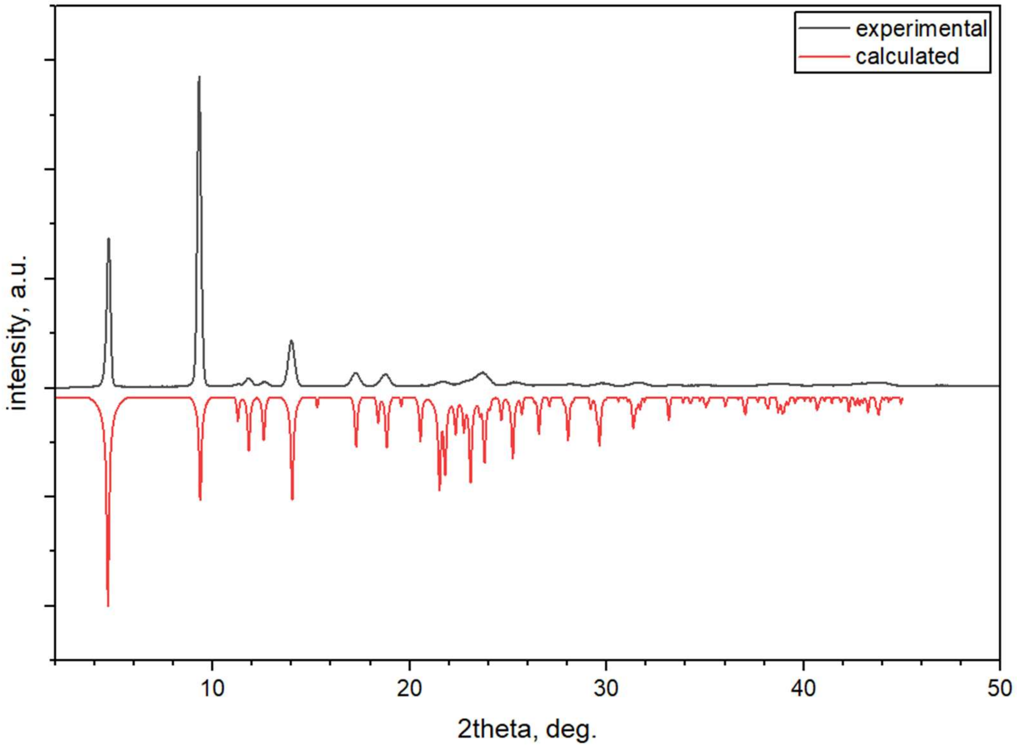

Figure 95. Experimental vs Calculated PXRD traces of NDI9.

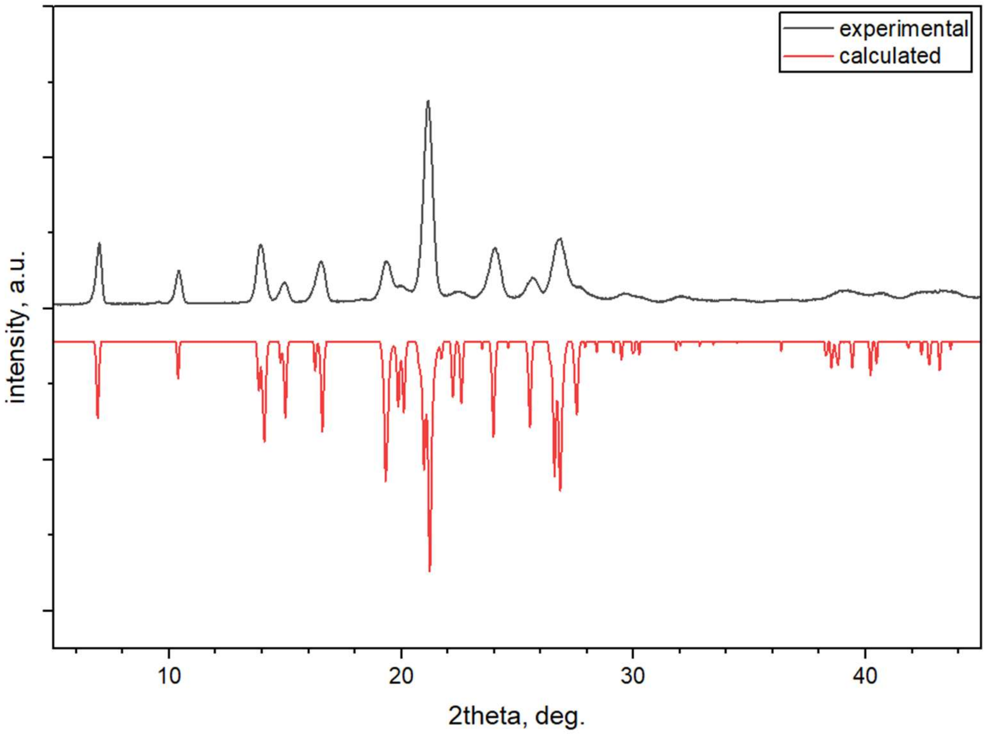

Figure 96. Experimental vs Calculated PXRD traces of NDI10.

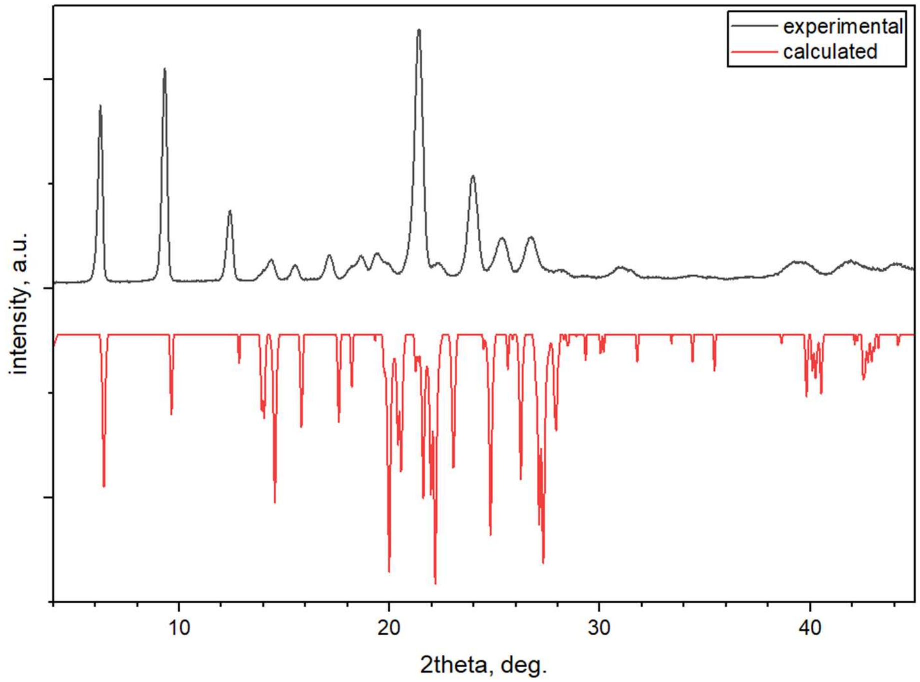

Figure 97. Experimental vs Calculated PXRD traces of NDI12.

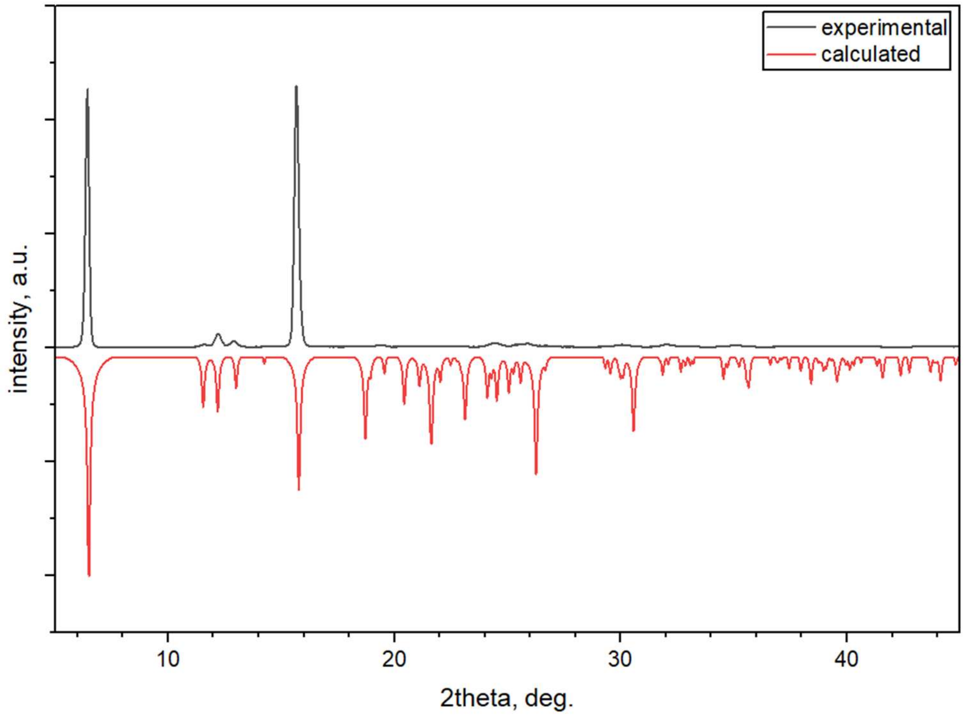

Figure 98. Experimental vs Calculated PXRD traces of NDI5a.

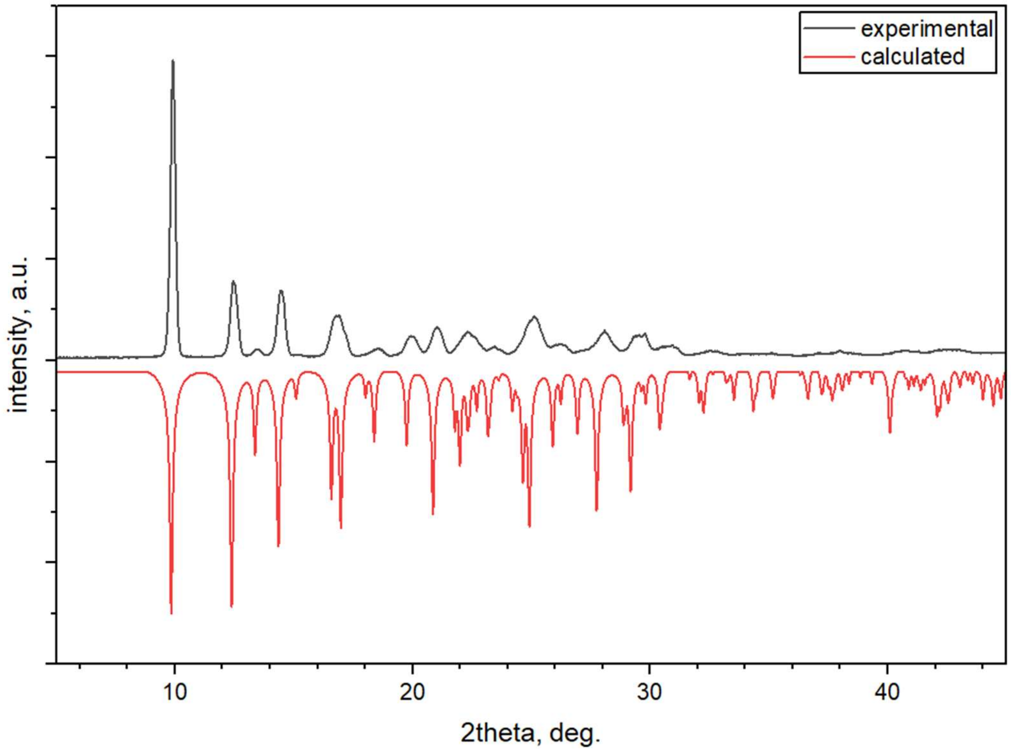

Figure 99. Experimental vs Calculated PXRD traces of NDI5b.

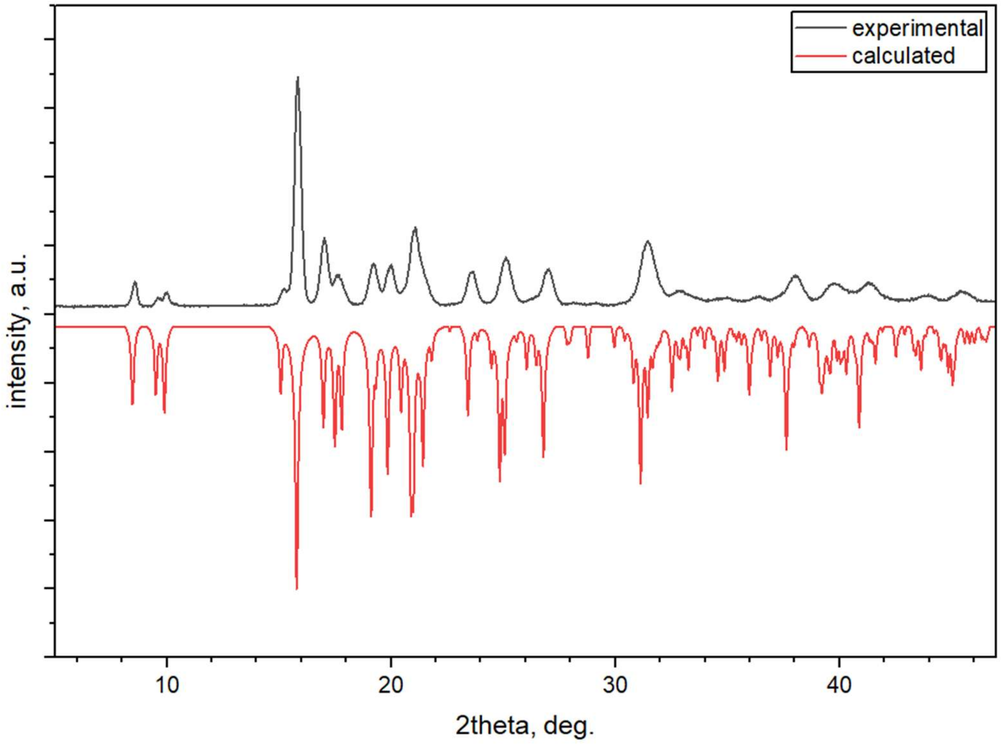

Figure 100. Experimental vs Calculated PXRD traces of NDIF.

Computational Methods

Hirshfeld surface analysis

Hirshfeld surfaces were generated for all NDIs using CrystalExplorer17.5.[26–28] from single-crystal X-ray diffraction .cif files with default settings. Hirshfeld surfaces were mapped with d_{norm} to highlight close intermolecular contacts.

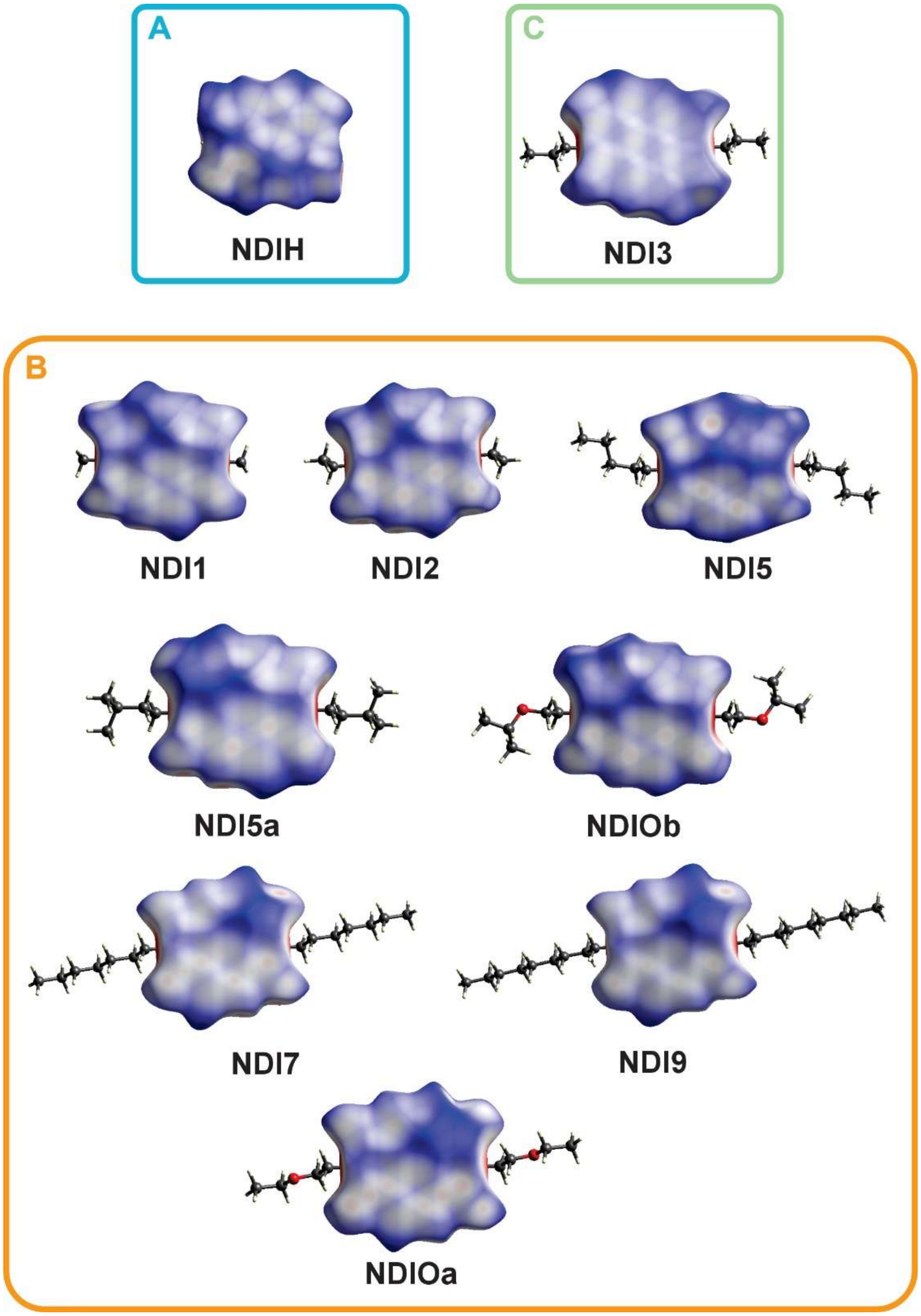

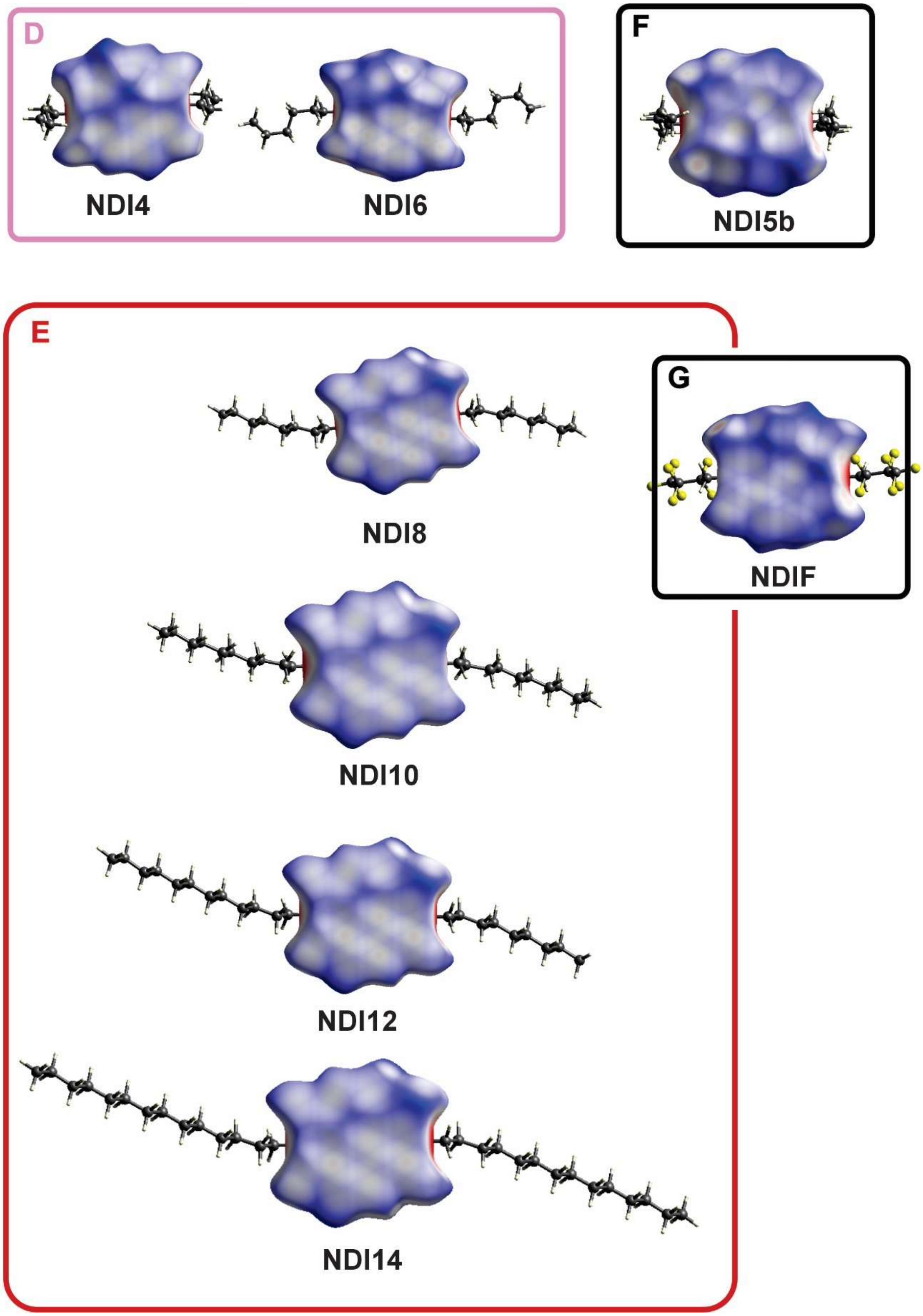

Figure 101. d_{norm} Hirshfeld surfaces for the NDI cores. Red regions indicating distances shorter than the van der Waals separation and blue regions indicating longer distances.

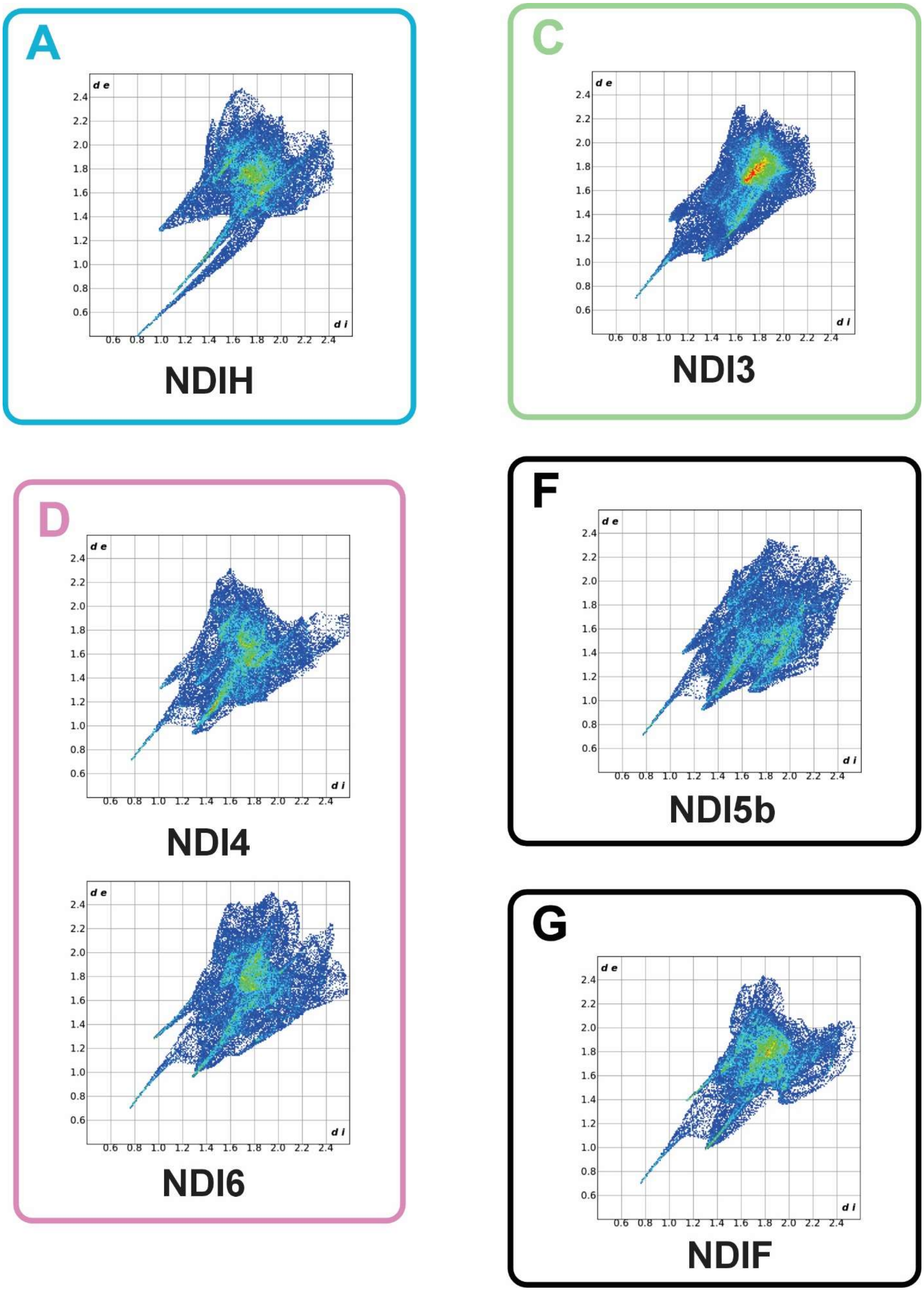
A
d e
d i
NDIH
C
d e
d i
NDI3
D
d e
d i
NDI4
d e
d i
NDI6
F
d e
d i
NDI5b
G
d e
d i
NDIF

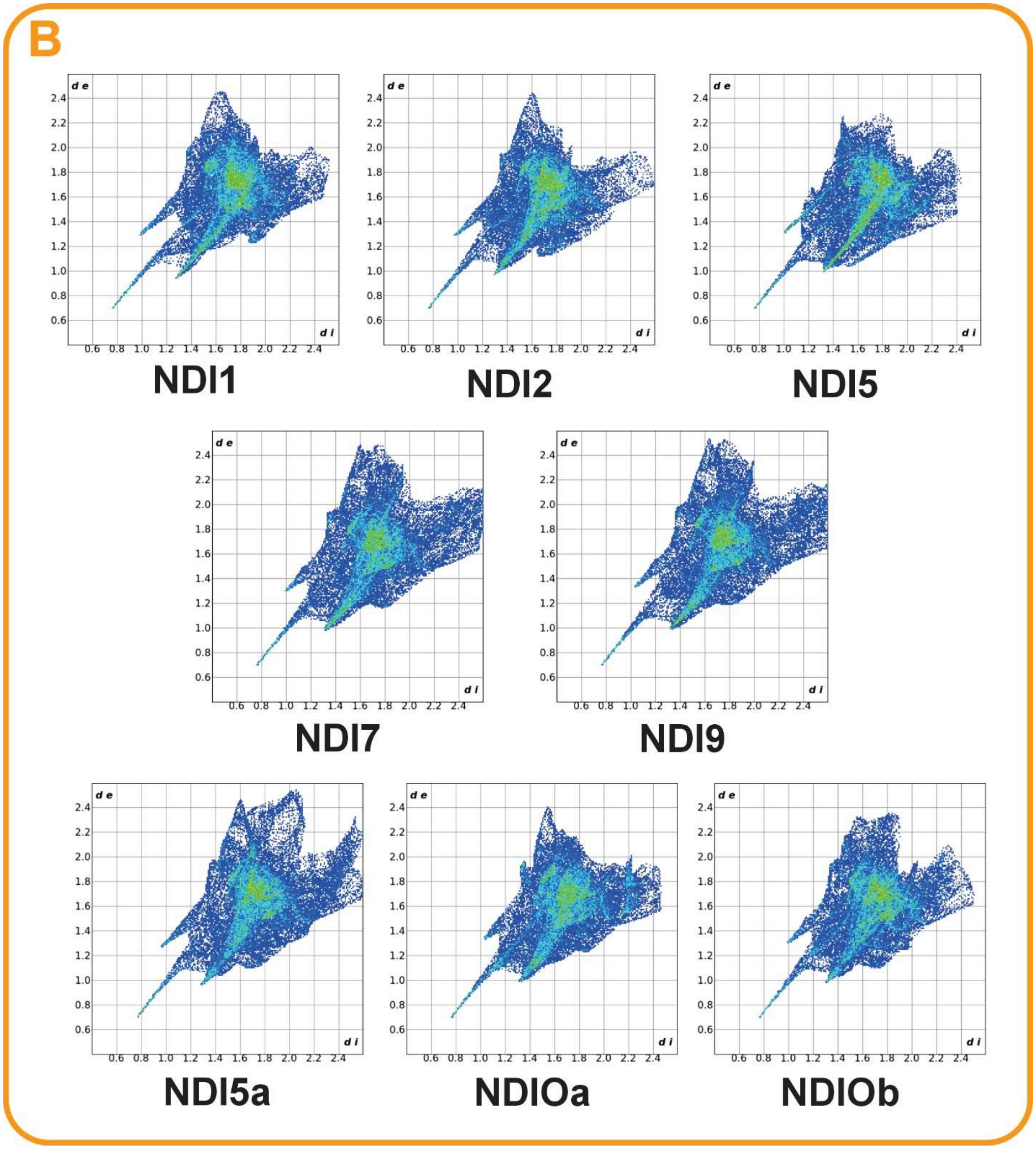
B
NDI1
NDI2
NDI5
NDI7
NDI9
NDI5a
NDIOa
NDIOb
d e
d i

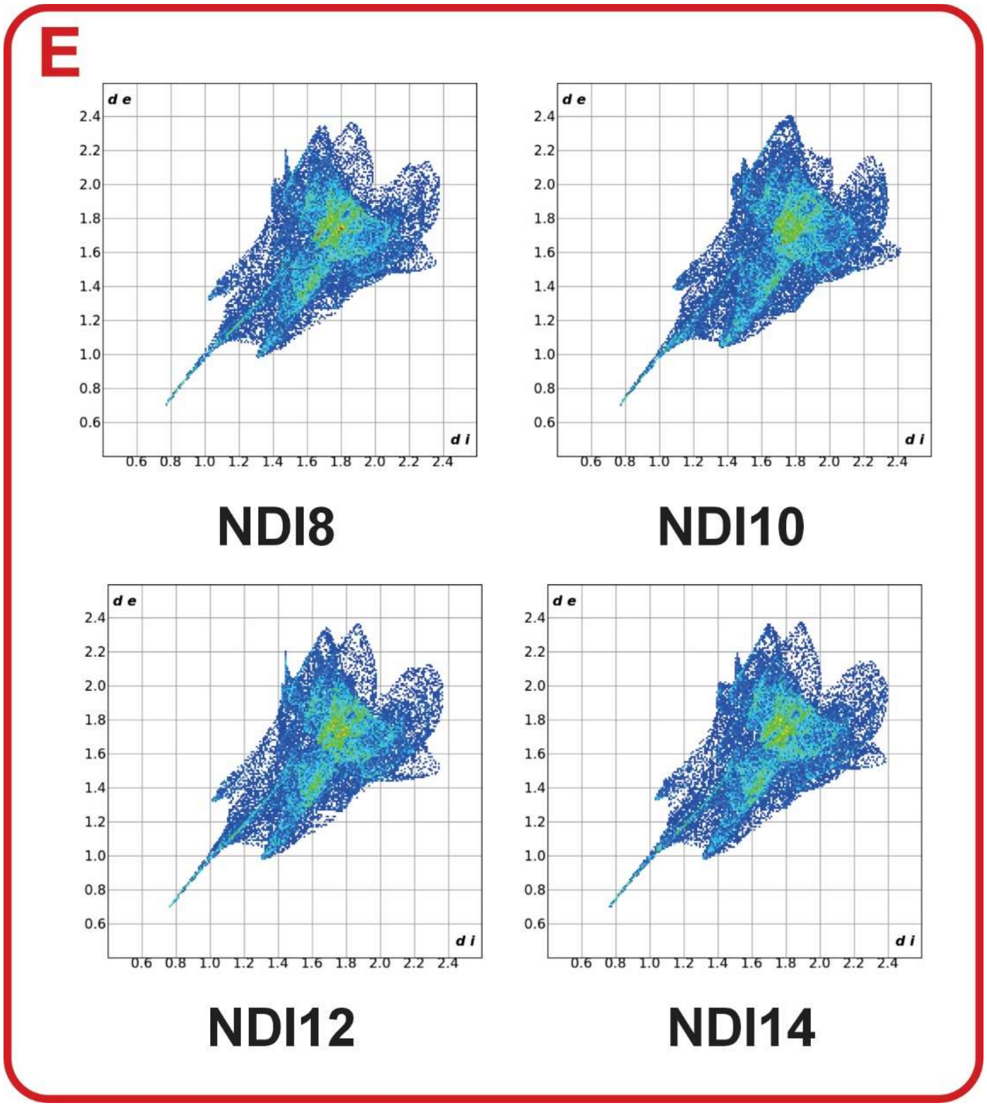

Figure 102. Hirshfeld core-core fingerprint plots for the series of NDIs. For the axes, de is the distance from the Hirshfeld surface to the nearest nucleus outside the surface and di is the distance from the Hirshfeld surface to the nearest nucleus inside the surface. These fingerprint plots show the population of each combination of de and di for a surface and are similar within the packing types identified through ssNMR.

DFT methods

DFT calculations were performed using Gaussian 09[29] and the M06-2X[30] exchange correlation functional with and without Grimme et al.'s D3 empirical dispersion correction.[31] All atoms were represented with the Schäfer et al triple-ζ basis set augmented with polarization functions.[32] Dimers extracted from the X-ray structures of **NDI2** (IIa), **NDI3** (Ib), and **NDI8** (IIb) were used as templates for dimers of other alkyl chain lengths. Each dimer was optimized within the C_2 (Ib) or C_i (IIa and IIb) point groups.

Cartesian coordinates

NDI2 Ib

O	-1.51142600	0.56991500	-4.16899300
O	0.14641200	4.25101000	-2.10441300
O	-2.55371500	-2.45566400	2.09191400
O	-1.18084800	1.34222500	4.15950700
N	-0.75536700	2.44817100	-3.14257000
N	-1.80922000	-0.57962600	3.12925900
C	-1.27435400	1.15478400	-3.13780400
C	-1.52352100	0.53392200	-1.80713200
C	-1.25410000	1.26529300	-0.63212900
C	-0.71612800	2.56737100	-0.68732900
C	-0.39775600	3.17564700	-2.00799800
C	-2.01990800	-0.74553900	-1.73263400
H	-2.22379200	-1.28342200	-2.65061800
C	-2.23306100	-1.34900700	-0.47942000
H	-2.60093100	-2.36530300	-0.40894800
C	-1.96713100	-0.65657300	0.67777000
C	-1.47426400	0.66275400	0.62134100
C	-1.18810300	1.38846700	1.79633200
C	-0.68996700	2.66715700	1.71968700
H	-0.46784600	3.19938100	2.63600800
C	-0.44601700	3.26002400	0.46772900
H	-0.02176800	4.25320700	0.39820200
C	-2.15105100	-1.31917700	1.99654900
C	-1.39167600	0.75031000	3.12547700
C	-0.46802000	3.06075900	-4.45088400
H	-0.64433000	4.12819500	-4.34332900
H	-1.18370400	2.64246800	-5.15317700
C	0.95959700	2.78014900	-4.89239700
H	1.66549600	3.23404400	-4.19523400
H	1.13817600	1.70557500	-4.94656300
C	-1.96713100	-1.23507600	4.43877800
H	-1.21311700	-0.80947100	5.09484800
H	-1.74662400	-2.28879000	4.28919000
C	-3.37009600	-1.03482700	4.98919900

H	-4.11121000	-1.46261100	4.31363500
H	-3.57756900	0.02677000	5.12578200
O	1.18084800	-1.34222500	4.15950700
O	2.55371500	2.45566400	2.09191400
O	-0.14641200	-4.25101000	-2.10441300
O	1.51142600	-0.56991500	-4.16899300
N	1.80922000	0.57962600	3.12925900
N	0.75536700	-2.44817100	-3.14257000
C	1.39167600	-0.75031000	3.12547700
C	1.18810300	-1.38846700	1.79633200
C	1.47426400	-0.66275400	0.62134100
C	1.96713100	0.65657300	0.67777000
C	2.15105100	1.31917700	1.99654900
C	0.68996700	-2.66715700	1.71968700
H	0.46784600	-3.19938100	2.63600800
C	0.44601700	-3.26002400	0.46772900
H	0.02176800	-4.25320700	0.39820200
C	0.71612800	-2.56737100	-0.68732900
C	1.25410000	-1.26529300	-0.63212900
C	1.52352100	-0.53392200	-1.80713200
C	2.01990800	0.74553900	-1.73263400
H	2.22379200	1.28342200	-2.65061800
C	2.23306100	1.34900700	-0.47942000
H	2.60093100	2.36530300	-0.40894800
C	0.39775600	-3.17564700	-2.00799800
C	1.27435400	-1.15478400	-3.13780400
C	1.96713100	1.23507600	4.43877800
H	1.74662400	2.28879000	4.28919000
H	1.21311700	0.80947100	5.09484800
C	3.37009600	1.03482700	4.98919900
H	4.11121000	1.46261100	4.31363500
H	3.57756900	-0.02677000	5.12578200
C	0.46802000	-3.06075900	-4.45088400
H	1.18370400	-2.64246800	-5.15317700
H	0.64433000	-4.12819500	-4.34332900
C	-0.95959700	-2.78014900	-4.89239700
H	-1.66549600	-3.23404400	-4.19523400
H	-1.13817600	-1.70557500	-4.94656300
H	-3.46294400	-1.52869900	5.95657600
H	-1.13375200	-3.21232200	-5.87812800
H	1.13375200	3.21232200	-5.87812800
H	3.46294400	1.52869900	5.95657600

NDI2 IIa

C	1.26430100	-1.57379000	-1.56728700
C	-0.14337600	-1.60942600	-1.57704800

C	1.98651900	-2.78245500	-1.52796200
C	1.92583300	-0.32899900	-1.58189900
C	-0.86873300	-0.39871100	-1.57493400
C	-0.80103200	-2.85464100	-1.54900200
C	1.32675700	-3.98706000	-1.50095300
C	1.20332300	0.83799900	-1.61235300
C	-0.20607100	0.80339900	-1.60154900
C	-0.07871600	-4.02348500	-1.51725200
C	3.47426900	-2.75351300	-1.51732800
C	3.41344000	-0.28293600	-1.51551100
C	-2.35486600	-0.43298500	-1.48324100
C	-2.28679400	-2.90456300	-1.58155700
H	1.90791800	-4.89956100	-1.47102700
H	1.73511500	1.78192900	-1.62072000
H	-0.78288800	1.72031300	-1.59391800
H	-0.61175100	-4.96543700	-1.50563800
O	4.13399400	-3.76561500	-1.49599200
O	4.01048900	0.76824200	-1.44974200
O	-3.01309900	0.58249500	-1.39288700
O	-2.89125900	-3.94664100	-1.66434400
N	4.08491700	-1.50086200	-1.54570700
N	-2.96101100	-1.68300900	-1.49671700
C	5.55457500	-1.48387400	-1.46258900
C	-4.43333600	-1.72842300	-1.49774000
C	6.03908500	-1.74583000	-0.04373000
H	5.92055200	-2.25006800	-2.14305900
H	5.87215900	-0.50709700	-1.81558900
C	-4.98586600	-1.58076000	-2.90613400
H	-4.71526100	-2.68038300	-1.05798600
H	-4.77391000	-0.93291200	-0.84444800
H	5.58873300	-1.03996800	0.65362500
H	5.77396900	-2.75421900	0.27028000
H	-4.68813300	-0.62416800	-3.33685200
H	-4.62896800	-2.38886500	-3.54530800
C	0.14337600	1.60942600	1.57704800
C	-1.26430100	1.57379000	1.56728700
C	0.86873300	0.39871100	1.57493400
C	0.80103200	2.85464100	1.54900200
C	-1.98651900	2.78245500	1.52796200
C	-1.92583300	0.32899900	1.58189900
C	0.20607100	-0.80339900	1.60154900
C	0.07871600	4.02348500	1.51725200
C	-1.32675700	3.98706000	1.50095300
C	-1.20332300	-0.83799900	1.61235300
C	2.35486600	0.43298500	1.48324100
C	2.28679400	2.90456300	1.58155700

C	-3.47426900	2.75351300	1.51732800
C	-3.41344000	0.28293600	1.51551100
H	0.78288800	-1.72031300	1.59391800
H	0.61175100	4.96543700	1.50563800
H	-1.90791800	4.89956100	1.47102700
H	-1.73511500	-1.78192900	1.62072000
O	3.01309900	-0.58249500	1.39288700
O	2.89125900	3.94664100	1.66434400
O	-4.13399400	3.76561500	1.49599200
O	-4.01048900	-0.76824200	1.44974200
N	2.96101100	1.68300900	1.49671700
N	-4.08491700	1.50086200	1.54570700
C	4.43333600	1.72842300	1.49774000
C	-5.55457500	1.48387400	1.46258900
C	4.98586600	1.58076000	2.90613400
H	4.77391000	0.93291200	0.84444800
H	4.71526100	2.68038300	1.05798600
C	-6.03908500	1.74583000	0.04373000
H	-5.87215900	0.50709700	1.81558900
H	-5.92055200	2.25006800	2.14305900
H	4.62896800	2.38886500	3.54530800
H	4.68813300	0.62416800	3.33685200
H	-5.77396900	2.75421900	-0.27028000
H	-5.58873300	1.03996800	-0.65362500
H	-7.12376700	1.64339600	0.00045900
H	-6.07526500	-1.61997500	-2.88248300
H	7.12376700	-1.64339600	-0.00045900
H	6.07526500	1.61997500	2.88248300

NDI2 IIb

O	-2.04706300	-0.14526700	-4.13865800
C	-1.37746300	0.70663700	-3.59911900
N	-0.66271400	1.63853000	-4.34255000
C	0.27460700	2.53194100	-3.81780300
O	0.96194000	3.21002500	-4.54226700
C	0.33807400	2.64129000	-2.33464500
C	-0.48709800	1.82343200	-1.53705800
C	-1.31096000	0.83574800	-2.11516200
C	-2.05345800	-0.00183200	-1.31880300
C	-1.98868300	0.11157800	0.08349600
C	-0.82825800	1.62193000	-5.80459300
C	0.09292400	0.62537700	-6.49018500
H	-2.67467800	-0.75576500	-1.78764500
H	-2.54785100	-0.56286500	0.72108800
H	-0.62031600	2.63324300	-6.14382600
H	-1.87145300	1.38242900	-5.99902300

H	1.12079100	0.75888600	-6.15115700
H	-0.22810000	-0.39453600	-6.28493700
O	1.12840200	3.91188300	2.45948000
C	0.46342200	3.05975800	1.91987500
N	-0.29560500	2.16069600	2.67198800
C	-1.08783000	1.14357300	2.14793100
O	-1.65567100	0.35123700	2.86923200
C	-1.19509600	1.06947000	0.66574800
C	-0.43332600	1.94508300	-0.13542600
C	0.41844100	2.90773500	0.44170800
C	1.21002500	3.70070500	-0.35322400
C	1.17359900	3.56264500	-1.75334000
C	-0.25735900	2.32151500	4.13432300
C	-1.22319300	3.40207700	4.59427700
H	1.85655900	4.43022200	0.11756400
H	1.79582600	4.17794000	-2.39018500
H	-0.52517300	1.35744300	4.55292100
H	0.76920900	2.55350400	4.40693400
H	-2.24525900	3.14682600	4.31128500
H	-0.95942600	4.36547400	4.15886500
O	-1.12840200	-3.91188300	-2.45948000
C	-0.46342200	-3.05975800	-1.91987500
N	0.29560500	-2.16069600	-2.67198800
C	1.08783000	-1.14357300	-2.14793100
O	1.65567100	-0.35123700	-2.86923200
C	1.19509600	-1.06947000	-0.66574800
C	0.43332600	-1.94508300	0.13542600
C	-0.41844100	-2.90773500	-0.44170800
C	-1.21002500	-3.70070500	0.35322400
C	-1.17359900	-3.56264500	1.75334000
C	0.25735900	-2.32151500	-4.13432300
C	1.22319300	-3.40207700	-4.59427700
H	-1.85655900	-4.43022200	-0.11756400
H	-1.79582600	-4.17794000	2.39018500
H	0.52517300	-1.35744300	-4.55292100
H	-0.76920900	-2.55350400	-4.40693400
H	2.24525900	-3.14682600	-4.31128500
H	0.95942600	-4.36547400	-4.15886500
O	2.04706300	0.14526700	4.13865800
C	1.37746300	-0.70663700	3.59911900
N	0.66271400	-1.63853000	4.34255000
C	-0.27460700	-2.53194100	3.81780300
O	-0.96194000	-3.21002500	4.54226700
C	-0.33807400	-2.64129000	2.33464500
C	0.48709800	-1.82343200	1.53705800
C	1.31096000	-0.83574800	2.11516200

C	2.05345800	0.00183200	1.31880300
C	1.98868300	-0.11157800	-0.08349600
C	0.82825800	-1.62193000	5.80459300
C	-0.09292400	-0.62537700	6.49018500
H	2.67467800	0.75576500	1.78764500
H	2.54785100	0.56286500	-0.72108800
H	0.62031600	-2.63324300	6.14382600
H	1.87145300	-1.38242900	5.99902300
H	-1.12079100	-0.75888600	6.15115700
H	0.22810000	0.39453600	6.28493700
H	0.06312900	0.77993000	-7.56911500
H	1.18331500	-3.49450800	-5.68002700
H	-0.06312900	-0.77993000	7.56911500
H	-1.18331500	3.49450800	5.68002700

NDI3 Ib

O	-0.05403300	1.61700400	-4.17242600
O	3.97173900	1.54693200	-2.08542300
O	-3.27971400	1.37803400	2.07099700
O	0.74173800	1.61266800	4.16036000
N	1.96426400	1.66475800	-3.13466800
N	-1.26775100	1.43585200	3.11987800
C	0.57084400	1.62860200	-3.13751600
C	-0.10496100	1.61128600	-1.81058200
C	0.66702200	1.65190500	-0.63154000
C	2.07610400	1.67203900	-0.67900300
C	2.76824400	1.62103800	-1.99590500
C	-1.47684900	1.56096300	-1.74373400
H	-2.04621300	1.53481000	-2.66515800
C	-2.12234700	1.51875700	-0.49403500
H	-3.20178100	1.45488800	-0.42945700
C	-1.38742500	1.54847400	0.66729500
C	0.01953900	1.61700000	0.61849600
C	0.79295100	1.63894100	1.79767900
C	2.16478400	1.68570000	1.72864100
H	2.73607100	1.69072100	2.64848600
C	2.81275100	1.69588700	0.48009500
H	3.89321500	1.69734900	0.41649300
C	-2.07610400	1.45711300	1.98242900
C	0.11923600	1.57470900	3.12334400
C	2.64369000	1.64902200	-4.43906200
H	3.56831100	2.21177800	-4.32480600
H	1.99062800	2.16158000	-5.14214000
C	2.92933600	0.22962100	-4.91114900
H	3.59056000	-0.25123700	-4.18516600
H	1.99099800	-0.32929500	-4.93722600

C	3.58434900	0.23692700	-6.28722600
H	3.79526900	-0.77721400	-6.62544500
H	4.52665500	0.78843000	-6.27113200
H	2.93094800	0.70596600	-7.02539700
C	-1.93782200	1.31923300	4.42417500
H	-1.25413800	0.79425000	5.08738100
H	-2.82211900	0.70330800	4.27314100
C	-2.31564800	2.68285700	4.98700000
H	-2.97115000	3.18997800	4.27522300
H	-1.40884900	3.28301900	5.09170300
C	-3.01170000	2.53998800	6.33513800
H	-3.28133200	3.51375000	6.74342600
H	-2.36293000	2.04068600	7.05726200
H	-3.92603500	1.95103600	6.24105200
O	-0.74173800	-1.61266800	4.16036000
O	3.27971400	-1.37803400	2.07099700
O	-3.97173900	-1.54693200	-2.08542300
O	0.05403300	-1.61700400	-4.17242600
N	1.26775100	-1.43585200	3.11987800
N	-1.96426400	-1.66475800	-3.13466800
C	-0.11923600	-1.57470900	3.12334400
C	-0.79295100	-1.63894100	1.79767900
C	-0.01953900	-1.61700000	0.61849600
C	1.38742500	-1.54847400	0.66729500
C	2.07610400	-1.45711300	1.98242900
C	-2.16478400	-1.68570000	1.72864100
H	-2.73607100	-1.69072100	2.64848600
C	-2.81275100	-1.69588700	0.48009500
H	-3.89321500	-1.69734900	0.41649300
C	-2.07610400	-1.67203900	-0.67900300
C	-0.66702200	-1.65190500	-0.63154000
C	0.10496100	-1.61128600	-1.81058200
C	1.47684900	-1.56096300	-1.74373400
H	2.04621300	-1.53481000	-2.66515800
C	2.12234700	-1.51875700	-0.49403500
H	3.20178100	-1.45488800	-0.42945700
C	-2.76824400	-1.62103800	-1.99590500
C	-0.57084400	-1.62860200	-3.13751600
C	1.93782200	-1.31923300	4.42417500
H	2.82211900	-0.70330800	4.27314100
H	1.25413800	-0.79425000	5.08738100
C	2.31564800	-2.68285700	4.98700000
H	2.97115000	-3.18997800	4.27522300
H	1.40884900	-3.28301900	5.09170300
C	3.01170000	-2.53998800	6.33513800
H	3.28133200	-3.51375000	6.74342600

H	3.92603500	-1.95103600	6.24105200
H	2.36293000	-2.04068600	7.05726200
C	-2.64369000	-1.64902200	-4.43906200
H	-1.99062800	-2.16158000	-5.14214000
H	-3.56831100	-2.21177800	-4.32480600
C	-2.92933600	-0.22962100	-4.91114900
H	-3.59056000	0.25123700	-4.18516600
H	-1.99099800	0.32929500	-4.93722600
C	-3.58434900	-0.23692700	-6.28722600
H	-3.79526900	0.77721400	-6.62544500
H	-2.93094800	-0.70596600	-7.02539700
H	-4.52665500	-0.78843000	-6.27113200

NDI3 IIa

C	1.26038300	-1.58202800	-1.56692200
C	-0.14716500	-1.62242200	-1.58148500
C	1.98690100	-2.78803300	-1.52340100
C	1.91734100	-0.33514100	-1.58850400
C	-0.87606500	-0.41414500	-1.57641200
C	-0.80145300	-2.86947900	-1.55583400
C	1.33046600	-3.99454100	-1.49773700
C	1.19109800	0.82955800	-1.62028000
C	-0.21787200	0.79041000	-1.60531600
C	-0.07498200	-4.03573200	-1.52227200
C	3.47467600	-2.75358500	-1.49498200
C	3.40441400	-0.28336200	-1.52434900
C	-2.36099200	-0.45369500	-1.47726400
C	-2.28749900	-2.92423400	-1.59191600
H	1.91416900	-4.90522500	-1.46215400
H	1.72003300	1.77500700	-1.63342300
H	-0.79789900	1.70524400	-1.59570900
H	-0.60456200	-4.97965400	-1.51330000
O	4.13721100	-3.76309700	-1.45192900
O	3.99768300	0.77096400	-1.47601900
O	-3.02106000	0.55931500	-1.37240300
O	-2.88704000	-3.96831400	-1.68162700
N	4.08087200	-1.49876000	-1.53320600
N	-2.96536800	-1.70439800	-1.49860400
C	5.54727900	-1.47119600	-1.43090600
C	-4.43630100	-1.74572800	-1.47865600
C	6.02542600	-1.67891100	0.00207900
H	5.93192300	-2.25896900	-2.07731700
H	5.87085600	-0.50463800	-1.81009100
C	-5.03185500	-1.48112900	-2.85432600
H	-4.71795900	-2.73100100	-1.11625400
H	-4.76963300	-1.00640900	-0.75598400

H	5.51986100	-0.96374900	0.65426300
H	5.72612900	-2.67440400	0.33344600
H	-4.67683100	-0.51338600	-3.21584400
H	-4.67888500	-2.24681500	-3.54949000
C	0.14716500	1.62242200	1.58148500
C	-1.26038300	1.58202800	1.56692200
C	0.87606500	0.41414500	1.57641200
C	0.80145300	2.86947900	1.55583400
C	-1.98690100	2.78803300	1.52340100
C	-1.91734100	0.33514100	1.58850400
C	0.21787200	-0.79041000	1.60531600
C	0.07498200	4.03573200	1.52227200
C	-1.33046600	3.99454100	1.49773700
C	-1.19109800	-0.82955800	1.62028000
C	2.36099200	0.45369500	1.47726400
C	2.28749900	2.92423400	1.59191600
C	-3.47467600	2.75358500	1.49498200
C	-3.40441400	0.28336200	1.52434900
H	0.79789900	-1.70524400	1.59570900
H	0.60456200	4.97965400	1.51330000
H	-1.91416900	4.90522500	1.46215400
H	-1.72003300	-1.77500700	1.63342300
O	3.02106000	-0.55931500	1.37240300
O	2.88704000	3.96831400	1.68162700
O	-4.13721100	3.76309700	1.45192900
O	-3.99768300	-0.77096400	1.47601900
N	2.96536800	1.70439800	1.49860400
N	-4.08087200	1.49876000	1.53320600
C	4.43630100	1.74572800	1.47865600
C	-5.54727900	1.47119600	1.43090600
C	5.03185500	1.48112900	2.85432600
H	4.76963300	1.00640900	0.75598400
H	4.71795900	2.73100100	1.11625400
C	-6.02542600	1.67891100	-0.00207900
H	-5.87085600	0.50463800	1.81009100
H	-5.93192300	2.25896900	2.07731700
H	4.67888500	2.24681500	3.54949000
H	4.67683100	0.51338600	3.21584400
H	-5.72612900	2.67440400	-0.33344600
H	-5.51986100	0.96374900	-0.65426300
C	6.55460600	1.48645300	2.78672400
H	6.92719400	2.45010300	2.43419400
H	6.99414400	1.29396900	3.76518200
H	6.91339100	0.71656200	2.09971300
C	7.53826100	-1.52112800	0.09466200
H	7.84732500	-0.51762600	-0.20680800

H	7.88834600	-1.68771900	1.11356700
H	8.04801500	-2.23722600	-0.55316900
C	-6.55460600	-1.48645300	-2.78672400
H	-6.99414400	-1.29396900	-3.76518200
H	-6.92719400	-2.45010300	-2.43419400
H	-6.91339100	-0.71656200	-2.09971300
C	-7.53826100	1.52112800	-0.09466200
H	-7.88834600	1.68771900	-1.11356700
H	-7.84732500	0.51762600	0.20680800
H	-8.04801500	2.23722600	0.55316900

NDI3 IIb

O	-2.08639900	-0.12849200	-4.14880800
C	-1.39507600	0.70503900	-3.60786300
N	-0.65768900	1.62015200	-4.35020900
C	0.29058800	2.50102700	-3.82241200
O	0.99442600	3.16444400	-4.54405800
C	0.34323400	2.61768300	-2.33905600
C	-0.49196100	1.80782000	-1.54357800
C	-1.32339900	0.82785700	-2.12372700
C	-2.07158800	-0.00685400	-1.32939700
C	-2.00759400	0.10420400	0.07268800
C	-0.81997800	1.60443200	-5.81109600
C	0.05635800	0.56818500	-6.50275500
C	0.05383500	0.77775500	-8.01228600
H	-2.69763800	-0.75548100	-1.80024200
H	-2.57078100	-0.56786800	0.70917100
H	-0.56613600	2.60356200	-6.15919300
H	-1.87197900	1.40806200	-6.01359500
H	1.07307200	0.64409200	-6.10834400
H	-0.32398500	-0.42638200	-6.26539900
H	0.46788200	1.75407100	-8.27002400
H	-0.96072200	0.72463900	-8.41254000
O	1.12836200	3.88335100	2.45910300
C	0.45866700	3.03629500	1.91704500
N	-0.30513400	2.13846900	2.66597000
C	-1.10646500	1.13006800	2.13871900
O	-1.68340100	0.34307500	2.85812200
C	-1.20988900	1.05763900	0.65675400
C	-0.44176700	1.92980000	-0.14165400
C	0.41459800	2.88688600	0.43830900
C	1.21425400	3.67347000	-0.35476900
C	1.18320200	3.53320800	-1.75512000
C	-0.25874300	2.27956600	4.12757500
C	-1.23803700	3.33200600	4.62883300
C	-1.20164000	3.41235200	6.15061000

H	1.86478100	4.39851000	0.11742900
H	1.81431500	4.14163500	-2.38978500
H	-0.50876500	1.30661700	4.53948900
H	0.76526500	2.52709800	4.40135500
H	-2.24324000	3.06807100	4.29029100
H	-0.98141100	4.29822100	4.18992800
H	-1.48300700	2.45572100	6.59646300
H	-0.19974600	3.66559000	6.50296700
O	-1.12836200	-3.88335100	-2.45910300
C	-0.45866700	-3.03629500	-1.91704500
N	0.30513400	-2.13846900	-2.66597000
C	1.10646500	-1.13006800	-2.13871900
O	1.68340100	-0.34307500	-2.85812200
C	1.20988900	-1.05763900	-0.65675400
C	0.44176700	-1.92980000	0.14165400
C	-0.41459800	-2.88688600	-0.43830900
C	-1.21425400	-3.67347000	0.35476900
C	-1.18320200	-3.53320800	1.75512000
C	0.25874300	-2.27956600	-4.12757500
C	1.23803700	-3.33200600	-4.62883300
C	1.20164000	-3.41235200	-6.15061000
H	-1.86478100	-4.39851000	-0.11742900
H	-1.81431500	-4.14163500	2.38978500
H	0.50876500	-1.30661700	-4.53948900
H	-0.76526500	-2.52709800	-4.40135500
H	2.24324000	-3.06807100	-4.29029100
H	0.98141100	-4.29822100	-4.18992800
H	1.48300700	-2.45572100	-6.59646300
H	0.19974600	-3.66559000	-6.50296700
O	2.08639900	0.12849200	4.14880800
C	1.39507600	-0.70503900	3.60786300
N	0.65768900	-1.62015200	4.35020900
C	-0.29058800	-2.50102700	3.82241200
O	-0.99442600	-3.16444400	4.54405800
C	-0.34323400	-2.61768300	2.33905600
C	0.49196100	-1.80782000	1.54357800
C	1.32339900	-0.82785700	2.12372700
C	2.07158800	0.00685400	1.32939700
C	2.00759400	-0.10420400	-0.07268800
C	0.81997800	-1.60443200	5.81109600
C	-0.05635800	-0.56818500	6.50275500
C	-0.05383500	-0.77775500	8.01228600
H	2.69763800	0.75548100	1.80024200
H	2.57078100	0.56786800	-0.70917100
H	0.56613600	-2.60356200	6.15919300
H	1.87197900	-1.40806200	6.01359500

H	-1.07307200	-0.64409200	6.10834400
H	0.32398500	0.42638200	6.26539900
H	-0.46788200	-1.75407100	8.27002400
H	0.96072200	-0.72463900	8.41254000
H	0.64986000	0.01599600	-8.51488600
H	1.88984200	-4.17082900	-6.52283000
H	-0.64986000	-0.01599600	8.51488600
H	-1.88984200	4.17082900	6.52283000

NDI4 Ib

O	-0.51749900	1.53852500	-4.18039100
O	3.38005500	2.59550100	-2.11785300
O	-3.50999500	0.41643400	2.08465600
O	0.29573400	1.77703000	4.14742800
N	1.41288000	2.14987500	-3.15462900
N	-1.58746800	1.03600200	3.11969300
C	0.08532000	1.72593300	-3.14907600
C	-0.55111100	1.52134000	-1.81815600
C	0.18621300	1.77710800	-0.64381500
C	1.53278200	2.19123900	-0.70007700
C	2.20392800	2.33266000	-2.02089100
C	-1.85339300	1.08833000	-1.74244100
H	-2.39902900	0.90329200	-2.65995300
C	-2.45307100	0.86813100	-0.48847700
H	-3.47069300	0.50409000	-0.41706700
C	-1.74922000	1.10517200	0.66817900
C	-0.41831200	1.56605900	0.61036500
C	0.32542000	1.80605900	1.78442900
C	1.62908200	2.23435800	1.70677100
H	2.18151100	2.40121000	2.62280200
C	2.24054300	2.42234000	0.45426900
H	3.27674100	2.72642800	0.38329600
C	-2.37683100	0.82813300	1.98800500
C	-0.29573400	1.56028200	3.11436200
C	2.06664600	2.31204900	-4.46257700
H	2.79410500	3.11493500	-4.35933600
H	1.29353400	2.60949000	-5.16763200
C	2.73944100	1.02377600	-4.91476500
H	3.52093300	0.76433900	-4.19336400
H	1.99946100	0.21865400	-4.91571400
C	3.35374500	1.16509200	-6.30306400
H	4.07283200	1.98979400	-6.30253100
H	2.56860500	1.43343900	-7.01597300
C	-2.19094300	0.74276000	4.42894800
H	-1.38432700	0.43031900	5.08811200
H	-2.87002600	-0.09516200	4.28501600

C	-2.92652100	1.95131000	4.99022500
H	-3.70722300	2.25326600	4.28607400
H	-2.22243700	2.78325000	5.08340500
C	-3.54683300	1.64698500	6.34940700
H	-2.76097200	1.32772300	7.04011700
H	-4.23669700	0.80386300	6.25001900
O	-0.29573400	-1.77703000	4.14742800
O	3.50999500	-0.41643400	2.08465600
O	-3.38005500	-2.59550100	-2.11785300
O	0.51749900	-1.53852500	-4.18039100
N	1.58746800	-1.03600200	3.11969300
N	-1.41288000	-2.14987500	-3.15462900
C	0.29573400	-1.56028200	3.11436200
C	-0.32542000	-1.80605900	1.78442900
C	0.41831200	-1.56605900	0.61036500
C	1.74922000	-1.10517200	0.66817900
C	2.37683100	-0.82813300	1.98800500
C	-1.62908200	-2.23435800	1.70677100
H	-2.18151100	-2.40121000	2.62280200
C	-2.24054300	-2.42234000	0.45426900
H	-3.27674100	-2.72642800	0.38329600
C	-1.53278200	-2.19123900	-0.70007700
C	-0.18621300	-1.77710800	-0.64381500
C	0.55111100	-1.52134000	-1.81815600
C	1.85339300	-1.08833000	-1.74244100
H	2.39902900	-0.90329200	-2.65995300
C	2.45307100	-0.86813100	-0.48847700
H	3.47069300	-0.50409000	-0.41706700
C	-2.20392800	-2.33266000	-2.02089100
C	-0.08532000	-1.72593300	-3.14907600
C	2.19094300	-0.74276000	4.42894800
H	2.87002600	0.09516200	4.28501600
H	1.38432700	-0.43031900	5.08811200
C	2.92652100	-1.95131000	4.99022500
H	3.70722300	-2.25326600	4.28607400
H	2.22243700	-2.78325000	5.08340500
C	3.54683300	-1.64698500	6.34940700
H	4.23669700	-0.80386300	6.25001900
H	2.76097200	-1.32772300	7.04011700
C	-2.06664600	-2.31204900	-4.46257700
H	-1.29353400	-2.60949000	-5.16763200
H	-2.79410500	-3.11493500	-4.35933600
C	-2.73944100	-1.02377600	-4.91476500
H	-3.52093300	-0.76433900	-4.19336400
H	-1.99946100	-0.21865400	-4.91571400
C	-3.35374500	-1.16509200	-6.30306400

H	-2.56860500	-1.43343900	-7.01597300
H	-4.07283200	-1.98979400	-6.30253100
C	4.04051300	-0.11906400	-6.75467500
H	4.46315300	-0.01618500	-7.75467900
H	4.85134700	-0.38431100	-6.07306900
H	3.33201700	-0.94951300	-6.76952700
C	-4.28352000	2.84994300	6.92745800
H	-5.08834700	3.16815000	6.26179400
H	-4.72234700	2.62042100	7.89891100
H	-3.60417800	3.69486300	7.05670100
C	-4.04051300	0.11906400	-6.75467500
H	-4.46315300	0.01618500	-7.75467900
H	-4.85134700	0.38431100	-6.07306900
H	-3.33201700	0.94951300	-6.76952700
C	4.28352000	-2.84994300	6.92745800
H	5.08834700	-3.16815000	6.26179400
H	4.72234700	-2.62042100	7.89891100
H	3.60417800	-3.69486300	7.05670100

NDI4 IIa

C	1.06121500	-2.25174300	-0.57847800
C	-0.32589800	-2.22188800	-0.33791400
C	1.84688100	-3.25352400	0.02378500
C	1.64025800	-1.27531000	-1.41351800
C	-1.10495500	-1.18803400	-0.89898300
C	-0.90249400	-3.19868300	0.49697100
C	1.26612600	-4.19805900	0.83492400
C	0.85948200	-0.29929900	-1.98193800
C	-0.52349000	-0.24878900	-1.71445000
C	-0.12095400	-4.17560900	1.06630100
C	3.31777800	-3.27546700	-0.20394700
C	3.11359700	-1.26954100	-1.63210600
C	-2.54750900	-1.08363100	-0.54705200
C	-2.36901200	-3.18427200	0.74691000
H	1.89422100	-4.95461500	1.28705600
H	1.32949700	0.44192400	-2.61708700
H	-1.13981600	0.53955900	-2.12932700
H	-0.59232900	-4.91933100	1.69575600
O	4.02934700	-4.11305300	0.29826400
O	3.64583700	-0.41551700	-2.30544900
O	-3.23008600	-0.15356600	-0.92391600
O	-2.92034300	-4.06185400	1.36602800
N	3.84770100	-2.28720700	-1.03224100
N	-3.08282700	-2.08959800	0.24695200
C	5.31266400	-2.25771400	-1.16616700
C	-4.52129400	-2.01890600	0.55257000

C	5.98667200	-1.67371900	0.07094700
H	5.64405000	-3.28251200	-1.32916600
H	5.53570800	-1.66376700	-2.04931400
C	-5.38270700	-2.50007800	-0.60510600
H	-4.68174700	-2.63159200	1.43584500
H	-4.74768400	-0.98652100	0.80605300
H	5.55190900	-0.69549300	0.29507000
H	5.77376500	-2.31708200	0.92767900
H	-5.18245100	-1.88226900	-1.48547100
H	-5.11575200	-3.53146900	-0.85427800
C	0.32589800	2.22188800	0.33791400
C	-1.06121500	2.25174300	0.57847800
C	1.10495500	1.18803400	0.89898300
C	0.90249400	3.19868300	-0.49697100
C	-1.84688100	3.25352400	-0.02378500
C	-1.64025800	1.27531000	1.41351800
C	0.52349000	0.24878900	1.71445000
C	0.12095400	4.17560900	-1.06630100
C	-1.26612600	4.19805900	-0.83492400
C	-0.85948200	0.29929900	1.98193800
C	2.54750900	1.08363100	0.54705200
C	2.36901200	3.18427200	-0.74691000
C	-3.31777800	3.27546700	0.20394700
C	-3.11359700	1.26954100	1.63210600
H	1.13981600	-0.53955900	2.12932700
H	0.59232900	4.91933100	-1.69575600
H	-1.89422100	4.95461500	-1.28705600
H	-1.32949700	-0.44192400	2.61708700
O	3.23008600	0.15356600	0.92391600
O	2.92034300	4.06185400	-1.36602800
O	-4.02934700	4.11305300	-0.29826400
O	-3.64583700	0.41551700	2.30544900
N	3.08282700	2.08959800	-0.24695200
N	-3.84770100	2.28720700	1.03224100
C	4.52129400	2.01890600	-0.55257000
C	-5.31266400	2.25771400	1.16616700
C	5.38270700	2.50007800	0.60510600
H	4.74768400	0.98652100	-0.80605300
H	4.68174700	2.63159200	-1.43584500
C	-5.98667200	1.67371900	-0.07094700
H	-5.53570800	1.66376700	2.04931400
H	-5.64405000	3.28251200	1.32916600
H	5.11575200	3.53146900	0.85427800
H	5.18245100	1.88226900	1.48547100
H	-5.77376500	2.31708200	-0.92767900
H	-5.55190900	0.69549300	-0.29507000

C	6.86294500	2.41724500	0.24708300
H	7.06087800	3.04801900	-0.62425600
H	7.09891500	1.39120700	-0.05435400
C	7.49432000	-1.54988600	-0.12022200
H	7.70060500	-0.84652800	-0.93354200
H	7.90397500	-2.51468500	-0.43447800
C	-6.86294500	-2.41724500	-0.24708300
H	-7.06087800	-3.04801900	0.62425600
H	-7.09891500	-1.39120700	0.05435400
C	-7.49432000	1.54988600	0.12022200
H	-7.70060500	0.84652800	0.93354200
H	-7.90397500	2.51468500	0.43447800
C	7.76412600	2.83527000	1.40278700
H	7.58396700	2.21245500	2.28166600
H	8.81859000	2.74580100	1.13938300
H	7.57471400	3.87214200	1.68735100
C	8.19404600	-1.09058000	1.15446900
H	8.05949300	-1.82266600	1.95276800
H	9.26476200	-0.95433600	0.99818400
H	7.78214800	-0.14150000	1.50476700
C	-7.76412600	-2.83527000	-1.40278700
H	-8.81859000	-2.74580100	-1.13938300
H	-7.58396700	-2.21245500	-2.28166600
H	-7.57471400	-3.87214200	-1.68735100
C	-8.19404600	1.09058000	-1.15446900
H	-9.26476200	0.95433600	-0.99818400
H	-8.05949300	1.82266600	-1.95276800
H	-7.78214800	0.14150000	-1.50476700

NDI4 IIb

O	-4.00399400	-0.50928500	-2.36047500
C	-3.41645900	-1.21391400	-1.57099800
N	-4.09410200	-2.15289600	-0.80190200
C	-3.52687900	-2.90094600	0.23421400
O	-4.20939900	-3.59690500	0.94583900
C	-2.04555000	-2.83819300	0.37238300
C	-1.29915300	-1.99313000	-0.47332900
C	-1.93662300	-1.14576800	-1.40256000
C	-1.20032400	-0.26947500	-2.16281800
C	0.19762400	-0.20379700	-2.01287400
C	-5.53187900	-2.33145400	-1.05243000
C	-6.40800100	-1.32788600	-0.31422700
C	-7.87837600	-1.72841000	-0.38044800
H	-1.71569100	0.37405200	-2.86590300
H	0.78510400	0.50367900	-2.58578400
H	-5.77425200	-3.34386000	-0.73573300

H	-5.68044000	-2.24446000	-2.12778500
H	-6.08395900	-1.26678400	0.72972900
H	-6.27811300	-0.34056900	-0.76433900
H	-8.00644300	-2.70069500	0.10359100
H	-8.17080900	-1.85871400	-1.42696200
O	2.81117300	-3.46517600	1.50122000
C	2.21591400	-2.73131800	0.74862000
N	2.89830800	-1.79103800	-0.02623500
C	2.30800900	-0.91058800	-0.92775900
O	2.96573500	-0.08059300	-1.51843400
C	0.83809600	-1.02693900	-1.11889800
C	0.10147000	-1.94042600	-0.33744800
C	0.73541900	-2.76738600	0.61138900
C	-0.00877800	-3.59357000	1.41801600
C	-1.41169400	-3.62352100	1.30296900
C	4.35859300	-1.73077900	0.12079100
C	5.06632800	-2.73042600	-0.78235000
C	6.57912100	-2.55780500	-0.69821400
H	0.50334100	-4.21628200	2.14039900
H	-2.00908900	-4.26237000	1.94039300
H	4.65288800	-0.71836100	-0.13979800
H	4.59013500	-1.90715000	1.16944400
H	4.73702200	-2.57449700	-1.81423800
H	4.78424700	-3.74627100	-0.49283900
H	6.83652100	-1.53861500	-1.00250700
H	6.89658700	-2.65987100	0.34440000
O	-2.81247500	3.46452900	-1.49937700
C	-2.21662200	2.73087600	-0.74705200
N	-2.89831600	1.79038300	0.02815500
C	-2.30731100	0.91029900	0.92958000
O	-2.96448800	0.08014700	1.52063000
C	-0.83736300	1.02723200	1.12009100
C	-0.10140000	1.94079800	0.33811600
C	-0.73606500	2.76738200	-0.61057400
C	0.00749800	3.59350200	-1.41784600
C	1.41048000	3.62371200	-1.30365600
C	-4.35861600	1.72940400	-0.11832900
C	-5.06657900	2.72838800	0.78536000
C	-6.57931000	2.55510500	0.70151100
H	-0.50516200	4.21590900	-2.14010800
H	2.00739100	4.26243500	-1.94166000
H	-4.65228200	0.71674900	0.14208600
H	-4.59062200	1.90596500	-1.16684800
H	-4.73694500	2.57223600	1.81710600
H	-4.78503800	3.74446400	0.49614400
H	-6.83617700	1.53565500	1.00537100

H	-6.89705900	2.65751500	-0.34098200
O	4.00547200	0.51145200	2.35954900
C	3.41736500	1.21555600	1.57002300
N	4.09440000	2.15426400	0.80007800
C	3.52644400	2.90169400	-0.23607000
O	4.20841900	3.59729600	-0.94856800
C	2.04502700	2.83877700	-0.37320500
C	1.29928000	1.99388200	0.47324500
C	1.93745800	1.14696800	1.40239000
C	1.20178900	0.27063200	2.16321300
C	-0.19620600	0.20446900	2.01392600
C	5.53238100	2.33284400	1.04937200
C	6.40776000	1.32844400	0.31139700
C	7.87817300	1.72920900	0.37540500
H	1.71768600	-0.37255100	2.86622400
H	-0.78319000	-0.50307900	2.58725100
H	5.77465800	3.34491900	0.73155700
H	5.68176200	2.24682300	2.12469200
H	6.08249300	1.26598000	-0.73209700
H	6.27849300	0.34168700	0.76291200
H	8.00559500	2.70078600	-0.11022600
H	8.17176100	1.86110400	1.42139400
C	-8.78732400	-0.69789800	0.27952700
H	-8.70510200	0.26940800	-0.22065200
H	-9.83246100	-1.00665000	0.24427800
H	-8.51446800	-0.55527000	1.32748100
C	-7.33113400	3.55603500	1.57061300
H	-7.11318200	4.58002200	1.26128000
H	-8.40966200	3.40791700	1.50619800
H	-7.04045400	3.45504500	2.61816200
C	8.78649700	0.69781000	-0.28403300
H	9.83162300	1.00679800	-0.25055900
H	8.70506400	-0.26870900	0.21779900
H	8.51238000	0.55346000	-1.33142200
C	7.33068100	-3.55949100	-1.56667700
H	8.40926400	-3.41182700	-1.50210900
H	7.11220300	-4.58323300	-1.25689000
H	7.04026000	-3.45888000	-2.61433500

NDI5 Ib

O	-4.18395000	-0.66500400	1.48628200
O	-2.09563500	3.08933800	2.93786900
O	2.05943900	-3.55408100	0.02641700
O	4.14902700	0.07455600	1.77460000
N	-3.14546500	1.18531300	2.29225800
N	3.10814700	-1.71552600	0.84532500

C	-3.14853000	-0.09038200	1.73108500
C	-1.82208600	-0.70818700	1.45465200
C	-0.64270900	-0.00905900	1.78370400
C	-0.69007500	1.28570900	2.34009500
C	-2.00657500	1.94627600	2.55415900
C	-1.75551300	-1.95687500	0.88411800
H	-2.67697400	-2.47328900	0.64330400
C	-0.50591700	-2.53708200	0.59737300
H	-0.44194100	-3.51028500	0.12620700
C	0.65562800	-1.86889300	0.90389300
C	0.60708700	-0.59460200	1.50444400
C	1.78640100	0.11248600	1.81818000
C	1.71744800	1.36279900	2.38465800
H	2.63717200	1.88908800	2.60688700
C	0.46919800	1.95798400	2.64157200
H	0.40587500	2.95651200	3.05402600
C	1.97087400	-2.47079800	0.55709900
C	3.11195300	-0.48598500	1.50198100
C	-4.45007100	1.82543000	2.52205900
H	-4.34069200	2.47281000	3.38991400
H	-5.15685700	1.02993900	2.74767000
C	-4.90477500	2.61828100	1.30478100
H	-4.18286700	3.42016000	1.11979100
H	-4.90765600	1.95765400	0.43325700
C	-6.29206900	3.21597800	1.50707500
H	-6.29183400	3.86086500	2.39277600
H	-7.00735700	2.41081000	1.70678300
C	4.41264400	-2.29150400	0.48371800
H	5.07608200	-1.45937800	0.25984600
H	4.26209500	-2.87422400	-0.42278900
C	4.97134900	-3.15953200	1.60252600
H	4.25918200	-3.96006400	1.82215500
H	5.07617300	-2.55004900	2.50466800
C	6.32093600	-3.75788500	1.22364800
H	7.02268800	-2.95147800	0.98524300
H	6.21162100	-4.35454400	0.31162400
O	4.14848700	-0.07538900	-1.77563900
O	2.05933000	3.55304600	-0.02652100
O	-2.09655900	-3.09016700	-2.93719300
O	-4.18440900	0.66465400	-1.48614600
N	3.10783300	1.71460000	-0.84590400
N	-3.14616000	-1.18593300	-2.29180900
C	3.11148600	0.48509700	-1.50263800
C	1.78584600	-0.11340000	-1.81845200
C	0.60661300	0.59371000	-1.50445900
C	0.65530700	1.86798900	-0.90389400

C	1.97062800	2.46982300	-0.55730900
C	1.71673300	-1.36372800	-2.38488100
H	2.63639300	-1.89004100	-2.60731800
C	0.46840800	-1.95888900	-2.64150700
H	0.40497300	-2.95743400	-3.05390300
C	-0.69078500	-1.28655600	-2.33984600
C	-0.64325600	0.00823500	-1.78352700
C	-1.82254200	0.70745600	-1.45434600
C	-1.75581600	1.95613300	-0.88381000
H	-2.67720900	2.47263700	-0.64294700
C	-0.50614900	2.53624000	-0.59718300
H	-0.44204200	3.50942700	-0.12600400
C	-2.00736700	-1.94705600	-2.55366400
C	-3.14906400	0.08983400	-1.73079700
C	4.41235800	2.29070900	-0.48461600
H	4.26214900	2.87272100	0.42240900
H	5.07618500	1.45863500	-0.26172700
C	4.97010400	3.15976300	-1.60310700
H	4.25726700	3.95987600	-1.82207700
H	5.07512000	2.55086000	-2.50562200
C	6.31931000	3.75899600	-1.22427100
H	6.20975400	4.35494600	-0.31181200
H	7.02184900	2.95304800	-0.98663100
C	-4.45087900	-1.82577500	-2.52171700
H	-5.15737400	-1.03015500	-2.74780000
H	-4.34145400	-2.47348000	-3.38932200
C	-4.90621000	-2.61804700	-1.30429600
H	-4.18487000	-3.42037100	-1.11902100
H	-4.90873200	-1.95722600	-0.43292300
C	-6.29391300	-3.21478300	-1.50664500
H	-7.00853400	-2.40912800	-1.70678700
H	-6.29400600	-3.85997700	-2.39212300
C	-6.76380400	4.01781300	0.29814200
H	-6.05211200	4.82600700	0.10507200
H	-6.74723800	3.37265700	-0.58492800
C	6.91113800	-4.62948500	2.32748300
H	6.20709100	-5.43226000	2.56531300
H	7.01803800	-4.03172900	3.23740500
C	-6.76655700	-4.01581300	-0.29753600
H	-6.05559400	-4.82454500	-0.10403600
H	-6.74963300	-3.37036800	0.58531300
C	6.90837000	4.63188500	-2.32769900
H	6.20345000	5.43409900	-2.56484000
H	7.01565500	4.03482400	-3.23803200
C	-8.16038800	4.59743400	0.49144800
H	-8.18950500	5.26179200	1.35767500

H	-8.48105200	5.16934000	-0.37996800
H	-8.89077900	3.80298200	0.65799000
C	-8.16359400	-4.59430300	-0.49094600
H	-8.19309700	-5.25892600	-1.35695200
H	-8.48493900	-5.16564100	0.38059200
H	-8.89326500	-3.79927800	-0.65791300
C	8.25687200	5.22867100	-1.94120700
H	8.98221200	4.44166300	-1.72487300
H	8.66427600	5.84933300	-2.73996900
H	8.16500600	5.84962300	-1.04774500
C	8.26016000	-5.22513300	1.94105200
H	8.66839500	-5.84485900	2.74011500
H	8.16873100	-5.84675200	1.04800800
H	8.98463500	-4.43751500	1.72404300

NDI5 IIa

C	-0.99369500	2.26888900	-0.59955700
C	0.38439900	2.20482300	-0.31788900
C	-1.77386300	3.28302700	-0.01112800
C	-1.56994100	1.31277200	-1.45988800
C	1.15566000	1.15632800	-0.86243300
C	0.95833500	3.16239000	0.54065100
C	-1.19620500	4.20743700	0.82503600
C	-0.79522400	0.32280600	-2.01219500
C	0.57725200	0.23682800	-1.70209400
C	0.18265800	4.15206200	1.09584400
C	-3.23589200	3.34226600	-0.28356700
C	-3.03552700	1.34520600	-1.72454200
C	2.58415100	1.01611500	-0.46785000
C	2.41683900	3.11546700	0.82988400
H	-1.82022100	4.97430800	1.26526800
H	-1.26255800	-0.40179300	-2.66819300
H	1.18730700	-0.56327000	-2.10358300
H	0.65235100	4.88044300	1.74420800
O	-3.94270500	4.19187300	0.20515700
O	-3.56758900	0.51041100	-2.42171300
O	3.25492600	0.06995200	-0.82540500
O	2.96881600	3.97817600	1.46902700
N	-3.76268200	2.37619100	-1.13939100
N	3.12092500	2.00947500	0.34108800
C	-5.22106900	2.39325300	-1.33005800
C	4.55033000	1.90752800	0.67962900
C	-5.96580300	1.81514400	-0.13106600
H	-5.51327600	3.43065900	-1.48845200
H	-5.42782900	1.82069700	-2.23107000
C	5.44704500	2.33392300	-0.47271400

H	4.70889600	2.53970700	1.54925000
H	4.74256400	0.87659500	0.96504300
H	-5.60257100	0.80397300	0.07286600
H	-5.73732500	2.41717600	0.75110200
H	5.25813500	1.68735600	-1.33434800
H	5.20027300	3.35898200	-0.76496500
C	-0.38430900	-2.20501700	0.31838900
C	0.99376600	-2.26886500	0.60019500
C	-1.15571600	-1.15642000	0.86254000
C	-0.95808400	-3.16293200	-0.53986600
C	1.77406500	-3.28318600	0.01225700
C	1.56983900	-1.31243300	1.46028800
C	-0.57749600	-0.23668100	1.70206900
C	-0.18229000	-4.15280000	-1.09455300
C	1.19654400	-4.20799400	-0.82356300
C	0.79496200	-0.32242800	2.01230200
C	-2.58415700	-1.01639500	0.46770600
C	-2.41653900	-3.11614300	-0.82936400
C	3.23609000	-3.34214600	0.28478900
C	3.03542900	-1.34451900	1.72494500
H	-1.18766800	0.56345400	2.10331000
H	-0.65186600	-4.88146300	-1.74268500
H	1.82064700	-4.97502100	-1.26340200
H	1.26216500	0.40240200	2.66814000
O	-3.25503800	-0.07017100	0.82489800
O	-2.96837700	-3.97905900	-1.46834700
O	3.94298900	-4.19199900	-0.20338200
O	3.56730100	-0.50941600	2.42189000
N	-3.12073900	-2.00999300	-0.34107200
N	3.76280000	-2.37547800	1.13999400
C	-4.55004400	-1.90808400	-0.68007500
C	5.22124200	-2.39205700	1.33036600
C	-5.44718000	-2.33350800	0.47230300
H	-4.74202600	-0.87733500	-0.96634700
H	-4.70844400	-2.54092100	-1.54925000
C	5.96565400	-1.81527400	0.13052000
H	5.42805900	-1.81835900	2.23063700
H	5.51370200	-3.42920900	1.48999300
H	-5.20034200	-3.35823800	0.76566400
H	-5.25877300	-1.68606300	1.33339000
H	5.73752100	-2.41872600	-0.75076400
H	5.60188600	-0.80459500	-0.07490200
C	-6.91698200	-2.24679700	0.07691400
H	-7.11333400	-2.92790000	-0.75782500
H	-7.13407000	-1.23697300	-0.29302800
C	-7.47056700	1.80530100	-0.37430000

H	-7.70206400	1.15615200	-1.22709800
H	-7.80117400	2.81176200	-0.65445800
C	6.91700100	2.24650600	-0.07806500
H	7.11387300	2.92672900	0.75726300
H	7.13401900	1.23626800	0.29076000
C	7.47041100	-1.80429300	0.37373700
H	7.70160000	-1.15373500	1.22554600
H	7.80152500	-2.81015500	0.65543400
C	-7.85887900	-2.57347400	1.23065200
H	-7.65311700	-1.89454700	2.06422700
H	-7.64355100	-3.58215800	1.59536200
C	-8.26779600	1.34312900	0.84129200
H	-8.02242000	1.98131100	1.69485700
H	-7.95534800	0.33030300	1.11612000
C	7.85846700	2.57411500	-1.23188600
H	7.65215200	1.89608100	-2.06605000
H	7.64323100	3.58322400	-1.59547100
C	8.26741600	-1.34359700	-0.84256800
H	8.02186400	-1.98280700	-1.69529500
H	7.95495100	-0.33110000	-1.11858400
C	-9.32586200	-2.46829100	0.83152900
H	-9.55734900	-3.15560700	0.01528800
H	-9.98723500	-2.70306300	1.66627100
H	-9.56144900	-1.45735800	0.49193700
C	-9.77148500	1.37287800	0.59130500
H	-10.32973200	0.99682600	1.44970800
H	-10.11052800	2.39098800	0.38908500
H	-10.03483200	0.76059100	-0.27440300
C	9.32560600	2.46814700	-0.83353600
H	9.98665900	2.70382000	-1.66827600
H	9.55759300	3.15440700	-0.01654600
H	9.56113800	1.45675000	-0.49530100
C	9.77114000	-1.37315400	-0.59282100
H	10.32927300	-0.99800600	-1.45169200
H	10.03470800	-0.76007100	0.27223700
H	10.11011700	-2.39109200	-0.38968100

NDI5 IIb

O	-3.95513100	-0.75638600	-2.42861200
C	-3.36025700	-1.40182100	-1.59521100
N	-4.02096100	-2.32865500	-0.79715700
C	-3.44673800	-3.01631600	0.27658400
O	-4.11741300	-3.70571700	1.00555300
C	-1.97053800	-2.89864900	0.43497500
C	-1.23869500	-2.05832600	-0.42799400
C	-1.88816500	-1.26975700	-1.39950600

C	-1.16985400	-0.39361000	-2.17711100
C	0.22165300	-0.27163700	-2.00502300
C	-5.44951200	-2.55867700	-1.06131200
C	-6.35853900	-1.53261200	-0.39813100
C	-7.82325100	-1.94697800	-0.48836600
H	-1.69488900	0.20492300	-2.91207400
H	0.79428400	0.43604100	-2.59246300
H	-5.67363500	-3.55642500	-0.68996100
H	-5.58060000	-2.53738600	-2.14234200
H	-6.06552900	-1.41974100	0.65073600
H	-6.22277600	-0.56680900	-0.89065200
H	-7.96563300	-2.89773900	0.03603900
H	-8.08928900	-2.12545200	-1.53622700
O	2.88322700	-3.29983300	1.67799200
C	2.27665000	-2.62164000	0.88328600
N	2.93978600	-1.68886000	0.08332700
C	2.33564200	-0.86486100	-0.86171300
O	2.97417400	-0.03511300	-1.47329300
C	0.87445700	-1.04017100	-1.07211900
C	0.15642100	-1.95039400	-0.27005300
C	0.80163200	-2.71924400	0.71935700
C	0.07209700	-3.54223400	1.54246900
C	-1.32661200	-3.62683000	1.40431300
C	4.39063700	-1.55518200	0.26722600
C	5.17855000	-2.56459600	-0.55545100
C	6.67147000	-2.27095800	-0.46024000
H	0.59222900	-4.11913700	2.29649500
H	-1.91280200	-4.26270700	2.05495900
H	4.64572800	-0.54618500	-0.04286700
H	4.59810900	-1.66112000	1.33048200
H	4.85839900	-2.50278700	-1.59989000
H	4.96309100	-3.57518900	-0.19895300
H	6.86167000	-1.26570400	-0.85244800
H	6.97310800	-2.25171500	0.59370700
O	-2.88322700	3.29983300	-1.67799200
C	-2.27665000	2.62164000	-0.88328600
N	-2.93978600	1.68886000	-0.08332700
C	-2.33564200	0.86486100	0.86171300
O	-2.97417400	0.03511300	1.47329300
C	-0.87445700	1.04017100	1.07211900
C	-0.15642100	1.95039400	0.27005300
C	-0.80163200	2.71924400	-0.71935700
C	-0.07209700	3.54223400	-1.54246900
C	1.32661200	3.62683000	-1.40431300
C	-4.39063700	1.55518200	-0.26722600
C	-5.17855000	2.56459600	0.55545100

C	-6.67147000	2.27095800	0.46024000
H	-0.59222900	4.11913700	-2.29649500
H	1.91280200	4.26270700	-2.05495900
H	-4.64572800	0.54618500	0.04286700
H	-4.59810900	1.66112000	-1.33048200
H	-4.85839900	2.50278700	1.59989000
H	-4.96309100	3.57518900	0.19895300
H	-6.86167000	1.26570400	0.85244800
H	-6.97310800	2.25171500	-0.59370700
O	3.95513100	0.75638600	2.42861200
C	3.36025700	1.40182100	1.59521100
N	4.02096100	2.32865500	0.79715700
C	3.44673800	3.01631600	-0.27658400
O	4.11741300	3.70571700	-1.00555300
C	1.97053800	2.89864900	-0.43497500
C	1.23869500	2.05832600	0.42799400
C	1.88816500	1.26975700	1.39950600
C	1.16985400	0.39361000	2.17711100
C	-0.22165300	0.27163700	2.00502300
C	5.44951200	2.55867700	1.06131200
C	6.35853900	1.53261200	0.39813100
C	7.82325100	1.94697800	0.48836600
H	1.69488900	-0.20492300	2.91207400
H	-0.79428400	-0.43604100	2.59246300
H	5.67363500	3.55642500	0.68996100
H	5.58060000	2.53738600	2.14234200
H	6.06552900	1.41974100	-0.65073600
H	6.22277600	0.56680900	0.89065200
H	7.96563300	2.89773900	-0.03603900
H	8.08928900	2.12545200	1.53622700
C	-8.76510100	-0.89887600	0.09549600
H	-8.62685500	0.04587000	-0.43931300
H	-8.48412000	-0.70644700	1.13603700
C	-7.53950400	3.27296900	1.21231500
H	-7.37097500	4.27416400	0.80542200
H	-7.22517600	3.30627900	2.25966000
C	8.76510100	0.89887600	-0.09549600
H	8.62685500	-0.04587000	0.43931300
H	8.48412000	0.70644700	-1.13603700
C	7.53950400	-3.27296900	-1.21231500
H	7.37097500	-4.27416400	-0.80542200
H	7.22517600	-3.30627900	-2.25966000
C	10.22855900	1.31886000	-0.02742700
H	10.53428300	1.49474500	1.00598700
H	10.88298700	0.55221400	-0.44434700
H	10.39499400	2.24257400	-0.58526800

C	9.02118100	-2.92173600	-1.12996400
H	9.63720300	-3.64509900	-1.66509900
H	9.20936200	-1.93520400	-1.55982500
H	9.35685000	-2.89829800	-0.09069600
C	-10.22855900	-1.31886000	0.02742700
H	-10.88298700	-0.55221400	0.44434700
H	-10.53428300	-1.49474500	-1.00598700
H	-10.39499400	-2.24257400	0.58526800
C	-9.02118100	2.92173600	1.12996400
H	-9.63720300	3.64509900	1.66509900
H	-9.35685000	2.89829800	0.09069600
H	-9.20936200	1.93520400	1.55982500

NDI6 Ib

O	-0.84146300	1.38490400	-4.19514400
O	2.69149200	3.30763600	-2.10156300
O	-3.53026200	-0.43146000	2.04384000
O	-0.15849500	1.76683900	4.13899600
N	0.88723400	2.42375100	-3.15380500
N	-1.81377100	0.61662300	3.09550200
C	-0.30618300	1.70392300	-3.15892600
C	-0.88723400	1.35352700	-1.83325600
C	-0.23576500	1.76620700	-0.65290500
C	0.97859000	2.48144500	-0.69821200
C	1.60713600	2.78011400	-2.01387400
C	-2.05547300	0.63224000	-1.76819800
H	-2.53896600	0.33234900	-2.69009000
C	-2.59565900	0.27283300	-0.51953600
H	-3.50209500	-0.31679200	-0.45676800
C	-1.97160300	0.65862900	0.64296600
C	-0.78298500	1.41471200	0.59609300
C	-0.12269100	1.81474100	1.77642500
C	1.04653300	2.53394300	1.70942100
H	1.53979800	2.81957000	2.62998000
C	1.60607400	2.86458900	0.46201400
H	2.54447000	3.40009400	0.39997100
C	-2.52468600	0.23516400	1.95715900
C	-0.67813800	1.42496400	3.10113400
C	1.49529500	2.73360300	-4.45709400
H	2.02190700	3.67972000	-4.34789600
H	0.67925300	2.85044900	-5.16678000
C	2.44435000	1.63157700	-4.90649300
H	3.25761000	1.55223100	-4.17805100
H	1.90367700	0.68098800	-4.91581700
C	3.02202900	1.91436600	-6.28835700
H	3.53858700	2.88041200	-6.28166300

H	2.20372800	2.00189700	-7.01105500
C	-2.34065200	0.18286200	4.39877000
H	-1.48814100	0.06899700	5.06427900
H	-2.80034400	-0.79165000	4.24668800
C	-3.34843800	1.17907800	4.95488100
H	-4.16706800	1.29481500	4.23888600
H	-2.86094700	2.15208400	5.06396900
C	-3.89993400	0.72322600	6.30079600
H	-3.07284500	0.58925700	7.00602500
H	-4.37316200	-0.25777100	6.18584100
O	0.15849500	-1.76683900	4.13899600
O	3.53026200	0.43146000	2.04384000
O	-2.69149200	-3.30763600	-2.10156300
O	0.84146300	-1.38490400	-4.19514400
N	1.81377100	-0.61662300	3.09550200
N	-0.88723400	-2.42375100	-3.15380500
C	0.67813800	-1.42496400	3.10113400
C	0.12269100	-1.81474100	1.77642500
C	0.78298500	-1.41471200	0.59609300
C	1.97160300	-0.65862900	0.64296600
C	2.52468600	-0.23516400	1.95715900
C	-1.04653300	-2.53394300	1.70942100
H	-1.53979800	-2.81957000	2.62998000
C	-1.60607400	-2.86458900	0.46201400
H	-2.54447000	-3.40009400	0.39997100
C	-0.97859000	-2.48144500	-0.69821200
C	0.23576500	-1.76620700	-0.65290500
C	0.88723400	-1.35352700	-1.83325600
C	2.05547300	-0.63224000	-1.76819800
H	2.53896600	-0.33234900	-2.69009000
C	2.59565900	-0.27283300	-0.51953600
H	3.50209500	0.31679200	-0.45676800
C	-1.60713600	-2.78011400	-2.01387400
C	0.30618300	-1.70392300	-3.15892600
C	2.34065200	-0.18286200	4.39877000
H	2.80034400	0.79165000	4.24668800
H	1.48814100	-0.06899700	5.06427900
C	3.34843800	-1.17907800	4.95488100
H	4.16706800	-1.29481500	4.23888600
H	2.86094700	-2.15208400	5.06396900
C	3.89993400	-0.72322600	6.30079600
H	4.37316200	0.25777100	6.18584100
H	3.07284500	-0.58925700	7.00602500
C	-1.49529500	-2.73360300	-4.45709400
H	-0.67925300	-2.85044900	-5.16678000
H	-2.02190700	-3.67972000	-4.34789600

C	-2.44435000	-1.63157700	-4.90649300
H	-3.25761000	-1.55223100	-4.17805100
H	-1.90367700	-0.68098800	-4.91581700
C	-3.02202900	-1.91436600	-6.28835700
H	-2.20372800	-2.00189700	-7.01105500
H	-3.53858700	-2.88041200	-6.28166300
C	3.98709200	0.82758700	-6.74960000
H	4.81059600	0.74681700	-6.03162700
H	3.47245600	-0.13911100	-6.73905400
C	-4.90925400	1.70451600	6.88654600
H	-5.73426500	1.84143200	6.17906600
H	-4.43600600	2.68563800	7.00202500
C	-3.98709200	-0.82758700	-6.74960000
H	-4.81059600	-0.74681700	-6.03162700
H	-3.47245600	0.13911100	-6.73905400
C	4.90925400	-1.70451600	6.88654600
H	5.73426500	-1.84143200	6.17906600
H	4.43600600	-2.68563800	7.00202500
C	4.55674900	1.08394500	-8.14083700
H	5.06328400	2.05380100	-8.14888700
H	3.73329700	1.15998100	-8.85720300
C	-4.55674900	-1.08394500	-8.14083700
H	-5.06328400	-2.05380100	-8.14888700
H	-3.73329700	-1.15998100	-8.85720300
C	5.46942000	-1.25435100	8.23180500
H	4.64383900	-1.11694400	8.93646900
H	5.94093900	-0.27413500	8.11410400
C	-5.46942000	1.25435100	8.23180500
H	-5.94093900	0.27413500	8.11410400
H	-4.64383900	1.11694400	8.93646900
C	5.52575700	-0.00613000	-8.58497600
H	5.03131300	-0.97948400	-8.60617100
H	5.92254000	0.19009600	-9.58171100
H	6.37014500	-0.07879600	-7.89636700
C	-5.52575700	0.00613000	-8.58497600
H	-5.03131300	0.97948400	-8.60617100
H	-5.92254000	-0.19009600	-9.58171100
H	-6.37014500	0.07879600	-7.89636700
C	6.47678400	-2.24422300	8.80608000
H	6.86799200	-1.90765300	9.76681800
H	6.01699700	-3.22331100	8.95576800
H	7.32208000	-2.37563800	8.12738300
C	-6.47678400	2.24422300	8.80608000
H	-6.86799200	1.90765300	9.76681800
H	-6.01699700	3.22331100	8.95576800
H	-7.32208000	2.37563800	8.12738300

NDI6 IIa

C	0.87820300	-2.31526400	-0.60895600
C	-0.48043600	-2.19584500	-0.25898800
C	1.65020600	-3.34881600	-0.04402700
C	1.44386600	-1.39437100	-1.51356500
C	-1.24032100	-1.12859800	-0.78259200
C	-1.04392000	-3.11808200	0.64405500
C	1.08293000	-4.23848200	0.83580800
C	0.67803700	-0.38545300	-2.04360200
C	-0.67294800	-0.24433800	-1.66649300
C	-0.27725700	-4.12756100	1.17562900
C	3.09377700	-3.46511200	-0.38777200
C	2.89209100	-1.48410800	-1.85046000
C	-2.64064800	-0.92919300	-0.31883700
C	-2.48239800	-3.01079000	1.00809200
H	1.70044300	-5.02130100	1.25674900
H	1.13705600	0.31107300	-2.73494600
H	-1.27370300	0.57106000	-2.05090000
H	-0.73905400	-4.82784900	1.85965900
O	3.79321200	-4.33314500	0.07866000
O	3.41702900	-0.68057600	-2.58855400
O	-3.29484100	0.03394400	-0.66130600
O	-3.03192800	-3.84112800	1.69079100
N	3.61173100	-2.52988300	-1.28248900
N	-3.16947100	-1.88640300	0.53745100
C	5.05920700	-2.59625000	-1.53697600
C	-4.57230500	-1.72248100	0.95354400
C	5.87116300	-2.02837600	-0.37736900
H	5.31151600	-3.64419600	-1.69459100
H	5.24403500	-2.04092600	-2.45341800
C	-5.55105400	-2.15174400	-0.12865200
H	-4.70114800	-2.32093300	1.85154500
H	-4.71217200	-0.67718800	1.21542100
H	5.54583300	-1.00551000	-0.16889300
H	5.66217900	-2.61503000	0.51986100
H	-5.38898400	-1.54479500	-1.02395700
H	-5.36278700	-3.19608600	-0.39493800
C	0.48043600	2.19584500	0.25898800
C	-0.87820300	2.31526400	0.60895600
C	1.24032100	1.12859800	0.78259200
C	1.04392000	3.11808200	-0.64405500
C	-1.65020600	3.34881600	0.04402700
C	-1.44386600	1.39437100	1.51356500
C	0.67294800	0.24433800	1.66649300
C	0.27725700	4.12756100	-1.17562900

C	-1.08293000	4.23848200	-0.83580800
C	-0.67803700	0.38545300	2.04360200
C	2.64064800	0.92919300	0.31883700
C	2.48239800	3.01079000	-1.00809200
C	-3.09377700	3.46511200	0.38777200
C	-2.89209100	1.48410800	1.85046000
H	1.27370300	-0.57106000	2.05090000
H	0.73905400	4.82784900	-1.85965900
H	-1.70044300	5.02130100	-1.25674900
H	-1.13705600	-0.31107300	2.73494600
O	3.29484100	-0.03394400	0.66130600
O	3.03192800	3.84112800	-1.69079100
O	-3.79321200	4.33314500	-0.07866000
O	-3.41702900	0.68057600	2.58855400
N	3.16947100	1.88640300	-0.53745100
N	-3.61173100	2.52988300	1.28248900
C	4.57230500	1.72248100	-0.95354400
C	-5.05920700	2.59625000	1.53697600
C	5.55105400	2.15174400	0.12865200
H	4.71217200	0.67718800	-1.21542100
H	4.70114800	2.32093300	-1.85154500
C	-5.87116300	2.02837600	0.37736900
H	-5.24403500	2.04092600	2.45341800
H	-5.31151600	3.64419600	1.69459100
H	5.36278700	3.19608600	0.39493800
H	5.38898400	1.54479500	1.02395700
H	-5.66217900	2.61503000	-0.51986100
H	-5.54583300	1.00551000	0.16889300
C	6.98960100	1.98963100	-0.34988400
H	7.15019600	2.60864100	-1.23882000
H	7.15202500	0.95121300	-0.66422100
C	7.36494300	-2.06480500	-0.67932700
H	7.58407500	-1.42215200	-1.54012500
H	7.65229800	-3.08096300	-0.97152400
C	-6.98960100	-1.98963100	0.34988400
H	-7.15019600	-2.60864100	1.23882000
H	-7.15202500	-0.95121300	0.66422100
C	-7.36494300	2.06480500	0.67932700
H	-7.58407500	1.42215200	1.54012500
H	-7.65229800	3.08096300	0.97152400
C	8.01349800	2.36117600	0.71600600
H	7.84918100	1.74676500	1.60905000
H	7.85502100	3.40025500	1.02469900
C	8.21797300	-1.63205200	0.50825700
H	7.98316500	-2.26547500	1.37022500
H	7.95182400	-0.60856100	0.79998000

C	-8.01349800	-2.36117600	-0.71600600
H	-7.84918100	-1.74676500	-1.60905000
H	-7.85502100	-3.40025500	-1.02469900
C	-8.21797300	1.63205200	-0.50825700
H	-7.98316500	2.26547500	-1.37022500
H	-7.95182400	0.60856100	-0.79998000
C	9.45284500	2.18596900	0.24513900
H	9.60633600	2.76410700	-0.67092000
H	9.61325000	1.13679000	-0.02323400
C	9.71468600	-1.70300600	0.22488900
H	9.97690200	-2.72469700	-0.06580000
H	9.94910400	-1.07049500	-0.63735000
C	-9.45284500	-2.18596900	-0.24513900
H	-9.60633600	-2.76410700	0.67092000
H	-9.61325000	-1.13679000	0.02323400
C	-9.71468600	1.70300600	-0.22488900
H	-9.94910400	1.07049500	0.63735000
H	-9.97690200	2.72469700	0.06580000
C	10.46881700	2.61356000	1.29825600
H	11.49200200	2.43037400	0.96780700
H	10.37206600	3.67835000	1.52006000
H	10.31619900	2.06840800	2.23243900
C	10.55373900	-1.27712900	1.42406300
H	10.36923600	-1.93213700	2.27797500
H	11.62102700	-1.30732200	1.20134300
H	10.30407300	-0.25840600	1.72912200
C	-10.46881700	-2.61356000	-1.29825600
H	-10.37206600	-3.67835000	-1.52006000
H	-11.49200200	-2.43037400	-0.96780700
H	-10.31619900	-2.06840800	-2.23243900
C	-10.55373900	1.27712900	-1.42406300
H	-10.30407300	0.25840600	-1.72912200
H	-11.62102700	1.30732200	-1.20134300
H	-10.36923600	1.93213700	-2.27797500

NDI6 IIb

O	-3.84360900	-1.10547800	-2.51853000
C	-3.24978400	-1.66158800	-1.62248300
N	-3.89543200	-2.56118600	-0.78164700
C	-3.32770000	-3.14810200	0.35369700
O	-3.99116300	-3.81987400	1.10540800
C	-1.86521400	-2.94803800	0.55119300
C	-1.14318200	-2.13287400	-0.34372100
C	-1.79338700	-1.44456000	-1.38827900
C	-1.08890600	-0.59021900	-2.20224600
C	0.28878200	-0.39126600	-1.99571800

C	-5.30184200	-2.87818200	-1.07353900
C	-6.28079300	-1.86530600	-0.49470400
C	-7.71932300	-2.36011800	-0.60335300
H	-1.61481000	-0.06877400	-2.99317900
H	0.84951200	0.29975600	-2.61356800
H	-5.48810300	-3.86423000	-0.65305200
H	-5.39953700	-2.92278000	-2.15726300
H	-6.02659100	-1.68150000	0.55422900
H	-6.17756000	-0.92155900	-1.03568000
H	-7.82462700	-3.29377200	-0.04085900
H	-7.94525900	-2.59811700	-1.64871300
O	2.95412100	-3.02292300	1.97841500
C	2.34598000	-2.43329500	1.11707700
N	2.99296400	-1.52663600	0.27504800
C	2.38670700	-0.80472500	-0.74885400
O	3.00966400	0.00513100	-1.40158900
C	0.94272800	-1.06192900	-0.99072300
C	0.23926600	-1.94720900	-0.14913600
C	0.88409000	-2.61536200	0.91133300
C	0.16477900	-3.41521900	1.76548300
C	-1.22277700	-3.57719700	1.58838400
C	4.42673900	-1.30253600	0.49908400
C	5.29880700	-2.31031100	-0.23627000
C	6.76579700	-1.91117400	-0.12559900
H	0.68388700	-3.91372000	2.57413400
H	-1.80128500	-4.19587300	2.26207300
H	4.63887800	-0.29962700	0.13949100
H	4.60011400	-1.33648900	1.57307600
H	5.00471100	-2.33734200	-1.28968800
H	5.13823200	-3.30754500	0.18160900
H	6.89971400	-0.92657100	-0.58710800
H	7.03261600	-1.79358900	0.93137900
O	-2.95412100	3.02292300	-1.97841500
C	-2.34598000	2.43329500	-1.11707700
N	-2.99296400	1.52663600	-0.27504800
C	-2.38670700	0.80472500	0.74885400
O	-3.00966400	-0.00513100	1.40158900
C	-0.94272800	1.06192900	0.99072300
C	-0.23926600	1.94720900	0.14913600
C	-0.88409000	2.61536200	-0.91133300
C	-0.16477900	3.41521900	-1.76548300
C	1.22277700	3.57719700	-1.58838400
C	-4.42673900	1.30253600	-0.49908400
C	-5.29880700	2.31031100	0.23627000
C	-6.76579700	1.91117400	0.12559900
H	-0.68388700	3.91372000	-2.57413400

H	1.80128500	4.19587300	-2.26207300
H	-4.63887800	0.29962700	-0.13949100
H	-4.60011400	1.33648900	-1.57307600
H	-5.00471100	2.33734200	1.28968800
H	-5.13823200	3.30754500	-0.18160900
H	-6.89971400	0.92657100	0.58710800
H	-7.03261600	1.79358900	-0.93137900
O	3.84360900	1.10547800	2.51853000
C	3.24978400	1.66158800	1.62248300
N	3.89543200	2.56118600	0.78164700
C	3.32770000	3.14810200	-0.35369700
O	3.99116300	3.81987400	-1.10540800
C	1.86521400	2.94803800	-0.55119300
C	1.14318200	2.13287400	0.34372100
C	1.79338700	1.44456000	1.38827900
C	1.08890600	0.59021900	2.20224600
C	-0.28878200	0.39126600	1.99571800
C	5.30184200	2.87818200	1.07353900
C	6.28079300	1.86530600	0.49470400
C	7.71932300	2.36011800	0.60335300
H	1.61481000	0.06877400	2.99317900
H	-0.84951200	-0.29975600	2.61356800
H	5.48810300	3.86423000	0.65305200
H	5.39953700	2.92278000	2.15726300
H	6.02659100	1.68150000	-0.55422900
H	6.17756000	0.92155900	1.03568000
H	7.82462700	3.29377200	0.04085900
H	7.94525900	2.59811700	1.64871300
C	-8.73270100	-1.34244300	-0.09051700
H	-8.63491500	-0.41090000	-0.65967300
H	-8.49721900	-1.09040100	0.95026200
C	-7.72392400	2.90026300	0.77746600
H	-7.62978000	3.87772500	0.29255900
H	-7.44125400	3.04526600	1.82585100
C	8.73270100	1.34244300	0.09051700
H	8.63491500	0.41090000	0.65967300
H	8.49721900	1.09040100	-0.95026200
C	7.72392400	-2.90026300	-0.77746600
H	7.62978000	-3.87772500	-0.29255900
H	7.44125400	-3.04526600	-1.82585100
C	10.17355900	1.83471700	0.17514200
H	10.40629600	2.08488400	1.21457900
H	10.27062800	2.76339900	-0.39504300
C	9.17322900	-2.43056800	-0.70597300
H	9.25521900	-1.45498800	-1.19513100
H	9.44508500	-2.26838000	0.34232700

C	-10.17355900	-1.83471700	-0.17514200
H	-10.40629600	-2.08488400	-1.21457900
H	-10.27062800	-2.76339900	0.39504300
C	-9.17322900	2.43056800	0.70597300
H	-9.44508500	2.26838000	-0.34232700
H	-9.25521900	1.45498800	1.19513100
C	11.17287900	0.80563200	-0.34205200
H	12.19831000	1.16994800	-0.27046600
H	11.10430700	-0.12336000	0.22844000
H	10.97513400	0.56433200	-1.38900100
C	10.14990800	-3.41023600	-1.34507900
H	11.17644400	-3.04550200	-1.28721900
H	10.10975800	-4.38118800	-0.84708000
H	9.90879600	-3.56731800	-2.39846200
C	-10.14990800	3.41023600	1.34507900
H	-11.17644400	3.04550200	1.28721900
H	-9.90879600	3.56731800	2.39846200
H	-10.10975800	4.38118800	0.84708000
C	-11.17287900	-0.80563200	0.34205200
H	-11.10430700	0.12336000	-0.22844000
H	-12.19831000	-1.16994800	0.27046600
H	-10.97513400	-0.56433200	1.38900100

NDI7 Ib

O	-1.62026700	-0.00361300	-4.19853500
O	-1.43989700	4.01506200	-2.10605100
O	-1.46073400	-3.24288500	2.04105600
O	-1.59128800	0.78038400	4.13555400
N	-1.61736700	2.01343400	-3.15771900
N	-1.46639100	-1.23146100	3.09232600
C	-1.61736700	0.61973000	-3.16241900
C	-1.61856300	-0.05841800	-1.83660500
C	-1.63496400	0.71294100	-0.65643900
C	-1.61906100	2.12215800	-0.70221400
C	-1.54950800	2.81421600	-2.01807300
C	-1.60521400	-1.43128800	-1.77123000
H	-1.59845700	-2.00034000	-2.69302500
C	-1.57726200	-2.07913200	-0.52237800
H	-1.54165900	-3.15985900	-0.45935800
C	-1.58493600	-1.34518300	0.63989600
C	-1.61742900	0.06316400	0.59275900
C	-1.61736700	0.83547500	1.77293700
C	-1.62810200	2.20816600	1.70545800
H	-1.61691100	2.77846900	2.62574300
C	-1.62224000	2.85765800	0.45783500
H	-1.59531700	3.93774400	0.39548000

C	-1.50931700	-2.03740700	1.95421100
C	-1.56971700	0.15865900	3.09774700
C	-1.56928900	2.69356100	-4.46129300
H	-2.10405800	3.63493900	-4.35096700
H	-2.09466300	2.05676300	-5.16961400
C	-0.13579700	2.93219300	-4.91435200
H	0.35565800	3.58773700	-4.18835900
H	0.39623100	1.97674600	-4.92277900
C	-0.08136000	3.56990600	-6.29763500
H	-0.63692900	4.51404400	-6.29074200
H	-0.58507400	2.91553700	-7.01708900
C	-1.36789400	-1.90649600	4.39576600
H	-0.82497900	-1.23794500	5.05981100
H	-0.77589300	-2.80660100	4.24271600
C	-2.74217600	-2.24751500	4.95461000
H	-3.26944400	-2.88491400	4.23910000
H	-3.31801300	-1.32432300	5.06600700
C	-2.63812000	-2.95744500	6.29943800
H	-2.08963200	-2.32325200	7.00390900
H	-2.04958100	-3.87355000	6.18176800
O	1.59128800	-0.78038400	4.13555400
O	1.46073400	3.24288500	2.04105600
O	1.43989700	-4.01506200	-2.10605100
O	1.62026700	0.00361300	-4.19853500
N	1.46639100	1.23146100	3.09232600
N	1.61736700	-2.01343400	-3.15771900
C	1.56971700	-0.15865900	3.09774700
C	1.61736700	-0.83547500	1.77293700
C	1.61742900	-0.06316400	0.59275900
C	1.58493600	1.34518300	0.63989600
C	1.50931700	2.03740700	1.95421100
C	1.62810200	-2.20816600	1.70545800
H	1.61691100	-2.77846900	2.62574300
C	1.62224000	-2.85765800	0.45783500
H	1.59531700	-3.93774400	0.39548000
C	1.61906100	-2.12215800	-0.70221400
C	1.63496400	-0.71294100	-0.65643900
C	1.61856300	0.05841800	-1.83660500
C	1.60521400	1.43128800	-1.77123000
H	1.59845700	2.00034000	-2.69302500
C	1.57726200	2.07913200	-0.52237800
H	1.54165900	3.15985900	-0.45935800
C	1.54950800	-2.81421600	-2.01807300
C	1.61736700	-0.61973000	-3.16241900
C	1.36789400	1.90649600	4.39576600
H	0.77589300	2.80660100	4.24271600

H	0.82497900	1.23794500	5.05981100
C	2.74217600	2.24751500	4.95461000
H	3.26944400	2.88491400	4.23910000
H	3.31801300	1.32432300	5.06600700
C	2.63812000	2.95744500	6.29943800
H	2.04958100	3.87355000	6.18176800
H	2.08963200	2.32325200	7.00390900
C	1.56928900	-2.69356100	-4.46129300
H	2.09466300	-2.05676300	-5.16961400
H	2.10405800	-3.63493900	-4.35096700
C	0.13579700	-2.93219300	-4.91435200
H	-0.35565800	-3.58773700	-4.18835900
H	-0.39623100	-1.97674600	-4.92277900
C	0.08136000	-3.56990600	-6.29763500
H	0.58507400	-2.91553700	-7.01708900
H	0.63692900	-4.51404400	-6.29074200
C	1.34729100	3.82674900	-6.76536300
H	1.84816400	4.48750300	-6.04949700
H	1.90317800	2.88326700	-6.75661700
C	-4.00102700	-3.30530400	6.88828500
H	-4.55103400	-3.93430200	6.18008700
H	-4.58811400	-2.38835500	7.00743800
C	-1.34729100	-3.82674900	-6.76536300
H	-1.84816400	-4.48750300	-6.04949700
H	-1.90317800	-2.88326700	-6.75661700
C	4.00102700	3.30530400	6.88828500
H	4.55103400	3.93430200	6.18008700
H	4.58811400	2.38835500	7.00743800
C	1.41744900	4.44579100	-8.15676800
H	0.85564500	5.38644400	-8.16503200
H	0.91907300	3.78411900	-8.87377300
C	-1.41744900	-4.44579100	-8.15676800
H	-0.85564500	-5.38644400	-8.16503200
H	-0.91907300	-3.78411900	-8.87377300
C	3.90548800	4.02118100	8.23166300
H	3.35237000	3.39396400	8.93939700
H	3.31986100	4.93934300	8.11233800
C	-3.90548800	-4.02118100	8.23166300
H	-3.31986100	-4.93934300	8.11233800
H	-3.35237000	-3.39396400	8.93939700
C	2.84507300	4.70921300	-8.62467900
H	3.40471700	3.76914500	-8.61501900
H	3.34121800	5.37039500	-7.90787200
C	-2.84507300	-4.70921300	-8.62467900
H	-3.40471700	-3.76914500	-8.61501900
H	-3.34121800	-5.37039500	-7.90787200

C	5.26759300	4.36704700	8.82449000
H	5.85023700	3.44875900	8.94438900
H	5.81928500	4.99157000	8.11545900
C	-5.26759300	-4.36704700	8.82449000
H	-5.85023700	-3.44875900	8.94438900
H	-5.81928500	-4.99157000	8.11545900
C	-5.15832800	-5.08579400	10.16374000
H	-6.14022700	-5.32442500	10.57404200
H	-4.60259100	-6.01997400	10.05928500
H	-4.63145700	-4.46726400	10.89333100
C	5.15832800	5.08579400	10.16374000
H	4.63145700	4.46726400	10.89333100
H	6.14022700	5.32442500	10.57404200
H	4.60259100	6.01997400	10.05928500
C	2.90010800	5.32689500	-10.01751800
H	3.92692600	5.50950300	-10.33656400
H	2.43266900	4.66862700	-10.75282500
H	2.36737000	6.27988800	-10.04082100
C	-2.90010800	-5.32689500	-10.01751800
H	-3.92692600	-5.50950300	-10.33656400
H	-2.43266900	-4.66862700	-10.75282500
H	-2.36737000	-6.27988800	-10.04082100

NDI7 IIa

C	0.86729200	-2.31332000	-0.60783300
C	-0.48382800	-2.19080300	-0.23095900
C	1.65242900	-3.33861200	-0.04602700
C	1.41333000	-1.40169400	-1.53353800
C	-1.25497000	-1.12906000	-0.74932800
C	-1.02785800	-3.10356700	0.69326500
C	1.10442200	-4.21853500	0.85555800
C	0.63607600	-0.39872100	-2.05808300
C	-0.70664300	-0.25372900	-1.65388600
C	-0.24898700	-4.10573300	1.22102600
C	3.08877600	-3.45744300	-0.41830200
C	2.85401100	-1.49474600	-1.90033300
C	-2.64484100	-0.92481400	-0.25722700
C	-2.45927800	-2.99476700	1.08408700
H	1.73157300	-4.99532700	1.27340200
H	1.08031800	0.29082000	-2.76587600
H	-1.31527700	0.55802300	-2.03363900
H	-0.69597500	-4.79894900	1.92188900
O	3.79883300	-4.31936600	0.04342900
O	3.36208100	-0.70004300	-2.65951400
O	-3.30490000	0.03721600	-0.59172200
O	-2.99381100	-3.81960400	1.78505100

N	3.58659900	-2.53294200	-1.33520600
N	-3.15715300	-1.87595300	0.61565900
C	5.02686900	-2.60552300	-1.62519000
C	-4.55311100	-1.70959000	1.05435800
C	5.87418000	-2.03106800	-0.49410800
H	5.27190500	-3.65572700	-1.77966600
H	5.18940000	-2.05964500	-2.55150300
C	-5.54803100	-2.12307500	-0.01904000
H	-4.67150100	-2.31707700	1.94764500
H	-4.68443000	-0.66636700	1.32953700
H	5.56171200	-1.00411600	-0.28571000
H	5.68843600	-2.60861800	0.41399100
H	-5.39481300	-1.50724800	-0.90967400
H	-5.36724700	-3.16511700	-0.29929600
C	0.48382800	2.19080300	0.23095900
C	-0.86729200	2.31332000	0.60783300
C	1.25497000	1.12906000	0.74932800
C	1.02785800	3.10356700	-0.69326500
C	-1.65242900	3.33861200	0.04602700
C	-1.41333000	1.40169400	1.53353800
C	0.70664300	0.25372900	1.65388600
C	0.24898700	4.10573300	-1.22102600
C	-1.10442200	4.21853500	-0.85555800
C	-0.63607600	0.39872100	2.05808300
C	2.64484100	0.92481400	0.25722700
C	2.45927800	2.99476700	-1.08408700
C	-3.08877600	3.45744300	0.41830200
C	-2.85401100	1.49474600	1.90033300
H	1.31527700	-0.55802300	2.03363900
H	0.69597500	4.79894900	-1.92188900
H	-1.73157300	4.99532700	-1.27340200
H	-1.08031800	-0.29082000	2.76587600
O	3.30490000	-0.03721600	0.59172200
O	2.99381100	3.81960400	-1.78505100
O	-3.79883300	4.31936600	-0.04342900
O	-3.36208100	0.70004300	2.65951400
N	3.15715300	1.87595300	-0.61565900
N	-3.58659900	2.53294200	1.33520600
C	4.55311100	1.70959000	-1.05435800
C	-5.02686900	2.60552300	1.62519000
C	5.54803100	2.12307500	0.01904000
H	4.68443000	0.66636700	-1.32953700
H	4.67150100	2.31707700	-1.94764500
C	-5.87418000	2.03106800	0.49410800
H	-5.18940000	2.05964500	2.55150300
H	-5.27190500	3.65572700	1.77966600

H	5.36724700	3.16511700	0.29929600
H	5.39481300	1.50724800	0.90967400
H	-5.68843600	2.60861800	-0.41399100
H	-5.56171200	1.00411600	0.28571000
C	6.98075600	1.96108800	-0.47661600
H	7.14046900	2.60180800	-1.35012600
H	7.13232100	0.92985200	-0.81864100
C	7.35717600	-2.08363300	-0.84394100
H	7.55473600	-1.44597700	-1.71355800
H	7.62132800	-3.10391500	-1.14386600
C	-6.98075600	-1.96108800	0.47661600
H	-7.14046900	-2.60180800	1.35012600
H	-7.13232100	-0.92985200	0.81864100
C	-7.35717600	2.08363300	0.84394100
H	-7.55473600	1.44597700	1.71355800
H	-7.62132800	3.10391500	1.14386600
C	8.01284000	2.29530700	0.59371400
H	7.85281300	1.64994000	1.46522800
H	7.85543000	3.32261300	0.93985600
C	8.26053800	-1.66242300	0.31070400
H	8.03661200	-2.28093800	1.18608100
H	8.03473600	-0.62885400	0.59956600
C	-8.01284000	-2.29530700	-0.59371400
H	-7.85281300	-1.64994000	-1.46522800
H	-7.85543000	-3.32261300	-0.93985600
C	-8.26053800	1.66242300	-0.31070400
H	-8.03661200	2.28093800	-1.18608100
H	-8.03473600	0.62885400	-0.59956600
C	9.44932500	2.13929500	0.10995700
H	9.60859500	2.76819600	-0.77271600
H	9.61058300	1.10508300	-0.21834400
C	9.74196700	-1.78690700	-0.02746900
H	9.95926300	-2.82220000	-0.31302800
H	9.96920400	-1.17420200	-0.90799700
C	-9.44932500	-2.13929500	-0.10995700
H	-9.60859500	-2.76819600	0.77271600
H	-9.61058300	-1.10508300	0.21834400
C	-9.74196700	1.78690700	0.02746900
H	-9.96920400	1.17420200	0.90799700
H	-9.95926300	2.82220000	0.31302800
C	10.47846300	2.49862900	1.17660500
H	10.33411000	3.54078200	1.47688600
H	10.29524500	1.89491300	2.07131900
C	10.66164100	-1.38012300	1.11864500
H	10.40526800	-1.96000400	2.01025400
H	10.47815700	-0.33088500	1.37208800

C	-10.47846300	-2.49862900	-1.17660500
H	-10.33411000	-3.54078200	-1.47688600
H	-10.29524500	-1.89491300	-2.07131900
C	-10.66164100	1.38012300	-1.11864500
H	-10.47815700	0.33088500	-1.37208800
H	-10.40526800	1.96000400	-2.01025400
C	11.91111300	2.29091200	0.70010000
H	12.63503900	2.55585000	1.47164900
H	12.07934900	1.24672700	0.42756300
H	12.11985700	2.90147500	-0.18088300
C	12.13472900	-1.58227500	0.78075500
H	12.40377900	-1.03927100	-0.12827500
H	12.78468900	-1.23305500	1.58423000
H	12.35089500	-2.63852600	0.60758800
C	-11.91111300	-2.29091200	-0.70010000
H	-12.07934900	-1.24672700	-0.42756300
H	-12.63503900	-2.55585000	-1.47164900
H	-12.11985700	-2.90147500	0.18088300
C	-12.13472900	1.58227500	-0.78075500
H	-12.35089500	2.63852600	-0.60758800
H	-12.78468900	1.23305500	-1.58423000
H	-12.40377900	1.03927100	0.12827500

NDI7 IIb

O	-3.78371000	1.18803100	2.55963200
C	-3.19442500	1.72997500	1.65194300
N	-3.83738800	2.63706000	0.81723700
C	-3.27778400	3.20741800	-0.33038400
O	-3.94166600	3.88516800	-1.07637800
C	-1.82183600	2.98257600	-0.54872700
C	-1.09987000	2.15960900	0.33897800
C	-1.74565900	1.48690400	1.39639900
C	-1.04339700	0.62379500	2.20285300
C	0.32751700	0.40057200	1.97615600
C	-5.23466200	2.97685200	1.12705900
C	-6.23520900	1.97423600	0.56786600
C	-7.66623400	2.48772700	0.68894400
H	-1.56577400	0.11447700	3.00396800
H	0.88545400	-0.29786100	2.58822900
H	-5.41241300	3.96290700	0.70294900
H	-5.31640100	3.02948000	2.21173400
H	-5.99607700	1.77902600	-0.48256400
H	-6.13833600	1.03309500	1.11454300
H	-7.76712000	3.41840000	0.12081400
H	-7.87862000	2.73511900	1.73492000
O	2.97599900	2.97152700	-2.04859200

C	2.37134000	2.39610200	-1.17525900
N	3.01642000	1.48332400	-0.33836300
C	2.41326900	0.77380600	0.69619800
O	3.03167600	-0.04553700	1.34135500
C	0.97761500	1.05648900	0.95904600
C	0.27624200	1.95025300	0.12456100
C	0.91581500	2.60291400	-0.94862100
C	0.19693100	3.41086000	-1.79556400
C	-1.18491300	3.59647300	-1.59842800
C	4.44294700	1.23573700	-0.58354000
C	5.34358800	2.24135700	0.11999000
C	6.80079000	1.81112100	-0.00444700
H	0.71189700	3.89716400	-2.61422400
H	-1.76332800	4.22165100	-2.26617900
H	4.64679700	0.23543200	-0.21228400
H	4.59829000	1.25081500	-1.66076900
H	5.06568000	2.29441200	1.17682100
H	5.19567200	3.23311100	-0.31510500
H	6.92420600	0.83813000	0.48382600
H	7.04543200	1.65671800	-1.06206400
O	-2.97599900	-2.97152700	2.04859200
C	-2.37134000	-2.39610200	1.17525900
N	-3.01642000	-1.48332400	0.33836300
C	-2.41326900	-0.77380600	-0.69619800
O	-3.03167600	0.04553700	-1.34135500
C	-0.97761500	-1.05648900	-0.95904600
C	-0.27624200	-1.95025300	-0.12456100
C	-0.91581500	-2.60291400	0.94862100
C	-0.19693100	-3.41086000	1.79556400
C	1.18491300	-3.59647300	1.59842800
C	-4.44294700	-1.23573700	0.58354000
C	-5.34358800	-2.24135700	-0.11999000
C	-6.80079000	-1.81112100	0.00444700
H	-0.71189700	-3.89716400	2.61422400
H	1.76332800	-4.22165100	2.26617900
H	-4.64679700	-0.23543200	0.21228400
H	-4.59829000	-1.25081500	1.66076900
H	-5.06568000	-2.29441200	-1.17682100
H	-5.19567200	-3.23311100	0.31510500
H	-6.92420600	-0.83813000	-0.48382600
H	-7.04543200	-1.65671800	1.06206400
O	3.78371000	-1.18803100	-2.55963200
C	3.19442500	-1.72997500	-1.65194300
N	3.83738800	-2.63706000	-0.81723700
C	3.27778400	-3.20741800	0.33038400
O	3.94166600	-3.88516800	1.07637800

C	1.82183600	-2.98257600	0.54872700
C	1.09987000	-2.15960900	-0.33897800
C	1.74565900	-1.48690400	-1.39639900
C	1.04339700	-0.62379500	-2.20285300
C	-0.32751700	-0.40057200	-1.97615600
C	5.23466200	-2.97685200	-1.12705900
C	6.23520900	-1.97423600	-0.56786600
C	7.66623400	-2.48772700	-0.68894400
H	1.56577400	-0.11447700	-3.00396800
H	-0.88545400	0.29786100	-2.58822900
H	5.41241300	-3.96290700	-0.70294900
H	5.31640100	-3.02948000	-2.21173400
H	5.99607700	-1.77902600	0.48256400
H	6.13833600	-1.03309500	-1.11454300
H	7.76712000	-3.41840000	-0.12081400
H	7.87862000	-2.73511900	-1.73492000
C	-8.69362600	1.47571000	0.19320800
H	-8.59677300	0.54783400	0.76824100
H	-8.46859900	1.21444400	-0.84755600
C	-7.79077600	-2.80025700	-0.59863600
H	-7.70990400	-3.76176100	-0.08075600
H	-7.52770700	-2.98769200	-1.64525500
C	8.69362600	-1.47571000	-0.19320800
H	8.59677300	-0.54783400	-0.76824100
H	8.46859900	-1.21444400	0.84755600
C	7.79077600	2.80025700	0.59863600
H	7.70990400	3.76176100	0.08075600
H	7.52770700	2.98769200	1.64525500
C	10.13074800	-1.97575400	-0.28632800
H	10.36072500	-2.22986100	-1.32693800
H	10.23333100	-2.90335200	0.28741300
C	9.22707100	2.29441700	0.52054200
H	9.30186000	1.33948900	1.05333800
H	9.47723900	2.07707700	-0.52487100
C	-10.13074800	1.97575400	0.28632800
H	-10.36072500	2.22986100	1.32693800
H	-10.23333100	2.90335200	-0.28741300
C	-9.22707100	-2.29441700	-0.52054200
H	-9.47723900	-2.07707700	0.52487100
H	-9.30186000	-1.33948900	-1.05333800
C	11.14340200	-0.95321600	0.21862400
H	11.02697500	-0.02331700	-0.34662300
H	10.91176800	-0.70594200	1.25988000
C	10.25238300	3.26886500	1.08910200
H	10.19004200	4.21601400	0.54497100
H	9.99725300	3.49315200	2.12919100

C	-10.25238300	-3.26886500	-1.08910200
H	-9.99725300	-3.49315200	-2.12919100
H	-10.19004200	-4.21601400	-0.54497100
C	-11.14340200	0.95321600	-0.21862400
H	-11.02697500	0.02331700	0.34662300
H	-10.91176800	0.70594200	-1.25988000
C	-11.67440700	-2.72285500	-1.01463600
H	-12.39964700	-3.42982500	-1.41916400
H	-11.95454500	-2.50916000	0.01953000
H	-11.76172200	-1.79083100	-1.57766700
C	-12.58356400	1.44082600	-0.11516400
H	-13.28646100	0.68997700	-0.47910400
H	-12.84290100	1.66837500	0.92089800
H	-12.73023600	2.35048800	-0.70121300
C	11.67440700	2.72285500	1.01463600
H	12.39964700	3.42982500	1.41916400
H	11.76172200	1.79083100	1.57766700
H	11.95454500	2.50916000	-0.01953000
C	12.58356400	-1.44082600	0.11516400
H	12.84290100	-1.66837500	-0.92089800
H	13.28646100	-0.68997700	0.47910400
H	12.73023600	-2.35048800	0.70121300

NDI8 Ib

O	-0.97411300	1.29625900	-4.20959400
O	2.34751700	3.56220700	-2.11338900
O	-3.47173900	-0.78075400	2.02765900
O	-0.33488200	1.73954100	4.12498000
N	0.64154300	2.50227600	-3.16706300
N	-1.86812900	0.43213800	3.08035800
C	-0.47372900	1.66647000	-3.17291400
C	-1.01712400	1.25883900	-1.84771200
C	-0.41270400	1.73681900	-0.66695000
C	0.72317200	2.57113300	-0.71151300
C	1.32082400	2.92960900	-2.02663700
C	-2.10488100	0.42108700	-1.78360700
H	-2.55187200	0.07028400	-2.70601400
C	-2.60748000	0.00975000	-0.53520200
H	-3.44897500	-0.66939400	-0.47308900
C	-2.02777100	0.45835100	0.62767000
C	-0.92211600	1.33140700	0.58170800
C	-0.30612800	1.79557300	1.76248800
C	0.78406800	2.62979200	1.69618400
H	1.24506400	2.96409200	2.61704300
C	1.30791300	3.01570900	0.44915400
H	2.18768200	3.64295100	0.38789500

C	-2.53705500	-0.01788700	1.94157500
C	-0.81892700	1.34975200	3.08680000
C	1.21626700	2.87087800	-4.47017900
H	1.65134900	3.86216400	-4.35913300
H	0.39206300	2.91181000	-5.17890100
C	2.26417300	1.86396400	-4.92313700
H	3.08478900	1.86326800	-4.19856200
H	1.81646700	0.86616900	-4.92903400
C	2.80537400	2.19859500	-6.30813800
H	3.23255000	3.20736700	-6.30372400
H	1.97777000	2.21214300	-7.02541000
C	-2.34751700	-0.05438600	4.38328900
H	-1.48635700	-0.08663800	5.04666300
H	-2.71045800	-1.06856600	4.22904700
C	-3.44635800	0.83676800	4.94511800
H	-4.27630400	0.87100100	4.23360400
H	-3.05748800	1.85335500	5.05284300
C	-3.94208400	0.32752100	6.29355000
H	-3.10202000	0.27923500	6.99449000
H	-4.31286500	-0.69686400	6.18090900
O	0.33488200	-1.73954100	4.12498000
O	3.47173900	0.78075400	2.02765900
O	-2.34751700	-3.56220700	-2.11338900
O	0.97411300	-1.29625900	-4.20959400
N	1.86812900	-0.43213800	3.08035800
N	-0.64154300	-2.50227600	-3.16706300
C	0.81892700	-1.34975200	3.08680000
C	0.30612800	-1.79557300	1.76248800
C	0.92211600	-1.33140700	0.58170800
C	2.02777100	-0.45835100	0.62767000
C	2.53705500	0.01788700	1.94157500
C	-0.78406800	-2.62979200	1.69618400
H	-1.24506400	-2.96409200	2.61704300
C	-1.30791300	-3.01570900	0.44915400
H	-2.18768200	-3.64295100	0.38789500
C	-0.72317200	-2.57113300	-0.71151300
C	0.41270400	-1.73681900	-0.66695000
C	1.01712400	-1.25883900	-1.84771200
C	2.10488100	-0.42108700	-1.78360700
H	2.55187200	-0.07028400	-2.70601400
C	2.60748000	-0.00975000	-0.53520200
H	3.44897500	0.66939400	-0.47308900
C	-1.32082400	-2.92960900	-2.02663700
C	0.47372900	-1.66647000	-3.17291400
C	2.34751700	0.05438600	4.38328900
H	2.71045800	1.06856600	4.22904700

H	1.48635700	0.08663800	5.04666300
C	3.44635800	-0.83676800	4.94511800
H	4.27630400	-0.87100100	4.23360400
H	3.05748800	-1.85335500	5.05284300
C	3.94208400	-0.32752100	6.29355000
H	4.31286500	0.69686400	6.18090900
H	3.10202000	-0.27923500	6.99449000
C	-1.21626700	-2.87087800	-4.47017900
H	-0.39206300	-2.91181000	-5.17890100
H	-1.65134900	-3.86216400	-4.35913300
C	-2.26417300	-1.86396400	-4.92313700
H	-3.08478900	-1.86326800	-4.19856200
H	-1.81646700	-0.86616900	-4.92903400
C	-2.80537400	-2.19859500	-6.30813800
H	-1.97777000	-2.21214300	-7.02541000
H	-3.23255000	-3.20736700	-6.30372400
C	-5.04380500	1.20045900	6.88465600
H	-5.88303100	1.24861600	6.18251100
H	-4.67357400	2.22542200	6.99427800
C	5.04380500	-1.20045900	6.88465600
H	5.88303100	-1.24861600	6.18251100
H	4.67357400	-2.22542200	6.99427800
C	3.86137000	1.20335800	-6.77731100
H	4.69267800	1.19575200	-6.06402400
H	3.43456600	0.19492900	-6.76556800
C	-3.86137000	-1.20335800	-6.77731100
H	-4.69267800	-1.19575200	-6.06402400
H	-3.43456600	-0.19492900	-6.76556800
C	-4.39597300	-1.51338900	-8.17116500
H	-4.81828900	-2.52425000	-8.18131300
H	-3.56444300	-1.52019000	-8.88432500
C	4.39597300	1.51338900	-8.17116500
H	3.56444300	1.52019000	-8.88432500
H	4.81828900	2.52425000	-8.18131300
C	5.54338200	-0.69726100	8.23456800
H	4.70363200	-0.64948500	8.93637200
H	5.91125500	0.32873100	8.12490700
C	-5.54338200	0.69726100	8.23456800
H	-4.70363200	0.64948500	8.93637200
H	-5.91125500	-0.32873100	8.12490700
C	-5.45377800	-0.52072500	-8.64077400
H	-6.28634400	-0.51357100	-7.92861200
H	-5.03223600	0.49043900	-8.62981400
C	5.45377800	0.52072500	-8.64077400
H	6.28634400	0.51357100	-7.92861200
H	5.03223600	-0.49043900	-8.62981400

C	6.64643600	-1.56750200	8.82680600
H	7.48698700	-1.61502700	8.12569700
H	6.27964100	-2.59411800	8.93581700
C	-6.64643600	1.56750200	8.82680600
H	-7.48698700	1.61502700	8.12569700
H	-6.27964100	2.59411800	8.93581700
C	-5.98848700	-0.82761500	-10.03588900
H	-6.40909300	-1.83768500	-10.04476900
H	-5.15574400	-0.83469000	-10.74559400
C	5.98848700	0.82761500	-10.03588900
H	5.15574400	0.83469000	-10.74559400
H	6.40909300	1.83768500	-10.04476900
C	7.14672500	-1.06654800	10.17780600
H	6.30624900	-1.02018300	10.87679100
H	7.51207300	-0.04114800	10.06724800
C	-7.14672500	1.06654800	10.17780600
H	-6.30624900	1.02018300	10.87679100
H	-7.51207300	0.04114800	10.06724800
C	-8.24944800	1.94590100	10.75687100
H	-9.10921600	1.98345500	10.08463800
H	-8.59529300	1.57447700	11.72223800
H	-7.89557700	2.96921900	10.89866600
C	-7.04437300	0.17323800	-10.49201500
H	-7.41539700	-0.05996500	-11.49076000
H	-7.89707200	0.17591900	-9.80978200
H	-6.63548100	1.18558600	-10.51414600
C	7.04437300	-0.17323800	-10.49201500
H	6.63548100	-1.18558600	-10.51414600
H	7.41539700	0.05996500	-11.49076000
H	7.89707200	-0.17591900	-9.80978200
C	8.24944800	-1.94590100	10.75687100
H	8.59529300	-1.57447700	11.72223800
H	7.89557700	-2.96921900	10.89866600
H	9.10921600	-1.98345500	10.08463800

NDI8 IIa

C	0.81659800	-2.37488900	-0.42981000
C	-0.51954900	-2.20178800	-0.02037000
C	1.60845600	-3.36029500	0.19074300
C	1.34061100	-1.55382700	-1.44852300
C	-1.29615200	-1.17815900	-0.60289200
C	-1.04249100	-3.02569300	0.99540800
C	1.08085300	-4.15401300	1.18037300
C	0.55660200	-0.58965200	-2.03240900
C	-0.76974200	-0.39207100	-1.59800300
C	-0.25799900	-3.99153300	1.57934300

C	3.03051500	-3.52900100	-0.21560500
C	2.76667100	-1.69845400	-1.85381200
C	-2.66563700	-0.91231600	-0.08402500
C	-2.45791400	-2.86172600	1.42401400
H	1.71279900	-4.90184500	1.64166100
H	0.98336200	0.02941200	-2.81245800
H	-1.38255100	0.39127900	-2.02722300
H	-0.68868000	-4.61711800	2.35063600
O	3.74609300	-4.35816700	0.29477200
O	3.25625600	-0.98158000	-2.69803200
O	-3.32622000	0.02560200	-0.47958500
O	-2.97803600	-3.61502700	2.21125800
N	3.50800300	-2.69090600	-1.22213500
N	-3.15749800	-1.77583400	0.88626000
C	4.93812000	-2.80393500	-1.54752100
C	-4.53175900	-1.54548000	1.36215800
C	5.82296300	-2.14799800	-0.49233300
H	5.16950300	-3.86606600	-1.62234600
H	5.07767300	-2.33743600	-2.51998600
C	-5.57867600	-2.04478600	0.37810300
H	-4.62021200	-2.05829900	2.31586800
H	-4.63793700	-0.47829700	1.53534200
H	5.53297100	-1.10145900	-0.36842600
H	5.65128000	-2.63946500	0.46731200
H	-5.44856800	-1.53152400	-0.57935200
H	-5.43478300	-3.11601300	0.20509400
C	0.51954900	2.20178800	0.02037000
C	-0.81659800	2.37488900	0.42981000
C	1.29615200	1.17815900	0.60289200
C	1.04249100	3.02569300	-0.99540800
C	-1.60845600	3.36029500	-0.19074300
C	-1.34061100	1.55382700	1.44852300
C	0.76974200	0.39207100	1.59800300
C	0.25799900	3.99153300	-1.57934300
C	-1.08085300	4.15401300	-1.18037300
C	-0.55660200	0.58965200	2.03240900
C	2.66563700	0.91231600	0.08402500
C	2.45791400	2.86172600	-1.42401400
C	-3.03051500	3.52900100	0.21560500
C	-2.76667100	1.69845400	1.85381200
H	1.38255100	-0.39127900	2.02722300
H	0.68868000	4.61711800	-2.35063600
H	-1.71279900	4.90184500	-1.64166100
H	-0.98336200	-0.02941200	2.81245800
O	3.32622000	-0.02560200	0.47958500
O	2.97803600	3.61502700	-2.21125800

O	-3.74609300	4.35816700	-0.29477200
O	-3.25625600	0.98158000	2.69803200
N	3.15749800	1.77583400	-0.88626000
N	-3.50800300	2.69090600	1.22213500
C	4.53175900	1.54548000	-1.36215800
C	-4.93812000	2.80393500	1.54752100
C	5.57867600	2.04478600	-0.37810300
H	4.63793700	0.47829700	-1.53534200
H	4.62021200	2.05829900	-2.31586800
C	-5.82296300	2.14799800	0.49233300
H	-5.07767300	2.33743600	2.51998600
H	-5.16950300	3.86606600	1.62234600
H	5.43478300	3.11601300	-0.20509400
H	5.44856800	1.53152400	0.57935200
H	-5.65128000	2.63946500	-0.46731200
H	-5.53297100	1.10145900	0.36842600
C	6.98417300	1.78795100	-0.91055800
H	7.11603700	2.31183800	-1.86308900
H	7.09848400	0.71916600	-1.12866800
C	7.29570700	-2.25883400	-0.86913600
H	7.48381000	-1.70667200	-1.79764200
H	7.53639000	-3.30723200	-1.07993200
C	-6.98417300	-1.78795100	0.91055800
H	-7.11603700	-2.31183800	1.86308900
H	-7.09848400	-0.71916600	1.12866800
C	-7.29570700	2.25883400	0.86913600
H	-7.48381000	1.70667200	1.79764200
H	-7.53639000	3.30723200	1.07993200
C	8.07396800	2.22282800	0.06207300
H	7.93618600	1.70405100	1.01834500
H	7.96463100	3.29238300	0.27420100
C	8.23089000	-1.75122000	0.22317800
H	8.00934100	-2.27658000	1.15788700
H	8.03500800	-0.68952200	0.41526800
C	-8.07396800	-2.22282800	-0.06207300
H	-7.96463100	-3.29238300	-0.27420100
H	-7.93618600	-1.70405100	-1.01834500
C	-8.23089000	1.75122000	-0.22317800
H	-8.03500800	0.68952200	-0.41526800
H	-8.00934100	2.27658000	-1.15788700
C	-9.70213000	1.94641000	0.12543300
H	-9.92974300	1.42629000	1.06326800
H	-9.88636300	3.00969000	0.31504700
C	-9.47990400	-1.95047900	0.45942400
H	-9.60939000	-2.43978500	1.43081900
H	-9.59535200	-0.87508900	0.64076800

C	9.70213000	-1.94641000	-0.12543300
H	9.92974300	-1.42629000	-1.06326800
H	9.88636300	-3.00969000	-0.31504700
C	9.47990400	1.95047900	-0.45942400
H	9.60939000	2.43978500	-1.43081900
H	9.59535200	0.87508900	-0.64076800
C	10.57202200	2.42393600	0.49271300
H	10.41710600	1.97197300	1.47981700
H	10.48384000	3.50644600	0.63694900
C	10.64934900	-1.46088400	0.96467300
H	10.38975600	-1.94347300	1.91319200
H	10.50653500	-0.38467100	1.12089300
C	-10.64934900	1.46088400	-0.96467300
H	-10.50653500	0.38467100	-1.12089300
H	-10.38975600	1.94347300	-1.91319200
C	-10.57202200	-2.42393600	-0.49271300
H	-10.48384000	-3.50644600	-0.63694900
H	-10.41710600	-1.97197300	-1.47981700
C	-11.97728900	-2.09028000	-0.00465400
H	-12.12009100	-2.50552900	0.99742200
H	-12.06874900	-1.00436100	0.09820100
C	-12.11536300	1.73563400	-0.64691200
H	-12.36774900	1.27977300	0.31557100
H	-12.25817400	2.81275200	-0.52026300
C	12.11536300	-1.73563400	0.64691200
H	12.36774900	-1.27977300	-0.31557100
H	12.25817400	-2.81275200	0.52026300
C	11.97728900	2.09028000	0.00465400
H	12.12009100	2.50552900	-0.99742200
H	12.06874900	1.00436100	-0.09820100
C	13.06205500	2.61526800	0.93777500
H	12.92004700	2.23541300	1.95197000
H	14.05750500	2.31769100	0.60543200
H	13.03664900	3.70539900	0.99011400
C	13.05354400	-1.21312600	1.72765600
H	12.83245200	-1.67823700	2.69032700
H	14.09806500	-1.41566800	1.48824000
H	12.94060700	-0.13351100	1.84798500
C	-13.06205500	-2.61526800	-0.93777500
H	-13.03664900	-3.70539900	-0.99011400
H	-14.05750500	-2.31769100	-0.60543200
H	-12.92004700	-2.23541300	-1.95197000
C	-13.05354400	1.21312600	-1.72765600
H	-12.94060700	0.13351100	-1.84798500
H	-14.09806500	1.41566800	-1.48824000
H	-12.83245200	1.67823700	-2.69032700

NDI8 IIb

O	-2.55856500	1.40363100	-3.70267300
C	-1.61818800	1.88869300	-3.11464800
N	-0.75398200	2.77474200	-3.74757600
C	0.42624400	3.27865600	-3.19234900
O	1.19116600	3.94102300	-3.85050100
C	0.65763000	3.00452600	-1.74692200
C	-0.25481500	2.20298400	-1.03184000
C	-1.35148300	1.59574100	-1.67726900
C	-2.18480000	0.75094400	-0.98431500
C	-1.94630200	0.47975000	0.37592500
C	-1.06980500	3.16653500	-5.12975500
C	-0.56942900	2.17085100	-6.16818500
C	-0.67972500	2.73815400	-7.57982100
C	-0.24449500	1.74236000	-8.64990800
C	-0.31558100	2.31032500	-10.06297100
C	0.12693500	1.31336100	-11.12849500
C	0.05890600	1.87581400	-12.54468300
C	0.50109600	0.86739200	-13.59971700
H	-3.01643000	0.29262900	-1.50614300
H	-2.58034400	-0.20577800	0.92507300
H	-0.60593000	4.13809000	-5.28653000
H	-2.15219100	3.26839400	-5.19225700
H	0.47289800	1.91867500	-5.94760900
H	-1.15927100	1.25394900	-6.09562600
H	-0.06765900	3.64310200	-7.65444000
H	-1.71433000	3.04395000	-7.77113100
H	0.78145700	1.41611100	-8.44261600
H	-0.86906100	0.84413900	-8.58816500
H	0.30913200	3.20815100	-10.12473900
H	-1.34106800	2.63384100	-10.27269000
H	1.15340800	0.98961700	-10.91736200
H	-0.49342900	0.41139200	-11.06527000
H	0.68449300	2.77135800	-12.60505000
H	-0.96504000	2.20086300	-12.75265000
H	0.45224400	1.28875800	-14.60478100
H	1.52939200	0.54397800	-13.42134500
H	-0.13202500	-0.02240900	-13.57619300
O	2.22994300	2.79780100	3.02309400
C	1.32338700	2.27718800	2.41792700
N	0.45767000	1.38397500	3.05225300
C	-0.61505600	0.73544600	2.44741900
O	-1.28287100	-0.07521100	3.05318500
C	-0.89011300	1.07116100	1.02553900
C	-0.02781700	1.94620500	0.33413500

C	1.08356100	2.53298500	0.97235200
C	1.95482500	3.32093700	0.26069400
C	1.74444200	3.55249000	-1.11223500
C	0.71522000	1.08615300	4.46693200
C	0.07329700	2.09632100	5.40737800
C	0.20143300	1.61660000	6.84873400
C	-0.33731300	2.60068400	7.87993800
C	-0.26655000	2.03829200	9.29535400
C	-0.77177900	2.99679900	10.36706900
C	-0.72173400	2.38746400	11.76437100
C	-1.23352300	3.33022400	12.84671800
H	2.80273400	3.75567500	0.77423400
H	2.43099200	4.16194700	-1.68518100
H	0.30269200	0.09820200	4.65042200
H	1.79439800	1.04868200	4.60355800
H	-0.98428600	2.20570400	5.14949700
H	0.55142800	3.07106400	5.28085400
H	-0.33039900	0.66402300	6.95148100
H	1.25448000	1.40492100	7.06941000
H	-1.37658800	2.85001700	7.63902900
H	0.22833600	3.53702600	7.82507300
H	-0.84682700	1.10961800	9.34069700
H	0.76911900	1.75798700	9.52138400
H	-1.80168700	3.29119900	10.13652300
H	-0.17640700	3.91611000	10.34762000
H	-1.30882900	1.46359200	11.77148800
H	0.30897400	2.09239500	11.98902900
H	-1.20032800	2.86229600	13.83193100
H	-2.26702700	3.62309100	12.64922600
H	-0.63253200	4.24120100	12.88662900
O	-2.22994300	-2.79780100	-3.02309400
C	-1.32338700	-2.27718800	-2.41792700
N	-0.45767000	-1.38397500	-3.05225300
C	0.61505600	-0.73544600	-2.44741900
O	1.28287100	0.07521100	-3.05318500
C	0.89011300	-1.07116100	-1.02553900
C	0.02781700	-1.94620500	-0.33413500
C	-1.08356100	-2.53298500	-0.97235200
C	-1.95482500	-3.32093700	-0.26069400
C	-1.74444200	-3.55249000	1.11223500
C	-0.71522000	-1.08615300	-4.46693200
C	-0.07329700	-2.09632100	-5.40737800
C	-0.20143300	-1.61660000	-6.84873400
C	0.33731300	-2.60068400	-7.87993800
C	0.26655000	-2.03829200	-9.29535400
C	0.77177900	-2.99679900	-10.36706900

C	0.72173400	-2.38746400	-11.76437100
C	1.23352300	-3.33022400	-12.84671800
H	-2.80273400	-3.75567500	-0.77423400
H	-2.43099200	-4.16194700	1.68518100
H	-0.30269200	-0.09820200	-4.65042200
H	-1.79439800	-1.04868200	-4.60355800
H	0.98428600	-2.20570400	-5.14949700
H	-0.55142800	-3.07106400	-5.28085400
H	0.33039900	-0.66402300	-6.95148100
H	-1.25448000	-1.40492100	-7.06941000
H	1.37658800	-2.85001700	-7.63902900
H	-0.22833600	-3.53702600	-7.82507300
H	0.84682700	-1.10961800	-9.34069700
H	-0.76911900	-1.75798700	-9.52138400
H	1.80168700	-3.29119900	-10.13652300
H	0.17640700	-3.91611000	-10.34762000
H	1.30882900	-1.46359200	-11.77148800
H	-0.30897400	-2.09239500	-11.98902900
H	1.20032800	-2.86229600	-13.83193100
H	2.26702700	-3.62309100	-12.64922600
H	0.63253200	-4.24120100	-12.88662900
O	2.55856500	-1.40363100	3.70267300
C	1.61818800	-1.88869300	3.11464800
N	0.75398200	-2.77474200	3.74757600
C	-0.42624400	-3.27865600	3.19234900
O	-1.19116600	-3.94102300	3.85050100
C	-0.65763000	-3.00452600	1.74692200
C	0.25481500	-2.20298400	1.03184000
C	1.35148300	-1.59574100	1.67726900
C	2.18480000	-0.75094400	0.98431500
C	1.94630200	-0.47975000	-0.37592500
C	1.06980500	-3.16653500	5.12975500
C	0.56942900	-2.17085100	6.16818500
C	0.67972500	-2.73815400	7.57982100
C	0.24449500	-1.74236000	8.64990800
C	0.31558100	-2.31032500	10.06297100
C	-0.12693500	-1.31336100	11.12849500
C	-0.05890600	-1.87581400	12.54468300
C	-0.50109600	-0.86739200	13.59971700
H	3.01643000	-0.29262900	1.50614300
H	2.58034400	0.20577800	-0.92507300
H	0.60593000	-4.13809000	5.28653000
H	2.15219100	-3.26839400	5.19225700
H	-0.47289800	-1.91867500	5.94760900
H	1.15927100	-1.25394900	6.09562600
H	0.06765900	-3.64310200	7.65444000

H	1.71433000	-3.04395000	7.77113100
H	-0.78145700	-1.41611100	8.44261600
H	0.86906100	-0.84413900	8.58816500
H	-0.30913200	-3.20815100	10.12473900
H	1.34106800	-2.63384100	10.27269000
H	-1.15340800	-0.98961700	10.91736200
H	0.49342900	-0.41139200	11.06527000
H	-0.68449300	-2.77135800	12.60505000
H	0.96504000	-2.20086300	12.75265000
H	-0.45224400	-1.28875800	14.60478100
H	-1.52939200	-0.54397800	13.42134500
H	0.13202500	0.02240900	13.57619300

References

(1) Chen, S.-M.; Chang, L.-M.; Yang, X.-K.; Luo, T.; Xu, H.; Gu, Z.-G.; Zhang, J. Liquid-Phase Epitaxial Growth of Azapyrene-Based Chiral Metal–Organic Framework Thin Films for Circularly Polarized Luminescence. *ACS Appl. Mater. Interfaces* **2019**, *11* (34), 31421–31426. https://doi.org/10.1021/acsami.9b11872.

(2) Shukla, D.; Welter, T. R. N-Type Semiconductor Materials in Thin Film Transistors and Electronic Devices. US 2009/0256137 A1, October 15, 2009.

(3) Rademacher, A.; Märkle, S.; Langhals, H. Lösliche Perylen-Fluoreszenzfarbstoffe mit hoher Photostabilität. *Chem. Ber.* **1982**, *115* (8), 2927–2934. https://doi.org/10.1002/cber.19821150823.

(4) Johnson, R. L.; Schmidt-Rohr, K. Quantitative Solid-State 13C NMR with Signal Enhancement by Multiple Cross Polarization. *J. Magn. Reson.* **2014**, *239*, 44–49. https://doi.org/10.1016/j.jmr.2013.11.009.

(5) Mao, J.; Chen, N.; Cao, X. Characterization of Humic Substances by Advanced Solid State NMR Spectroscopy: Demonstration of a Systematic Approach. *Organic Geochemistry* **2011**, *42* (8), 891–902. https://doi.org/10.1016/j.orggeochem.2011.03.023.

(6) Opella, S. J.; Frey, M. H. Selection of Nonprotonated Carbon Resonances in Solid-State Nuclear Magnetic Resonance. *J. Am. Chem. Soc.* **1979**, *101* (19), 5854–5856. https://doi.org/10.1021/ja00513a079.

(7) Vinogradov, E.; Madhu, P. K.; Vega, S. High-Resolution Proton Solid-State NMR Spectroscopy by Phase-Modulated Lee–Goldburg Experiment. *Chem. Phys. Lett.* **1999**, *314* (5–6), 443–450. https://doi.org/10.1016/S0009-2614(99)01174-4.

(8) SAINT PLUS, 2001.

(9) SADABS, 2001.

(10) Sheldrick, G. M. Crystal Structure Refinement with SHELXL. *Acta Cryst C* **2015**, *71* (1), 3–8. https://doi.org/10.1107/S2053229614024218.

(11) Hübschle, C. B.; Sheldrick, G. M.; Dittrich, B. ShelXle: A Qt Graphical User Interface for SHELXL. *J Appl Cryst* **2011**, *44* (6), 1281–1284. https://doi.org/10.1107/S0021889811043202.

(12) Kovalevsky, A. Yu.; Ponomarev, I. I.; Shishkin, O. V. CCDC 155699: Experimental Crystal Structure Determination, 2001. https://doi.org/10.5517/CC570KY.

(13) Pandeeswar, M.; Khare, H.; Ramakumar, S.; Govindaraju, T. CCDC 937648: Experimental Crystal Structure Determination, 2014. https://doi.org/10.5517/CC10GPQW.

(14) Krishna, G. R.; Devarapalli, R.; Lal, G.; Reddy, C. M. Mechanically Flexible Organic Crystals Achieved by Introducing Weak Interactions in Structure: Supramolecular Shape Synthons. *J. Am. Chem. Soc.* **2016**, *138* (41), 13561–13567. https://doi.org/10.1021/jacs.6b05118.

(15) Alvey, P. M.; Reczek, J. J.; Lynch, V.; Iverson, B. L. CCDC 819749: Experimental Crystal Structure Determination, 2011. https://doi.org/10.5517/CCWJ0JX.

(16) Andric, G.; Boas, J. F.; Bond, A. M.; Fallon, G. D.; Ghiggino, K. P.; Hogan, C. F.; Hutchison, J. A.; Lee, M. A.-P.; Langford, S. J.; Pilbrow, J. R.; Troup, G. J.; Woodward, C. P. CCDC 238148: Experimental Crystal Structure Determination, 2005. https://doi.org/10.5517/CC7ZT66.

(17) Iengo, E.; Pantoş, G. D.; Sanders, J. K. M.; Orlandi, M.; Chiorboli, C.; Fracasso, S.; Scandola, F. A Fully Self-Assembled Non-Symmetric Triad for Photoinduced Charge Separation. *Chem. Sci.* **2011**, *2* (4), 676–685. https://doi.org/10.1039/C0SC00520G.

(18) Chlebosz, Dorota; Goldeman, Waldemar; Janus, Krzysztof; Szuster, Michał; Kiersnowski, Adam. CCDC 2242898: Experimental Crystal Structure Determination. https://doi.org/10.5517/CCDC.CSD.CC2F8XK6.

(19) Milita, Silvia; Liscio, Fabiola; Cowen, Lewis; Cavallini, Massimiliano; Drain, Ben A.; Degousée, Thibault; Luong, Sally; Fenwick, Oliver; Guagliardi, Antonietta; Schroeder, Bob C.; Masciocchi, Norberto. CCDC 1972223: Experimental Crystal Structure Determination. https://doi.org/10.5517/CCDC.CSD.CC24683R.

(20) Chlebosz, Dorota; Goldeman, Waldemar; Janus, Krzysztof; Szuster, Michał; Kiersnowski, Adam. CCDC 2242899: Experimental Crystal Structure Determination. https://doi.org/10.5517/CCDC.CSD.CC2F8XL7.

(21) Milita, Silvia; Liscio, Fabiola; Cowen, Lewis; Cavallini, Massimiliano; Drain, Ben A.; Degousée, Thibault; Luong, Sally; Fenwick, Oliver; Guagliardi, Antonietta; Schroeder, Bob C.; Masciocchi, Norberto. CCDC 1972224: Experimental Crystal Structure Determination. https://doi.org/10.5517/CCDC.CSD.CC24684S.

(22) Alvey, P. M.; Reczek, J. J.; Lynch, V.; Iverson, B. L. CCDC 819750: Experimental Crystal Structure Determination, 2011. https://doi.org/10.5517/CCWJ0KY.

(23) Lynch, D. E.; Hamilton, D. G. CCDC 230429: Experimental Crystal Structure Determination, 2004. https://doi.org/10.5517/CC7QS6X.

(24) Shokri, S.; Li, J.; Manna, M. K.; Wiederrecht, G. P.; Gosztola, D. J.; Ugrinov, A.; Jockusch, S.; Rogachev, A. Y.; Ayitou, A. J.-L. CCDC 1551374: Experimental Crystal Structure Determination, 2017. https://doi.org/10.5517/CCDC.CSD.CC1P2BBF.

(25) Shukla, D.; Rajeswaran, M.; Ahearn, W. G.; Meyer, D. M. N,N′-Bis(2,2,3,3,4,4,4-Hepta-fluoro-butyl)Naphthalene-1,4:5,8-Tetra-carboximide. *Acta Cryst E* **2008**, *64* (12), o2327–o2327. https://doi.org/10.1107/S1600536808036738.

(26) Spackman, P. R.; Turner, M. J.; McKinnon, J. J.; Wolff, S. K.; Grimwood, D. J.; Jayatilaka, D.; Spackman, M. A. CrystalExplorer: A Program for Hirshfeld Surface Analysis, Visualization and Quantitative Analysis of Molecular Crystals. *J. Appl. Crystallogr.* **2021**, *54* (3), 1006–1011. https://doi.org/10.1107/S1600576721002910.

(27) Spackman, M. A.; McKinnon, J. J. Fingerprinting Intermolecular Interactions in Molecular Crystals. *CrystEngComm.* **2002**, *4* (66), 378–392. https://doi.org/10.1039/B203191B.

(28) McKinnon, J. J.; Mitchell, A. S.; Spackman, M. A. Hirshfeld Surfaces: A New Tool for Visualising and Exploring Molecular Crystals. *Chem. Eur. J.* **1998**, *4* (11), 2136–2141. https://doi.org/10.1002/(SICI)1521-3765(19981102)4:11<2136::AID-CHEM2136>3.0.CO;2-G.

(29) Frisch, M. J. Gaussian 09, 2009.

(30) Zhao, Y.; Truhlar, D. G. The M06 Suite of Density Functionals for Main Group Thermochemistry, Thermochemical Kinetics, Noncovalent Interactions, Excited States, and Transition Elements: Two New Functionals and Systematic Testing of Four M06-Class Functionals and 12 Other Functionals. *Theor. Chem. Acc.* **2008**, *120* (1), 215–241. https://doi.org/10.1007/s00214-007-0310-x.

(31) Grimme, S.; Antony, J. A Consistent and Accurate Ab Initio Parametrization of Density Functional Dispersion Correction (DFT-D) for the 94 Elements H-Pu. *J. Chem. Phys.* **2010**, *132* (15), 154104. https://doi.org/10.1063/1.3382344.

(32) Schäfer, A.; Horn, H.; Ahlrichs, R. Fully Optimized Contracted Gaussian Basis Sets for Atoms Li to Kr. *J. Chem. Phys.* **1992**, *97* (4), 2571–2577. https://doi.org/10.1063/1.463096.
